# Supplementary material for: Efficient and selective N-alkylation of amines with alcohols catalysed by manganese pincer complexes
Source: Nat Commun. 2016 Oct 6;7:12641. doi: 10.1038/ncomms12641 (PMC5059641; doi:10.1038/ncomms12641)
Supplement: Supplementary Information — Supplementary Figures 1-64, Supplementary Tables 1-4, Supplementary Methods and Supplementary References [file ncomms12641-s1.pdf]

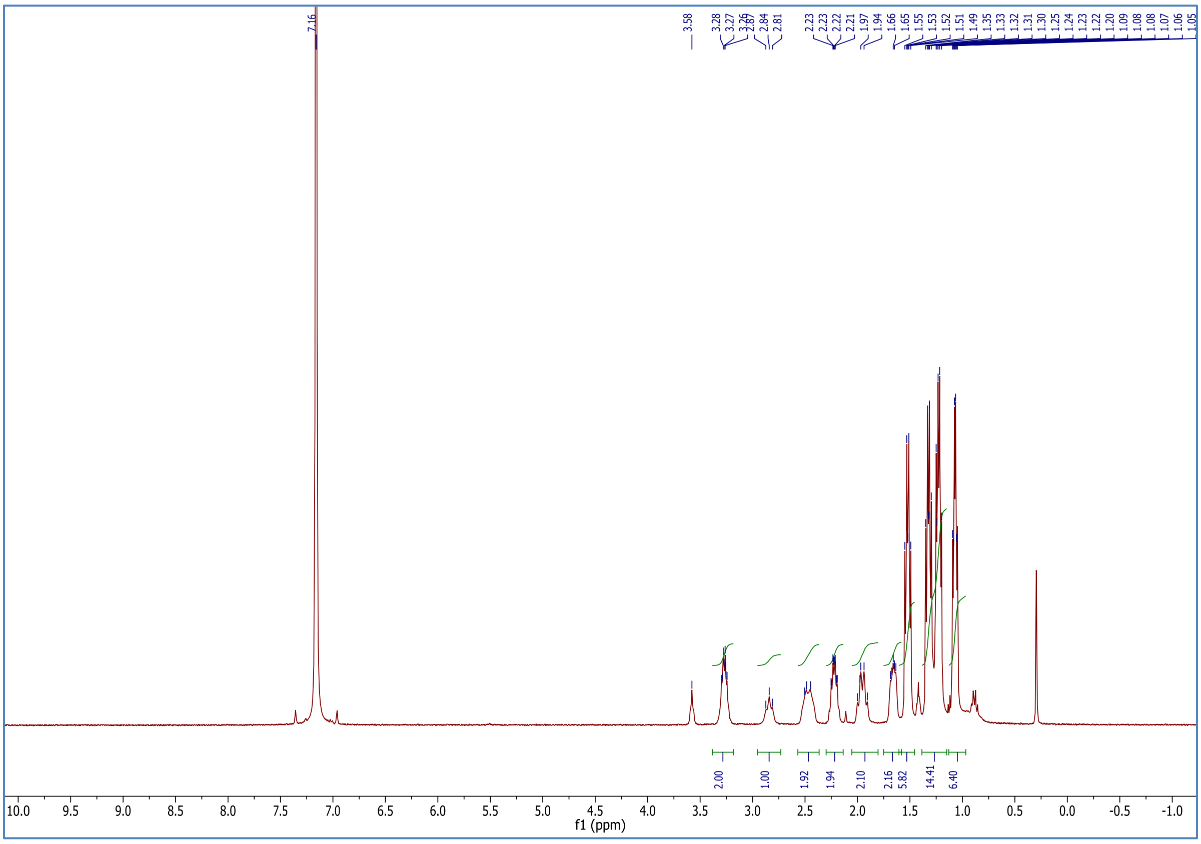

Supplementary Figure 1. ^{1}H NMR of **1**, C_6D_6.

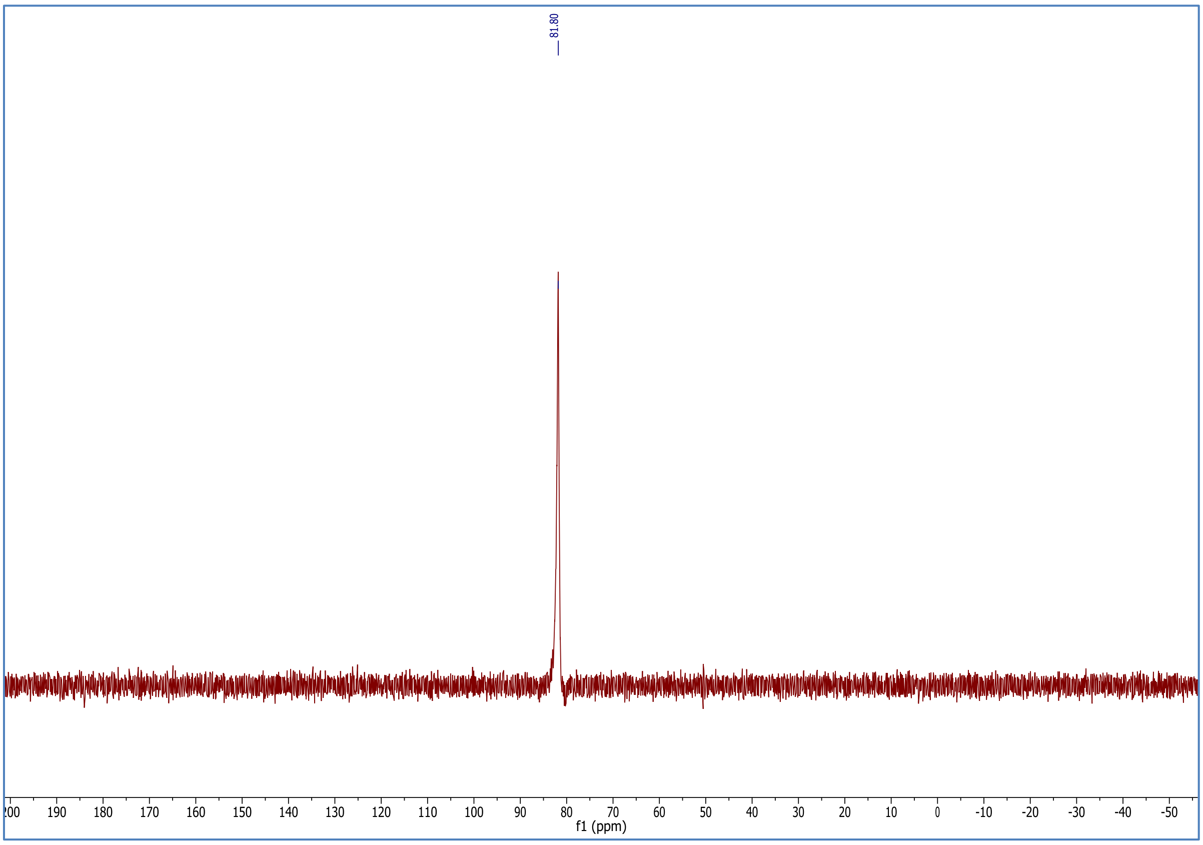

Supplementary Figure 2. ^{31}P NMR of **1**, C_6D_6.

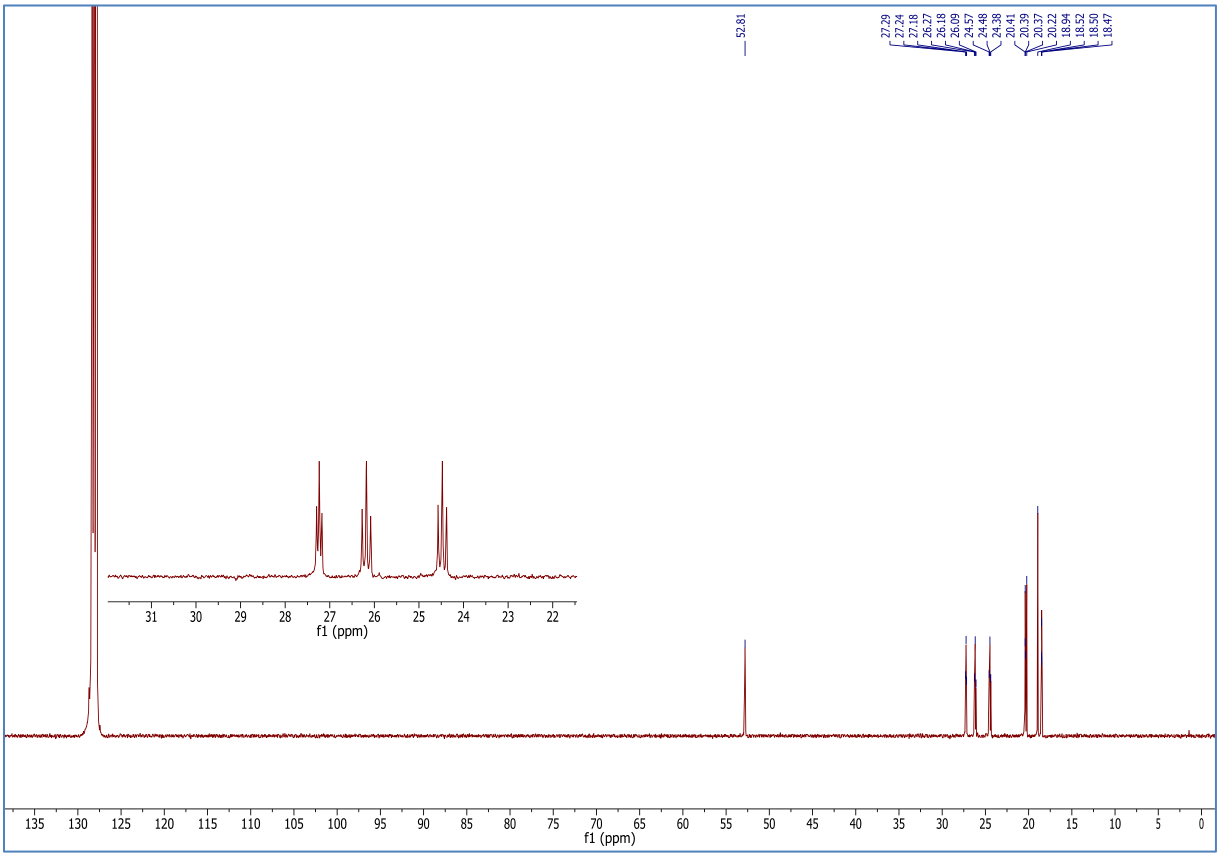

Supplementary Figure 3. ^{13}C NMR of **1**, C_6D_6.

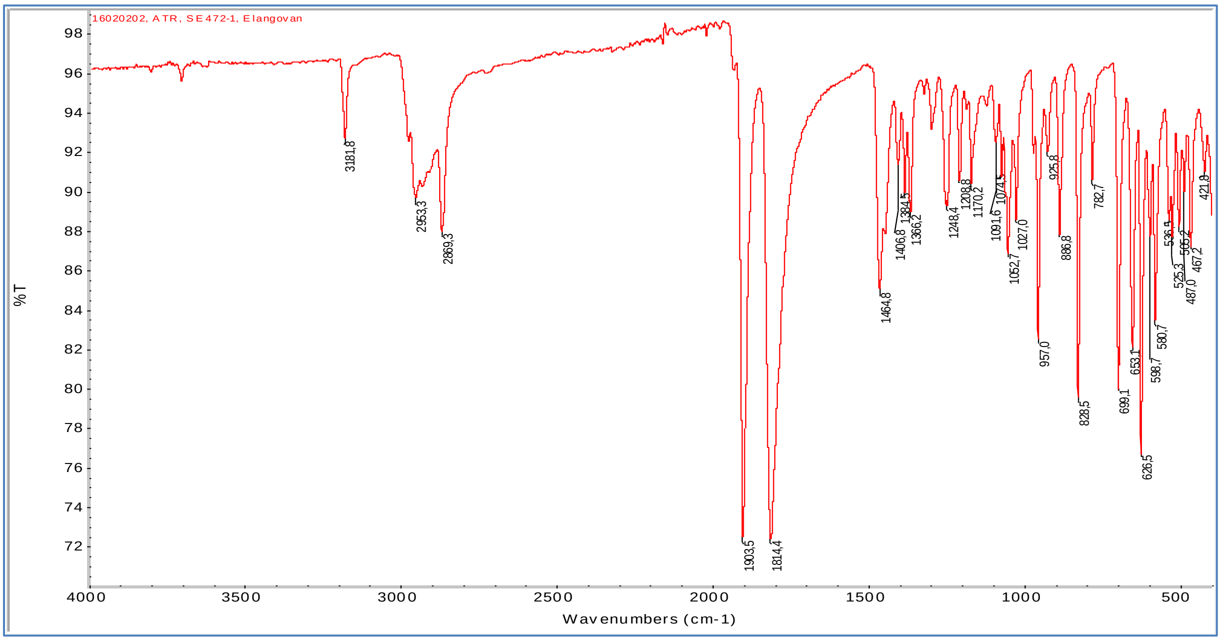

Supplementary Figure 4. IR (ATR) of **1**.

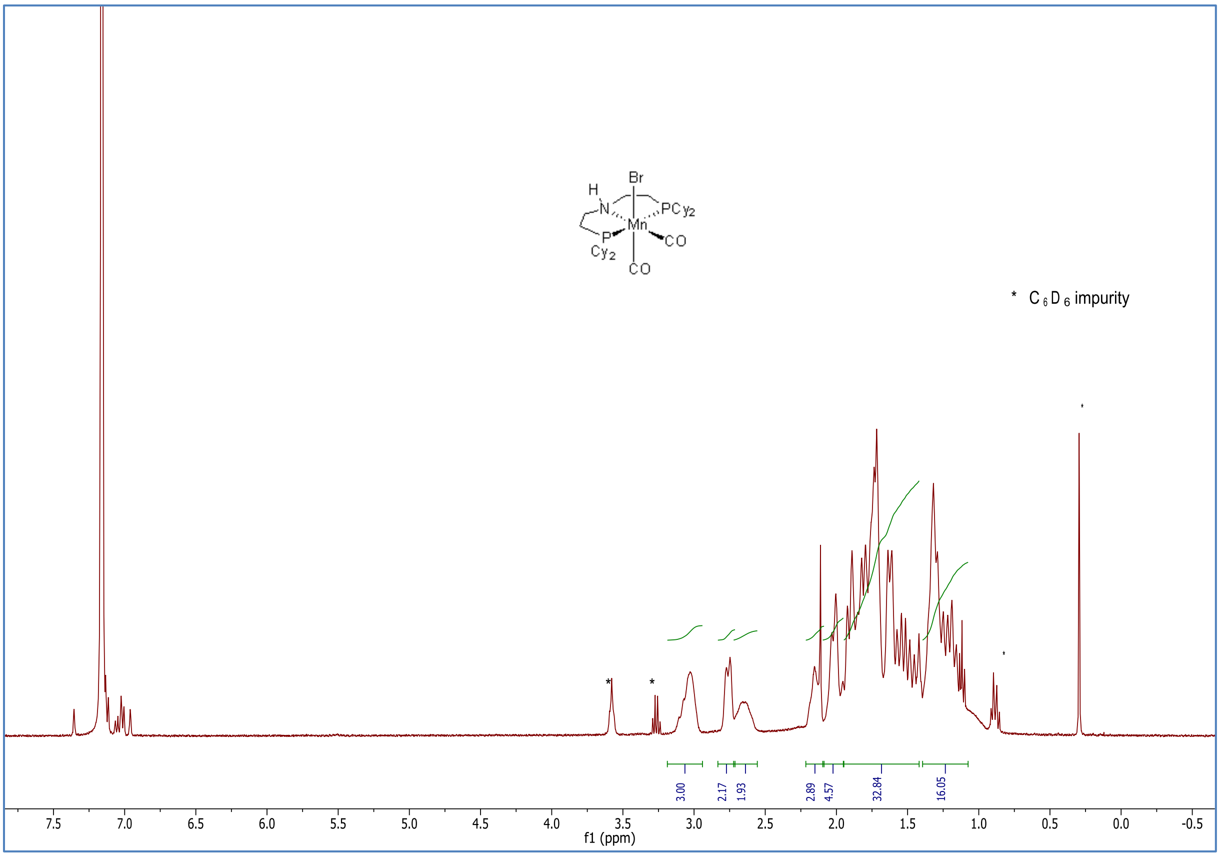

Supplemetary Figure 5. ^{1}H NMR of **2,** C_6D_6.

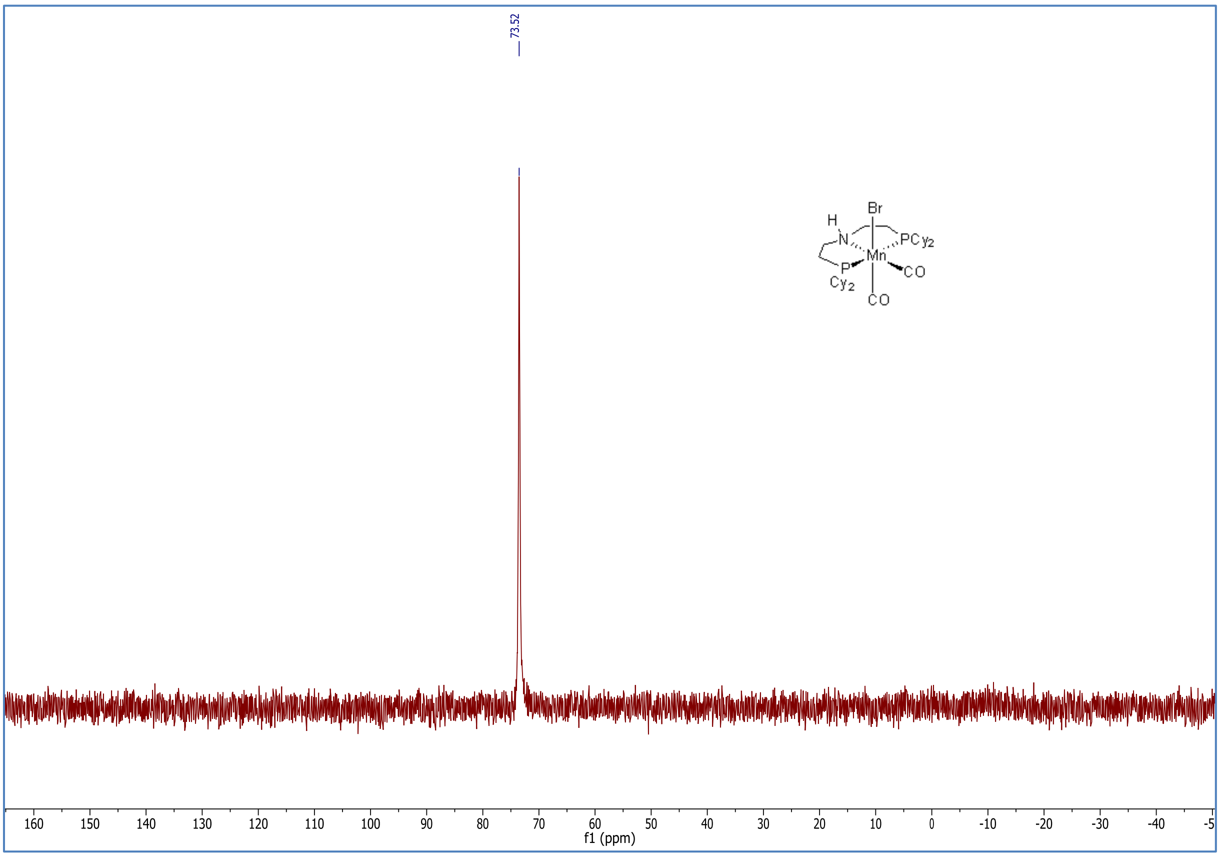

Supplementarry Figure 6. ^{31}P NMR of **2**, C_6D_6.

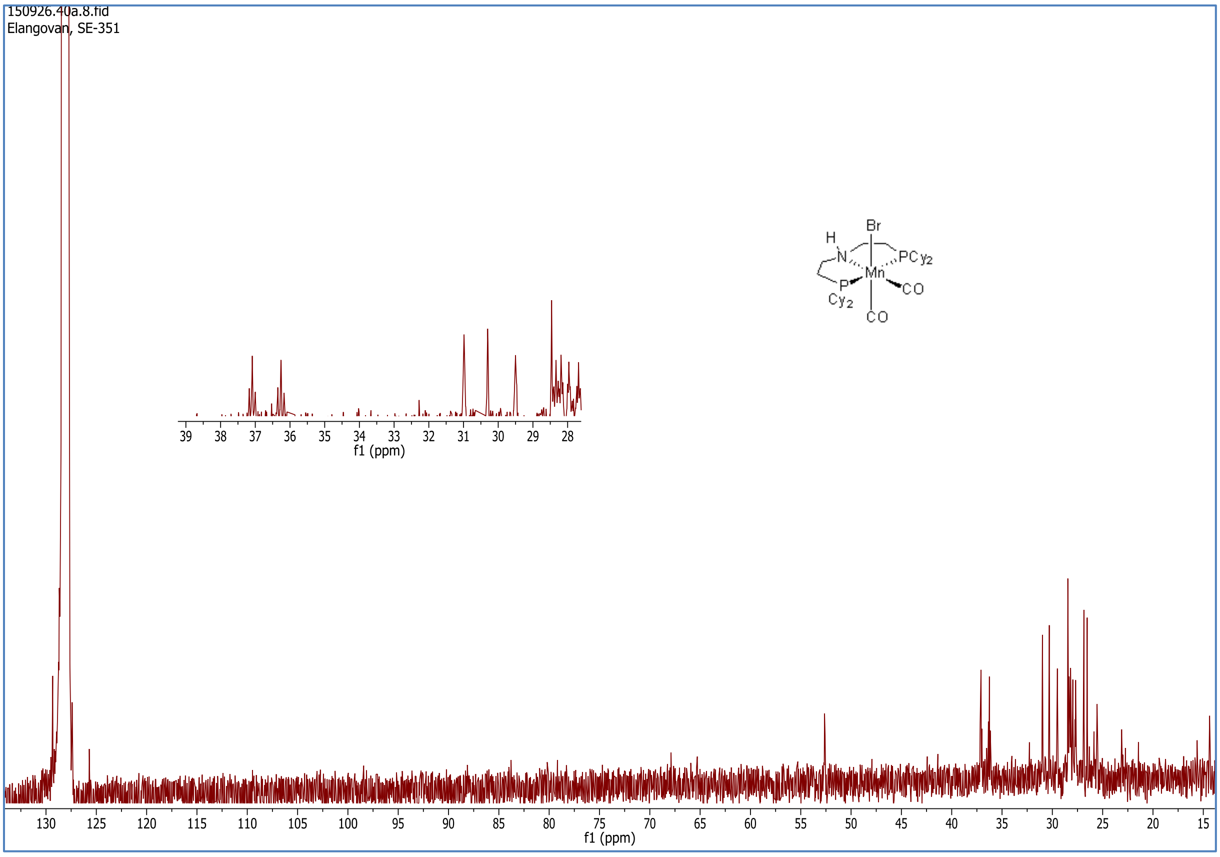

Supplementary Figure 7. $^{13}C\{^{1}H\}$ NMR of **2**, C_6D_6.

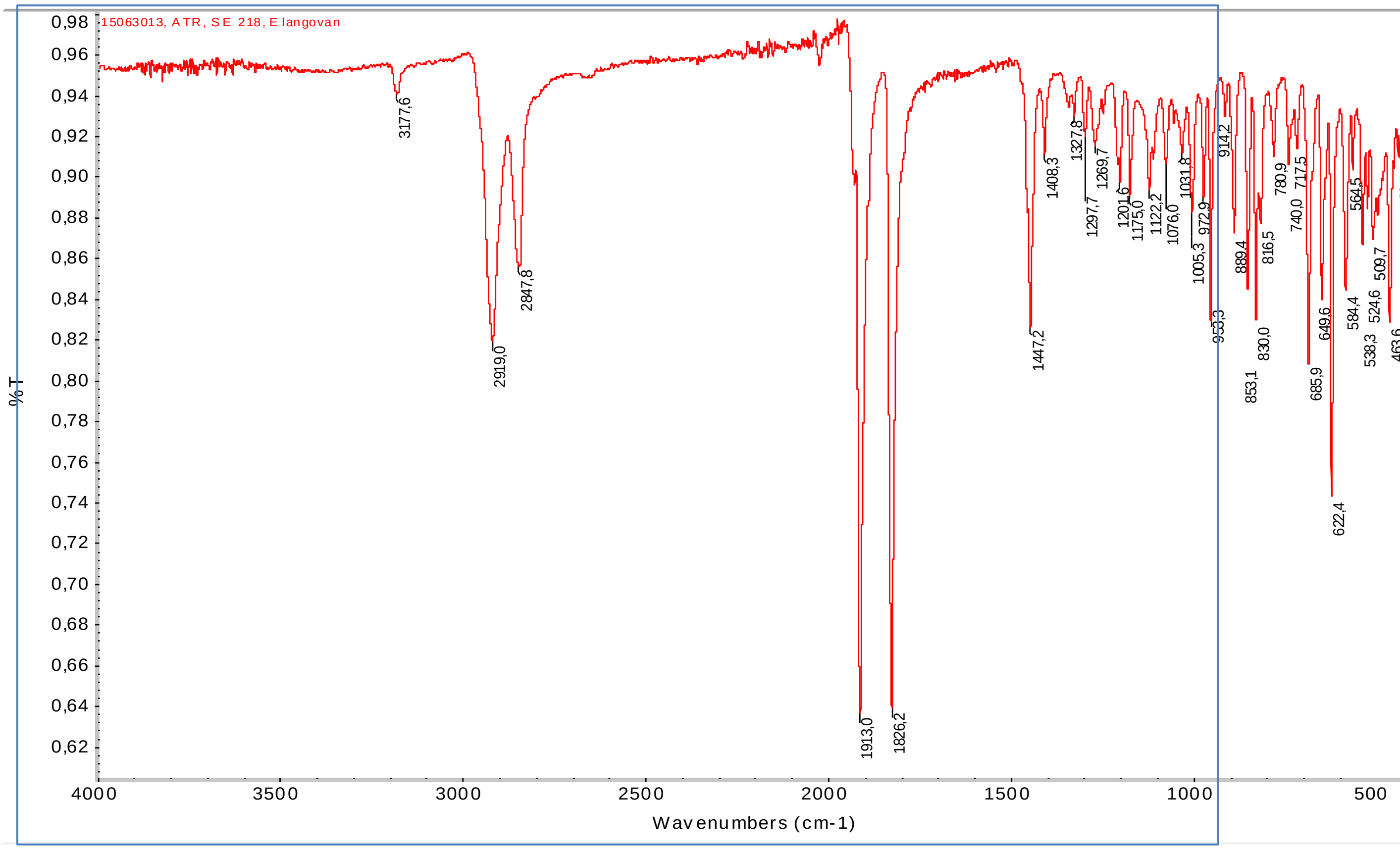

Supplementary Figure 8. IR (ATR) spectrum of **2**.

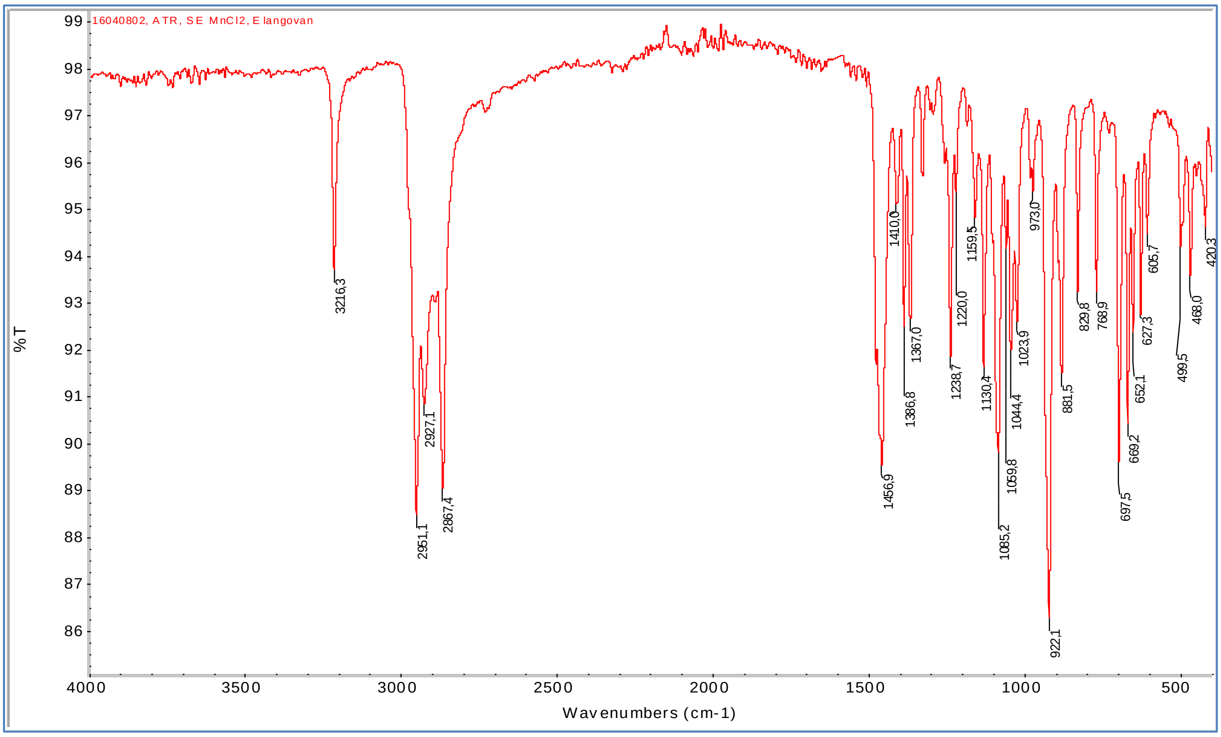

Supplementary Figure 9. IR spectrum of **3**

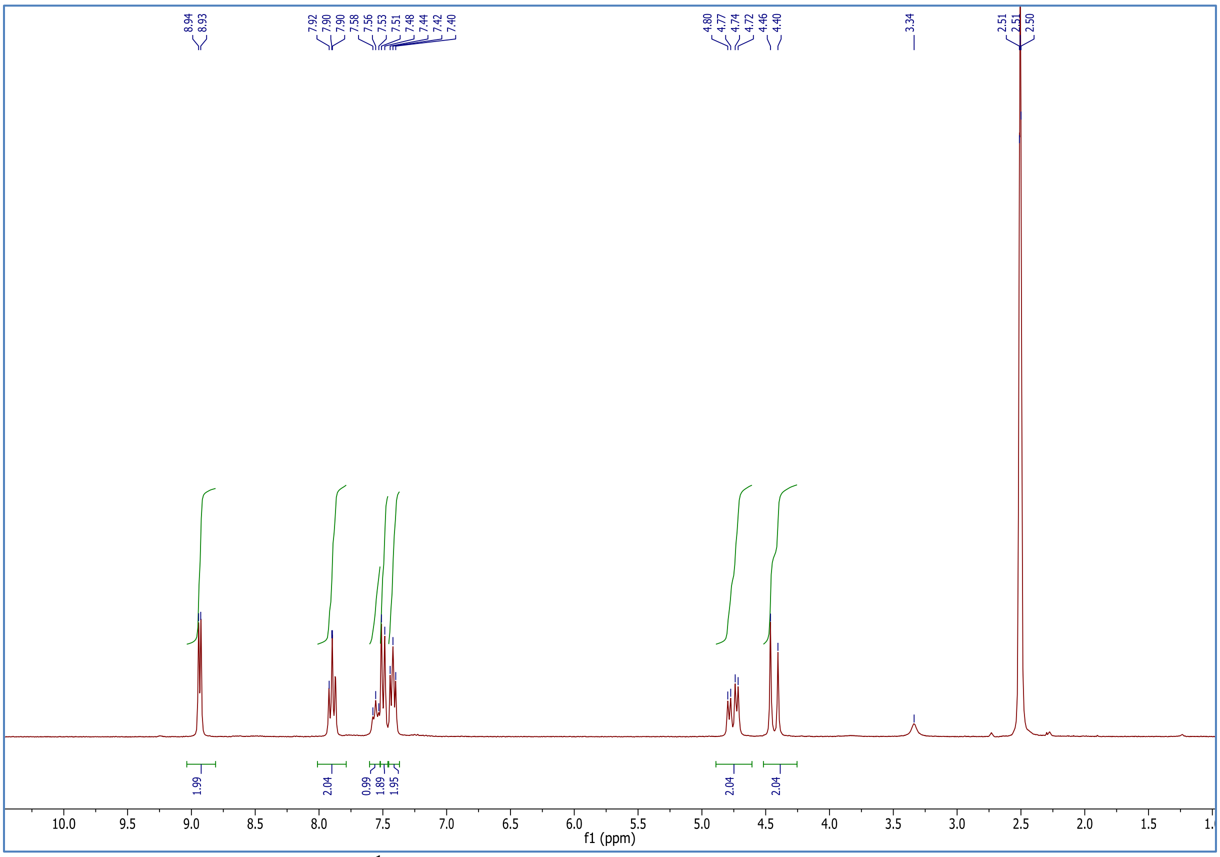

Supplementary Figure 10. ^{1}H NMR of **4,** DMSO d_6.

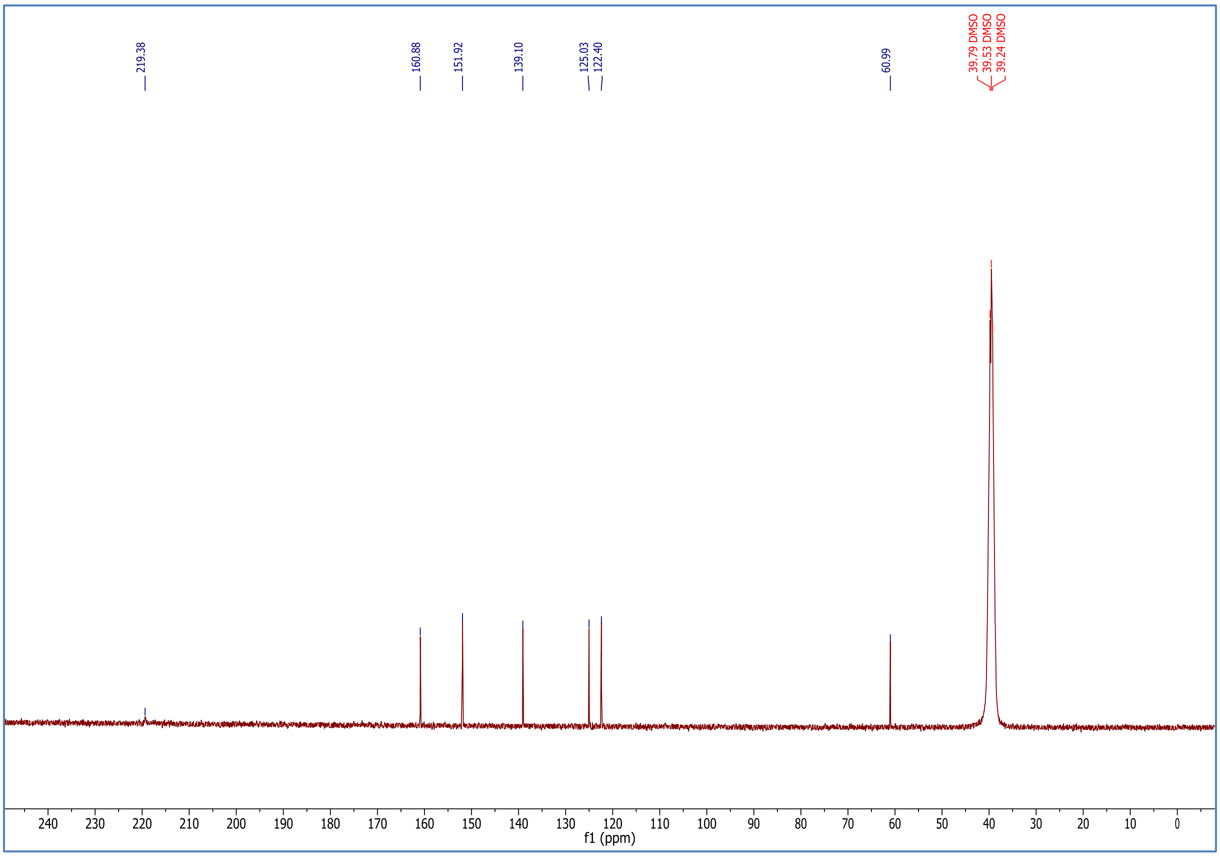

Supplementary Figure 11. $^{13}C\{^{1}H\}$ NMR of **4**, C_6D_6.

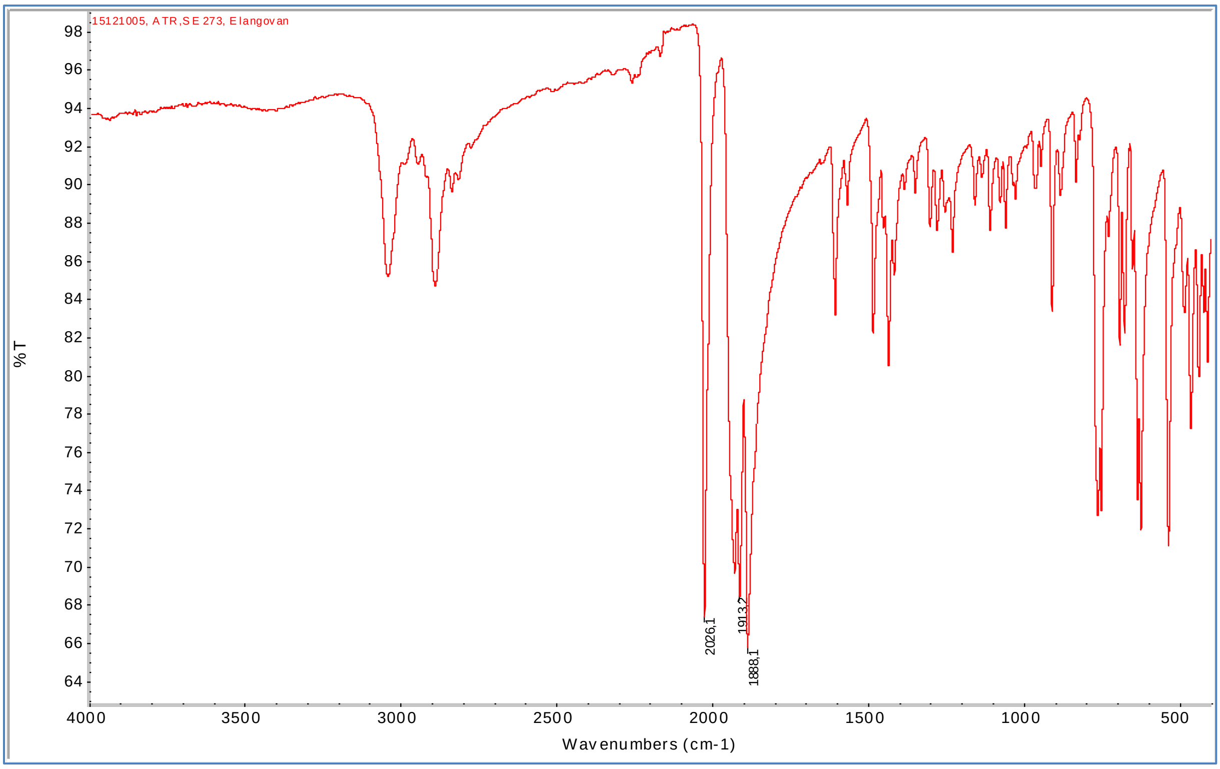

Supplementary Figure 12. IR spectrum of **4**.

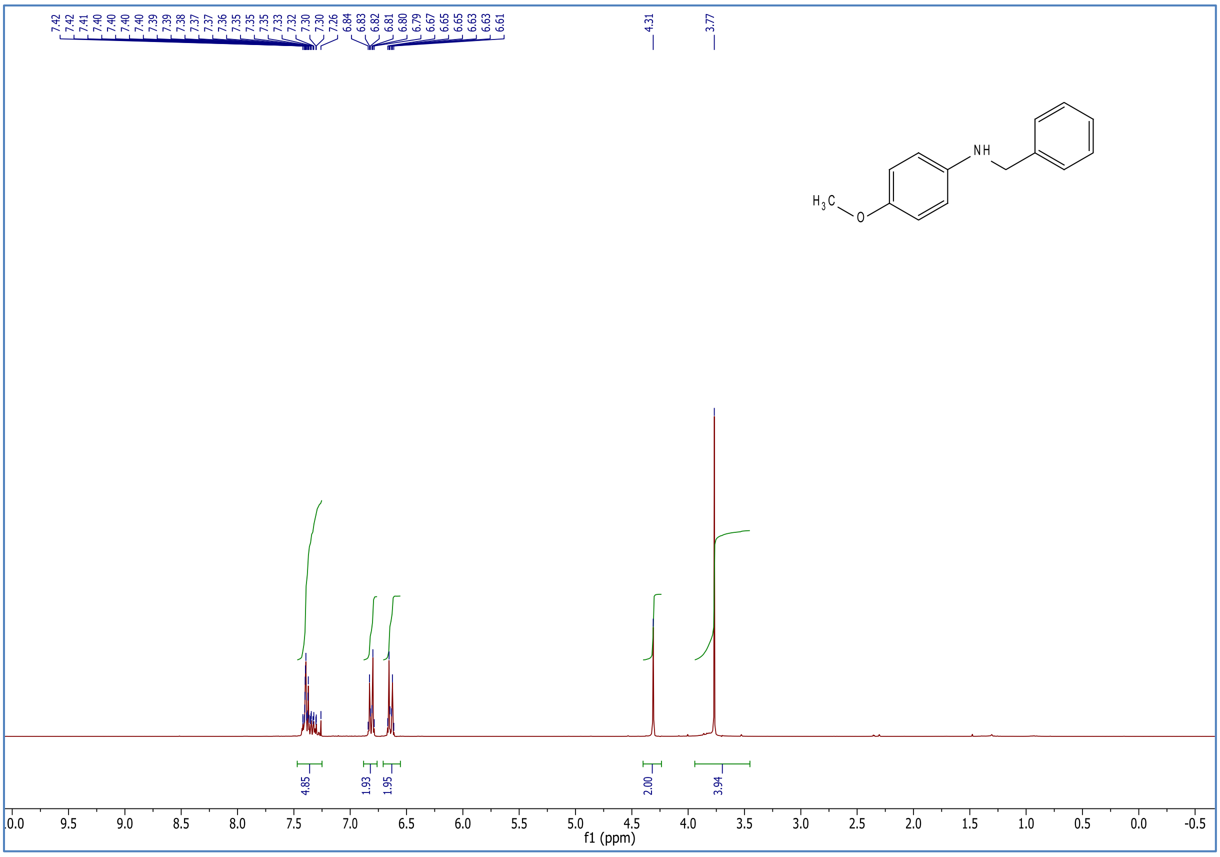

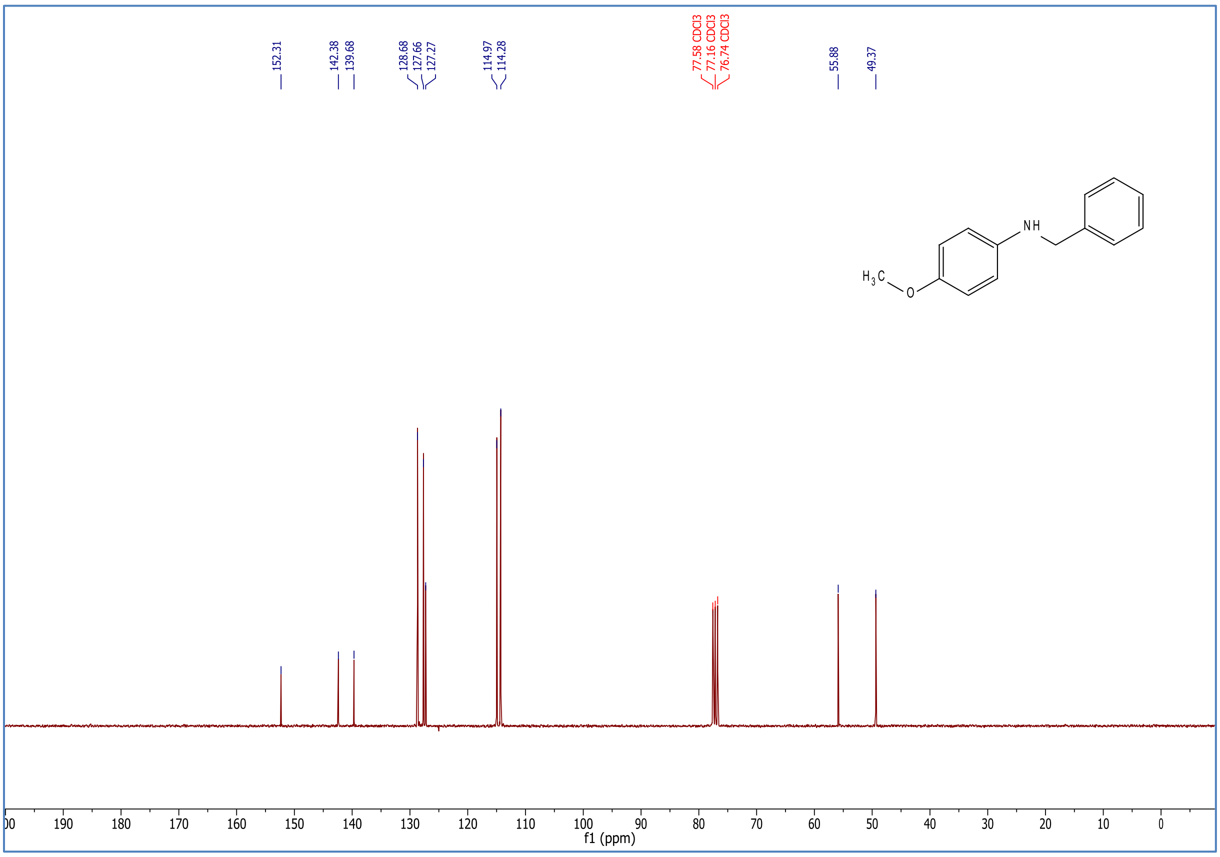

Supplementary Figure 13. ^{1}H and ^{13}C NMR of compound **5b**.

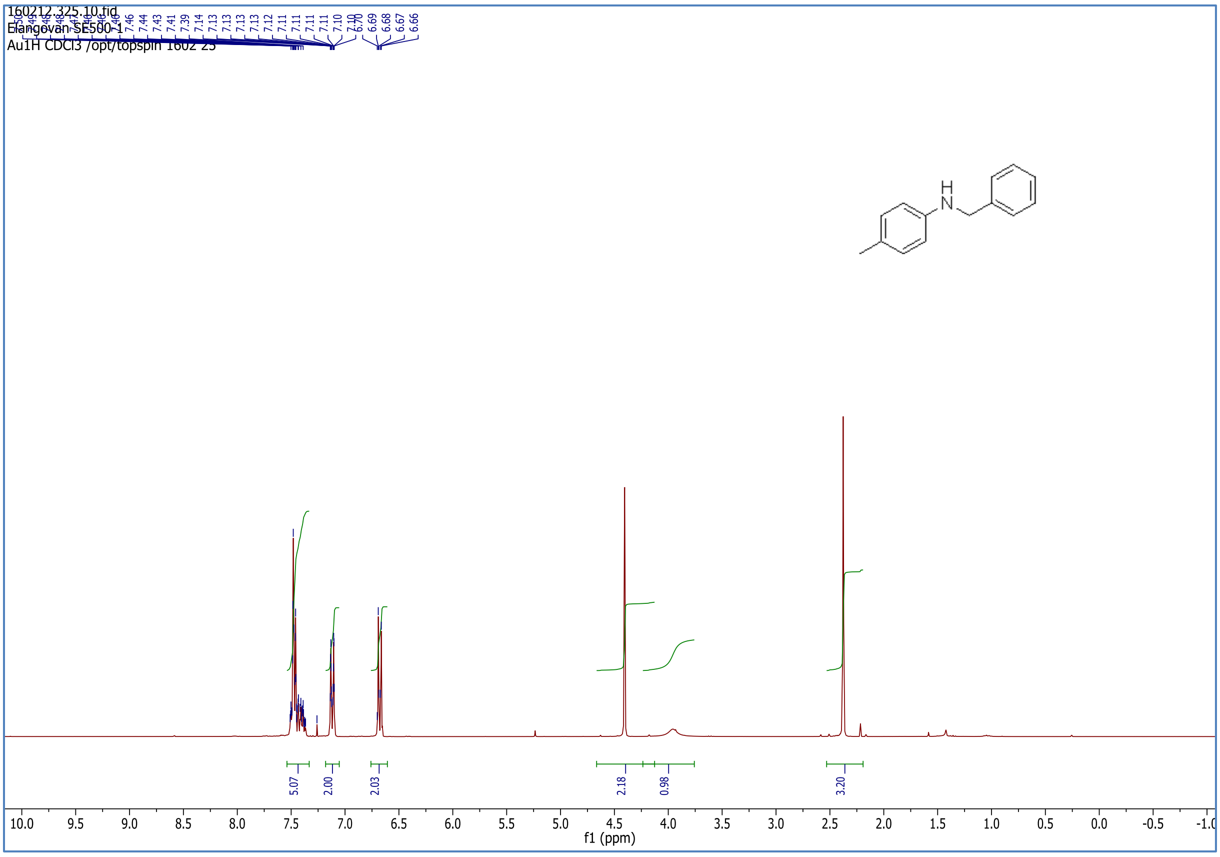

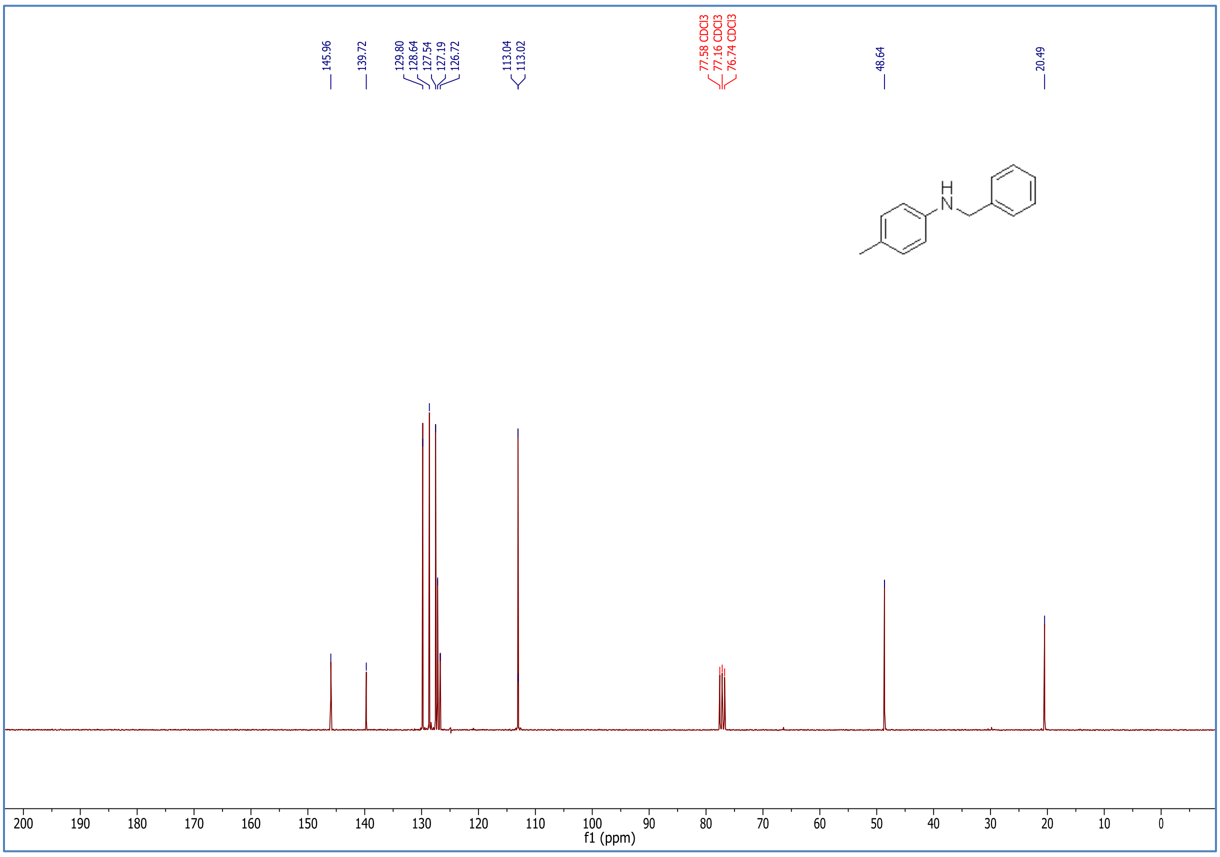

Supplementary Figure 14. ^{1}H and ^{13}C NMR of compound **5c**.

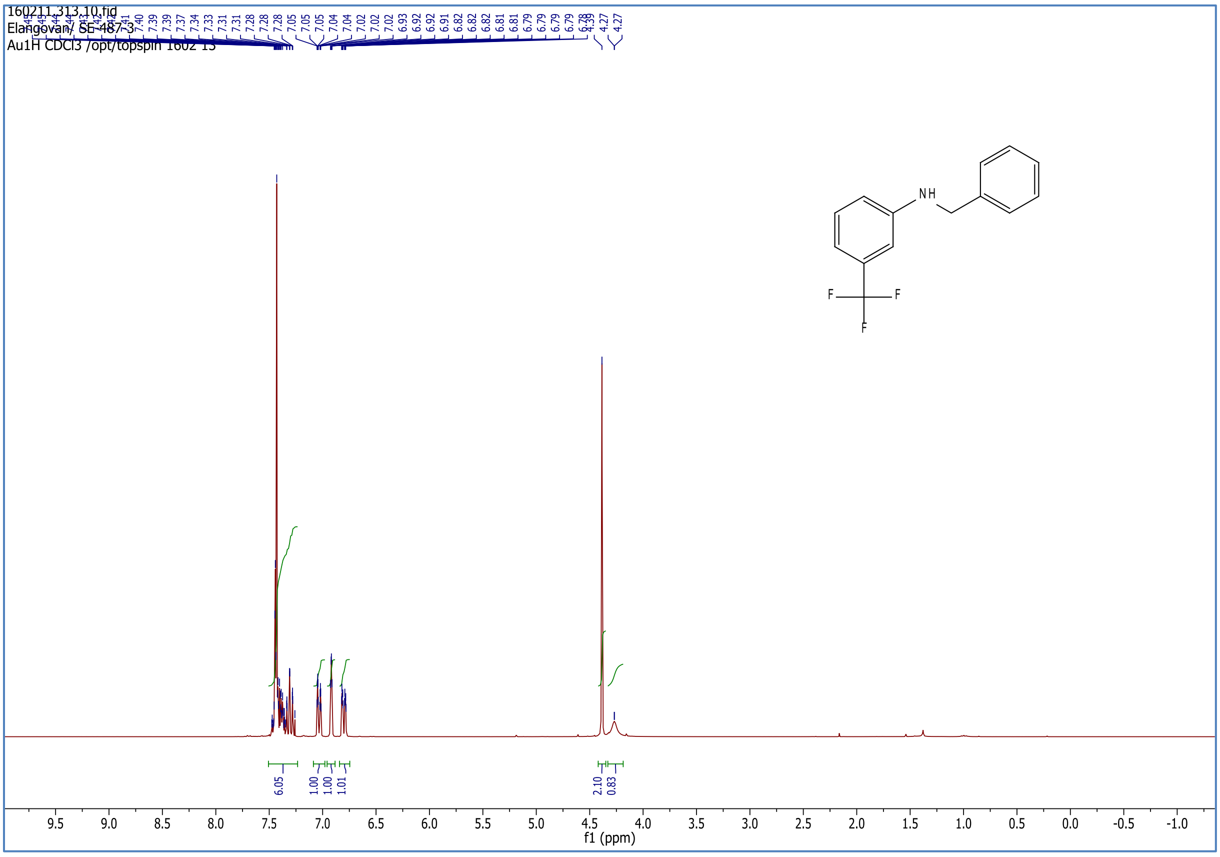

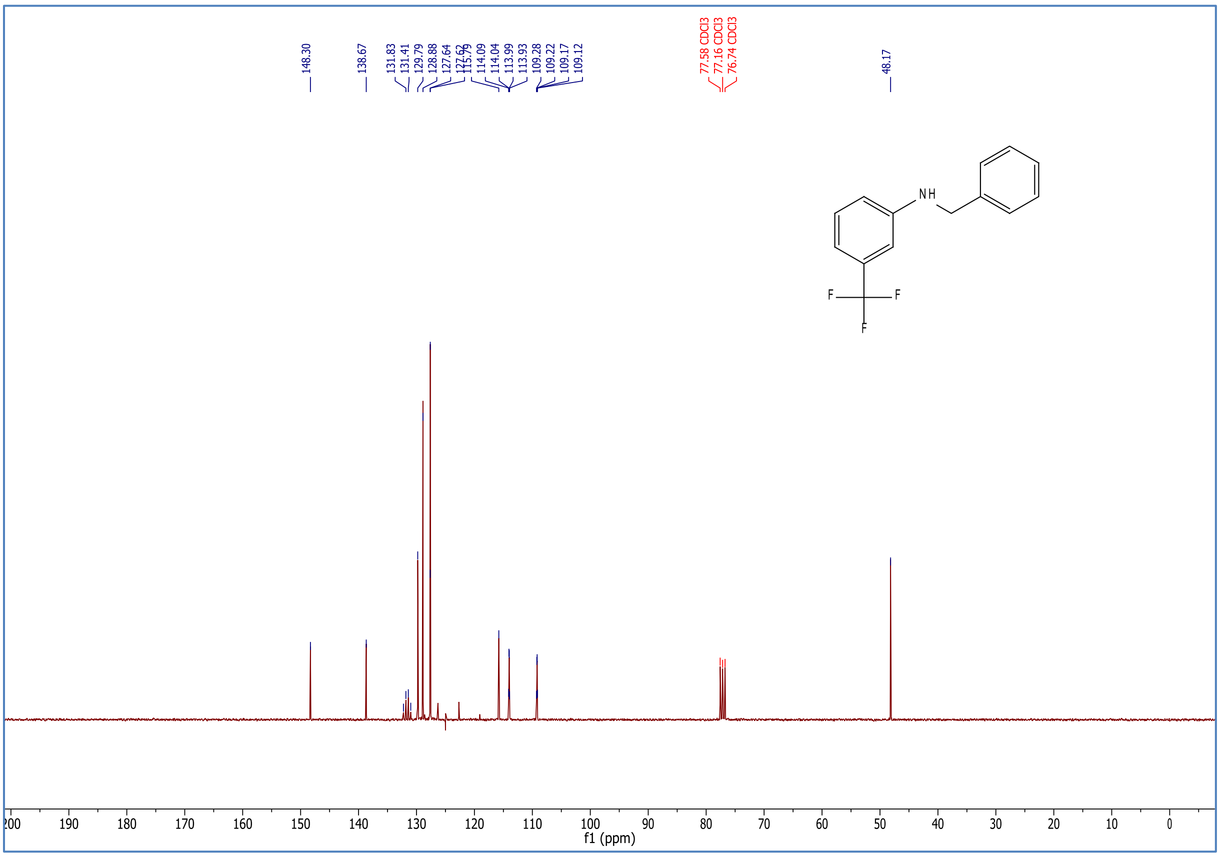

Supplementary Figure 15. ^{1}H and ^{13}C NMR of compound **5j**.

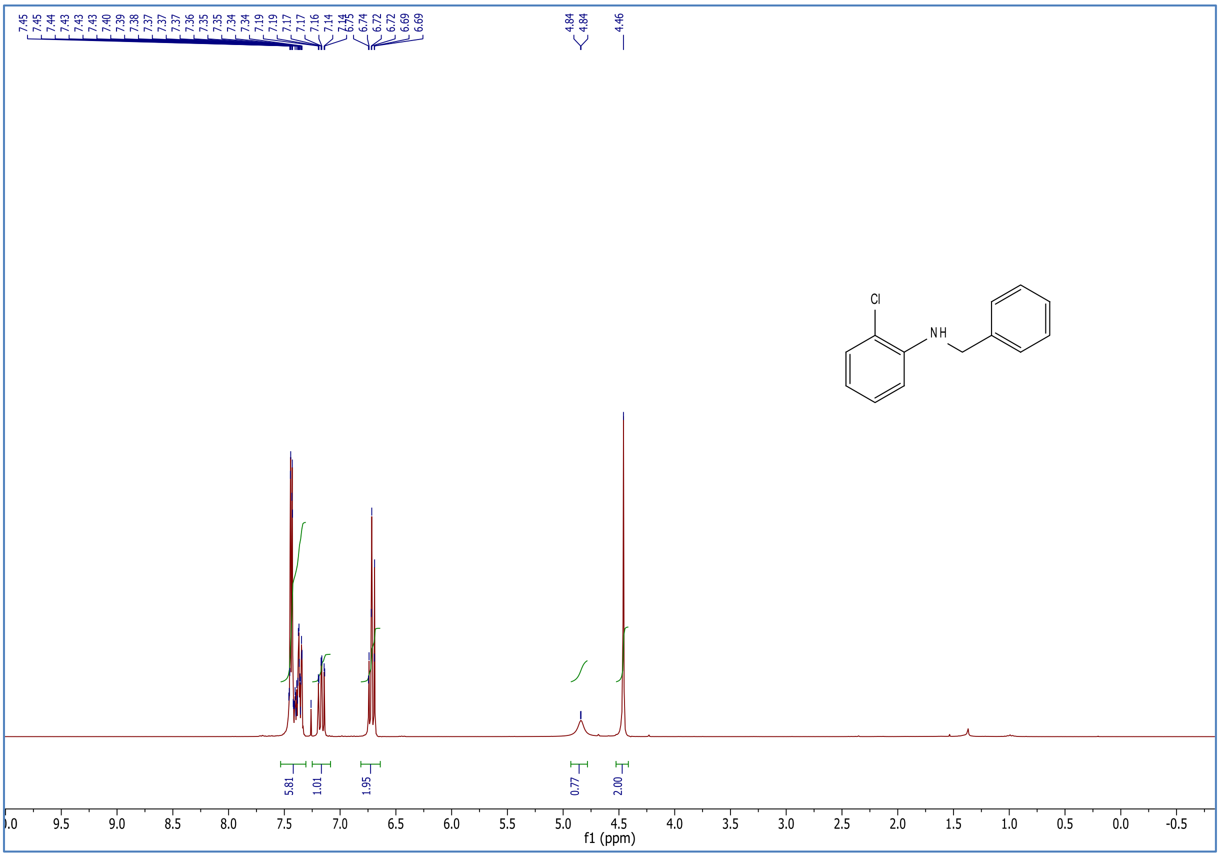

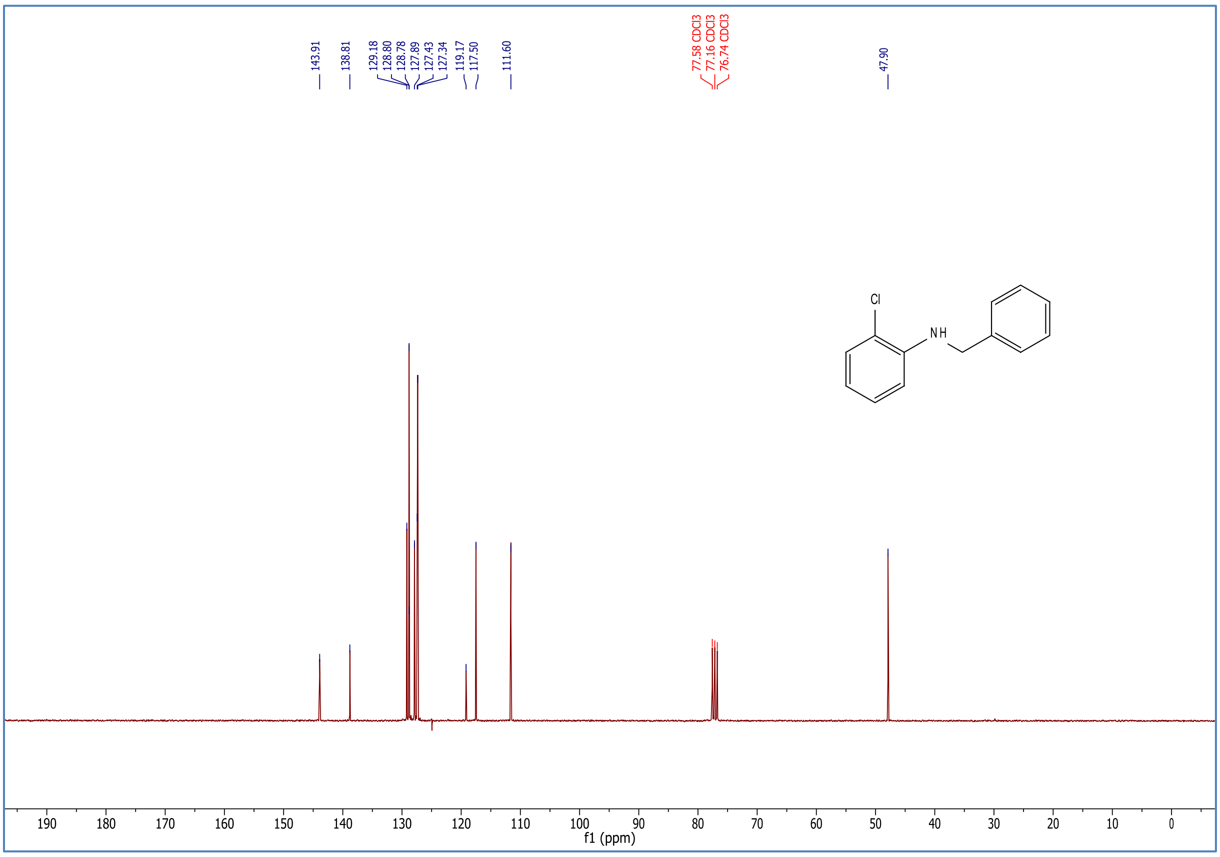

Supplementary Figure 16. ^{1}H and ^{13}C NMR of compound **5i**.

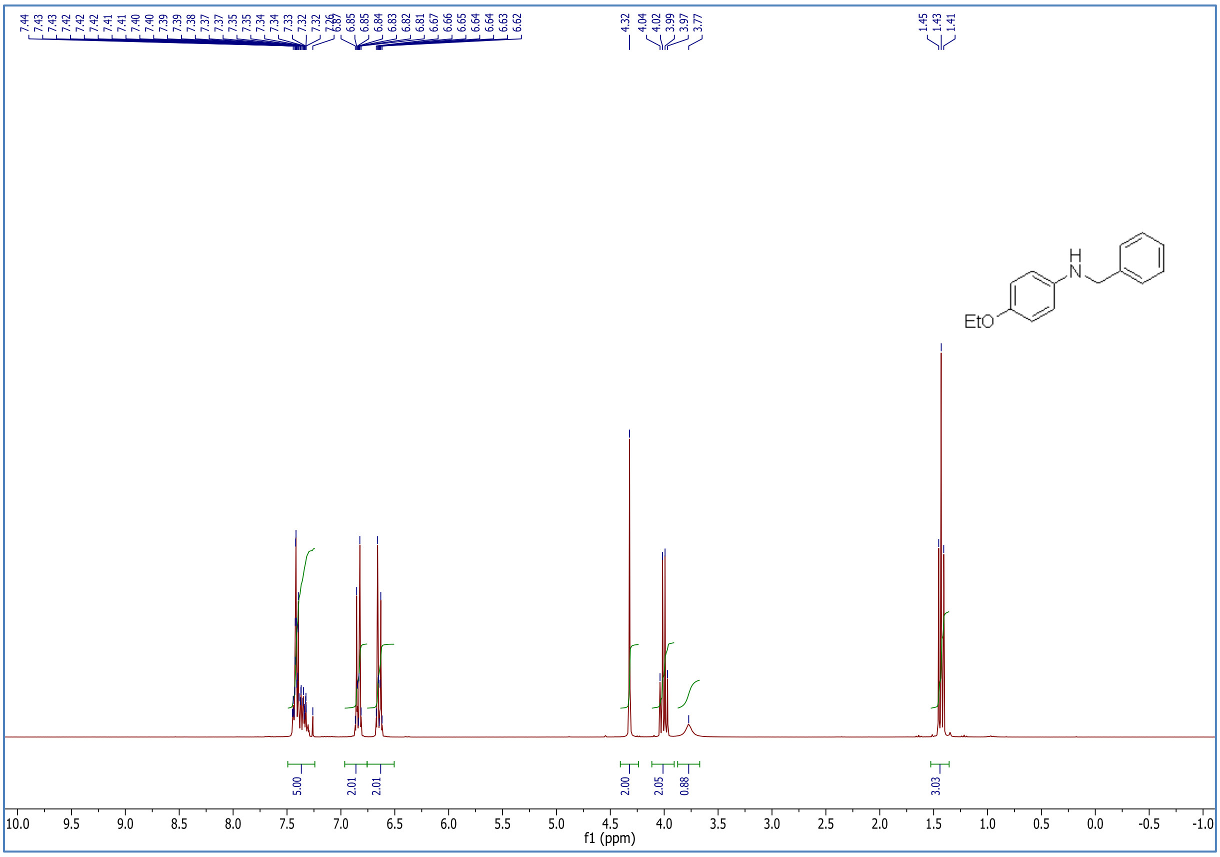

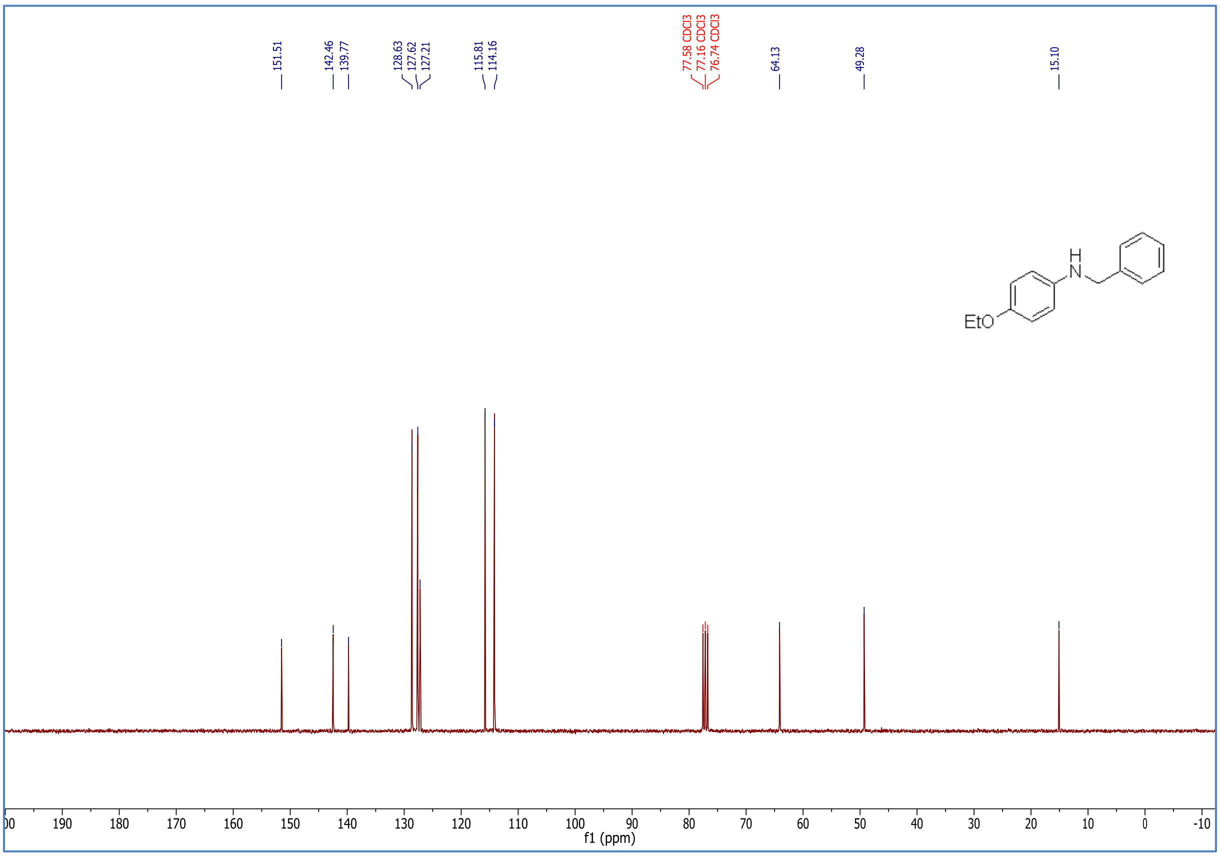

Supplementary Figure 17. ^{1}H and ^{13}C NMR of compound **5d**.

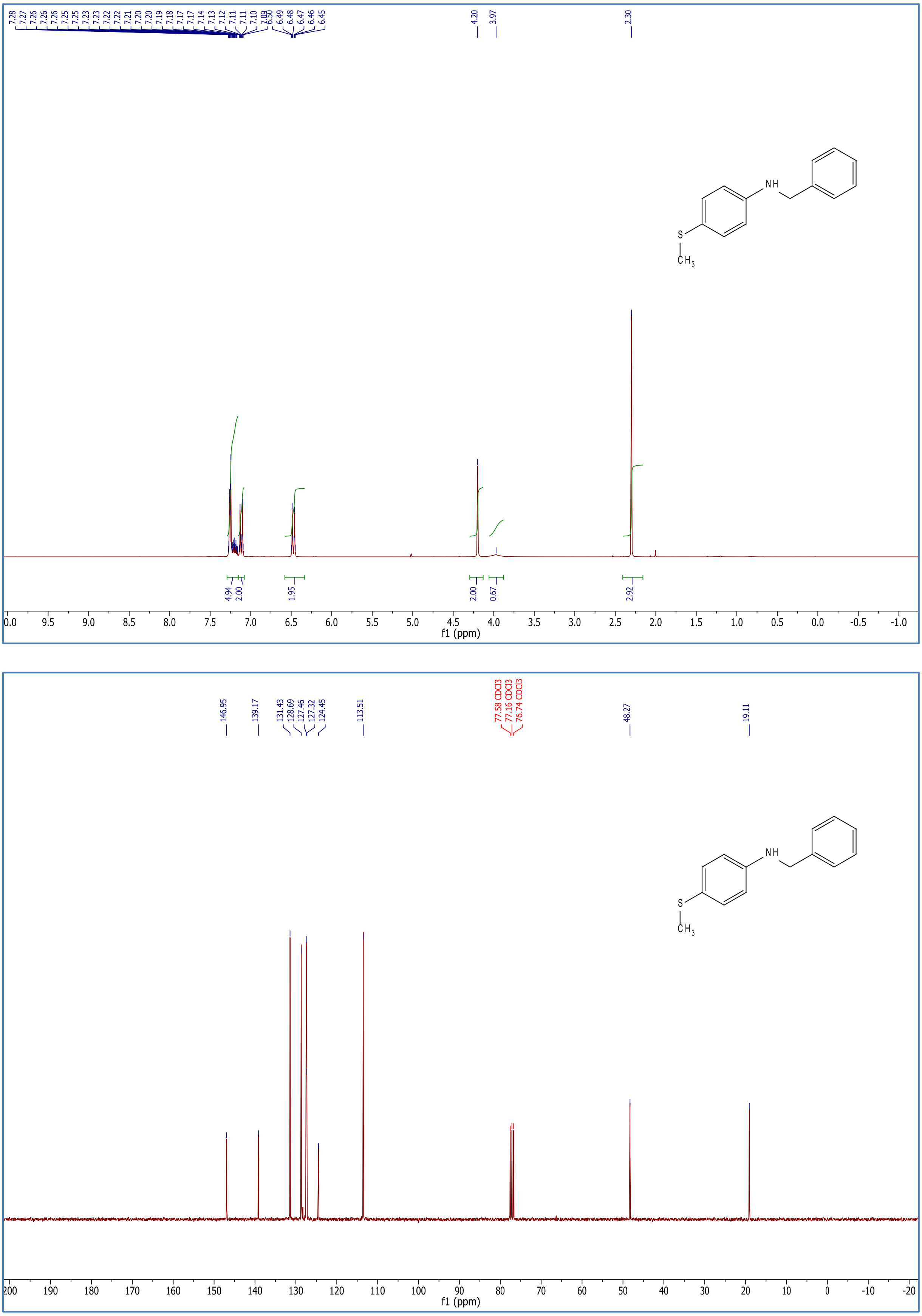

Supplementary Figure 18. ^{1}H and ^{13}C NMR of compound **5e**.

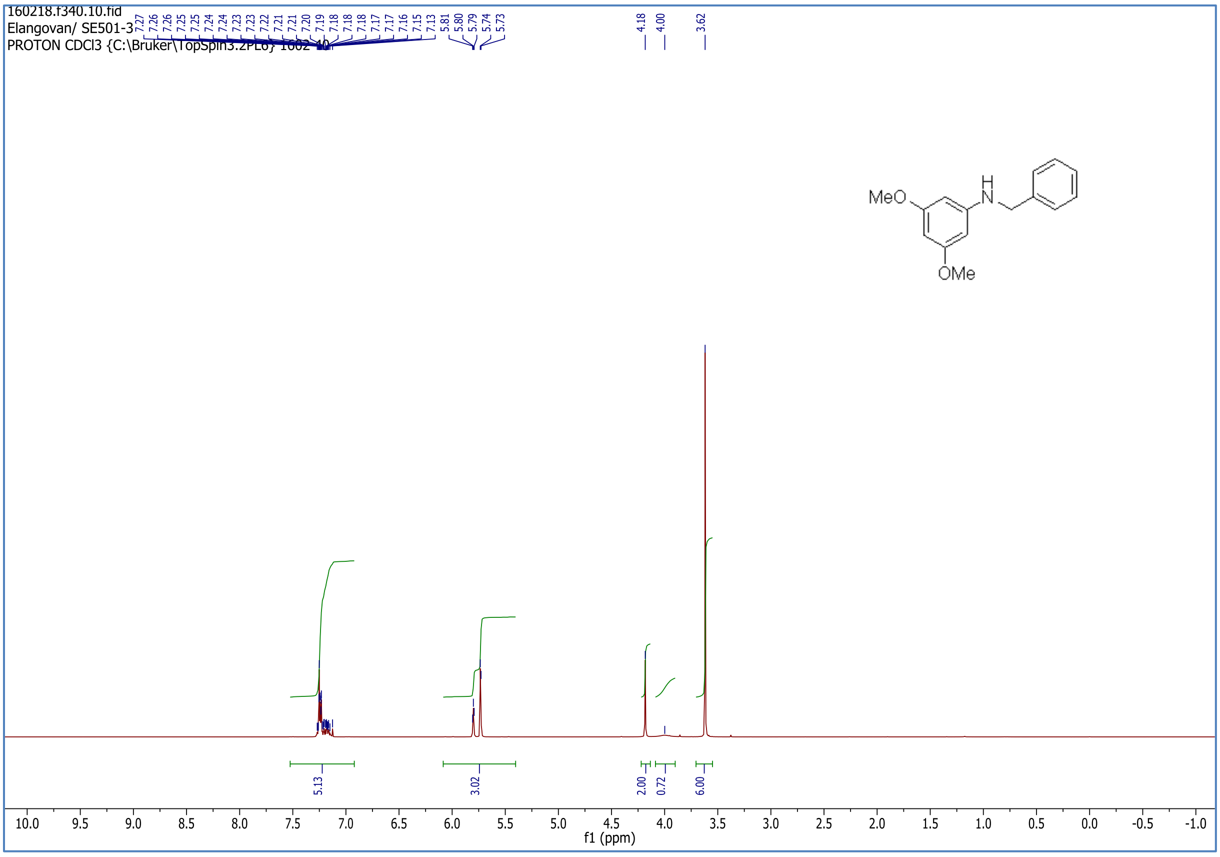

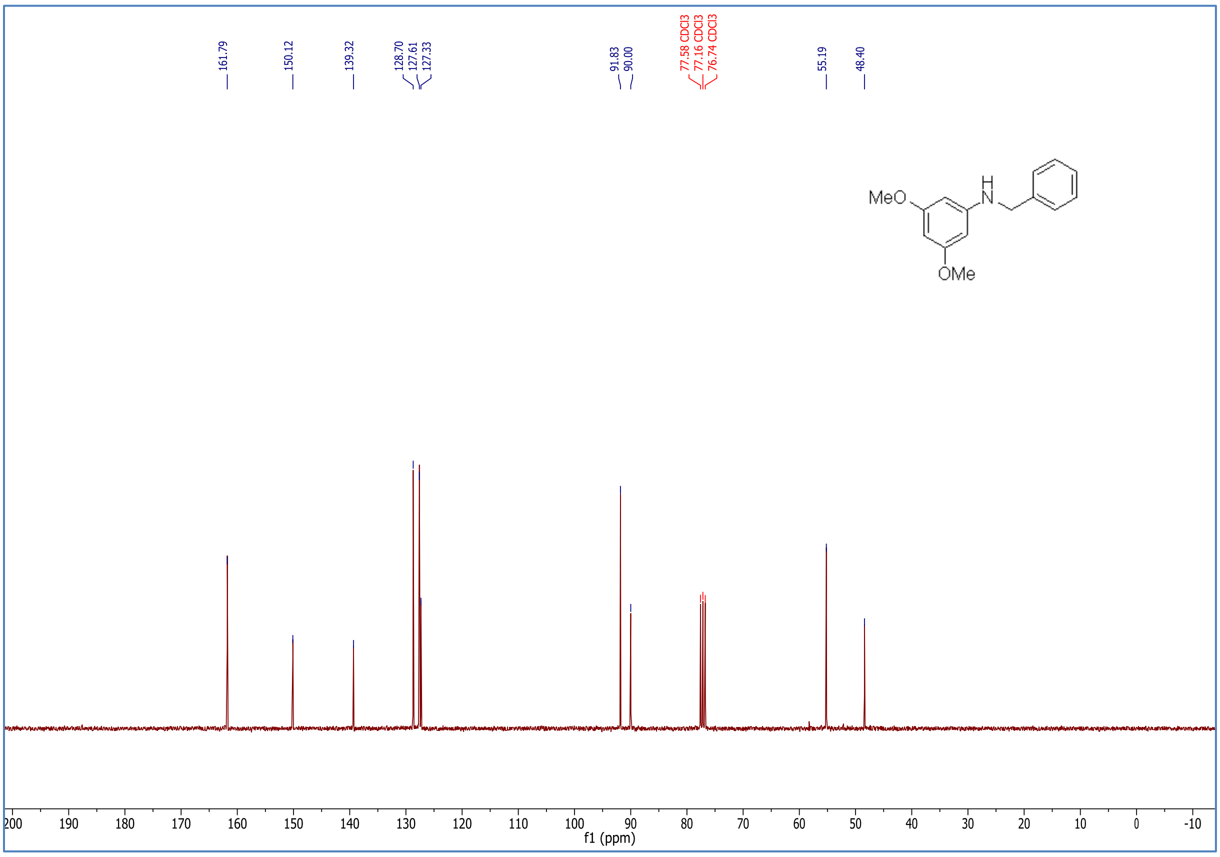

Supplementary Figure 19. ^{1}H and ^{13}C NMR of compound **5f**.

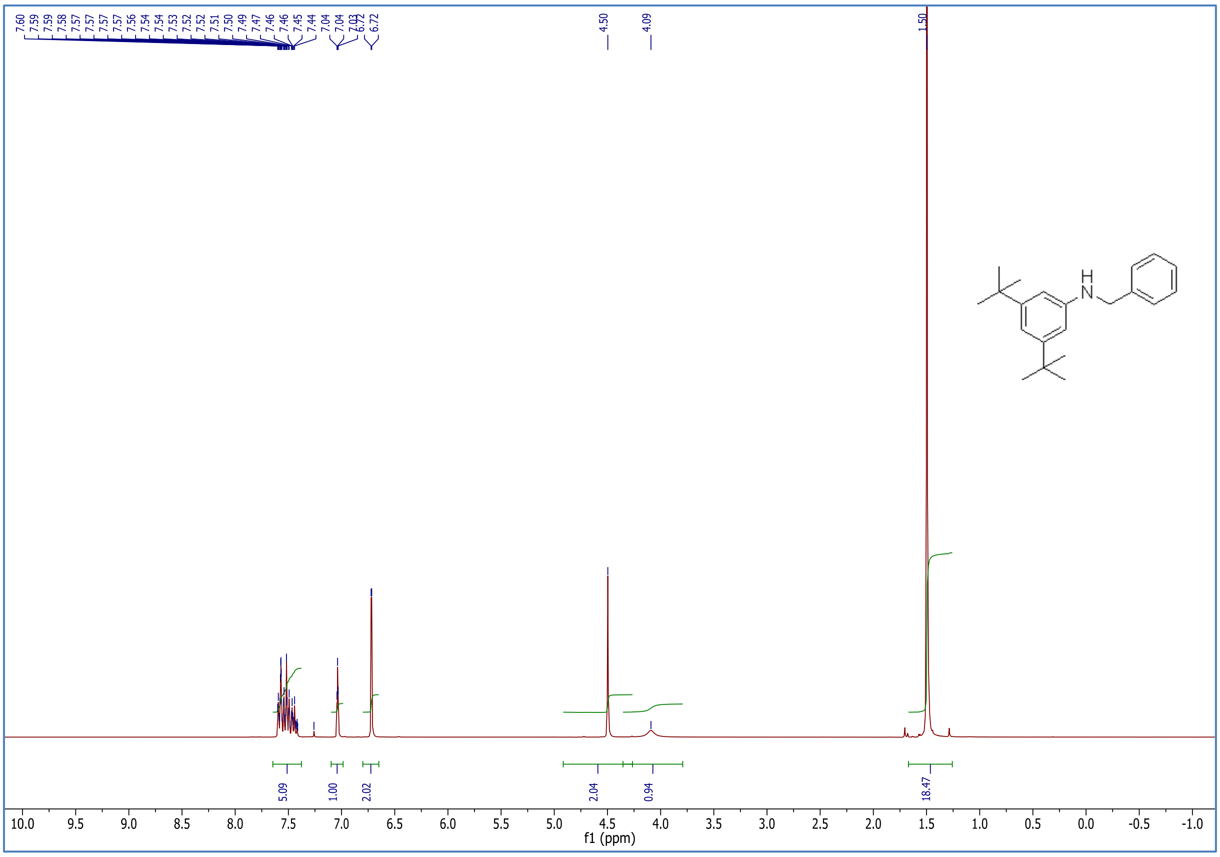

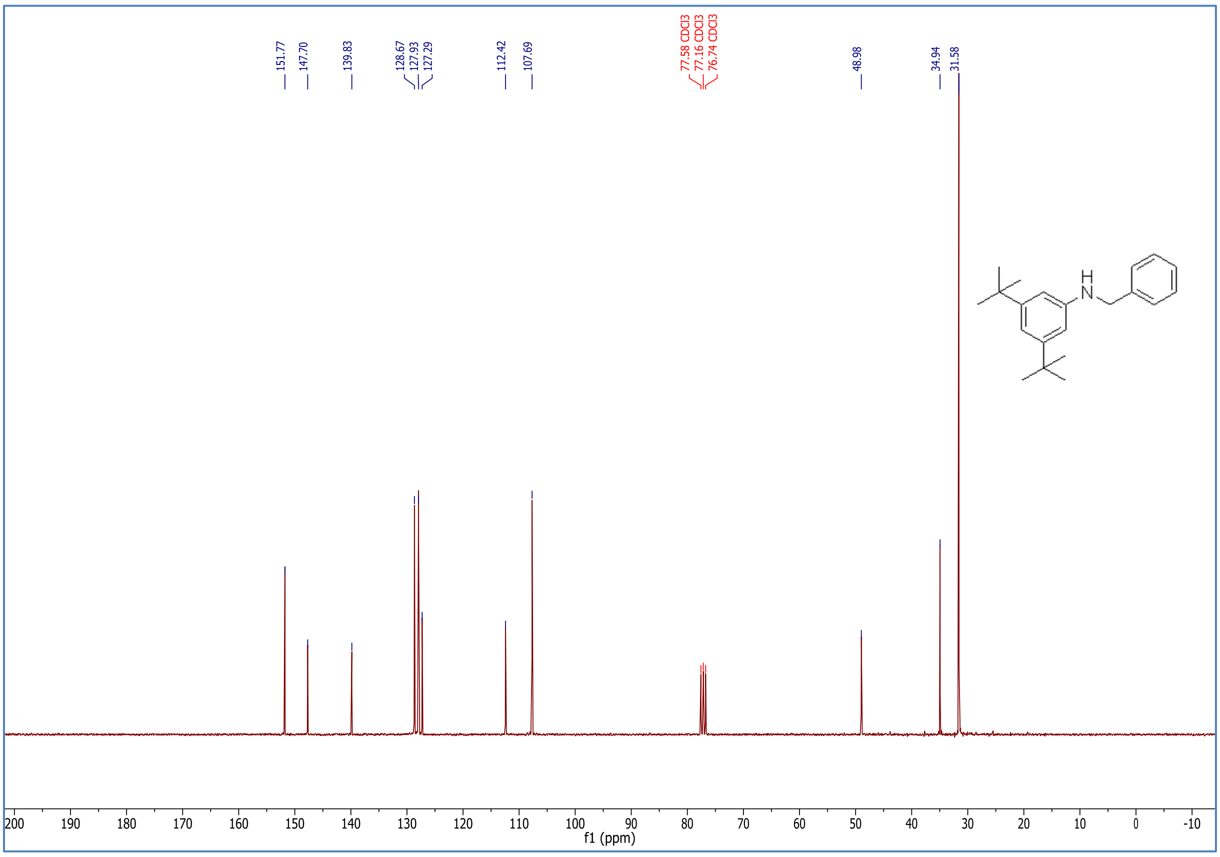

Supplementary Figure 20. ^{1}H and ^{13}C NMR of compound **5g**.

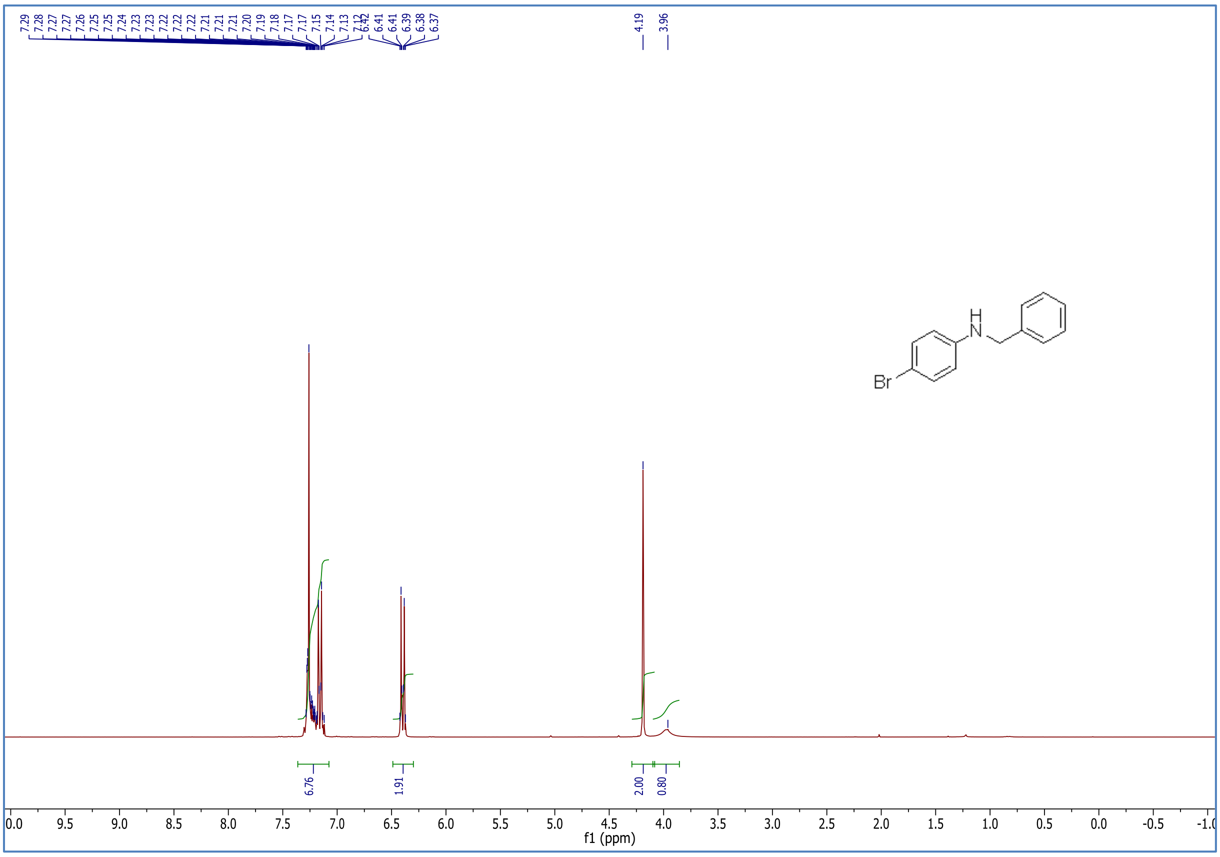

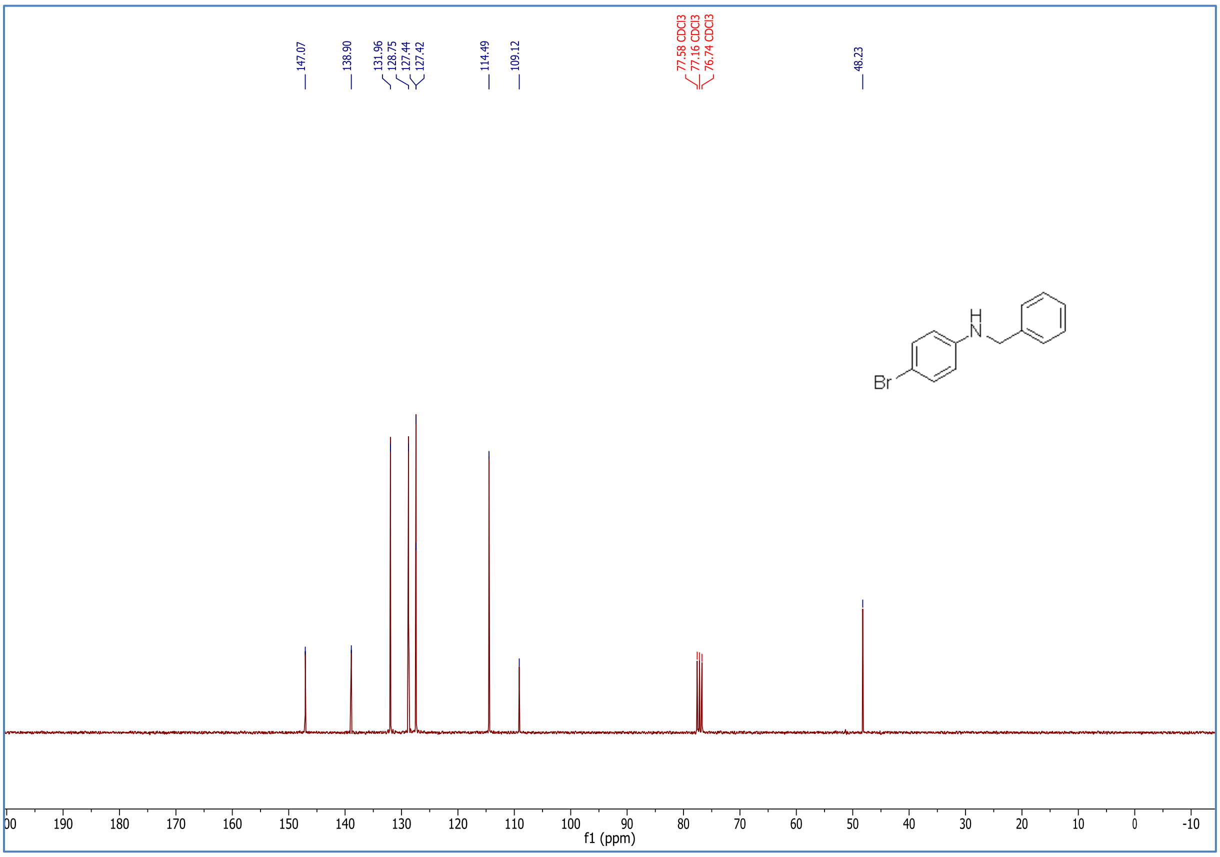

Supplementary Figure 21. ^{1}H and ^{13}C NMR of compound **5h**.

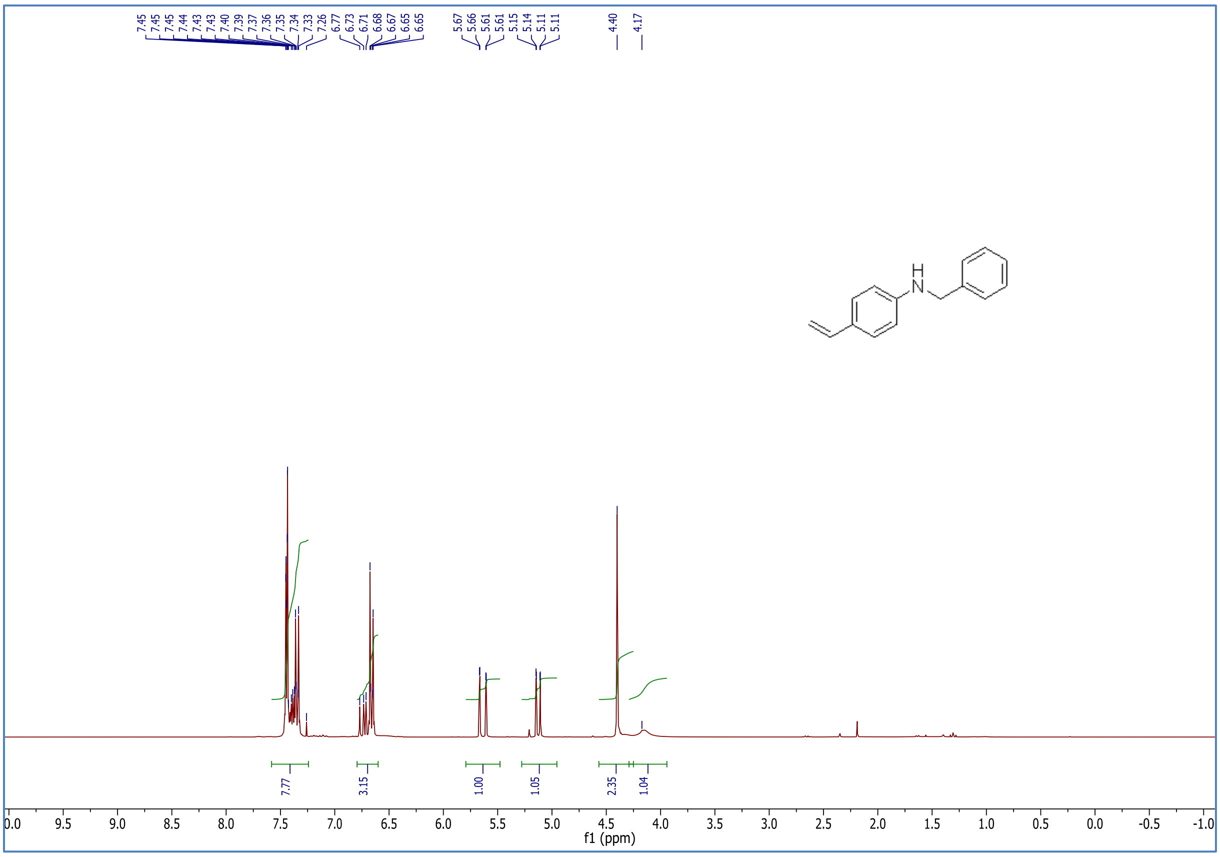

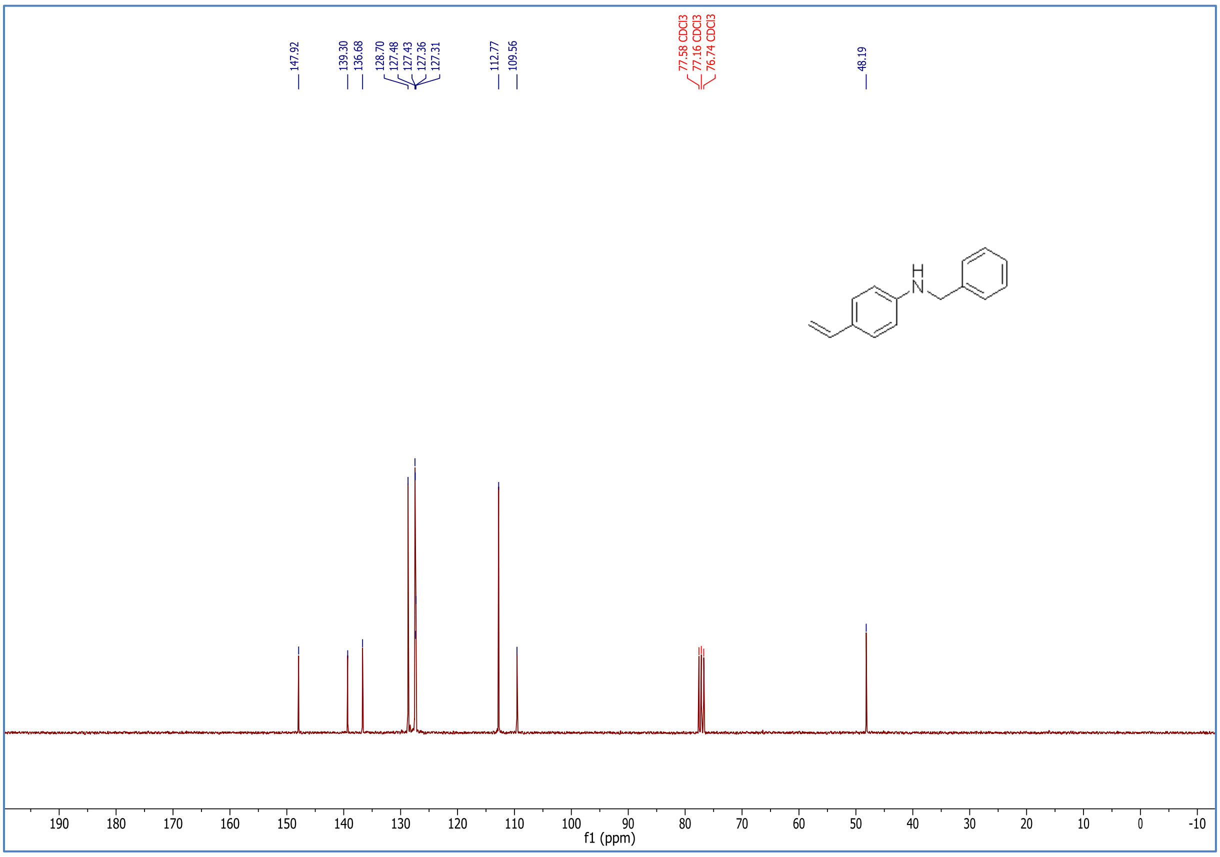

Supplementary Figure 22. ^{1}H and ^{13}C NMR of compound **5k**.

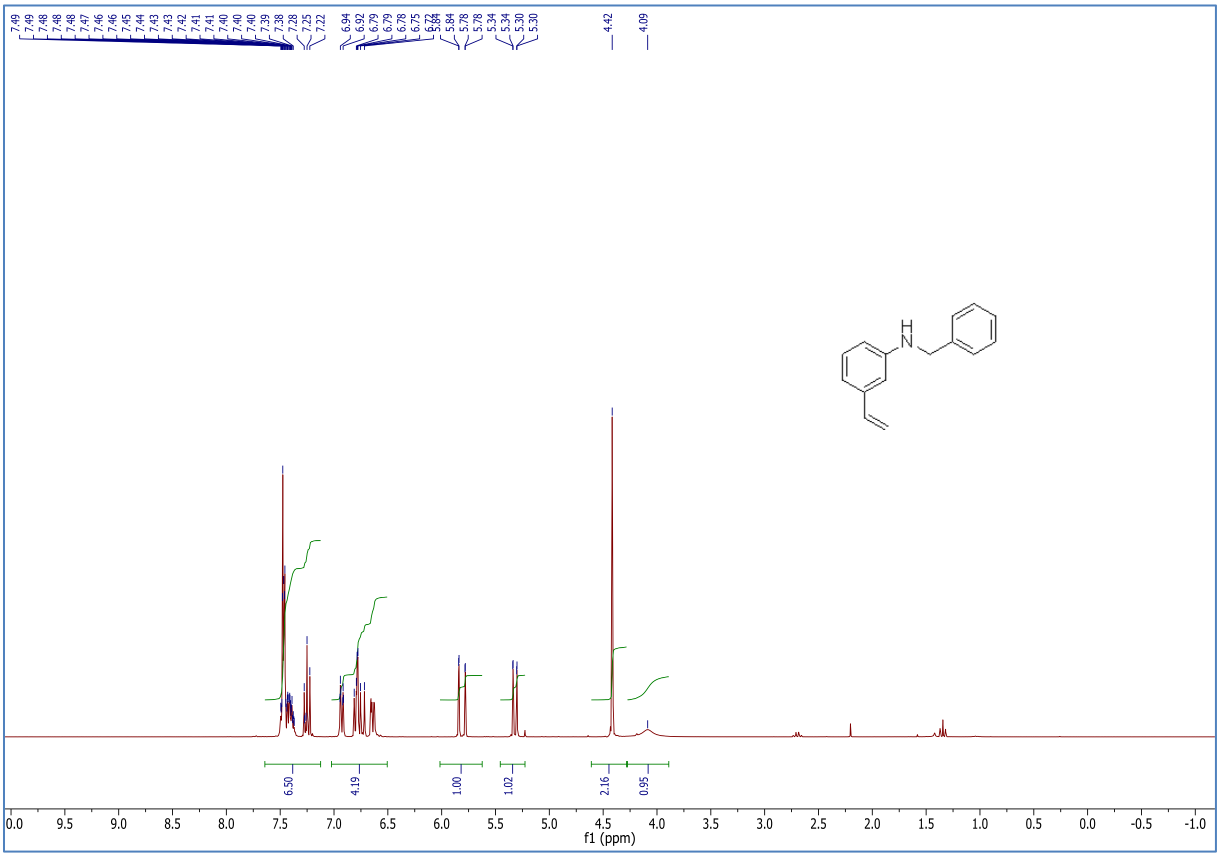

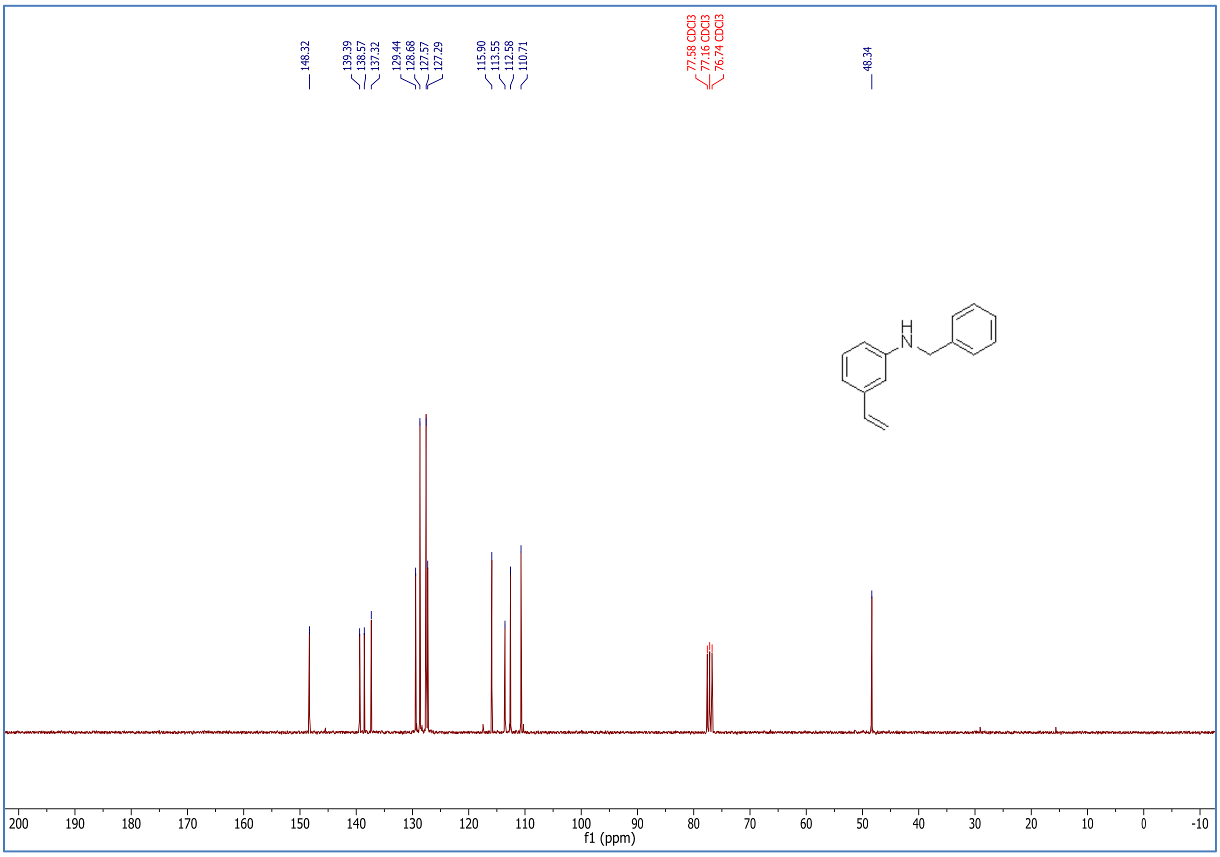

Supplementary Figure 23. ^{1}H and ^{13}C NMR of compound **5l**.

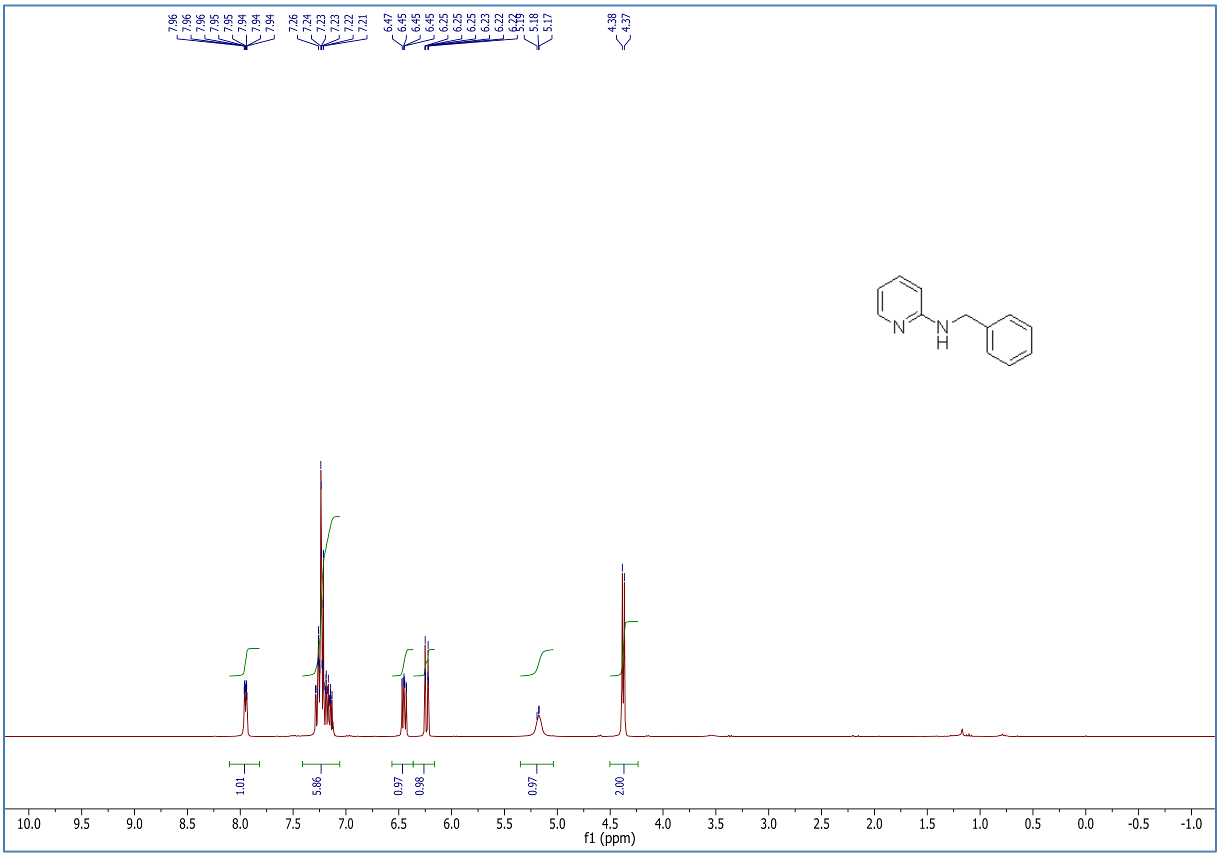

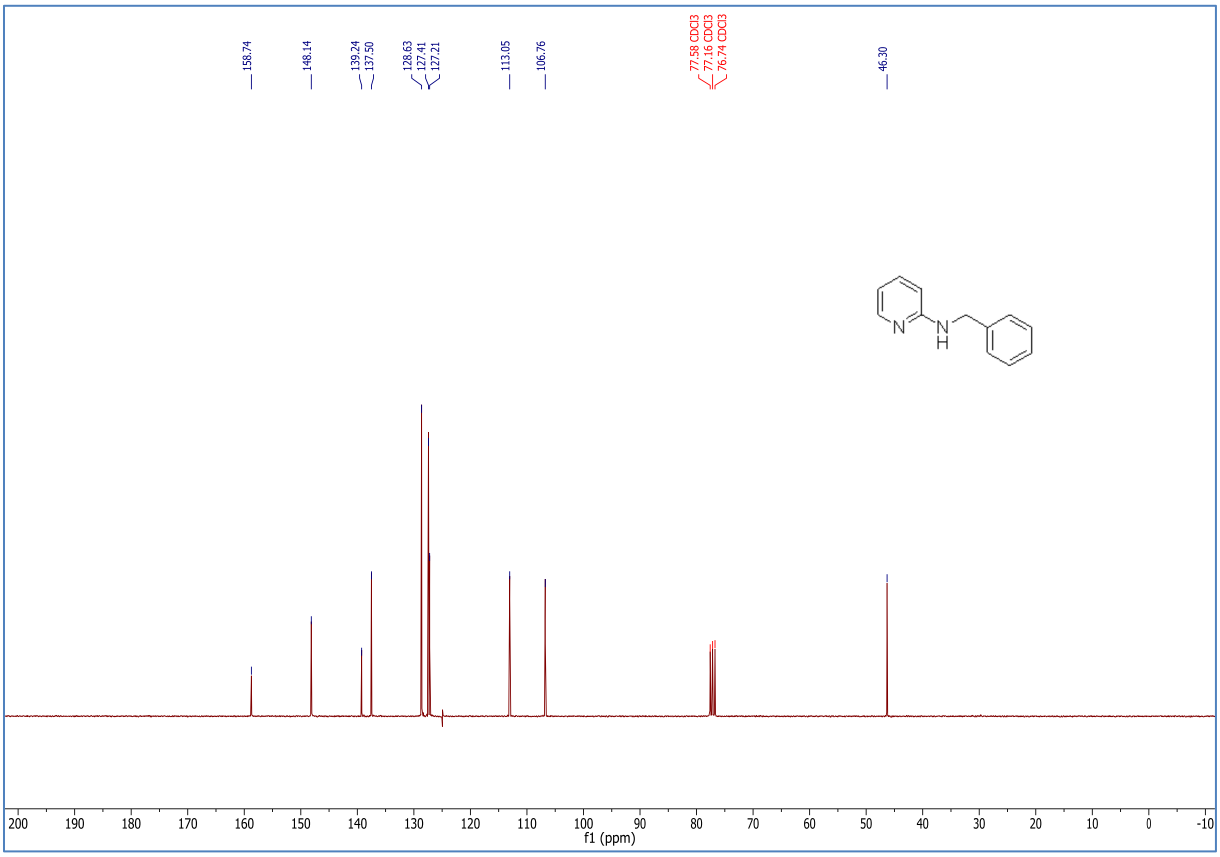

Supplementary Figure 24. ^{1}H and ^{13}C NMR of compound **5m**.

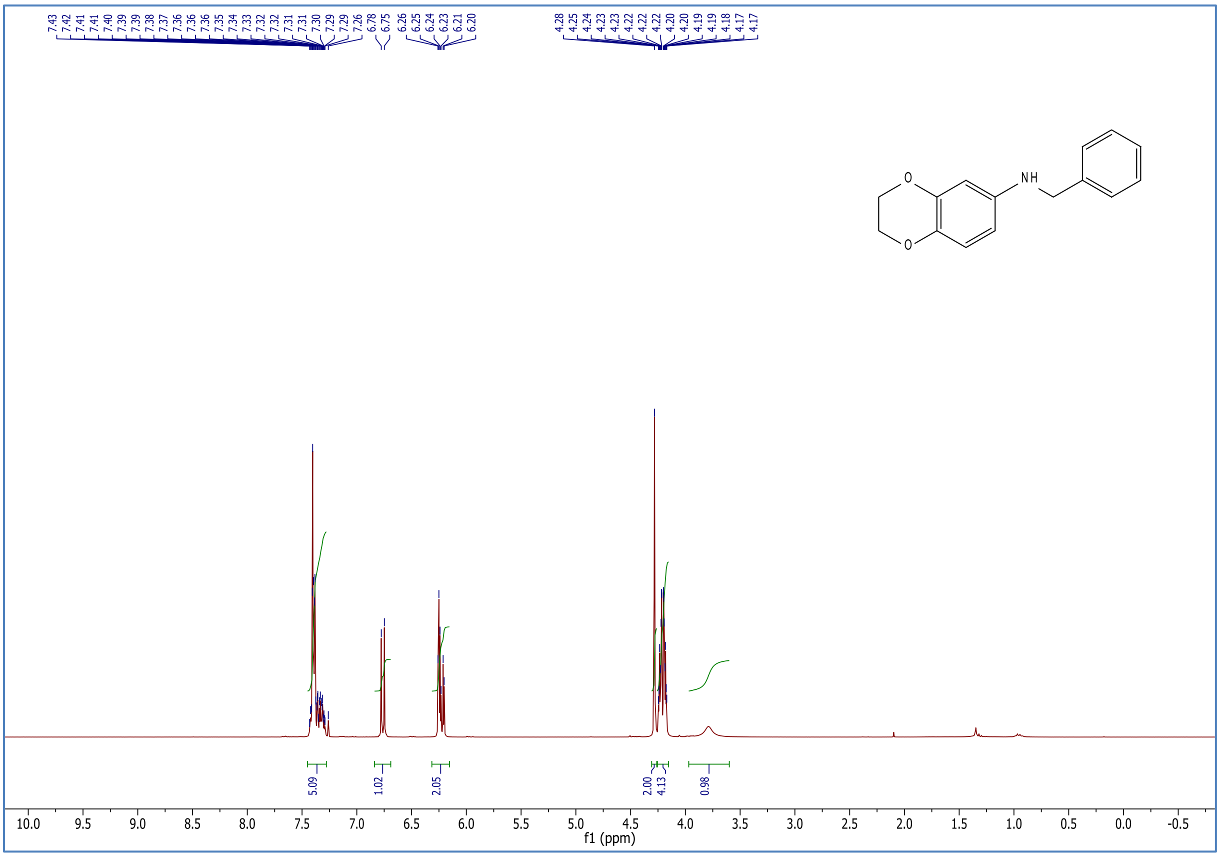

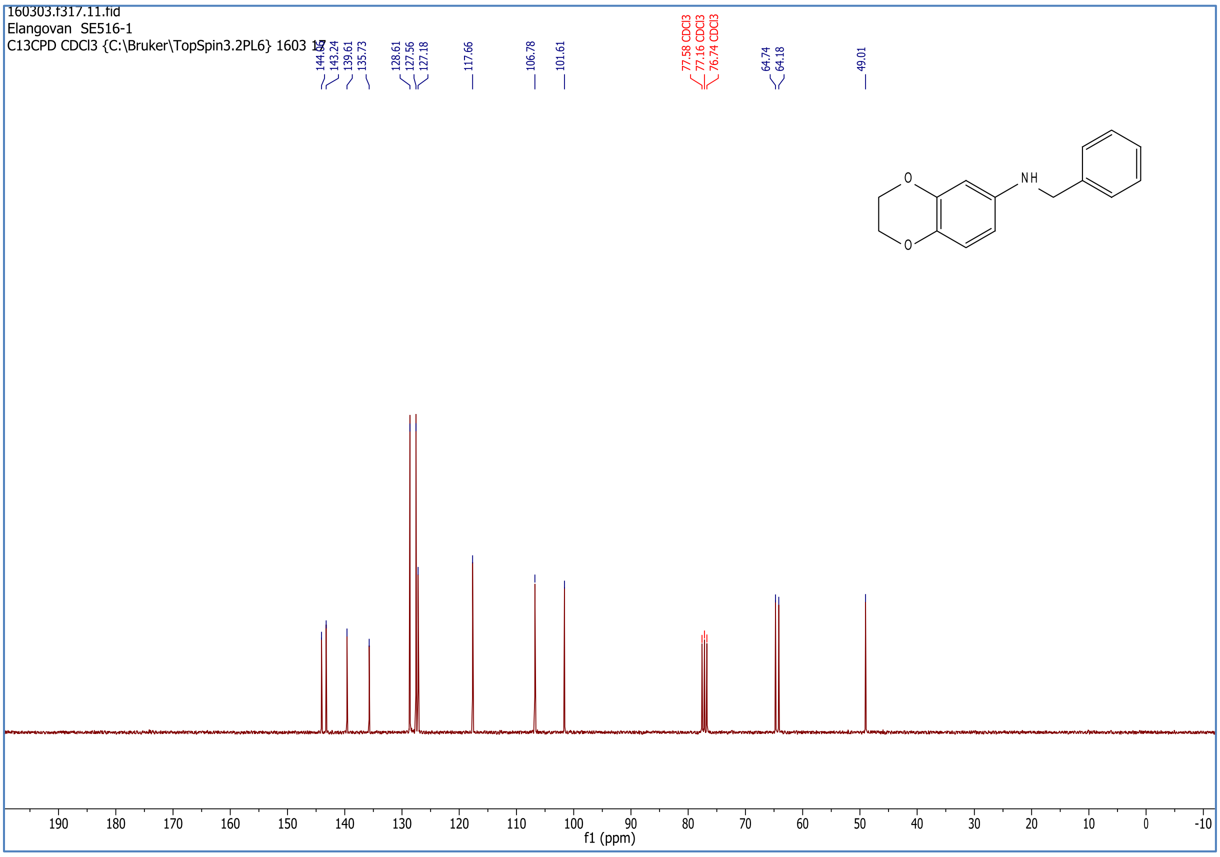

Supplementary Figure 25. ^{1}H and ^{13}C NMR of compound **5o**.

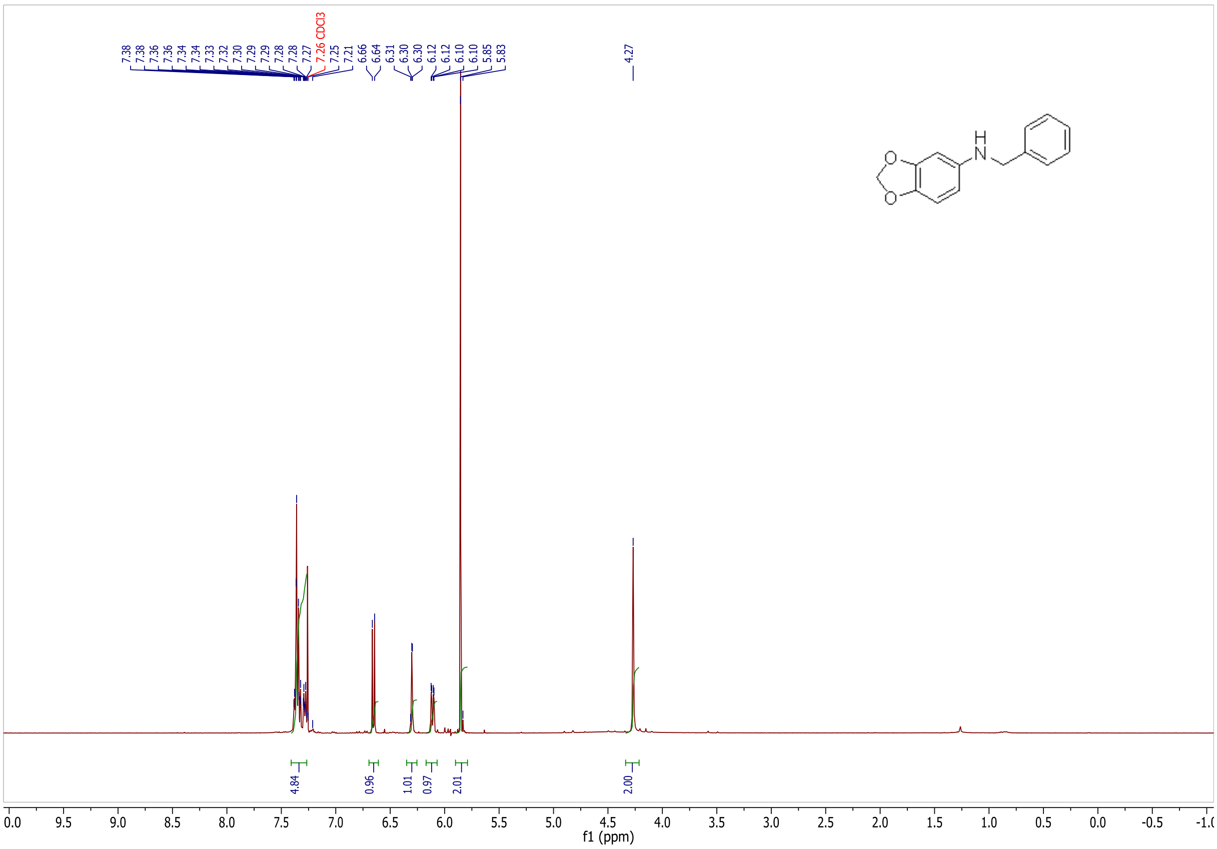

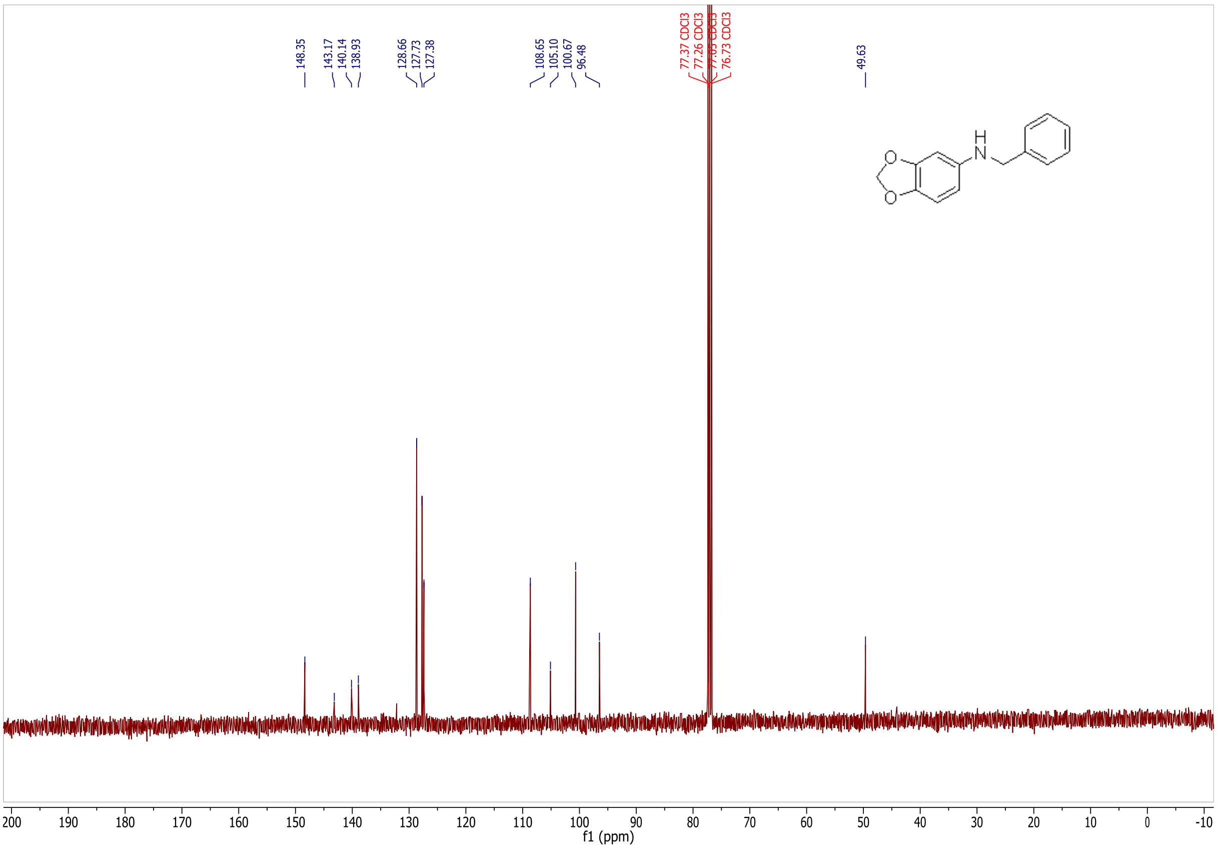

Supplementary Figure 26. ^{1}H and ^{13}C NMR of compound **5p**.

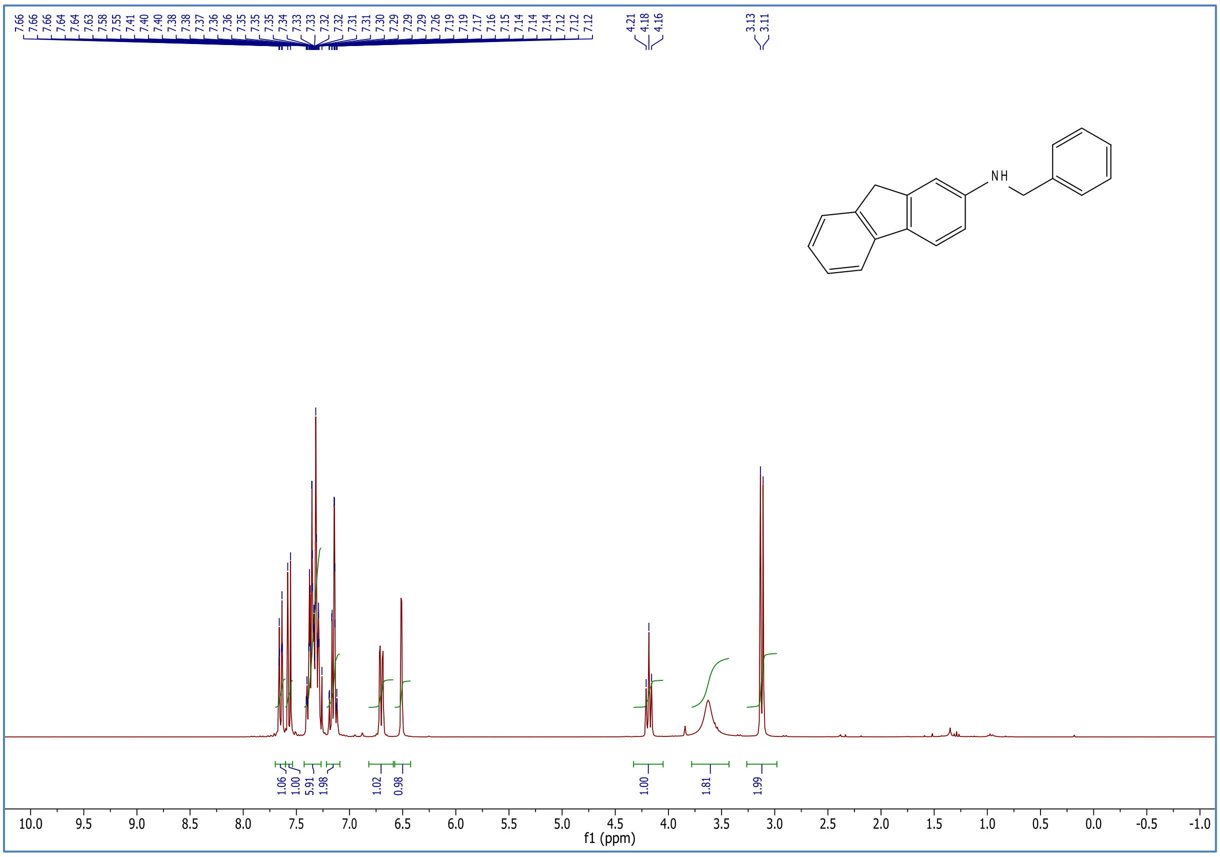

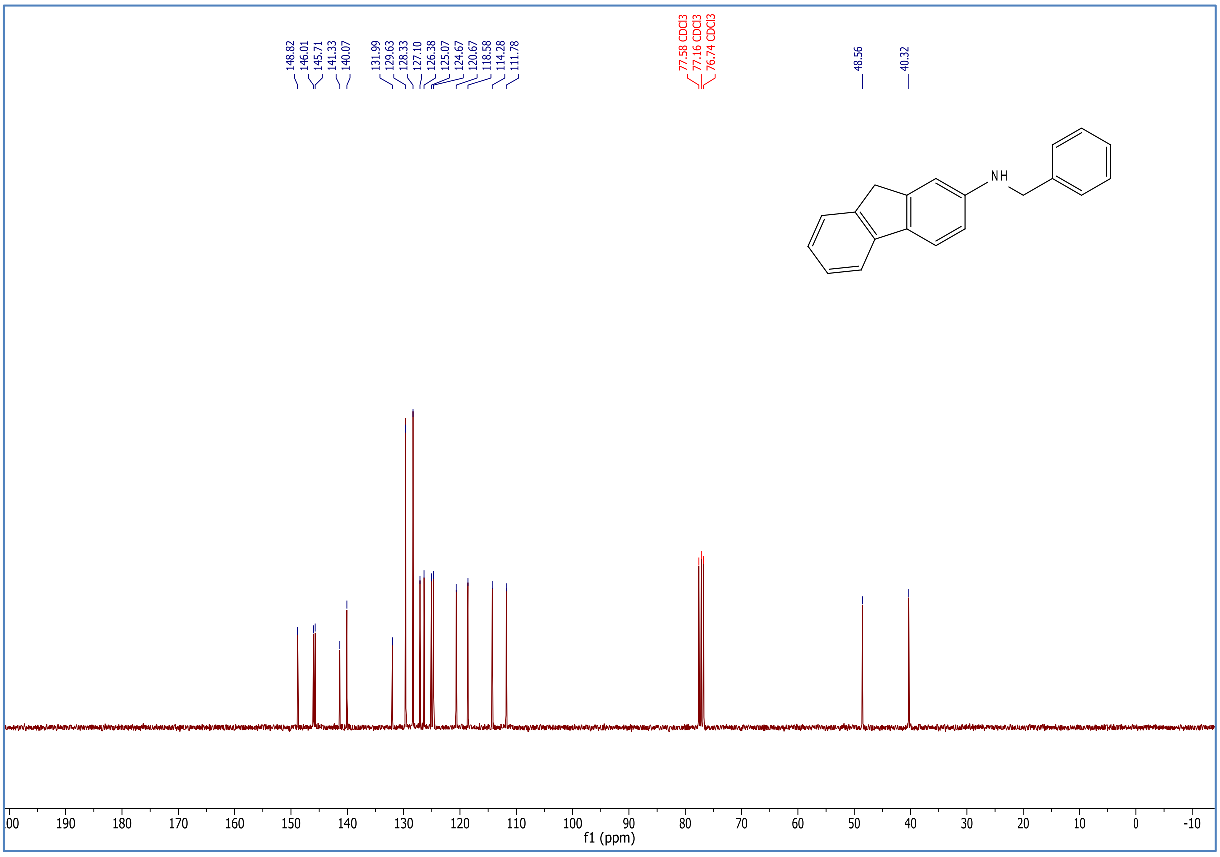

Supplementary Figure 27. ^{1}H and ^{13}C NMR of compound **5q**.

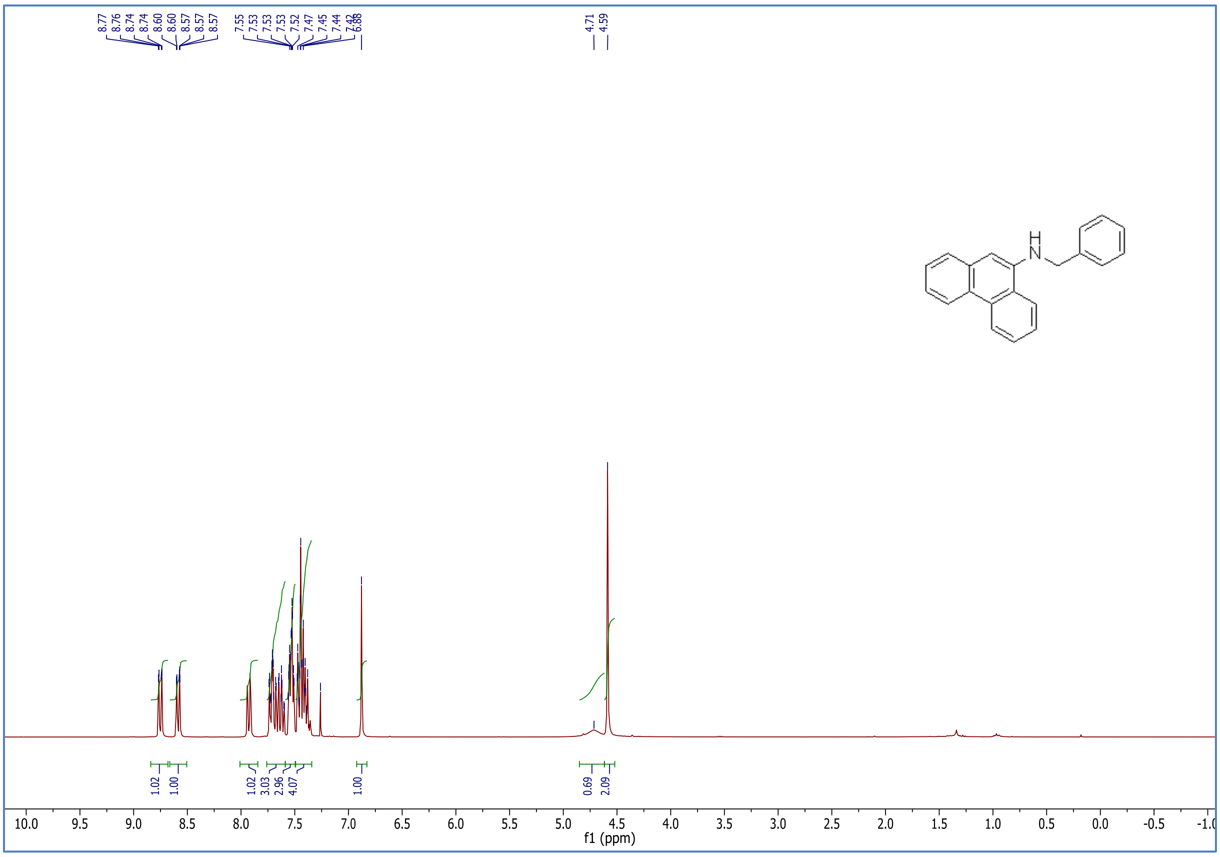

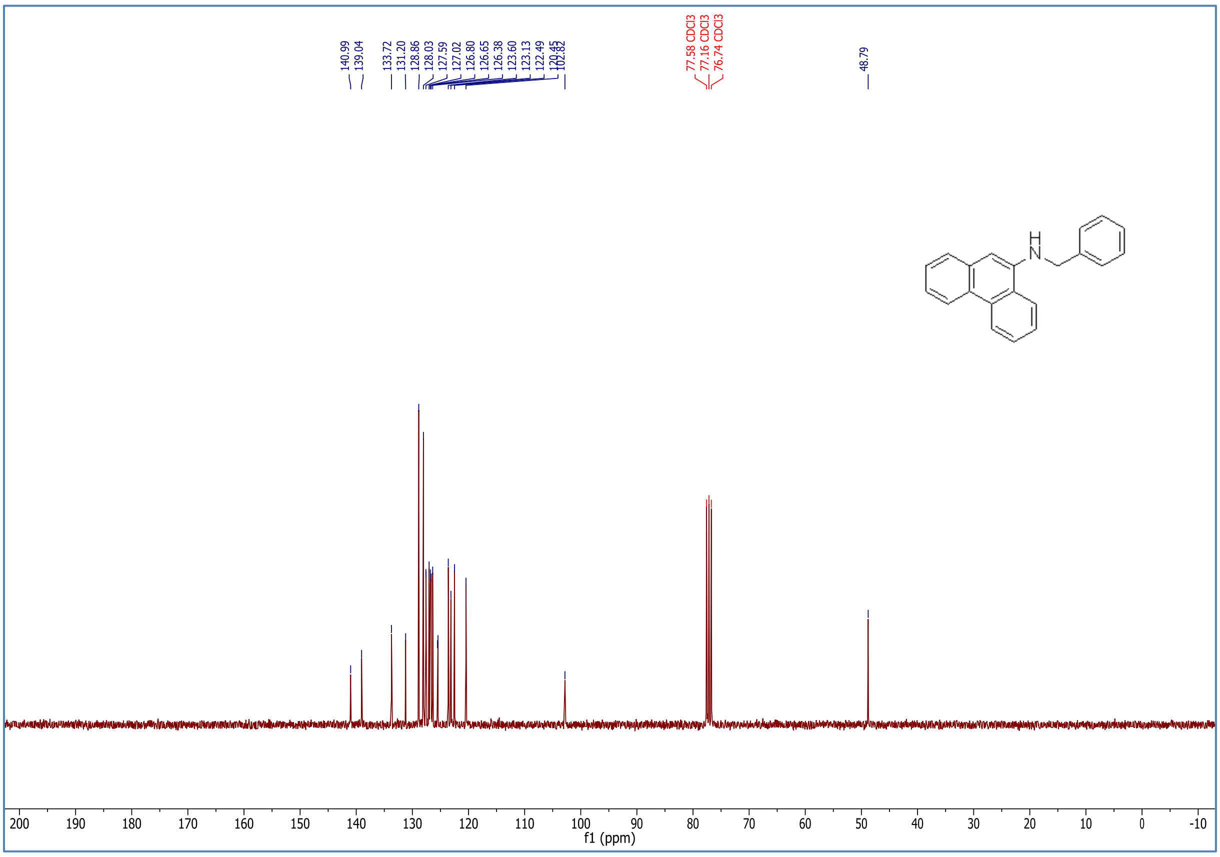

Supplementary Figure 28. ^{1}H and ^{13}C NMR of compound **5r**.

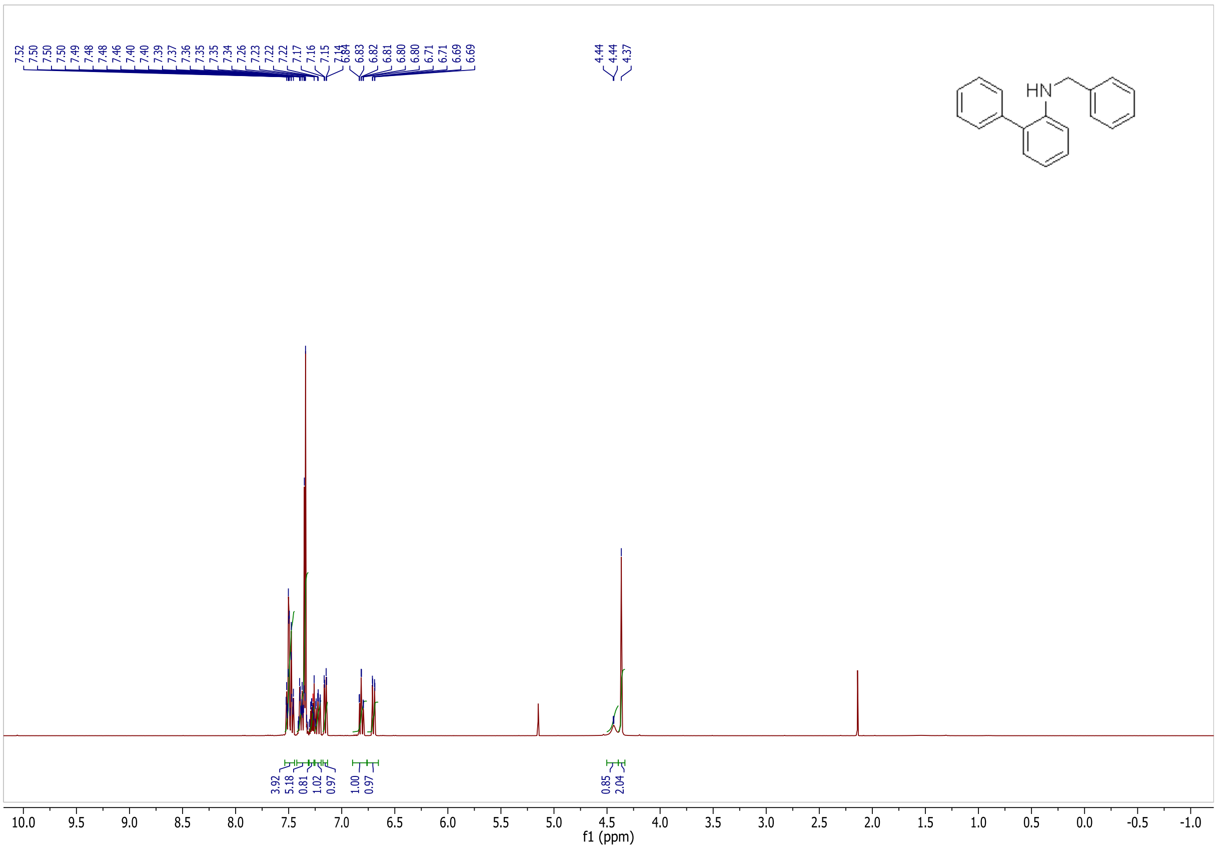

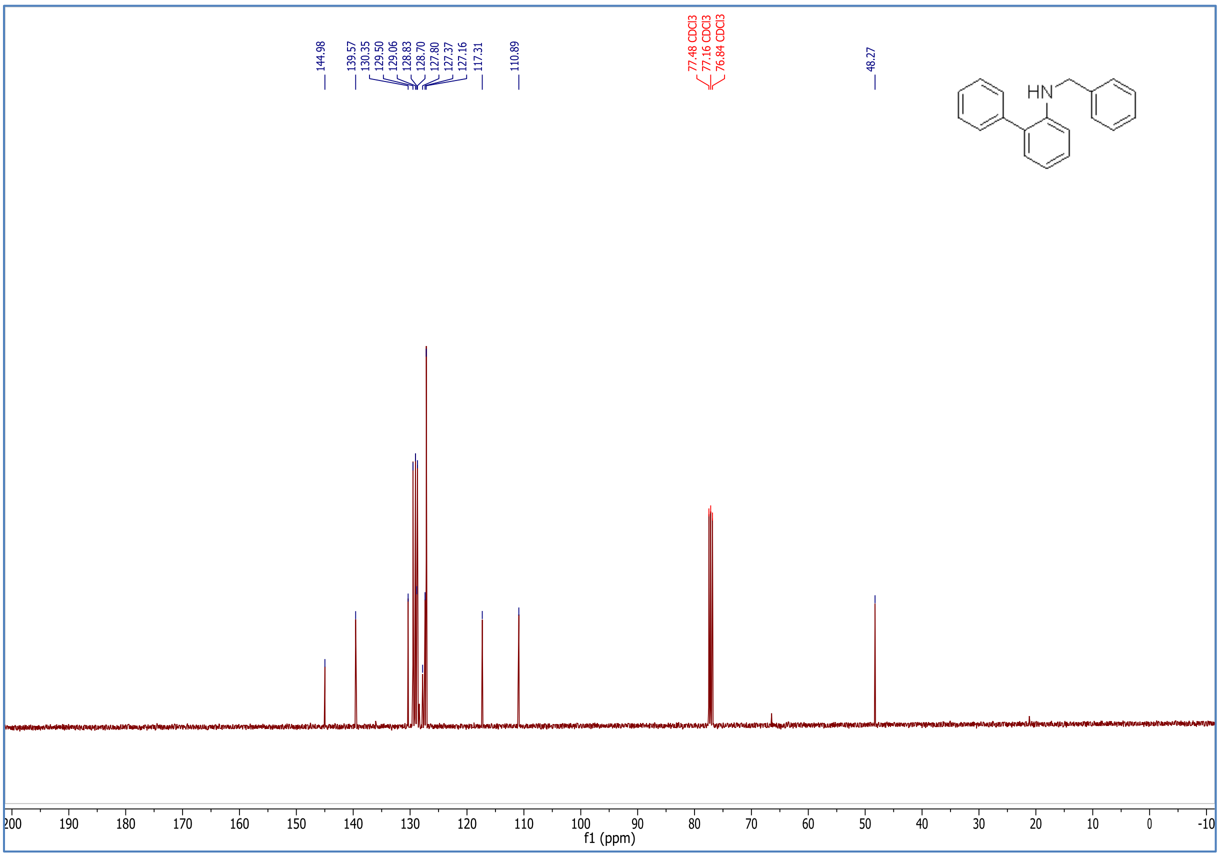

Supplementary Figure 29. ^{1}H and ^{13}C NMR of compound **5s**.

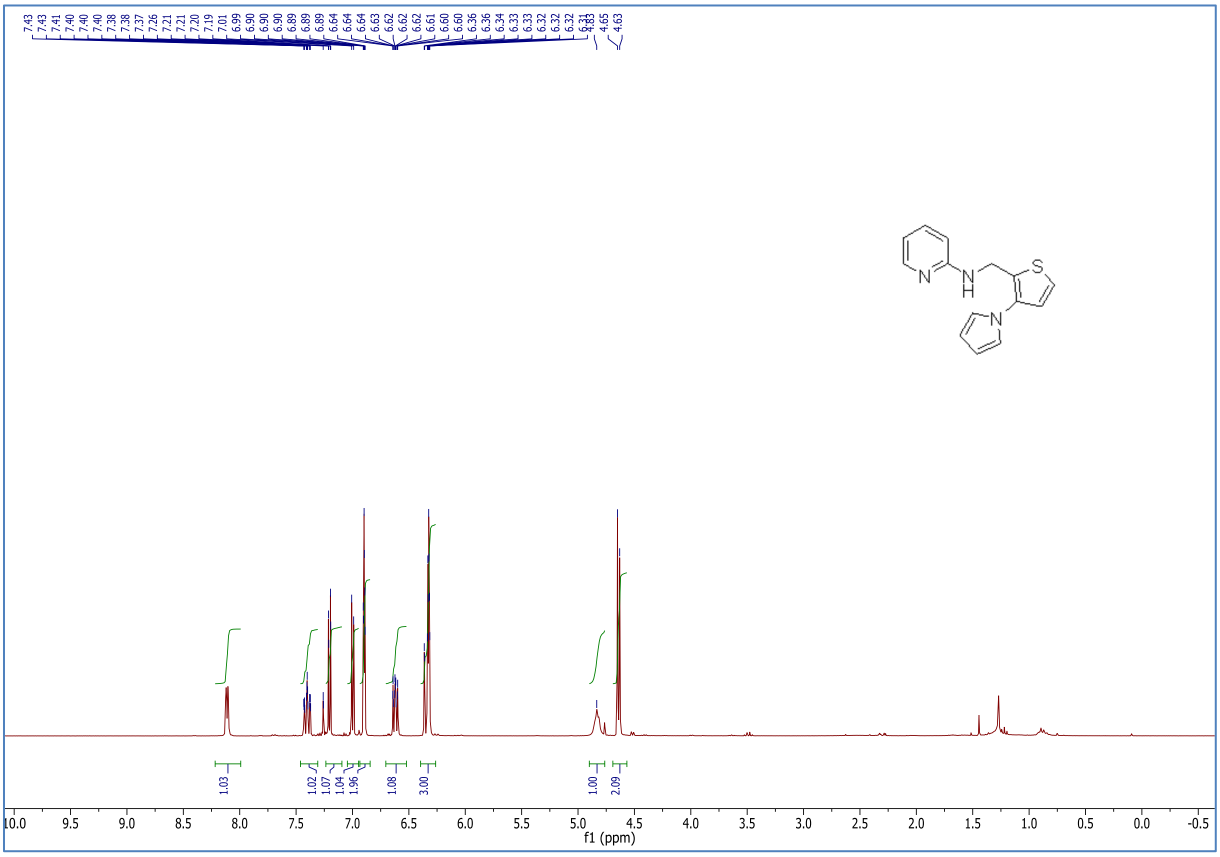

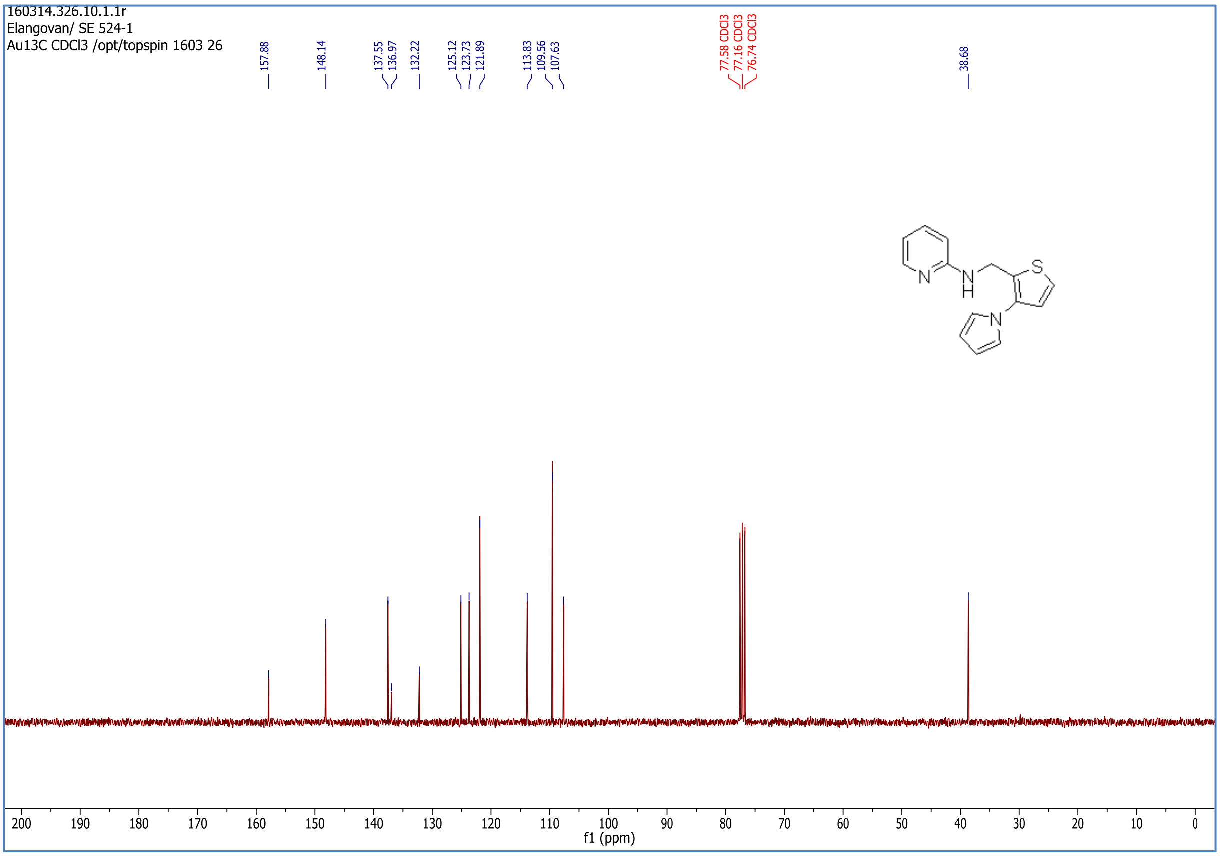

Supplementary Figure 30. ^{1}H and ^{13}C NMR of compound **6m**.

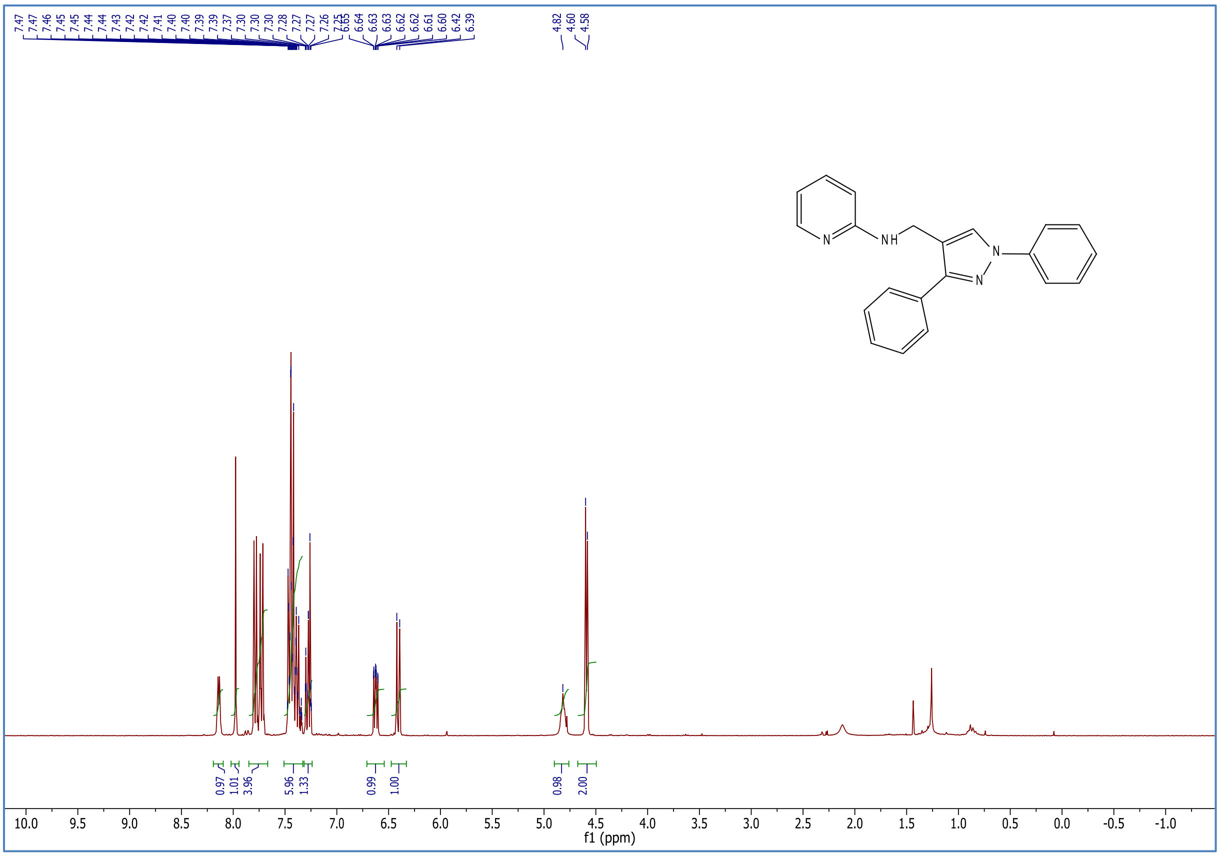

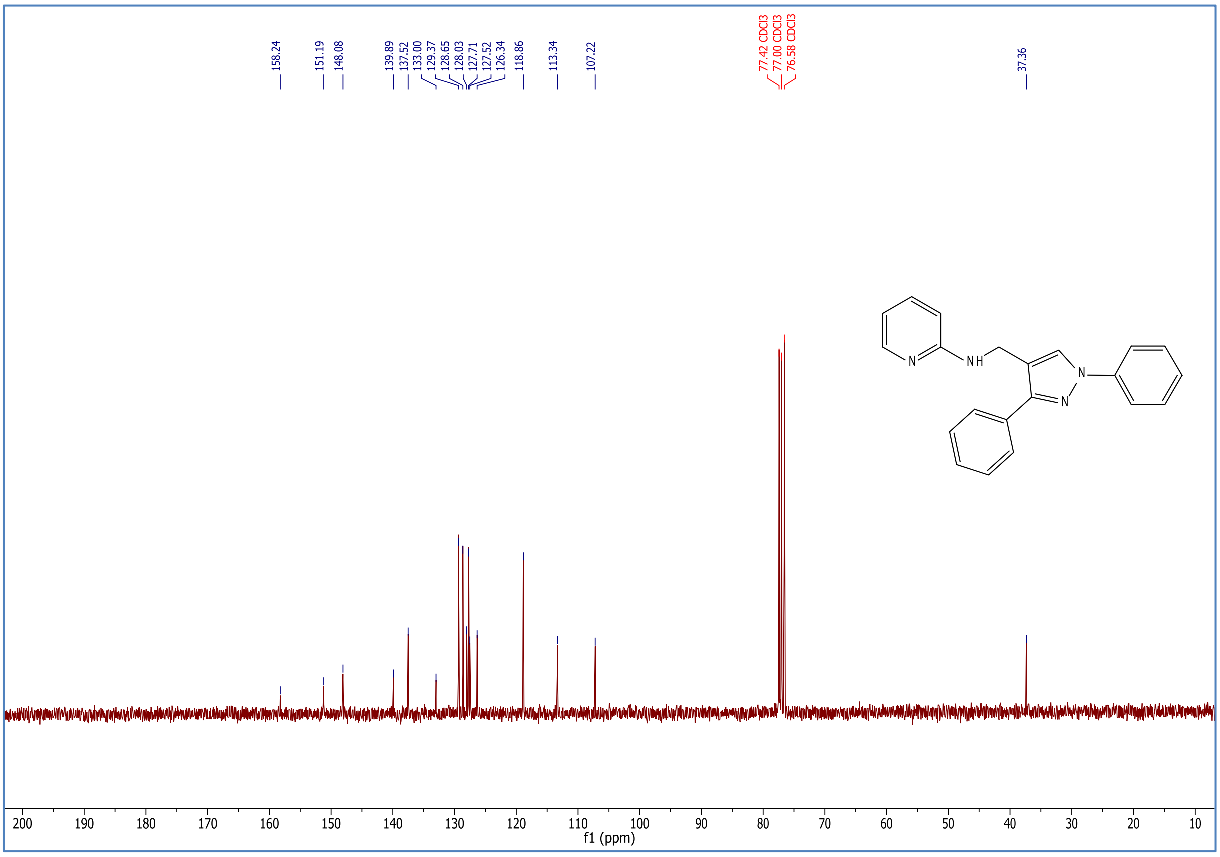

Supplementary Figure 31. ^{1}H and ^{13}C NMR of compound **6n**.

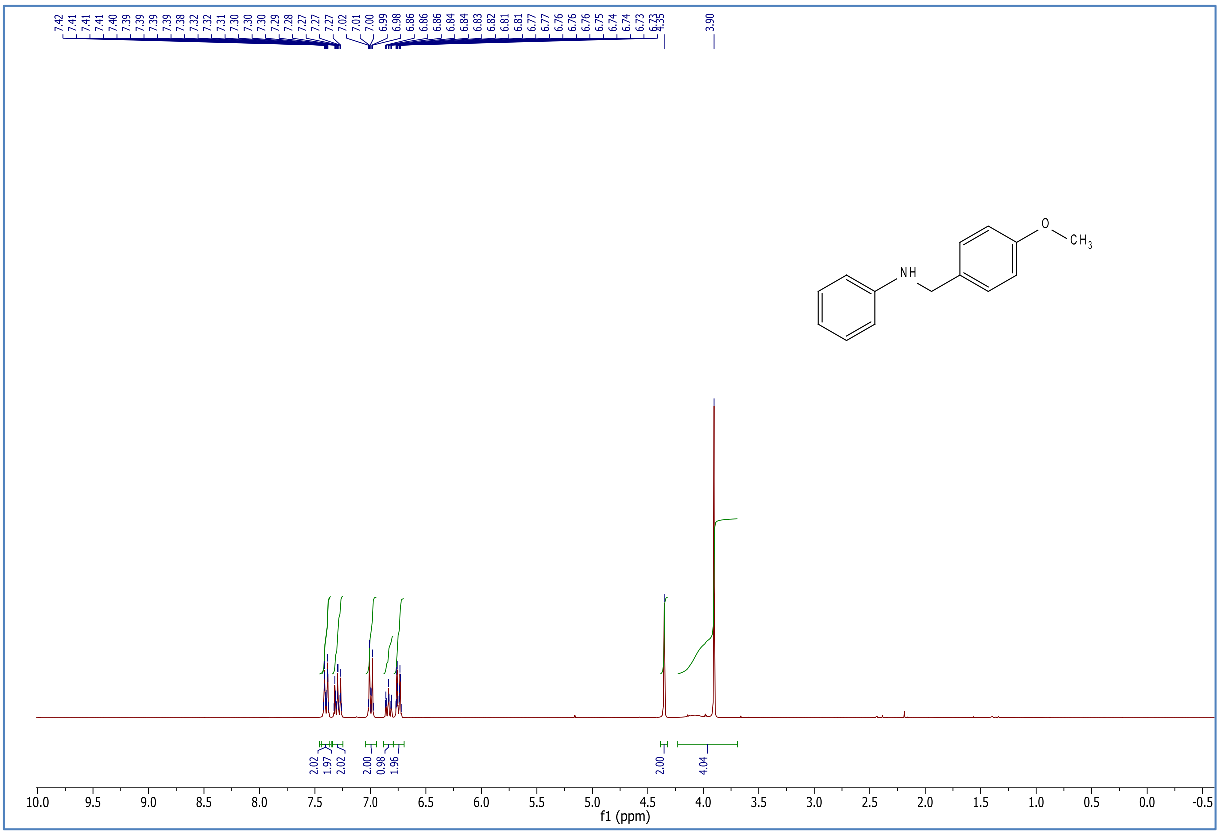

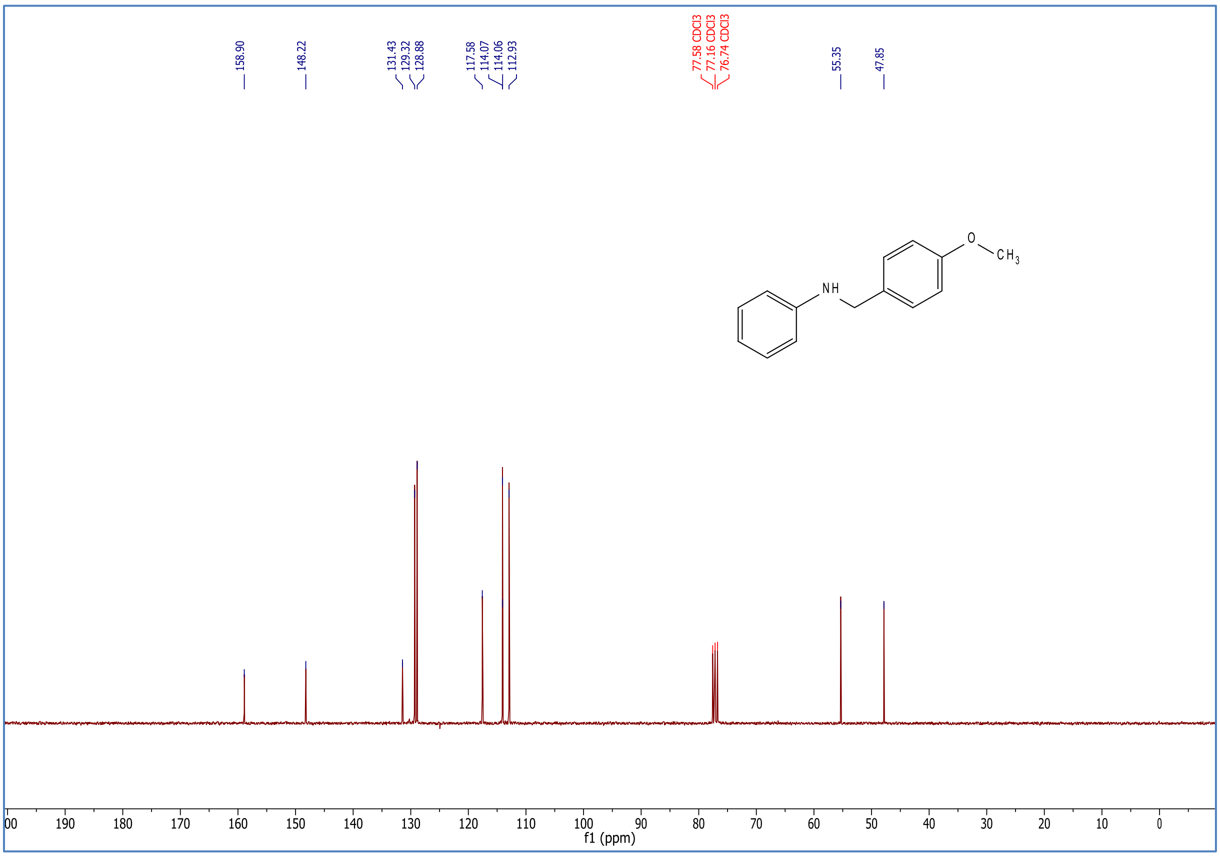

Supplementary Figure 32. ^{1}H and ^{13}C NMR of compound **6a**.

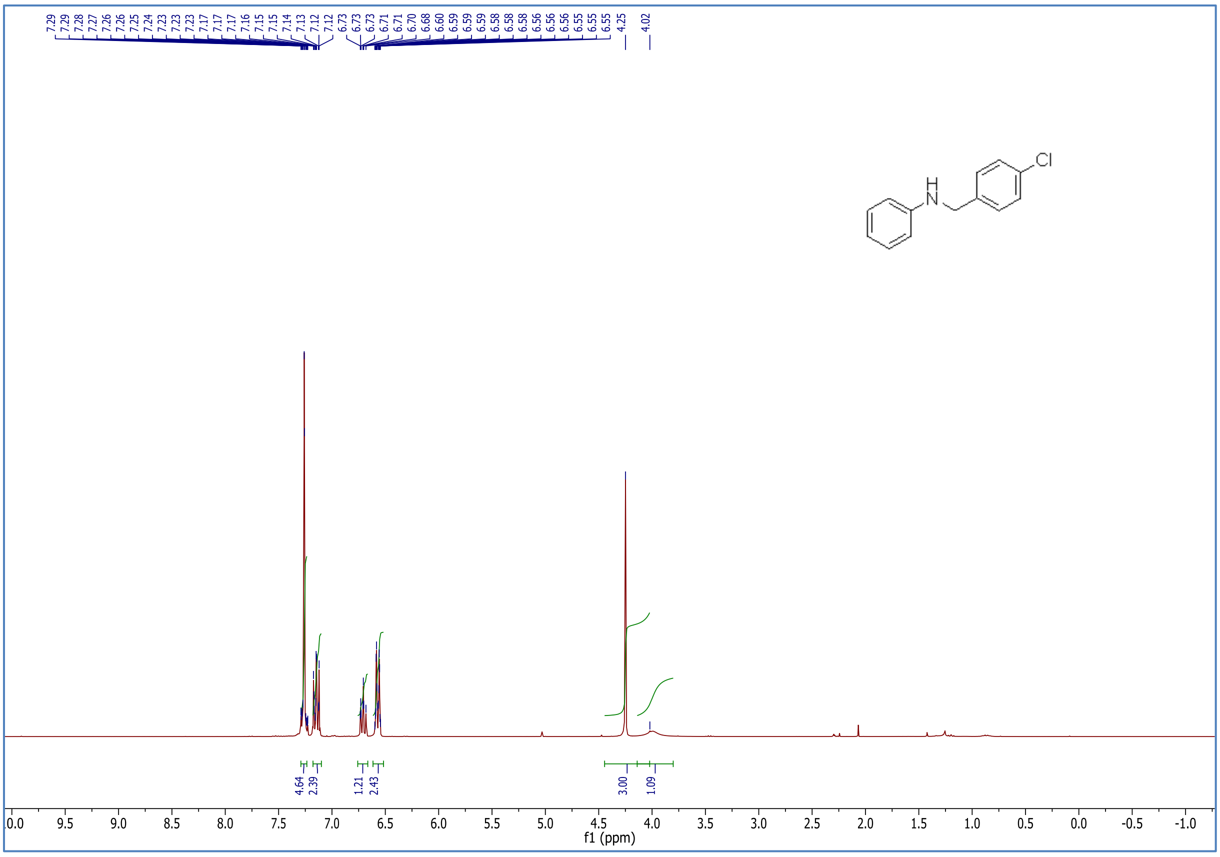

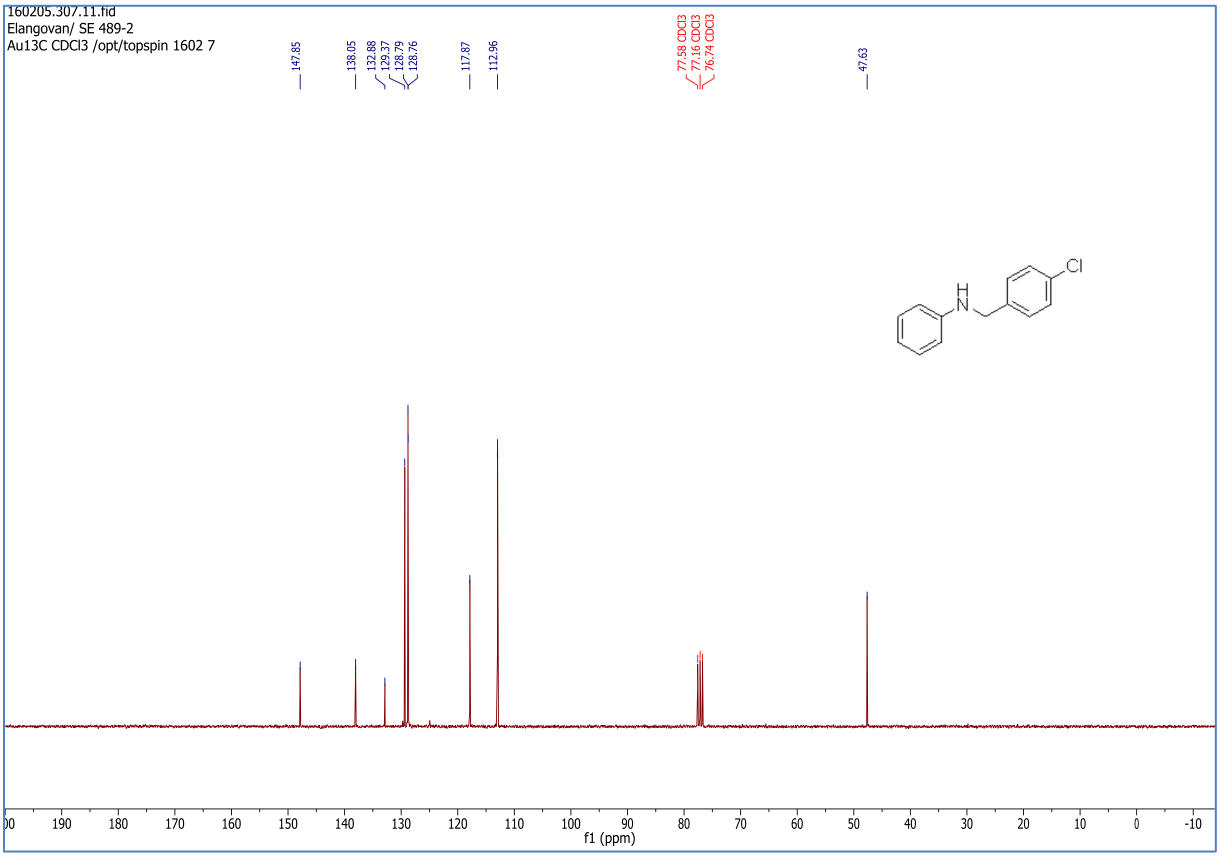

Supplementary Figure 33. ^{1}H and ^{13}C NMR of compound **6d**.

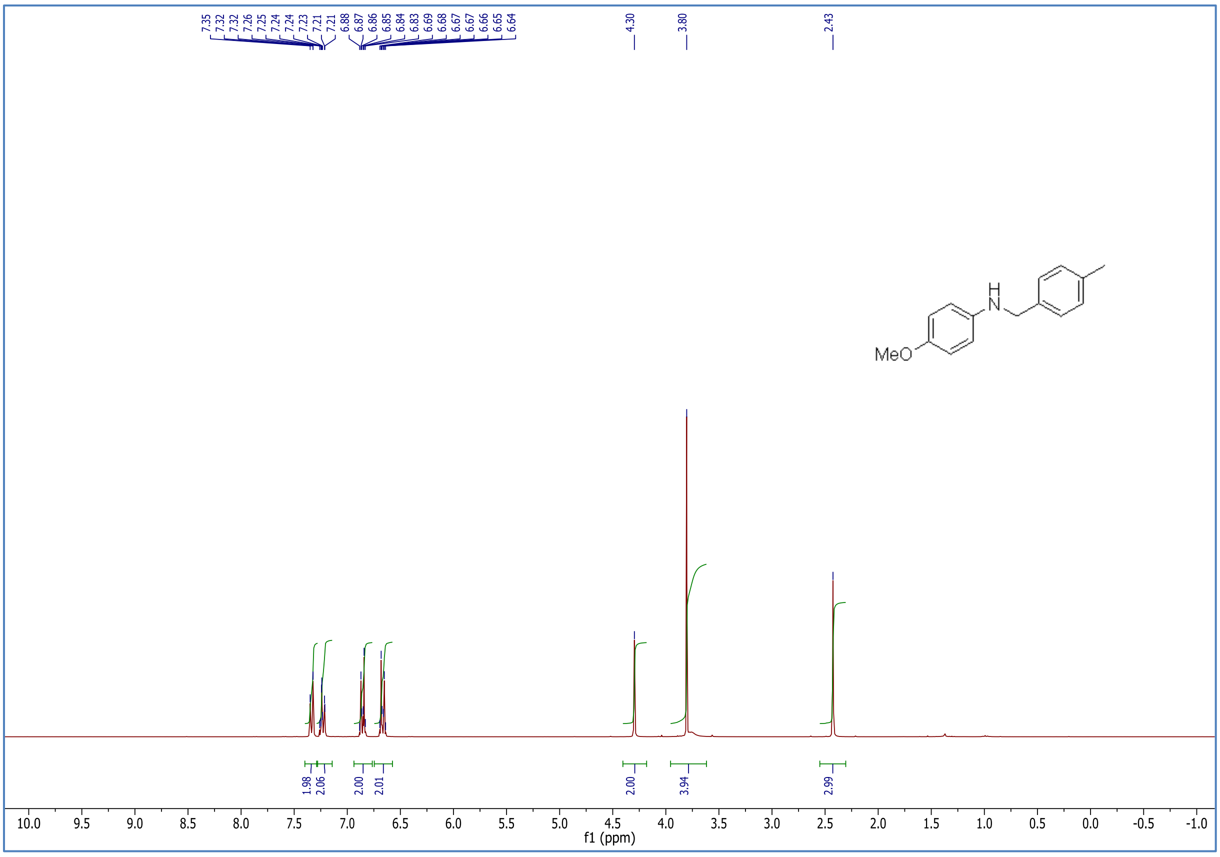

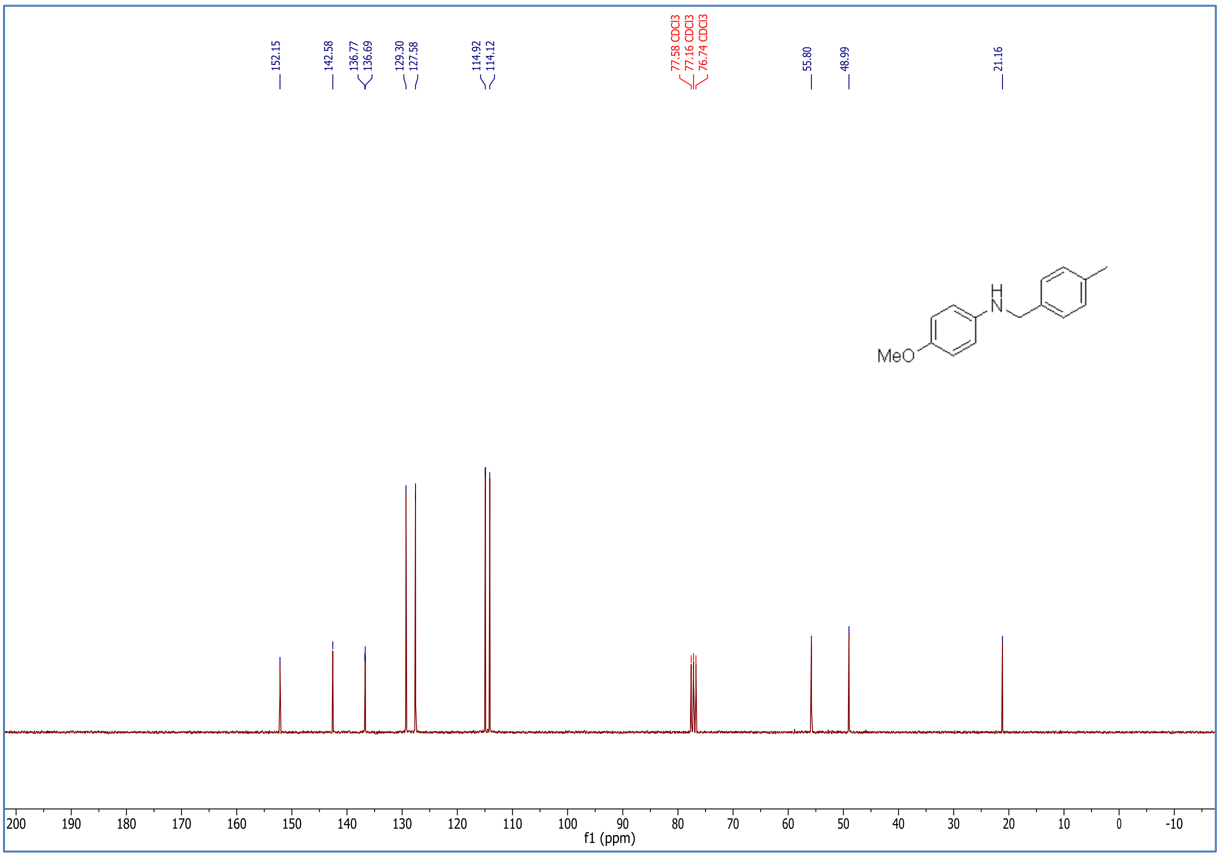

Supplementary Figure 34. ^{1}H and ^{13}C NMR of compound **6b**.

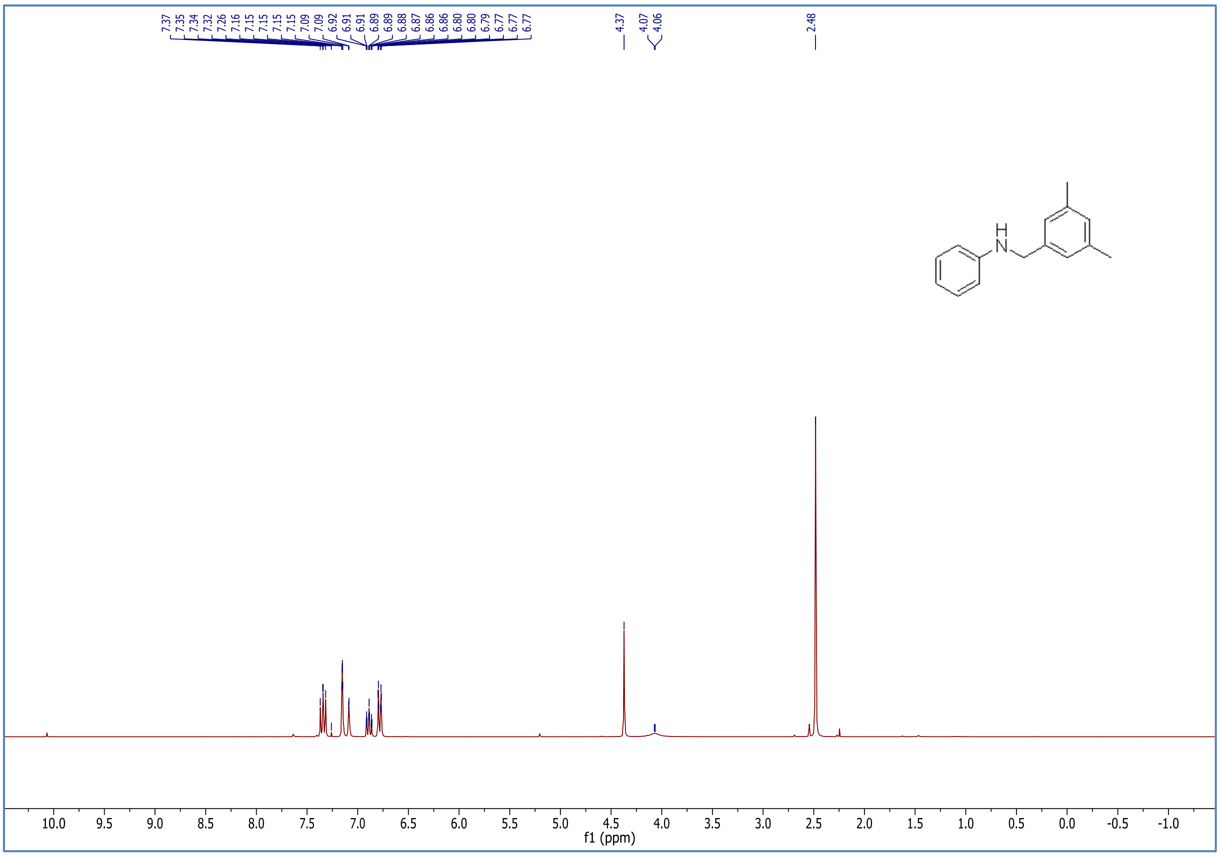

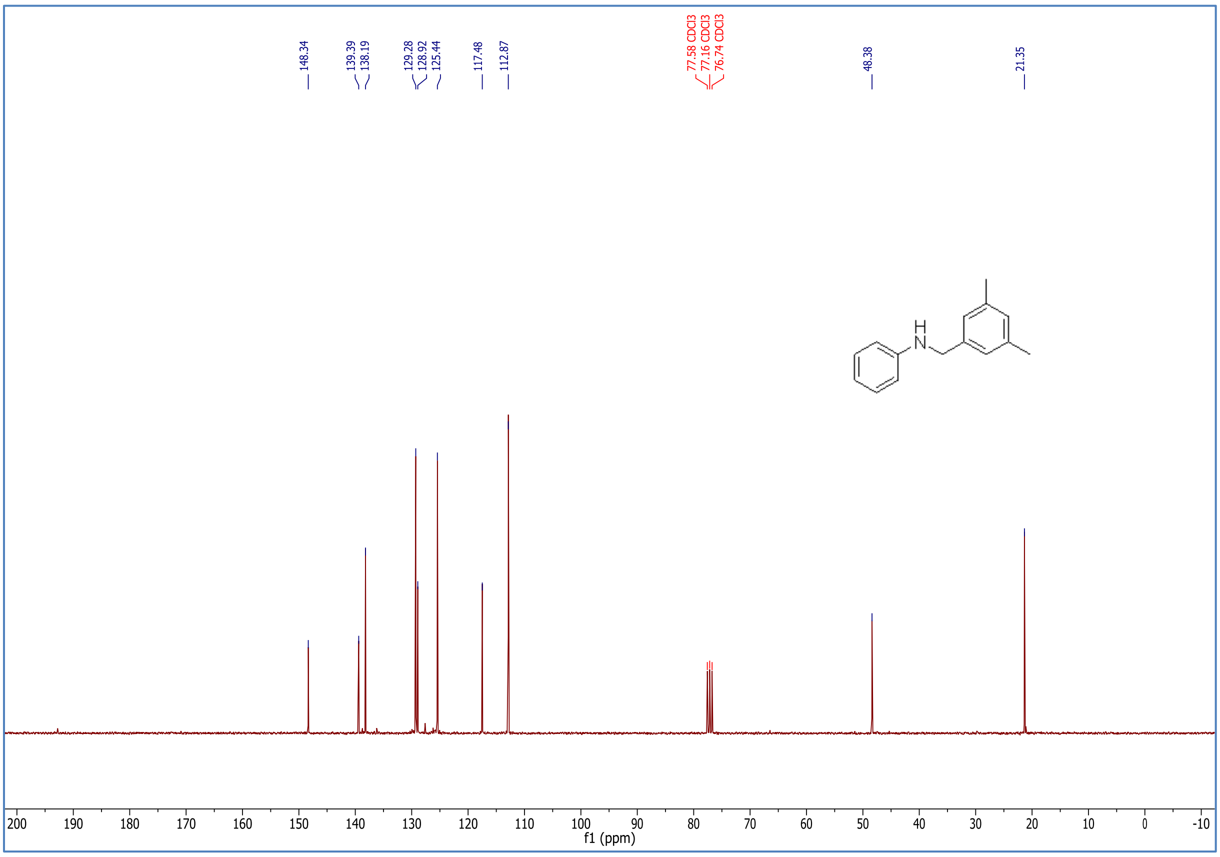

Supplementary Figure 35. ^{1}H and ^{13}C NMR of compound **6c**.

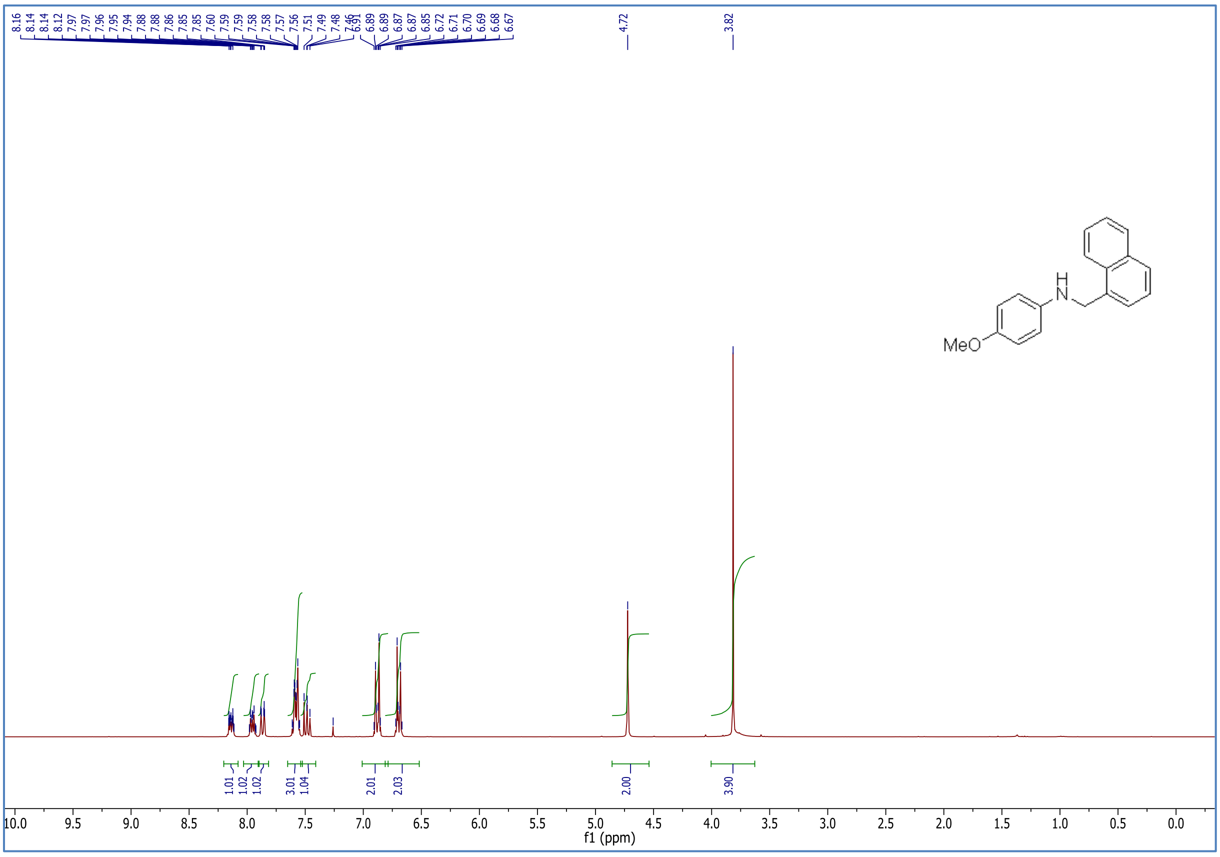

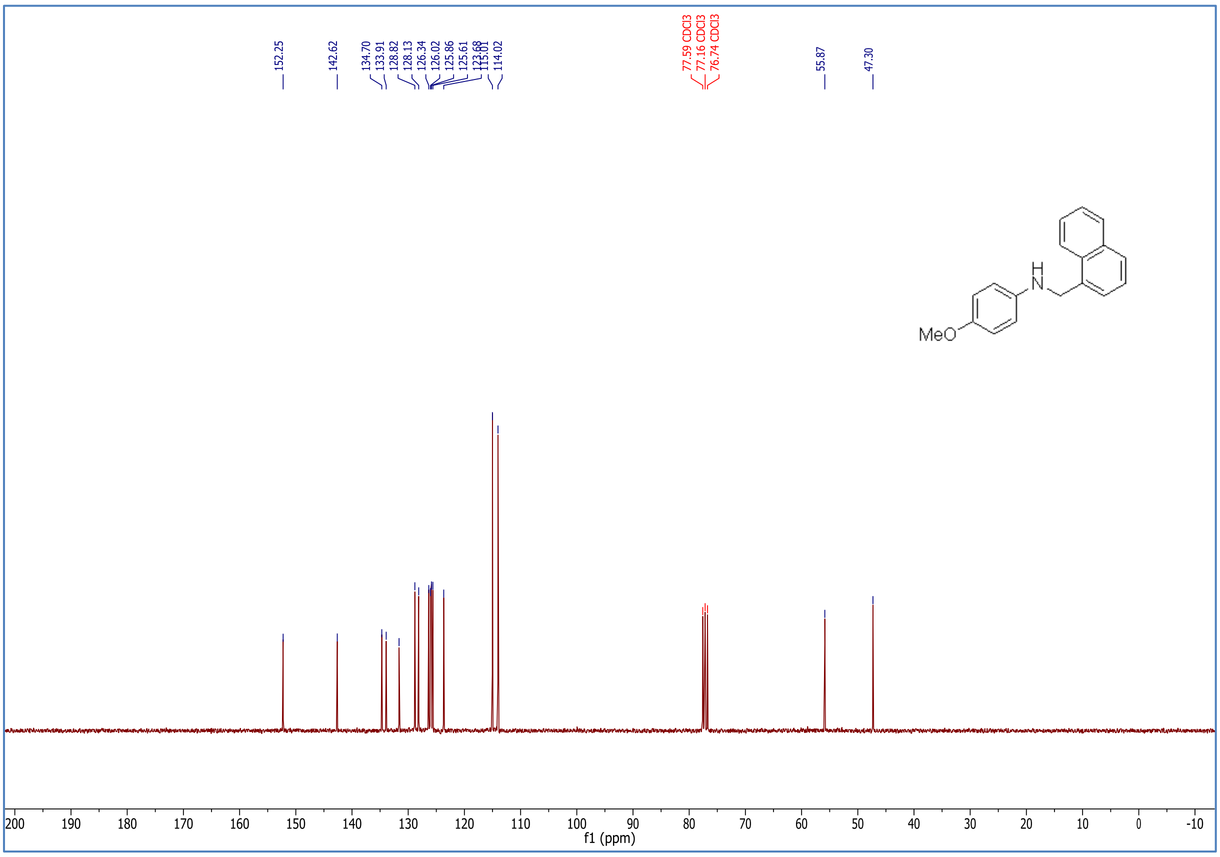

Supplementary Figure 36. ^{1}H and ^{13}C NMR of compound **6e**.

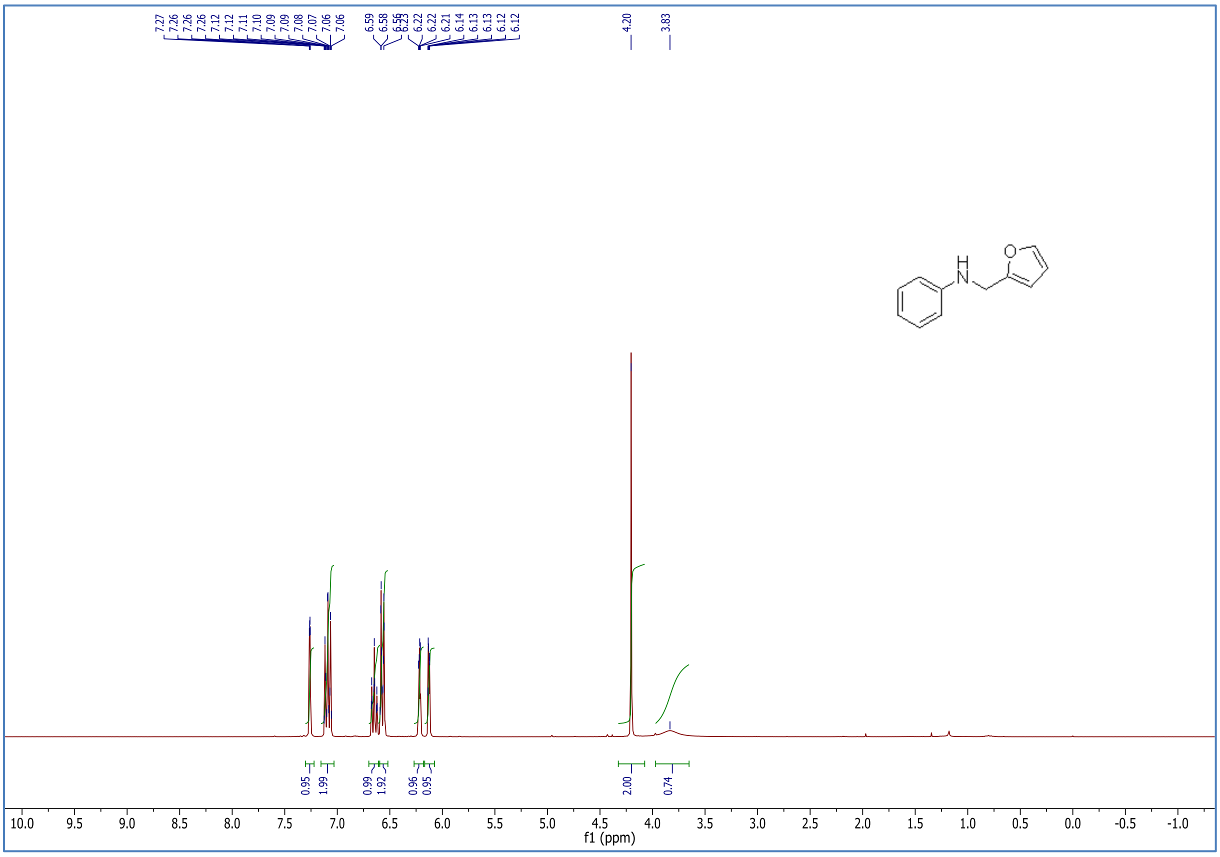

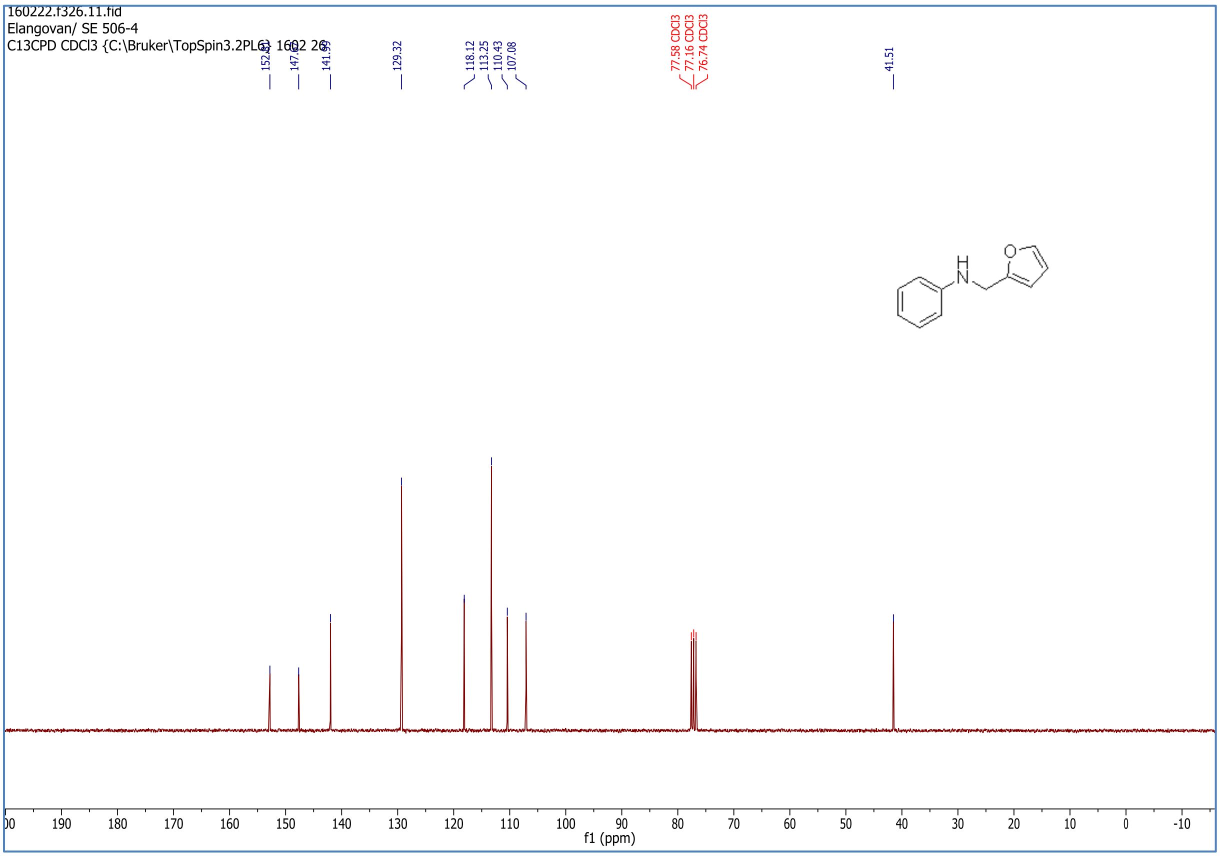

Supplementary Figure 37. ^{1}H and ^{13}C NMR of compound **6f**.

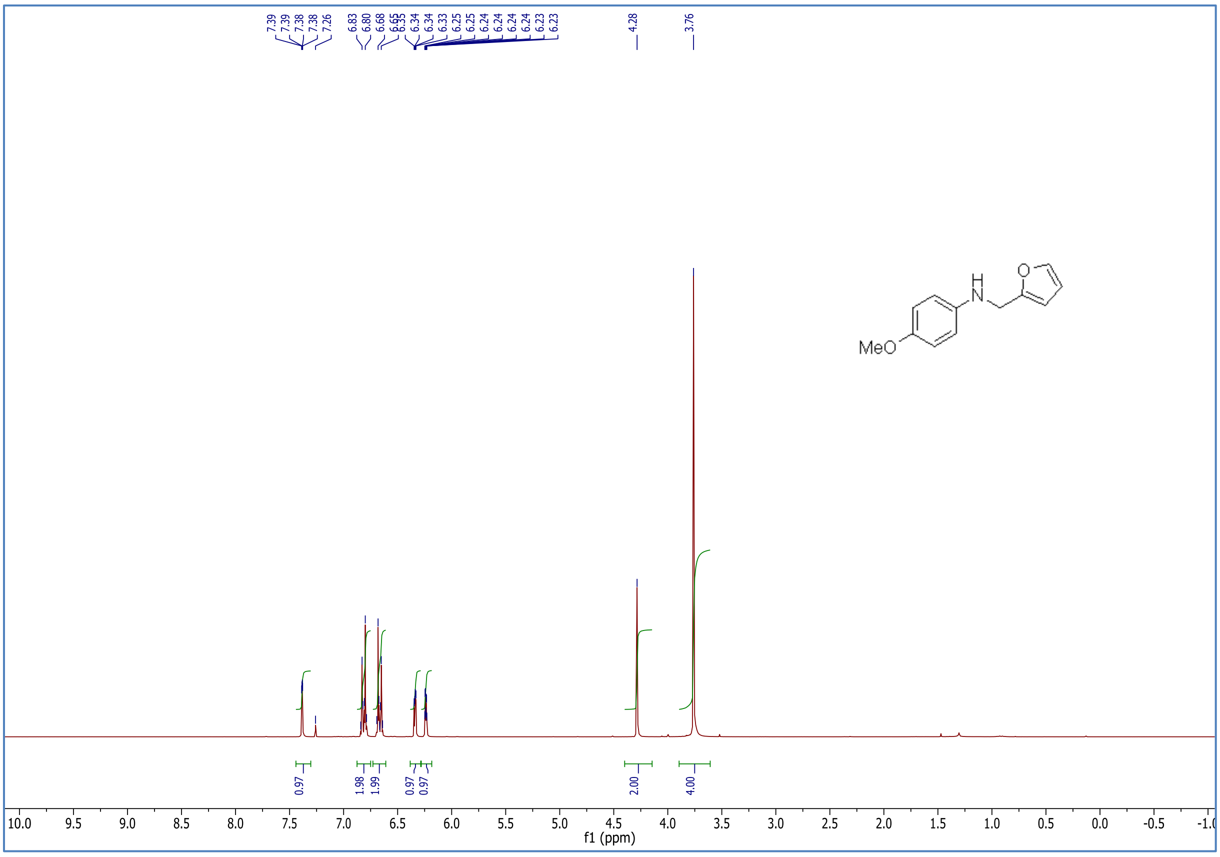

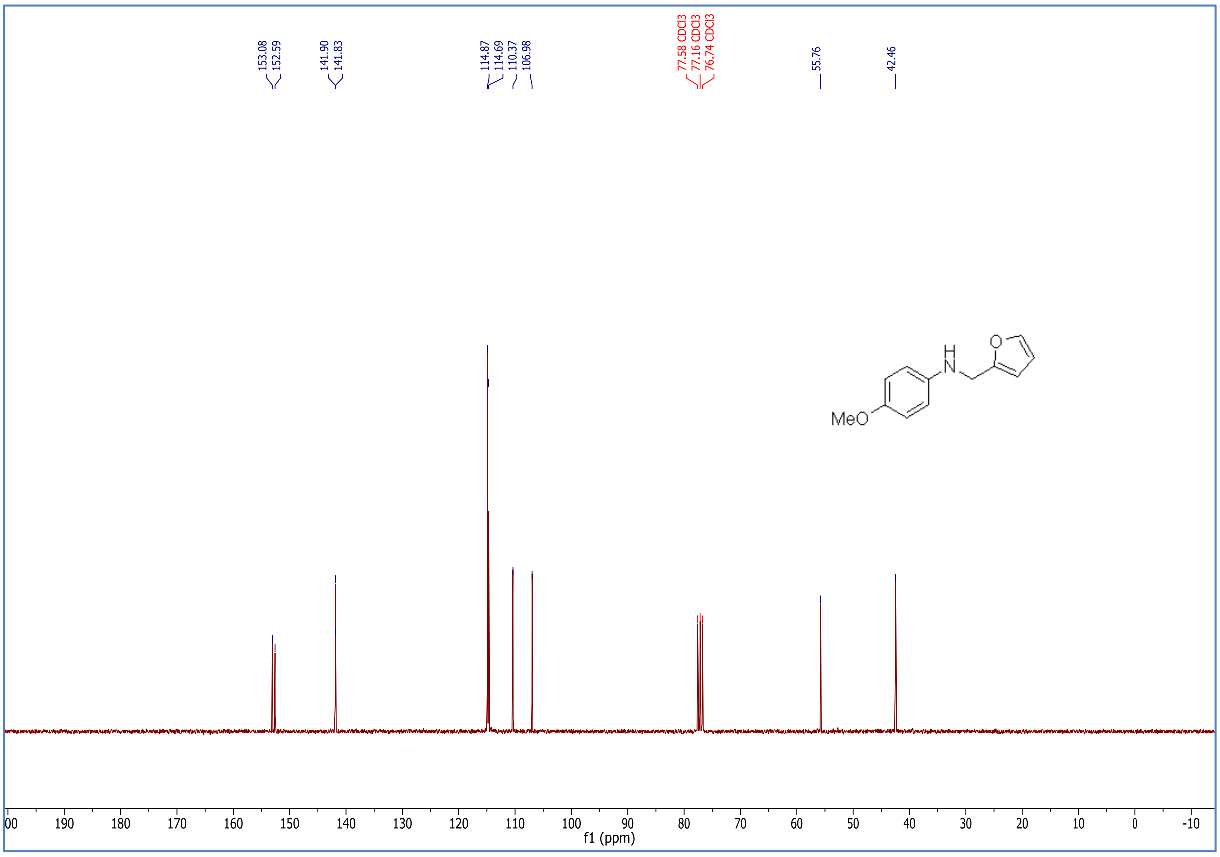

Supplementary Figure 38. ^{1}H and ^{13}C NMR of compound **6g**.

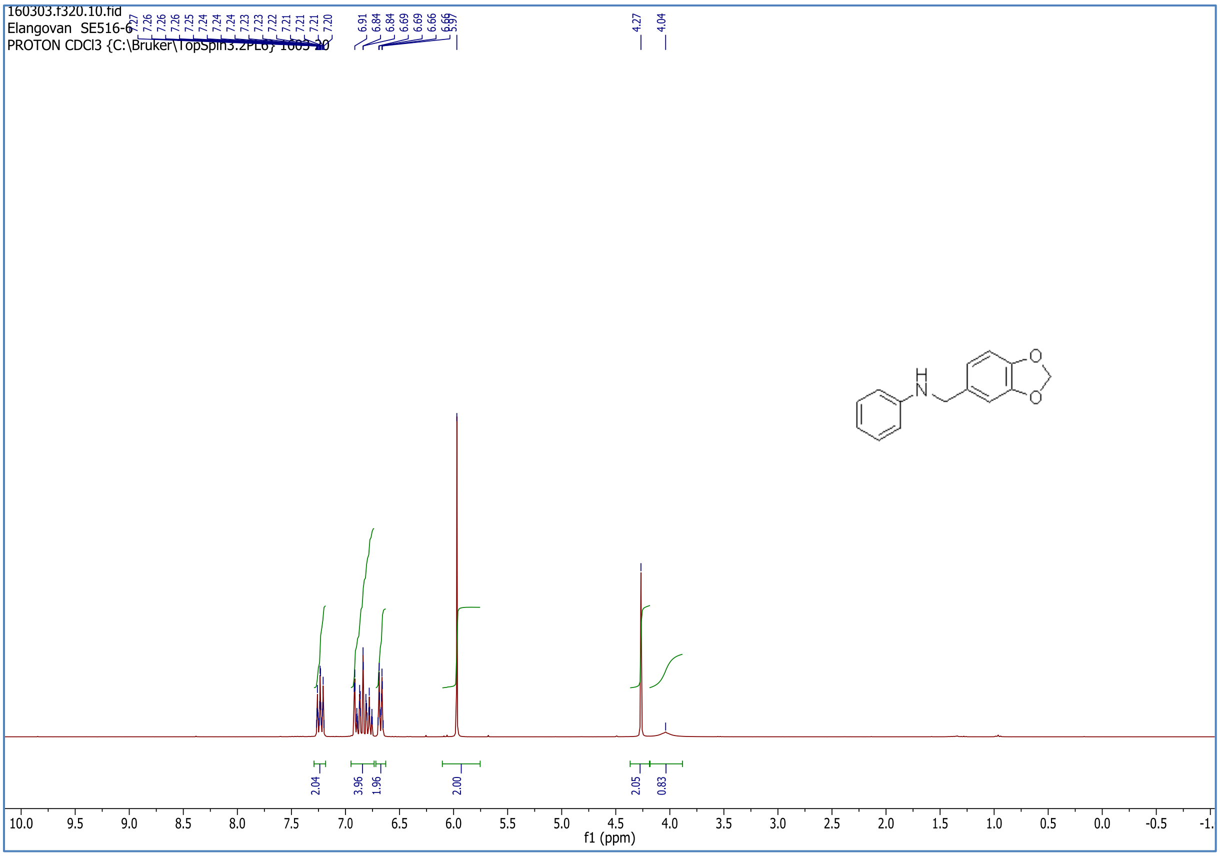

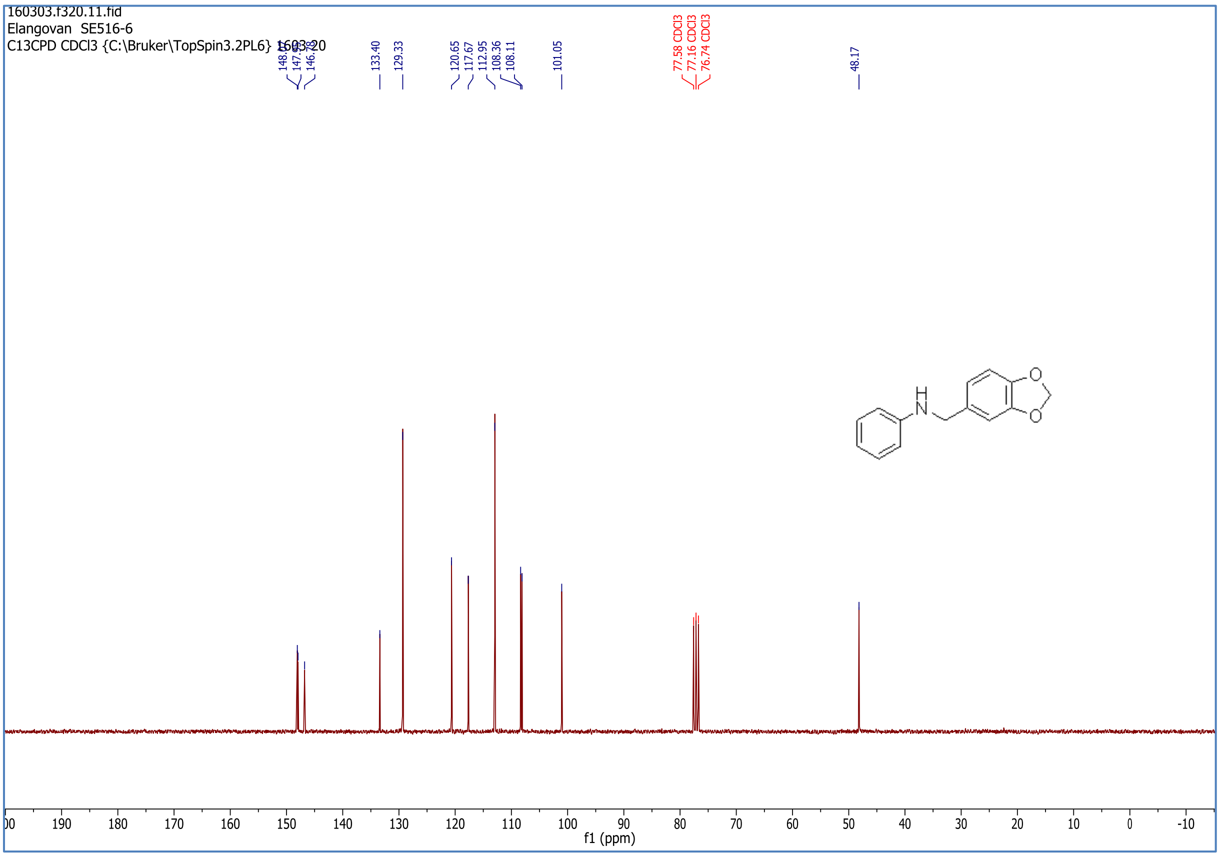

Supplementary Figure 39. ^{1}H and ^{13}C NMR of compound **6h**.

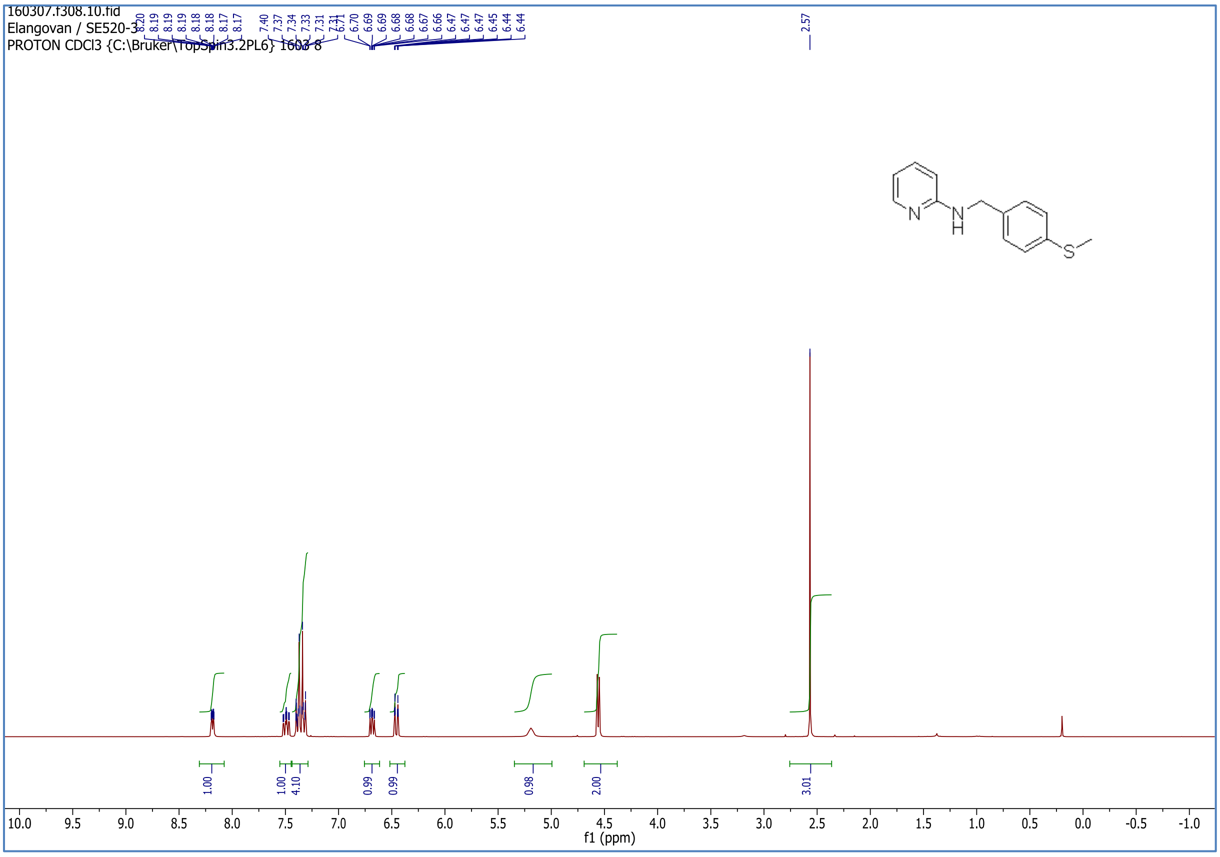

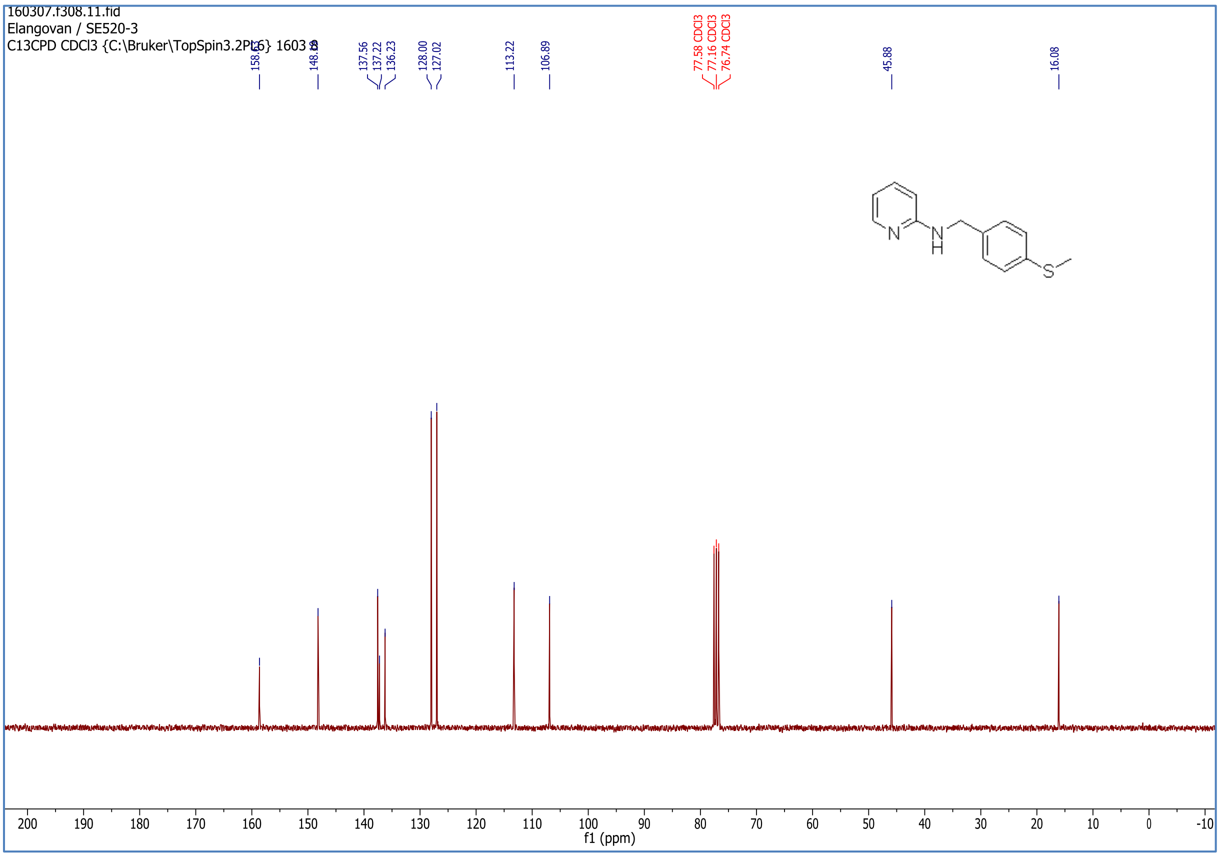

Supplementary Figure 40. ^{1}H and ^{13}C NMR of compound **6k**.

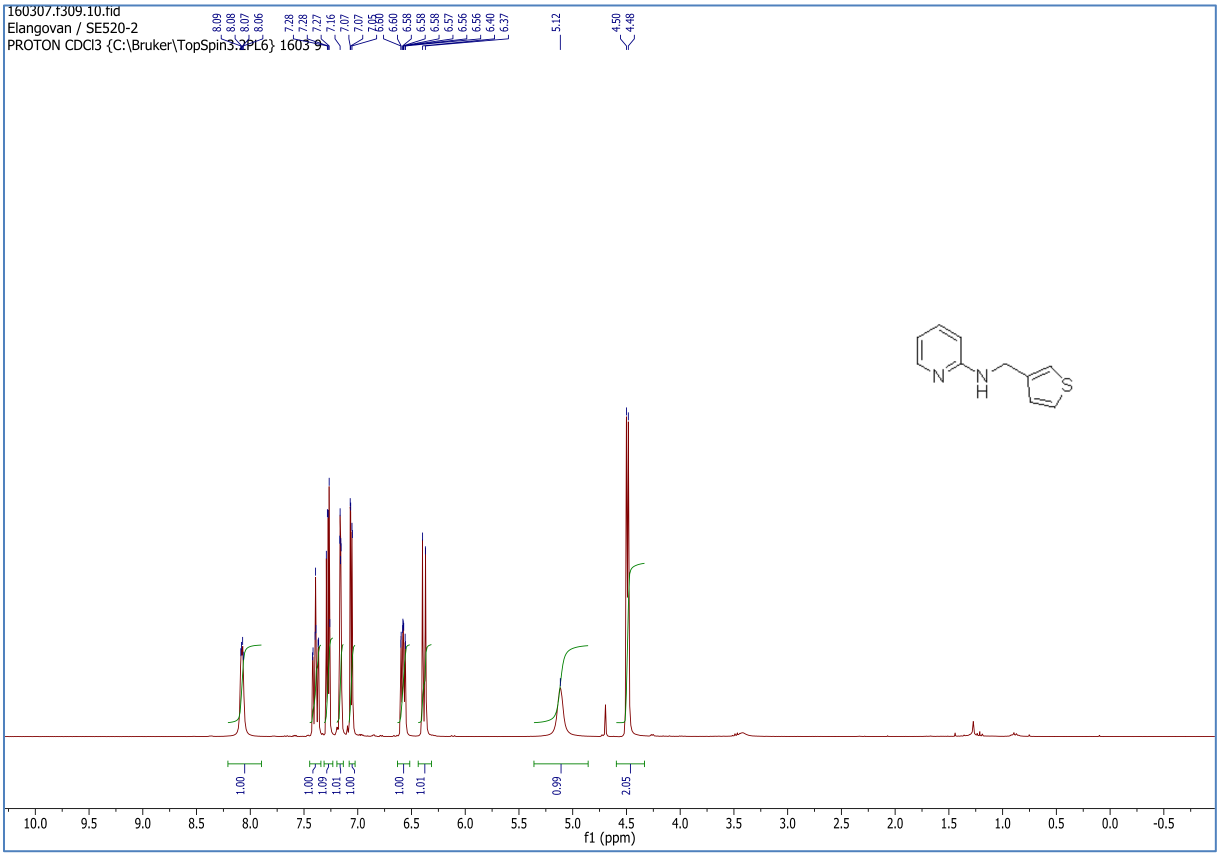

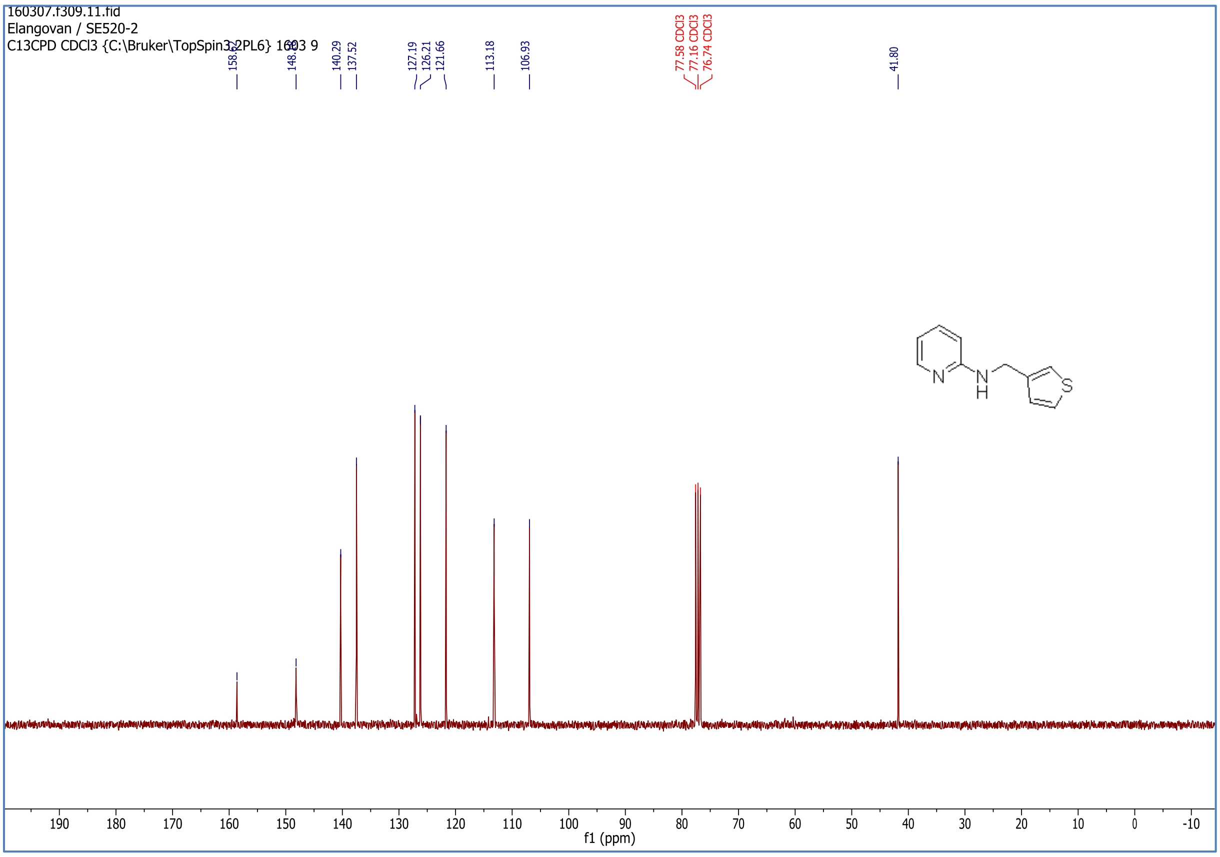

Supplementary Figure 41. ^{1}H and ^{13}C NMR of compound **6i**.

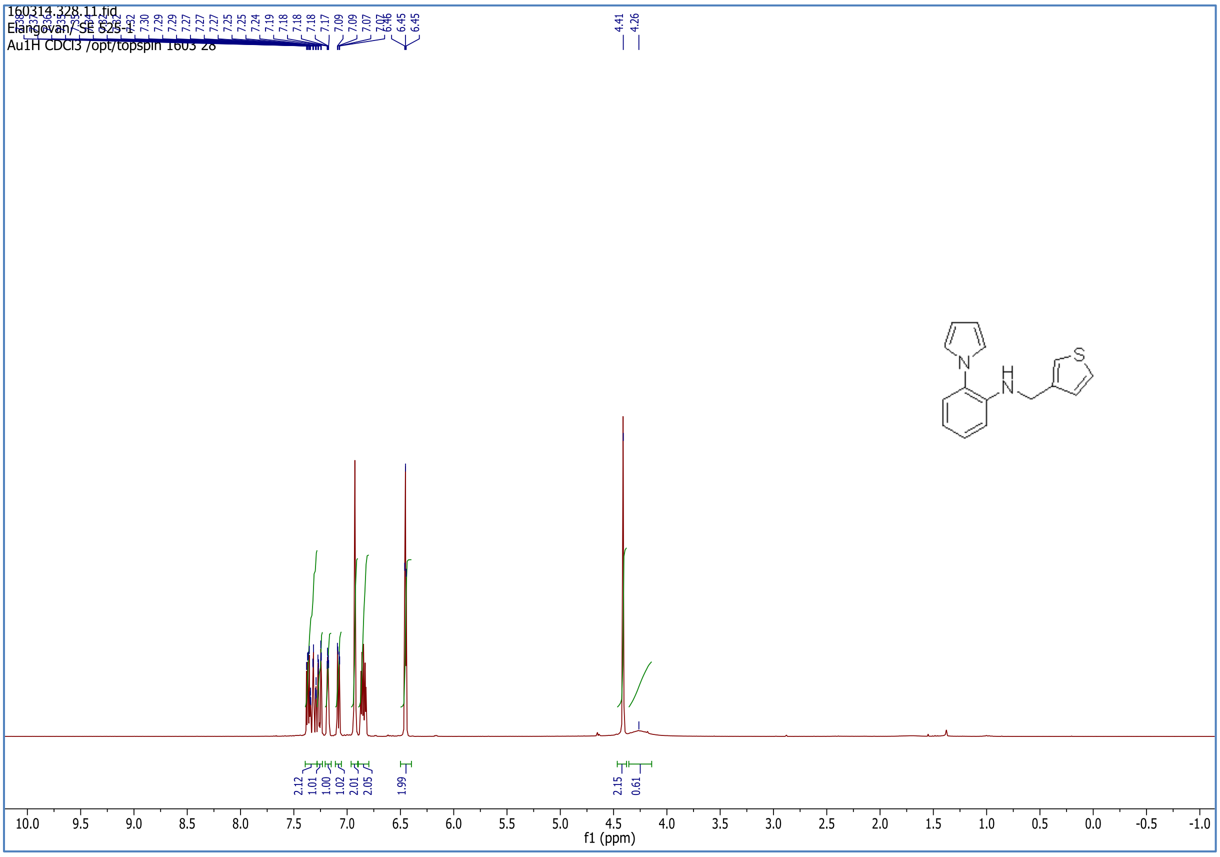

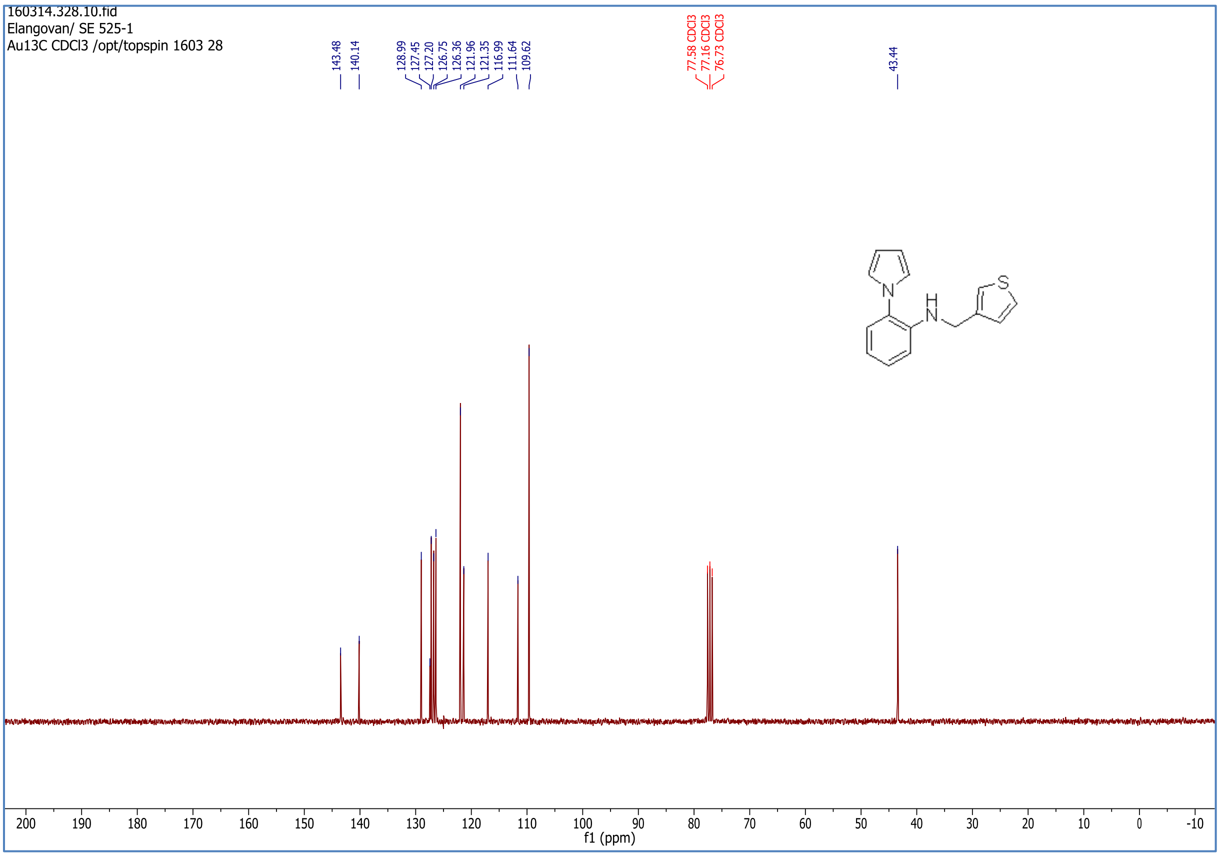

Supplementary Figure 42. ^{1}H and ^{13}C NMR of compound **6l**.

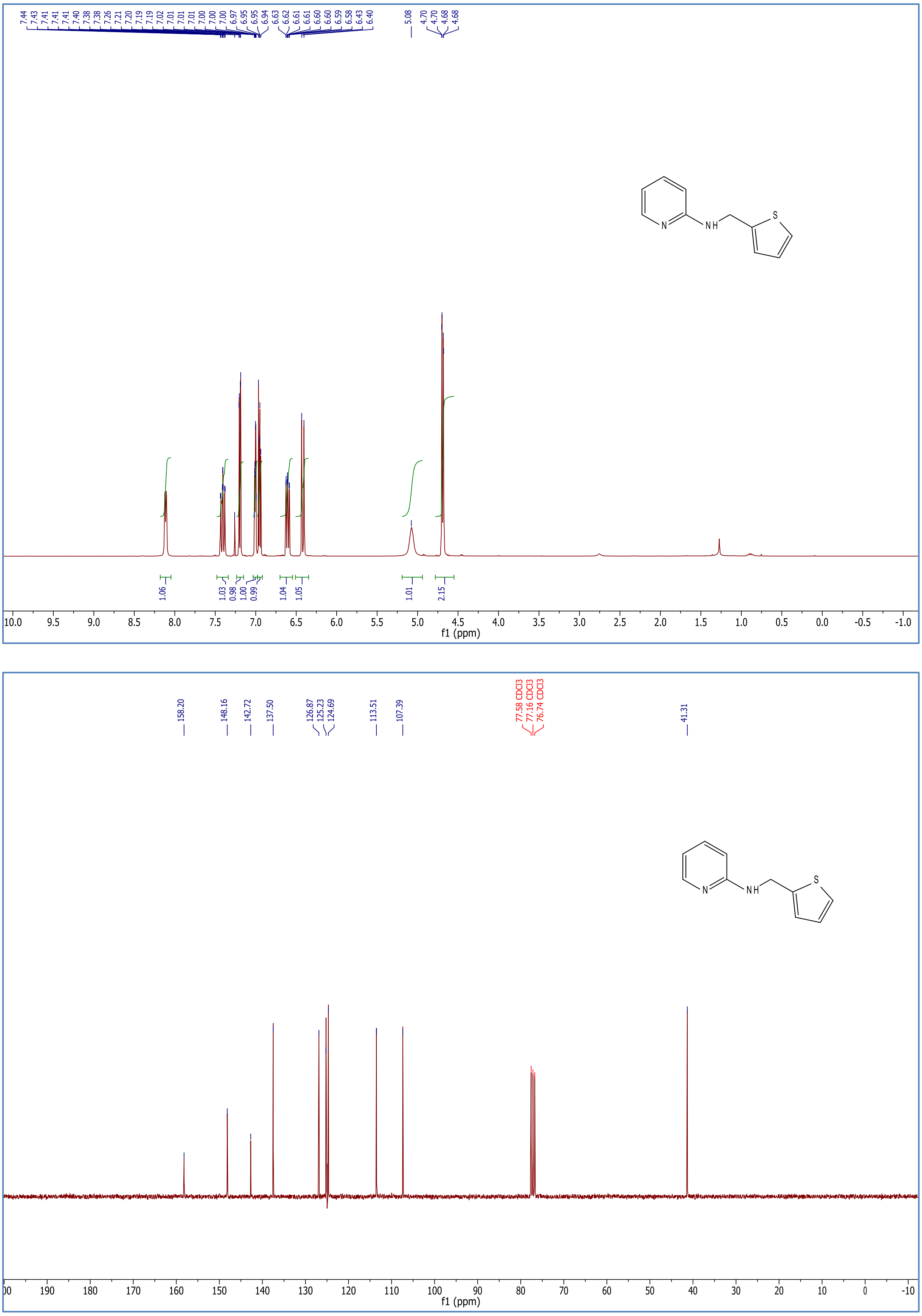

Supplementary Figure 43. ^{1}H and ^{13}C NMR of compound **6j**.

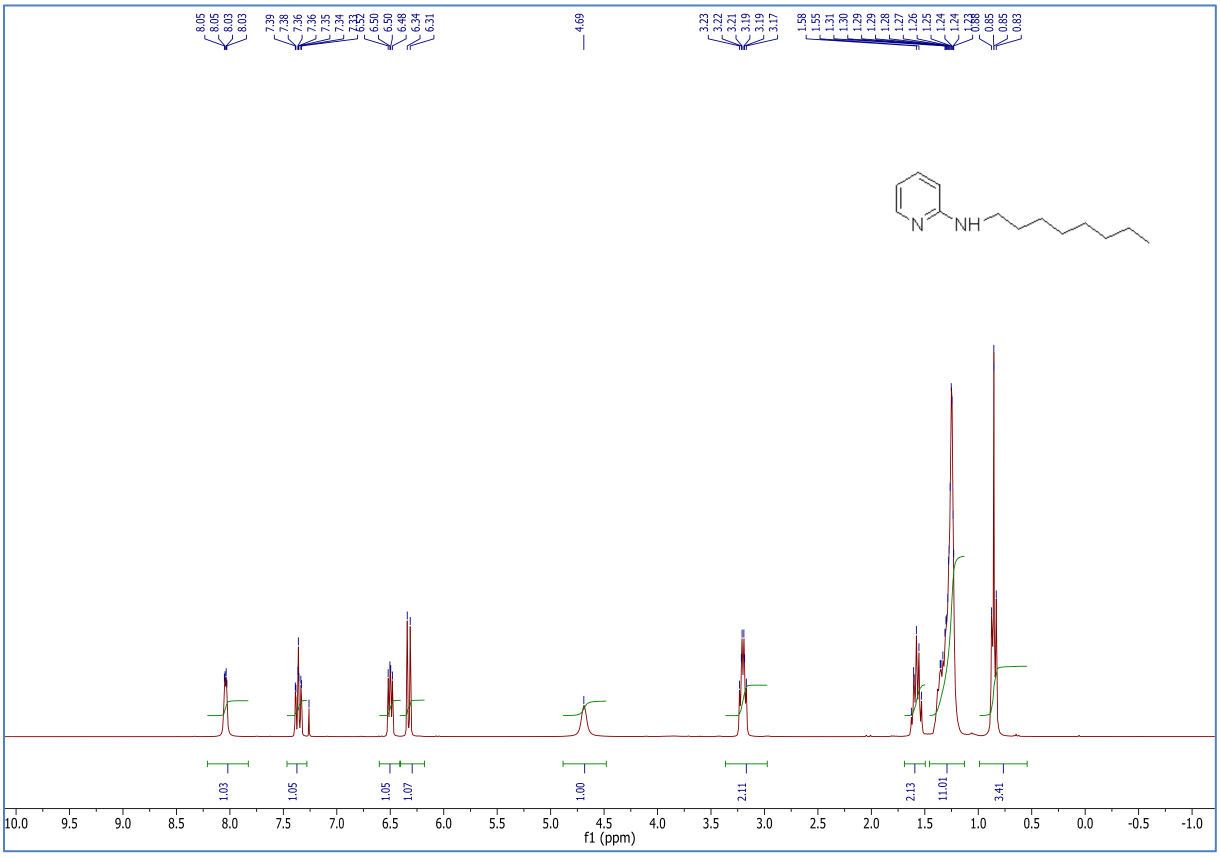

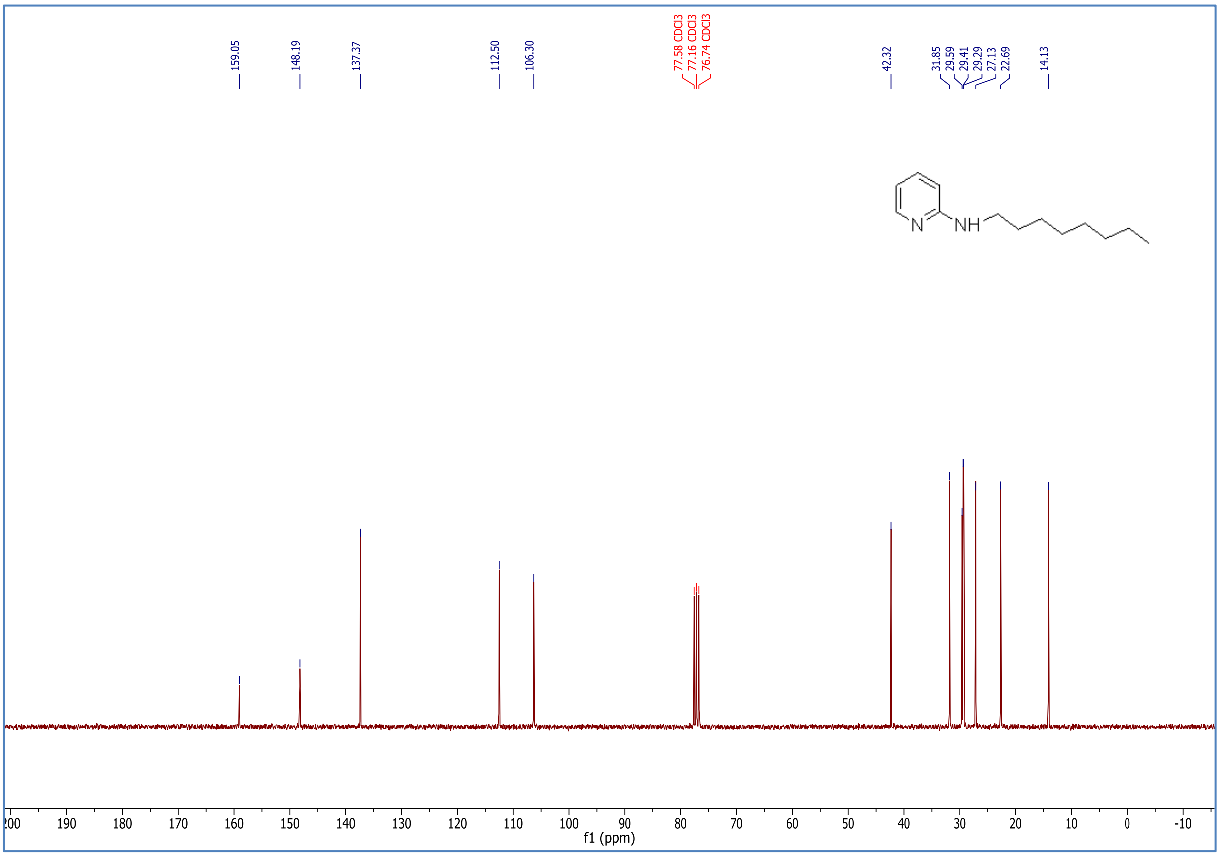

Supplementary Figure 44. ^{1}H and ^{13}C NMR of compound **6o**.

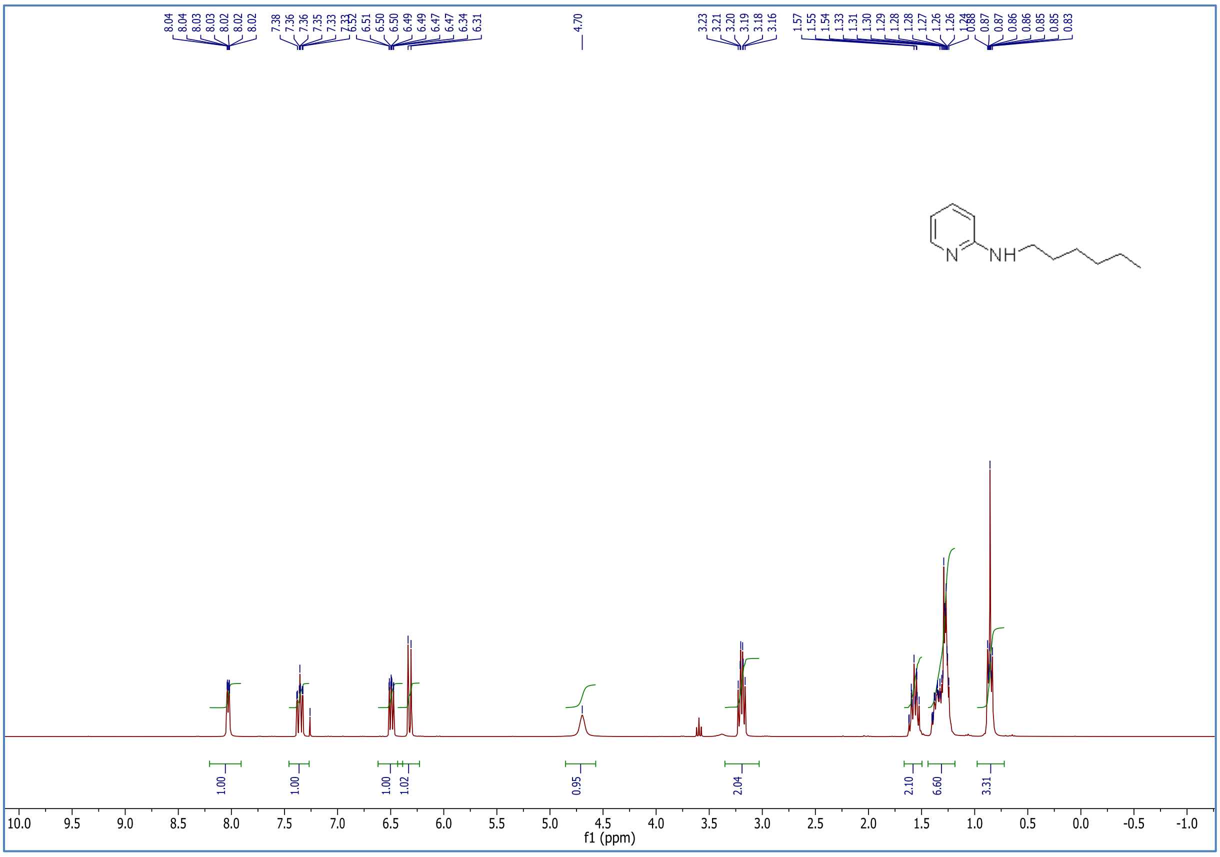

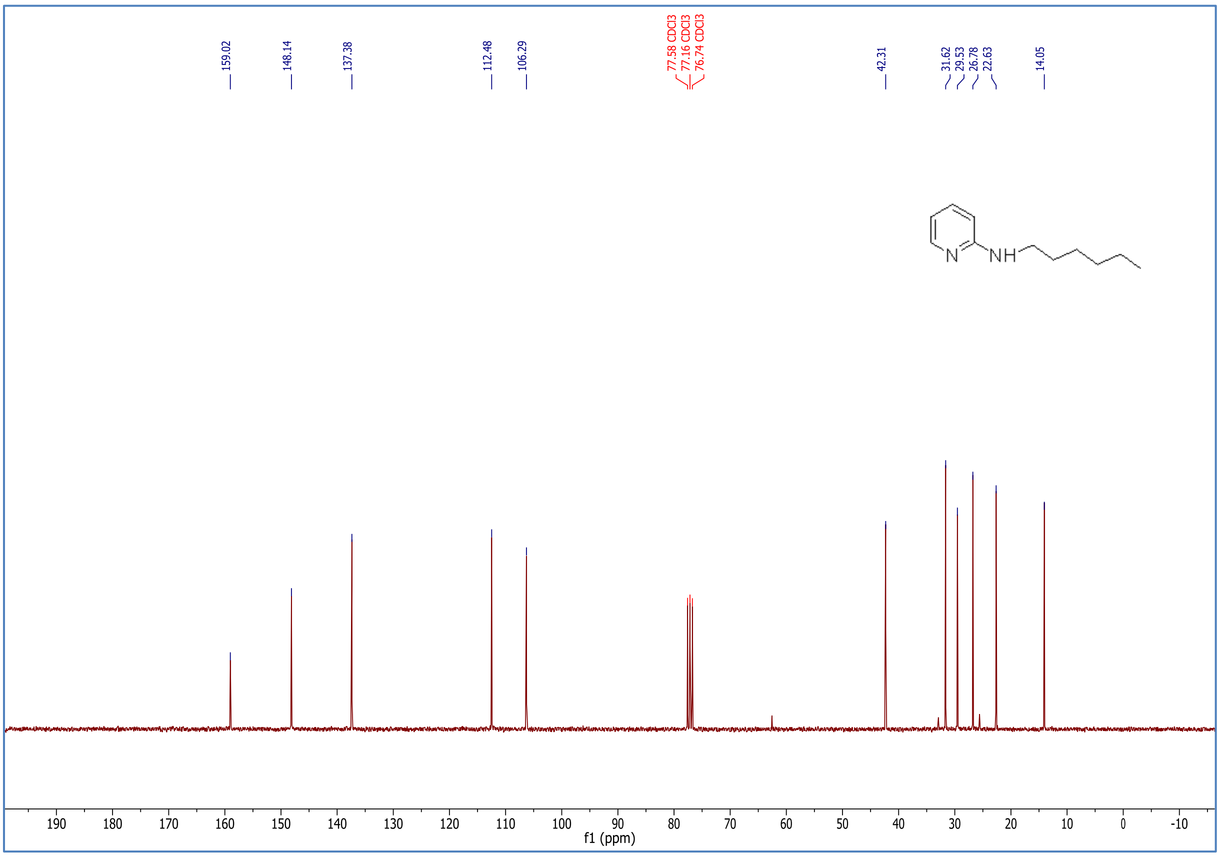

Supplementary Figure 45. ^{1}H and ^{13}C NMR of compound **6p**.

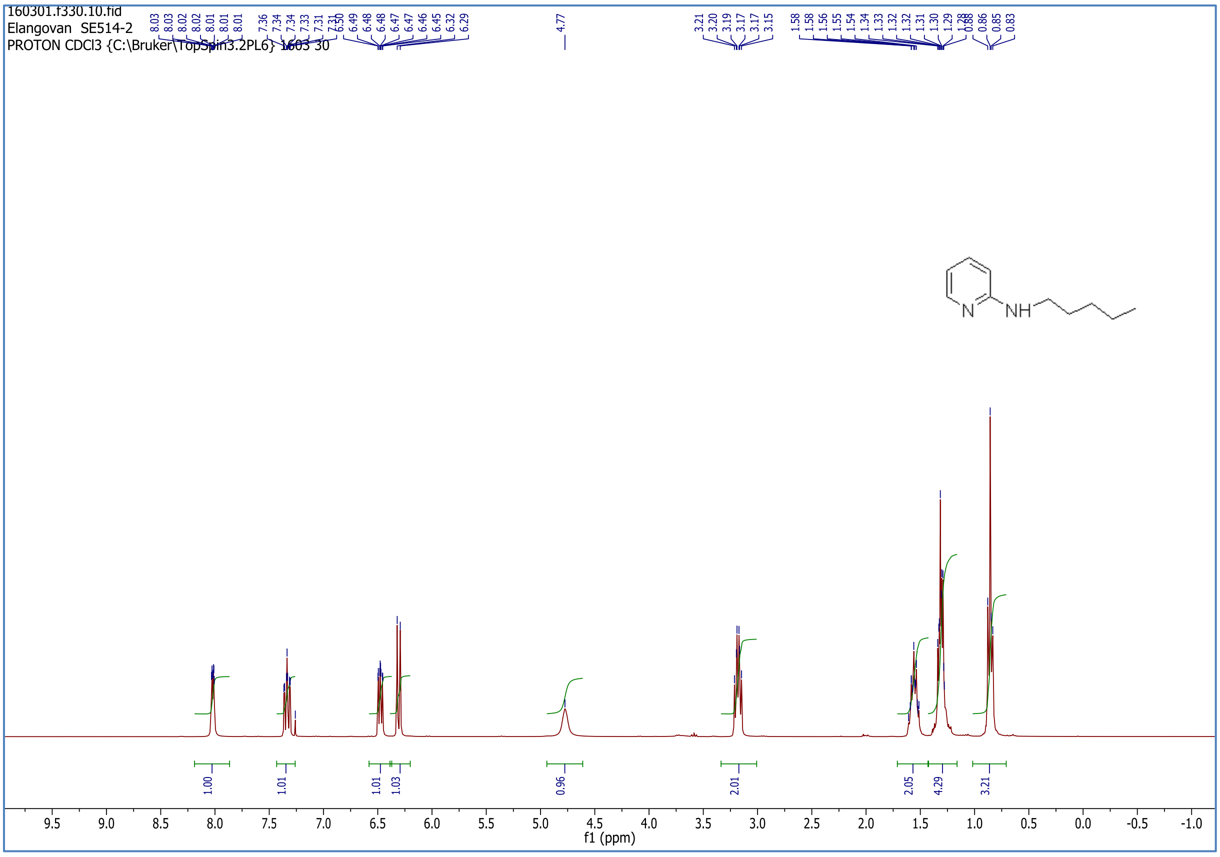

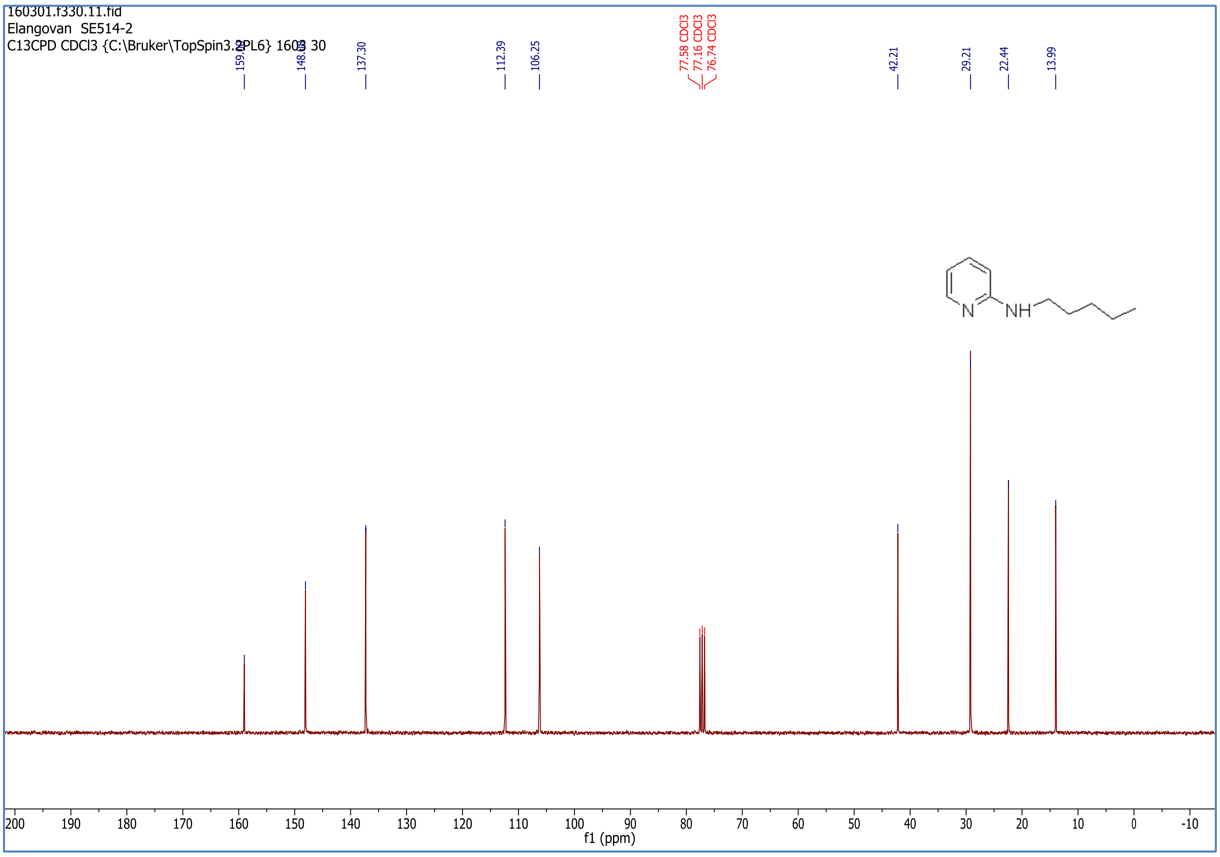

Supplementary Figure 46. ^{1}H and ^{13}C NMR of compound **6q**.

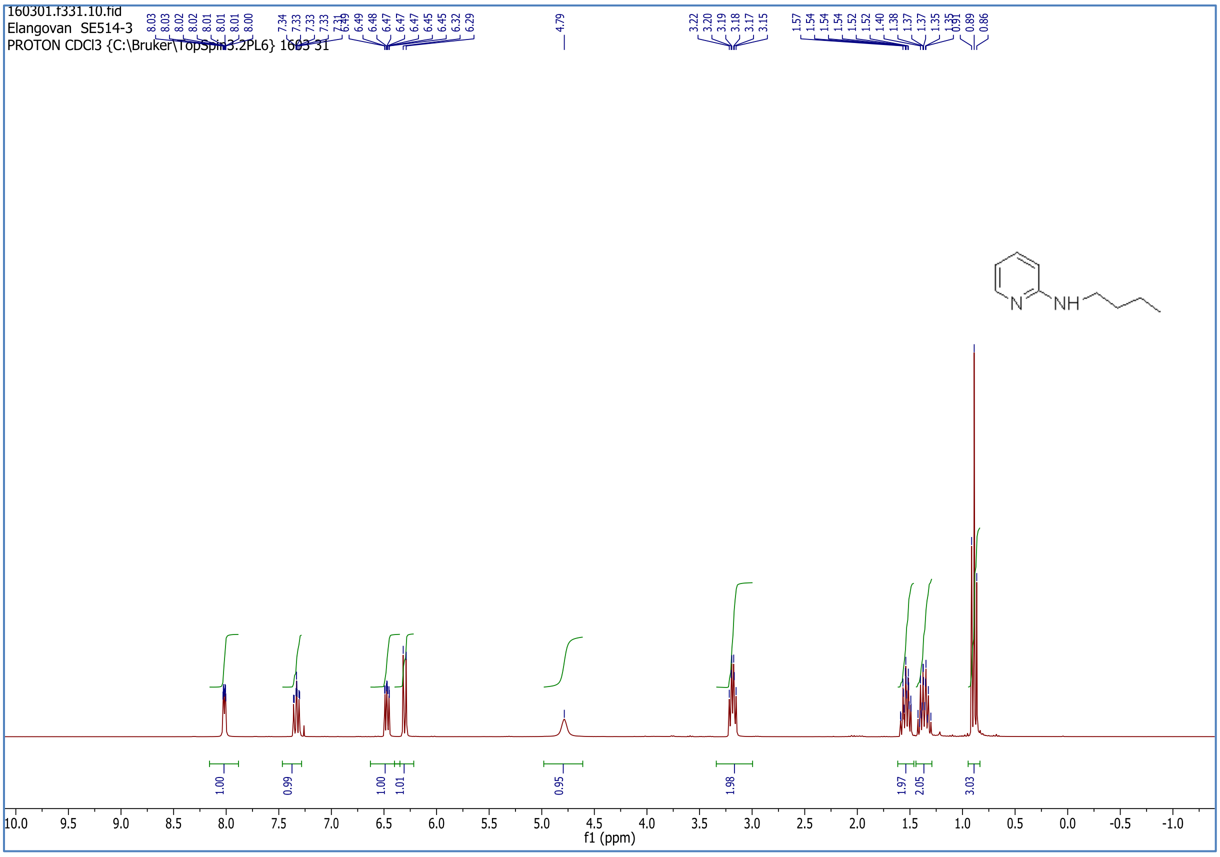

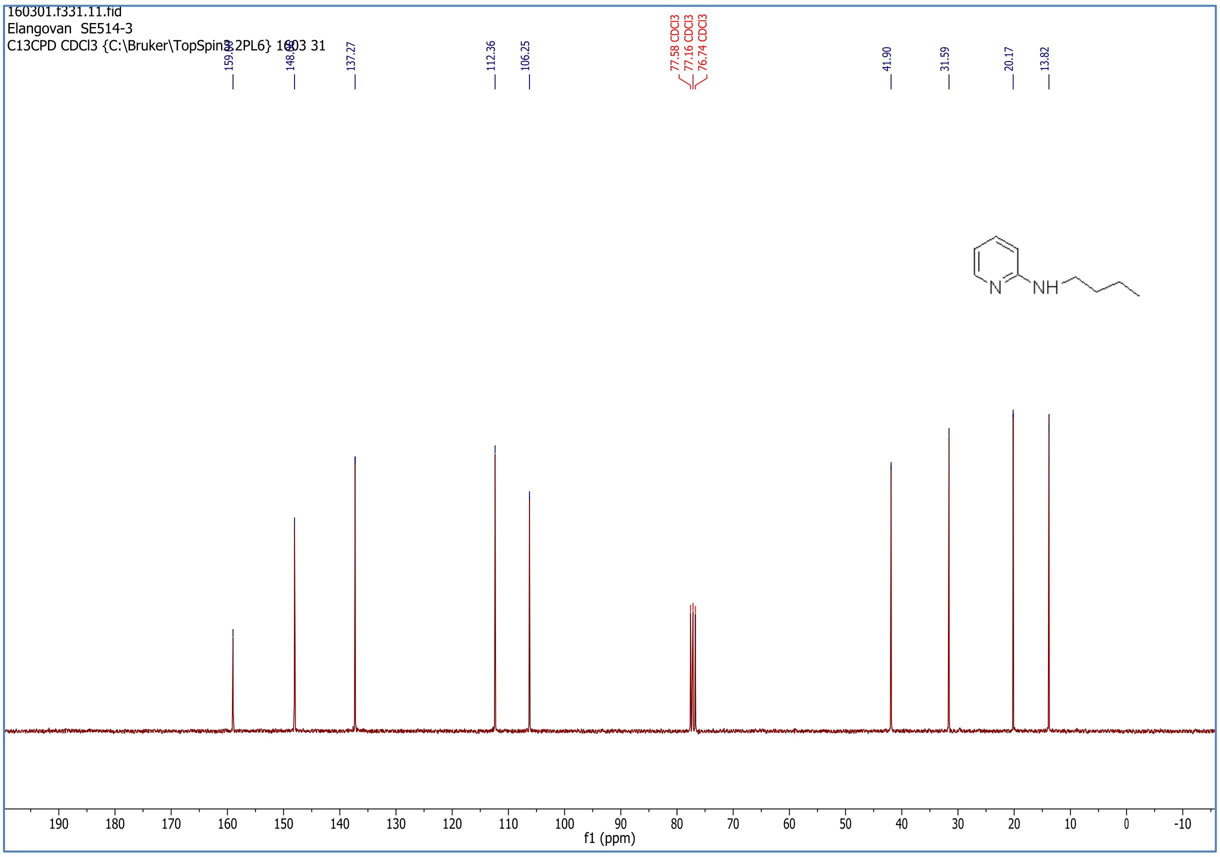

Supplementary Figure 47. ^{1}H and ^{13}C NMR of compound **6r**.

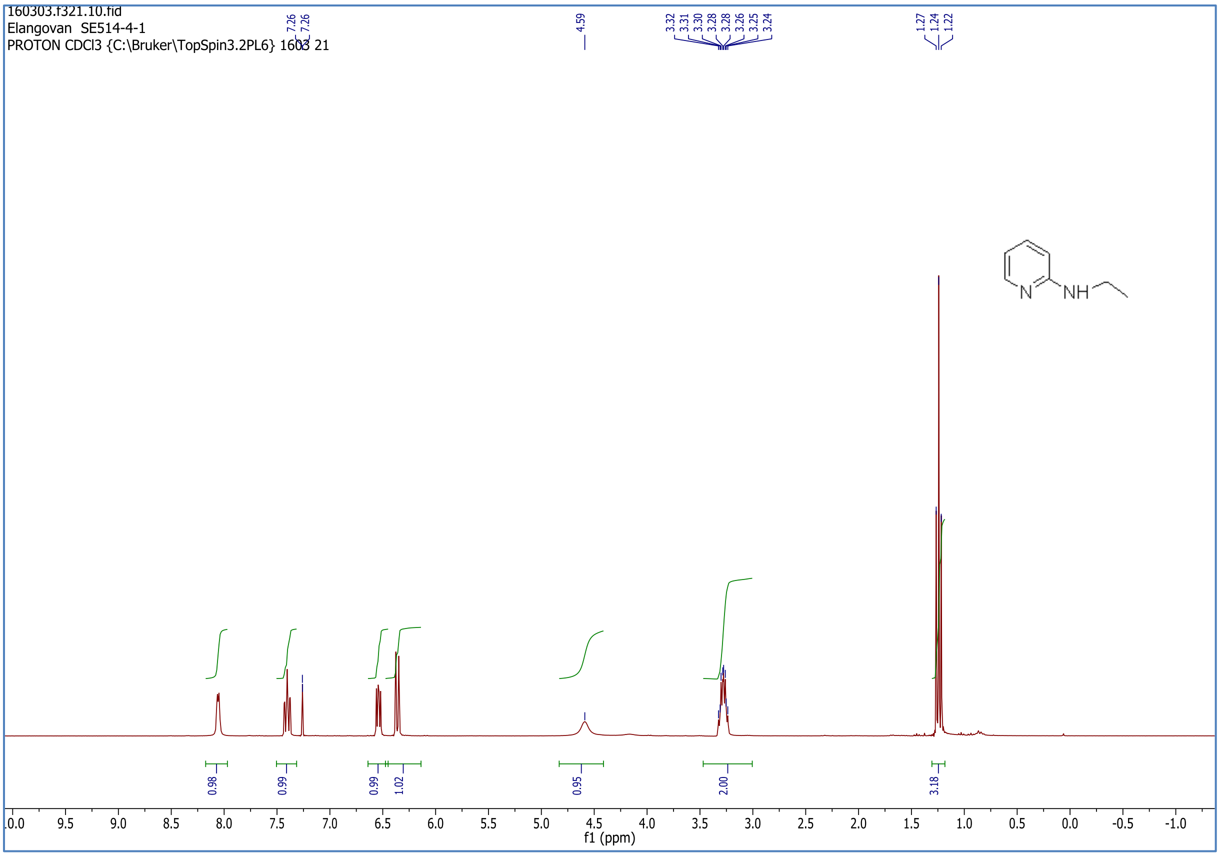

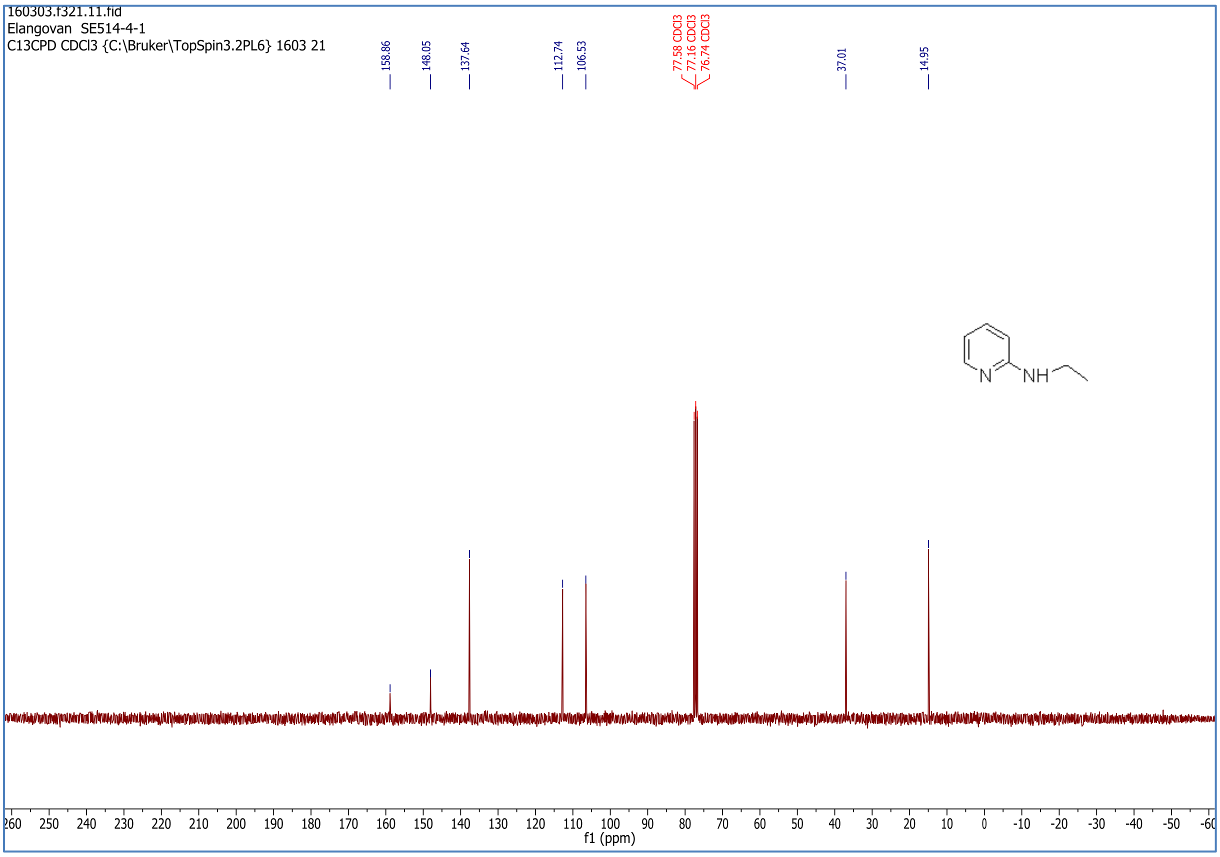

Supplementary Figure 48. ^{1}H and ^{13}C NMR of compound **6s**.

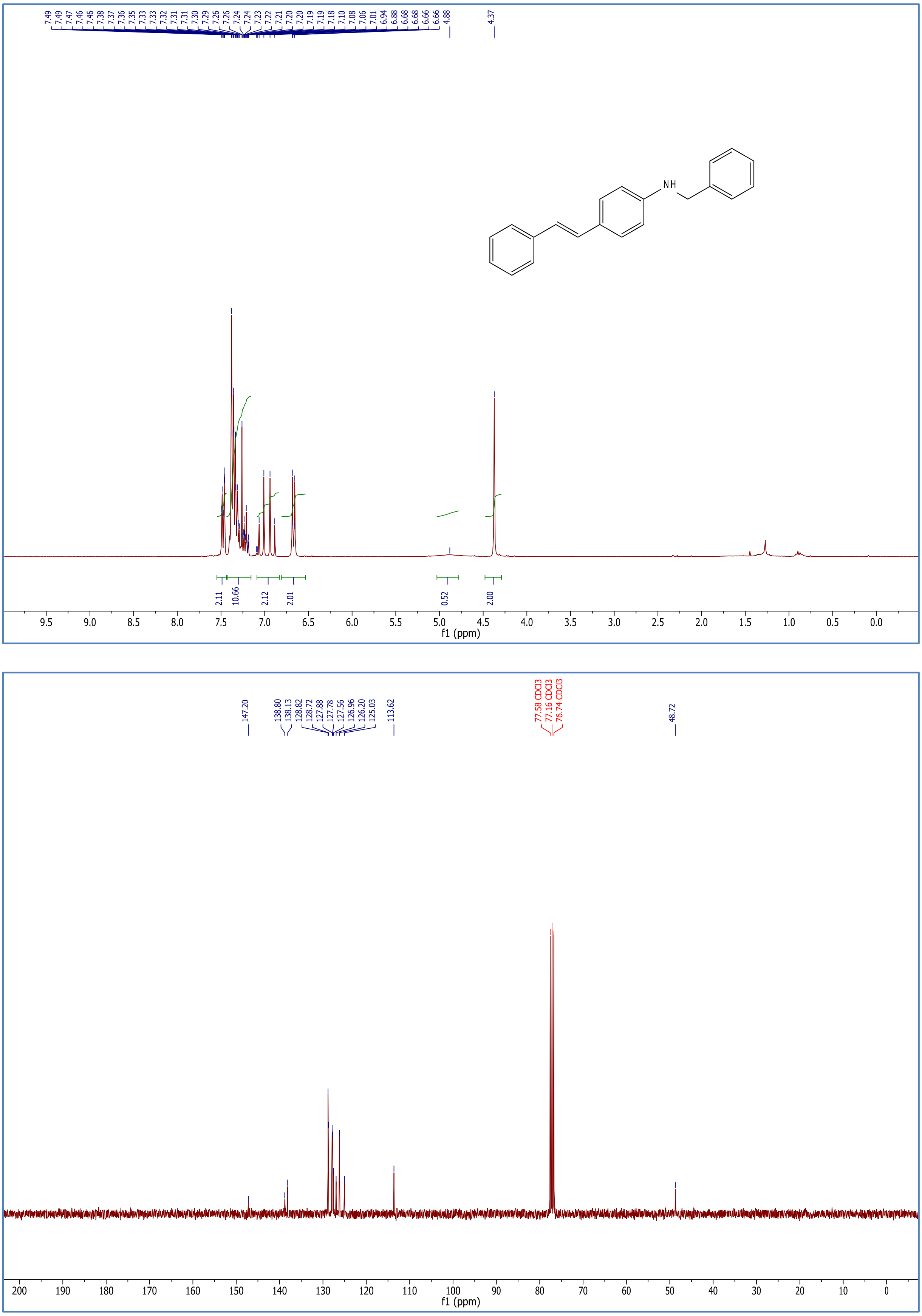

Supplementary Figure 49. ^{1}H and ^{13}C NMR of compound **7a**.

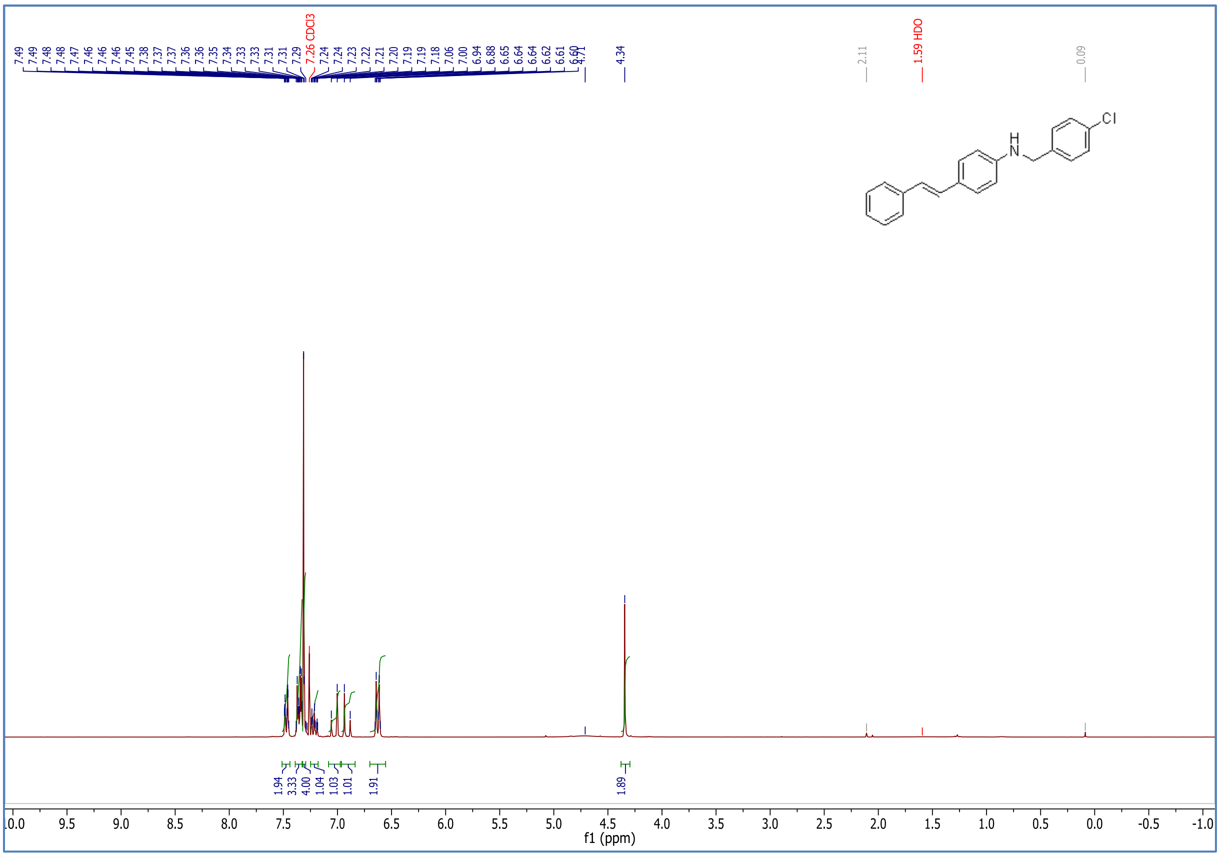

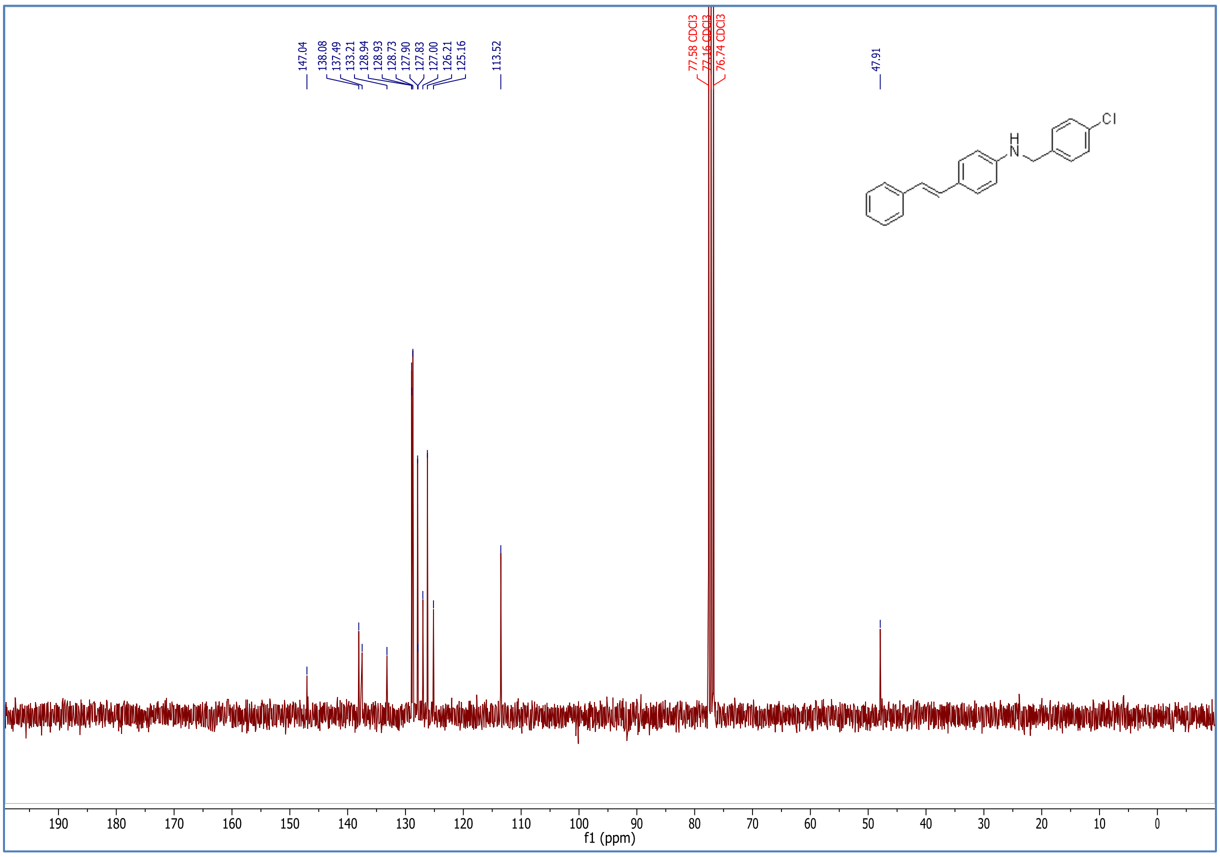

Supplementary Figure 50. ^{1}H and ^{13}C NMR of compound **7b**.

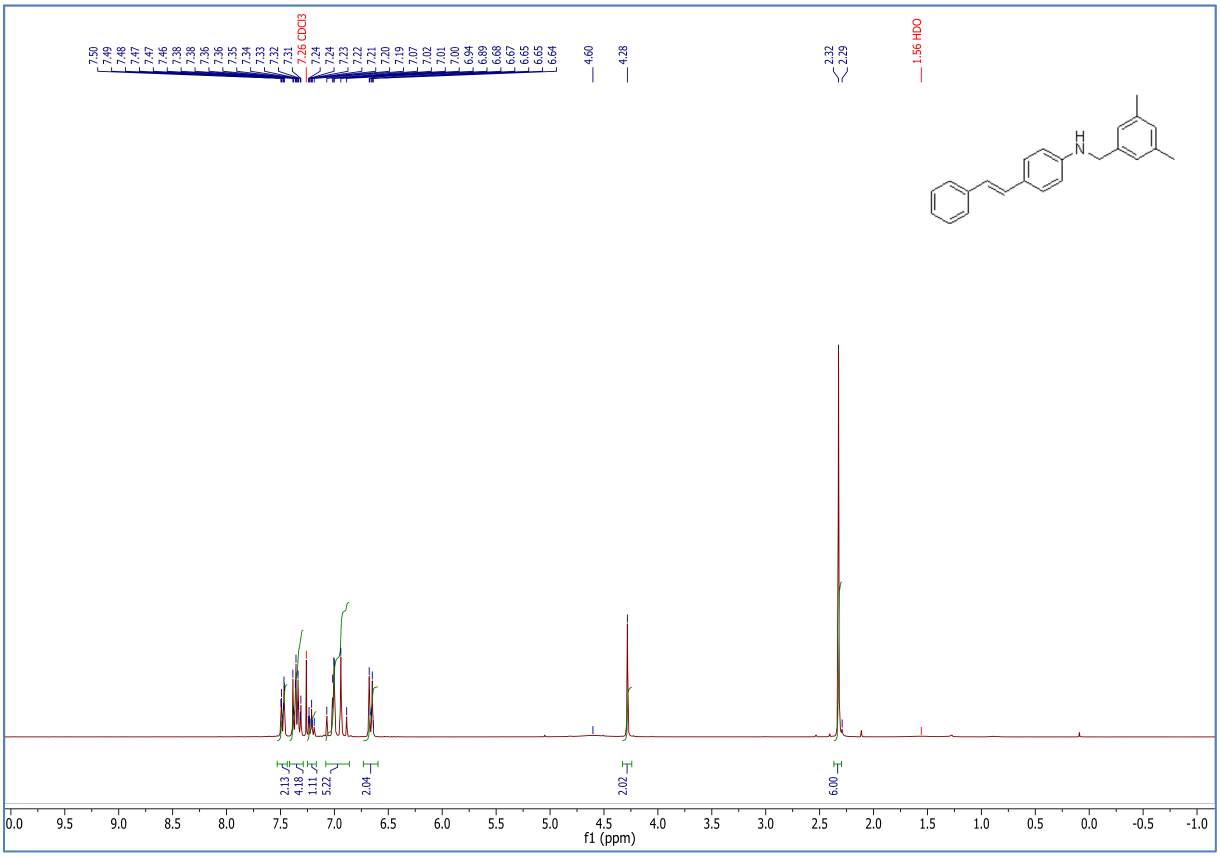

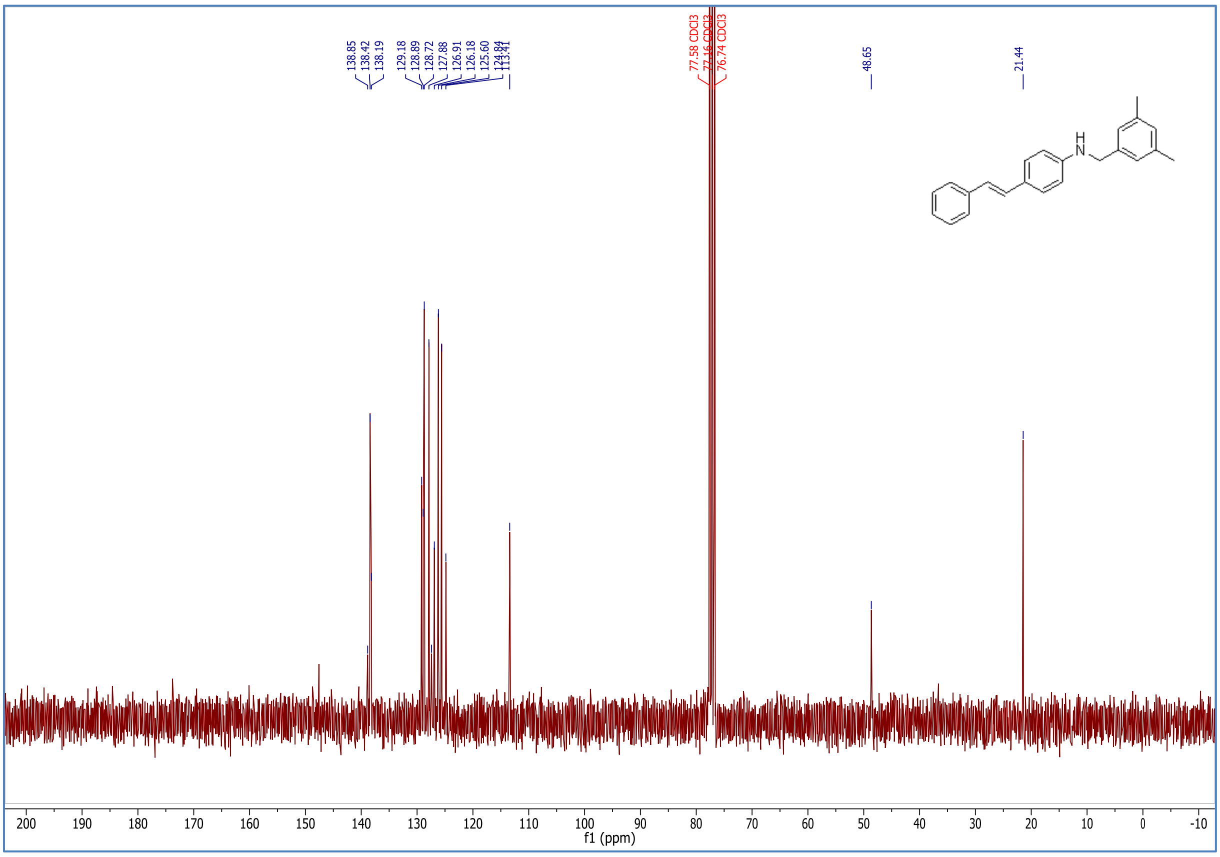

Supplementary Figure 51. ^{1}H and ^{13}C NMR of compound **7c**.

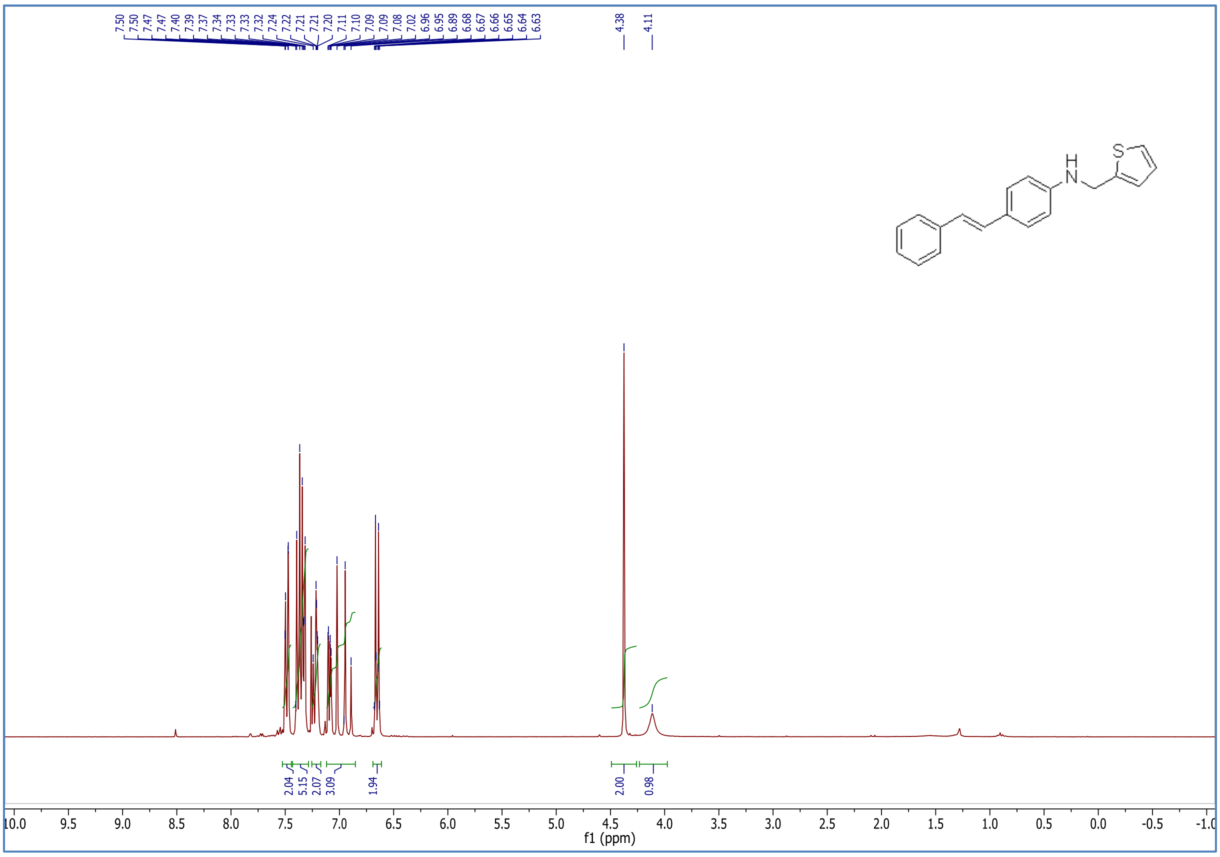

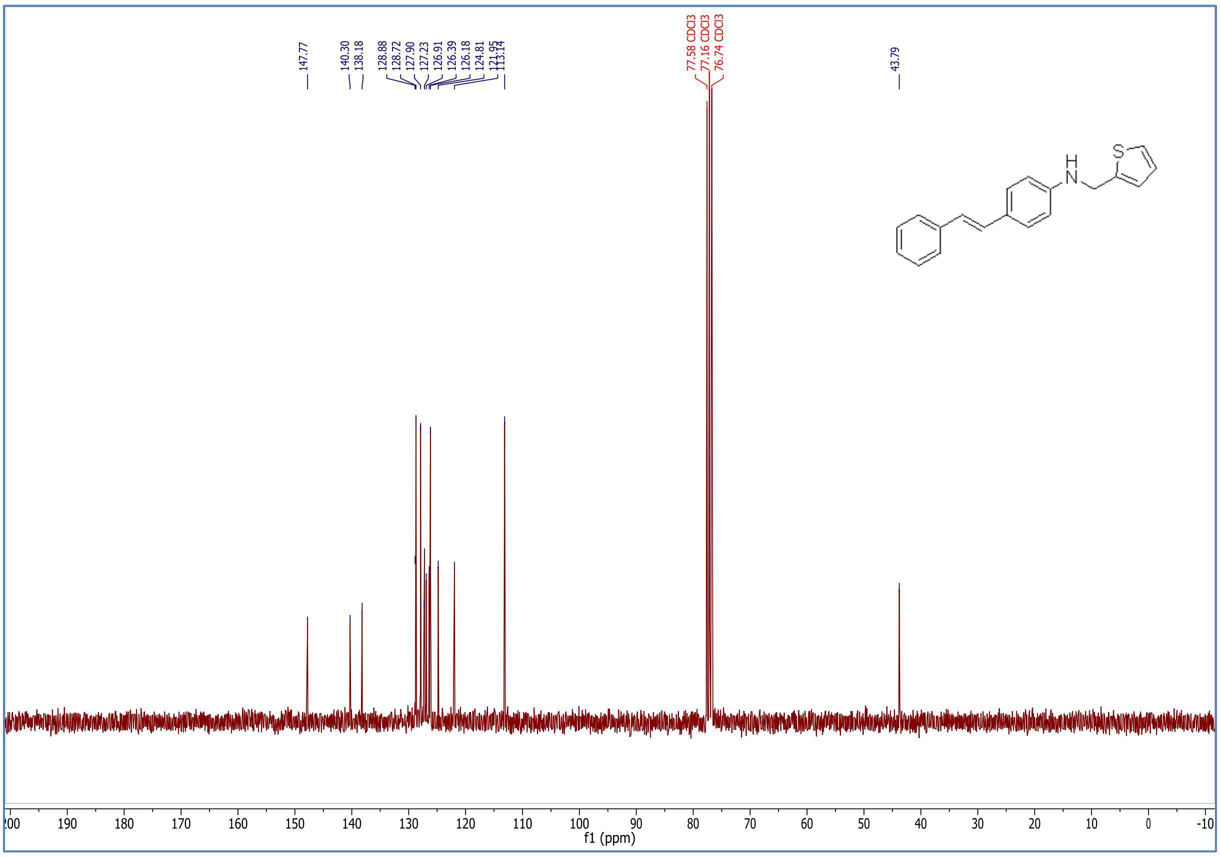

Supplementary Figure 52. ^{1}H and ^{13}C NMR of compound **7d**.

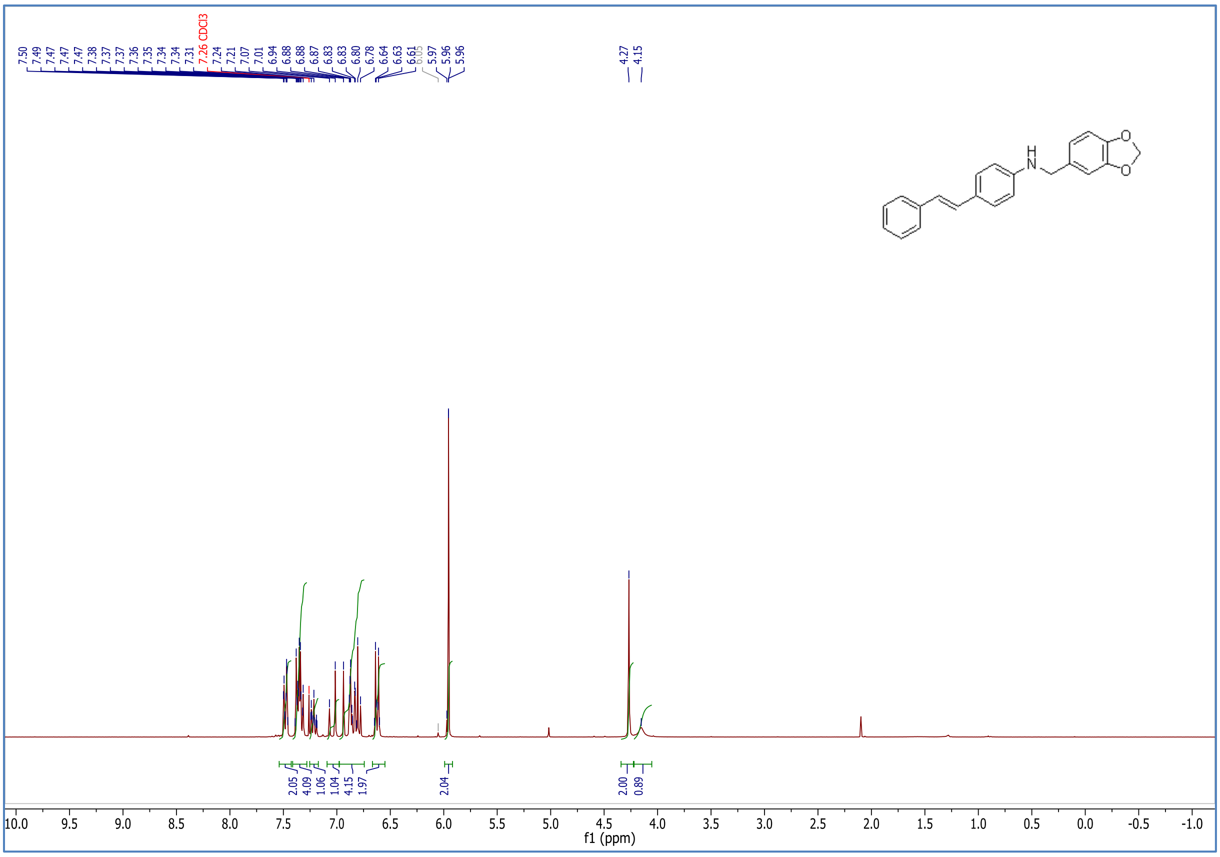

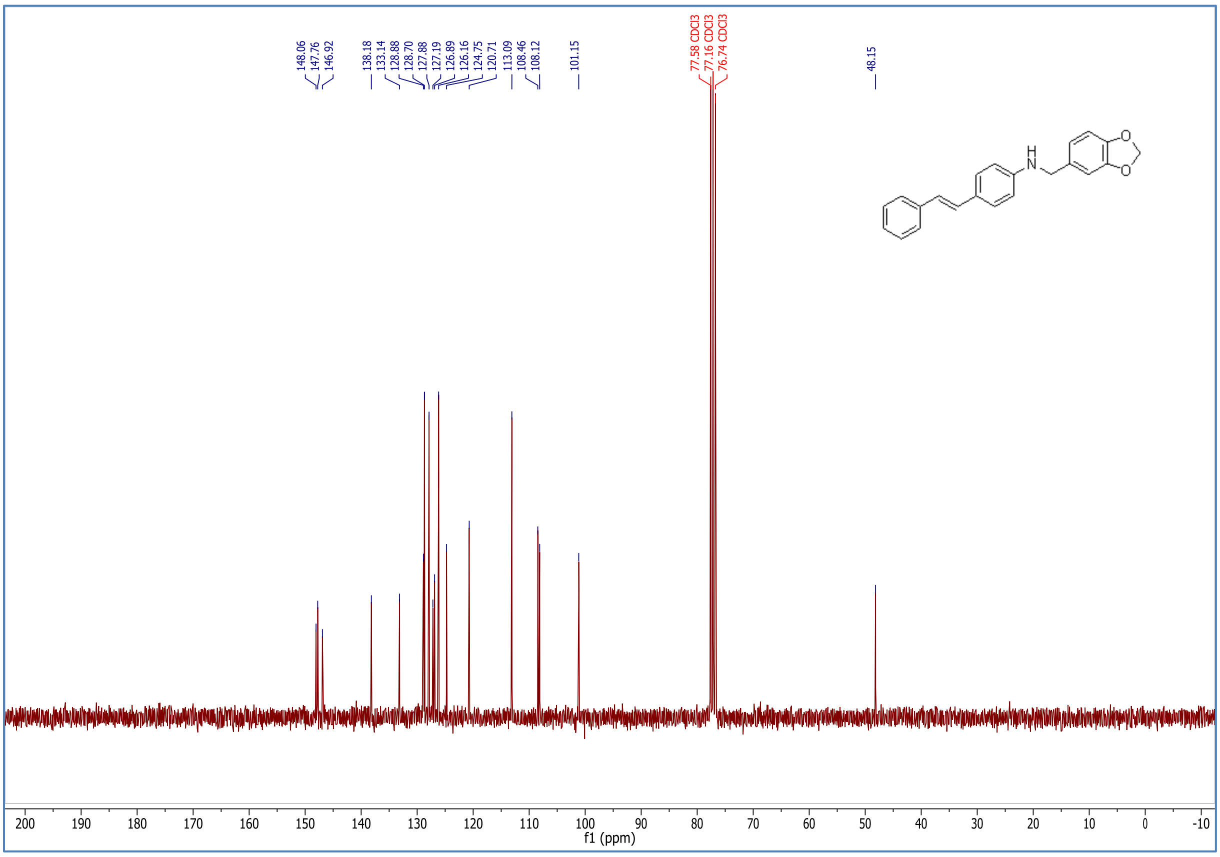

Supplementary Figure 53. ^{1}H and ^{13}C NMR of compound **7e**.

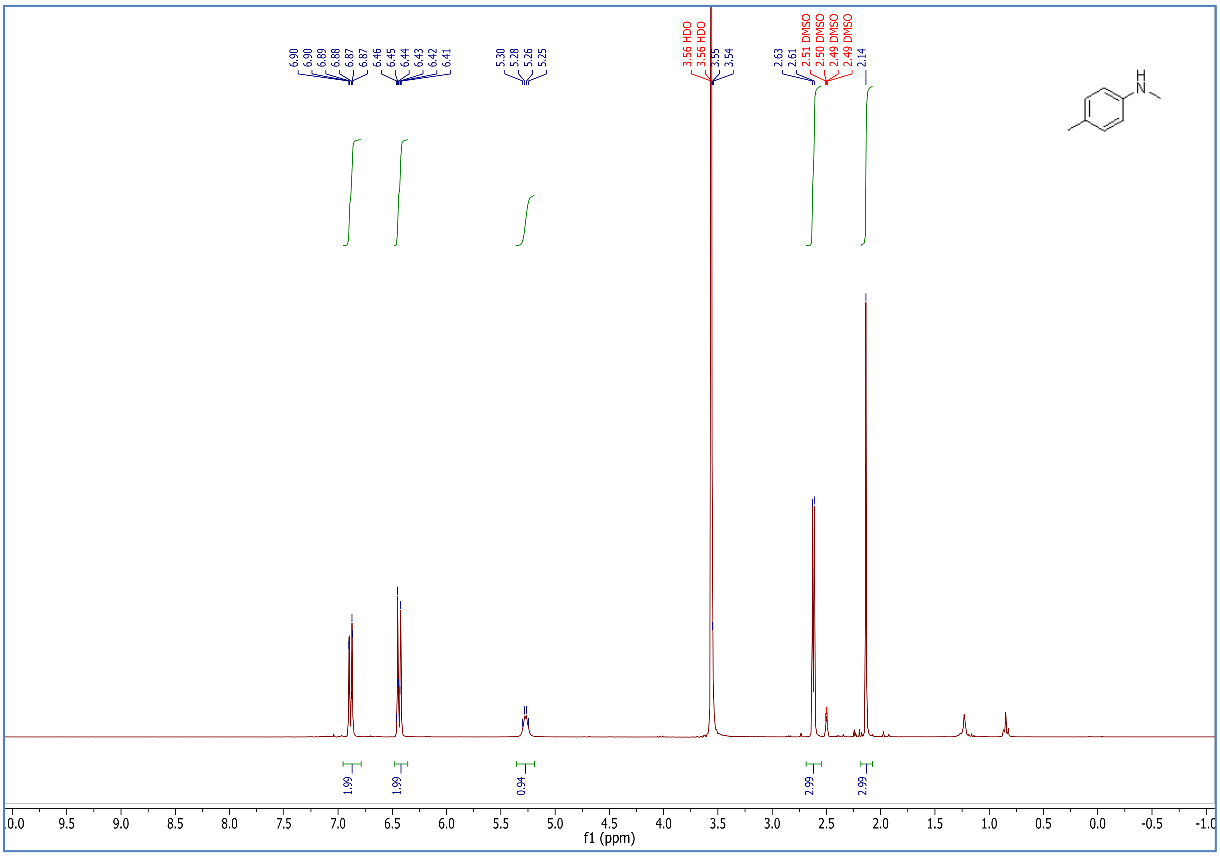

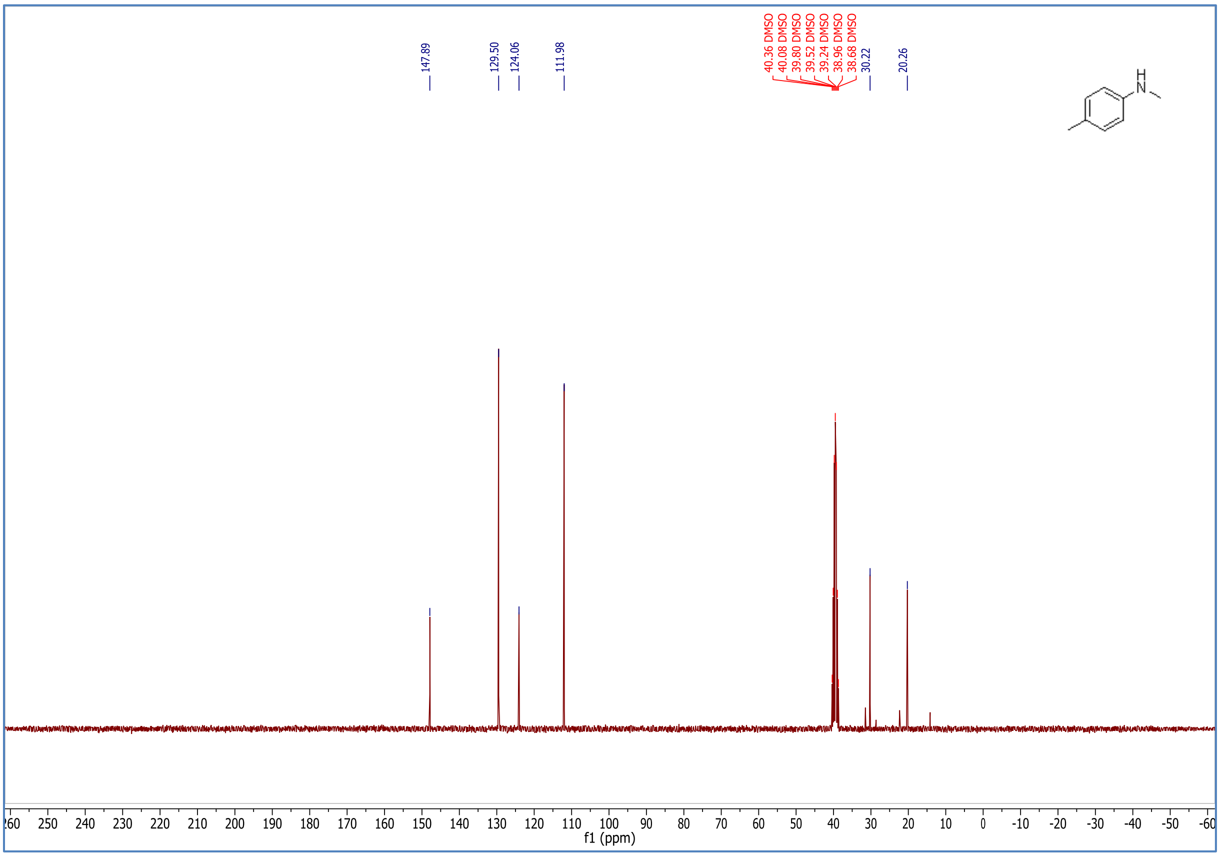

Supplementary Figure 54. ^{1}H and ^{13}C NMR of compound **9a**.

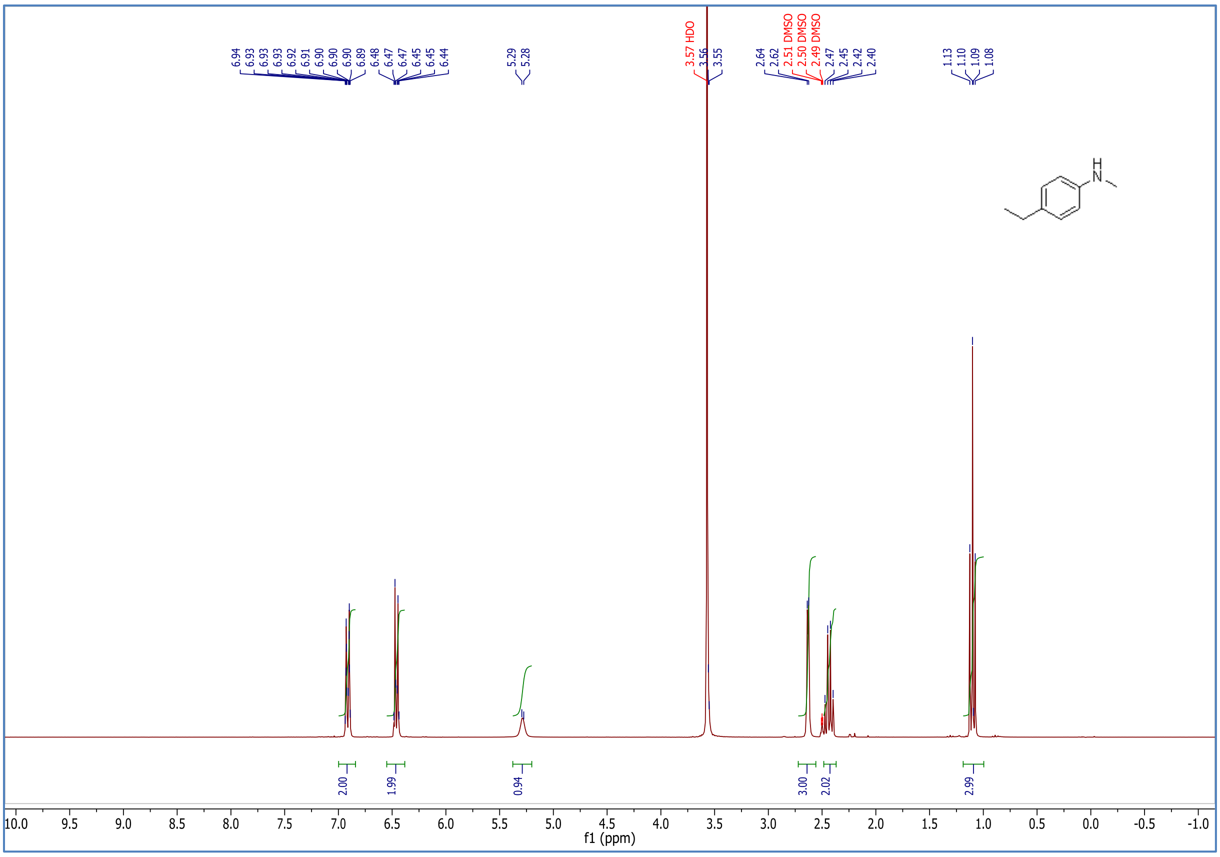

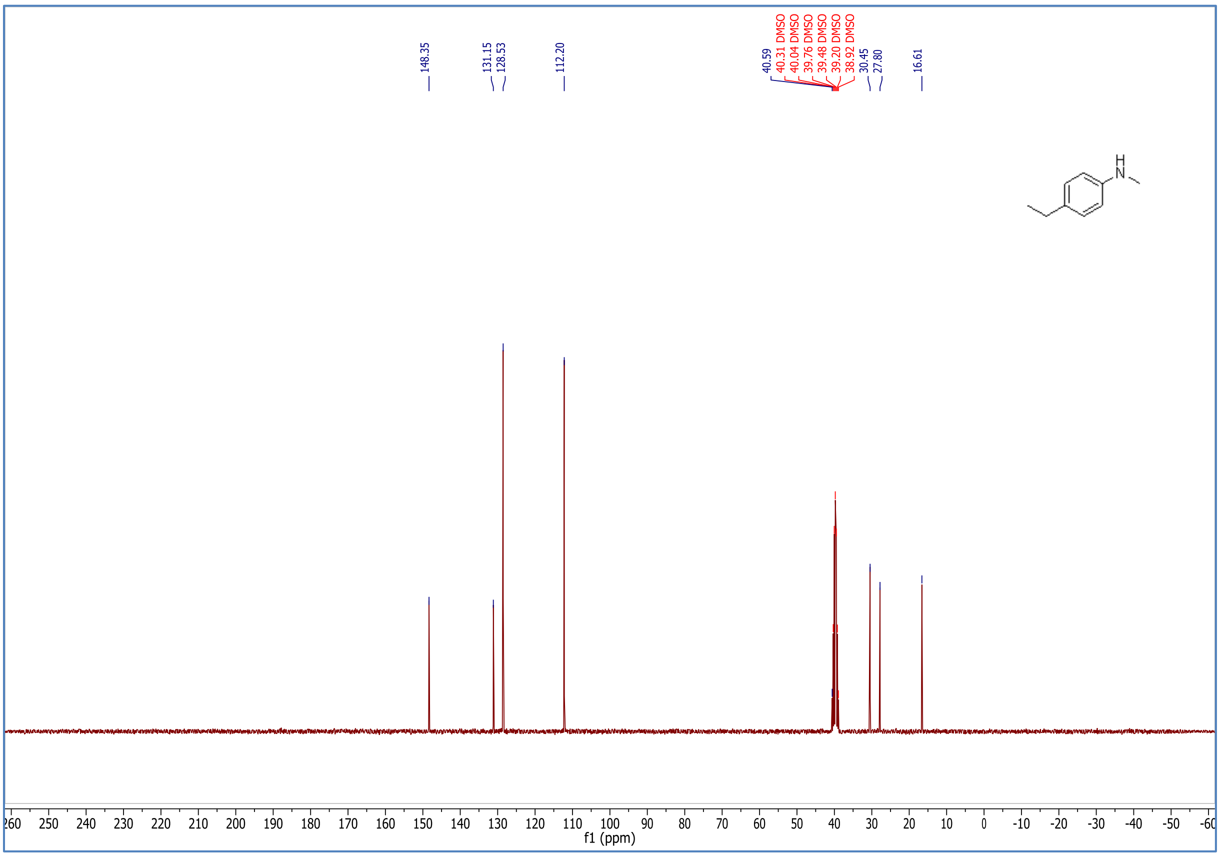

Supplementary Figure 55. ^{1}H and ^{13}C NMR of compound **9b**.

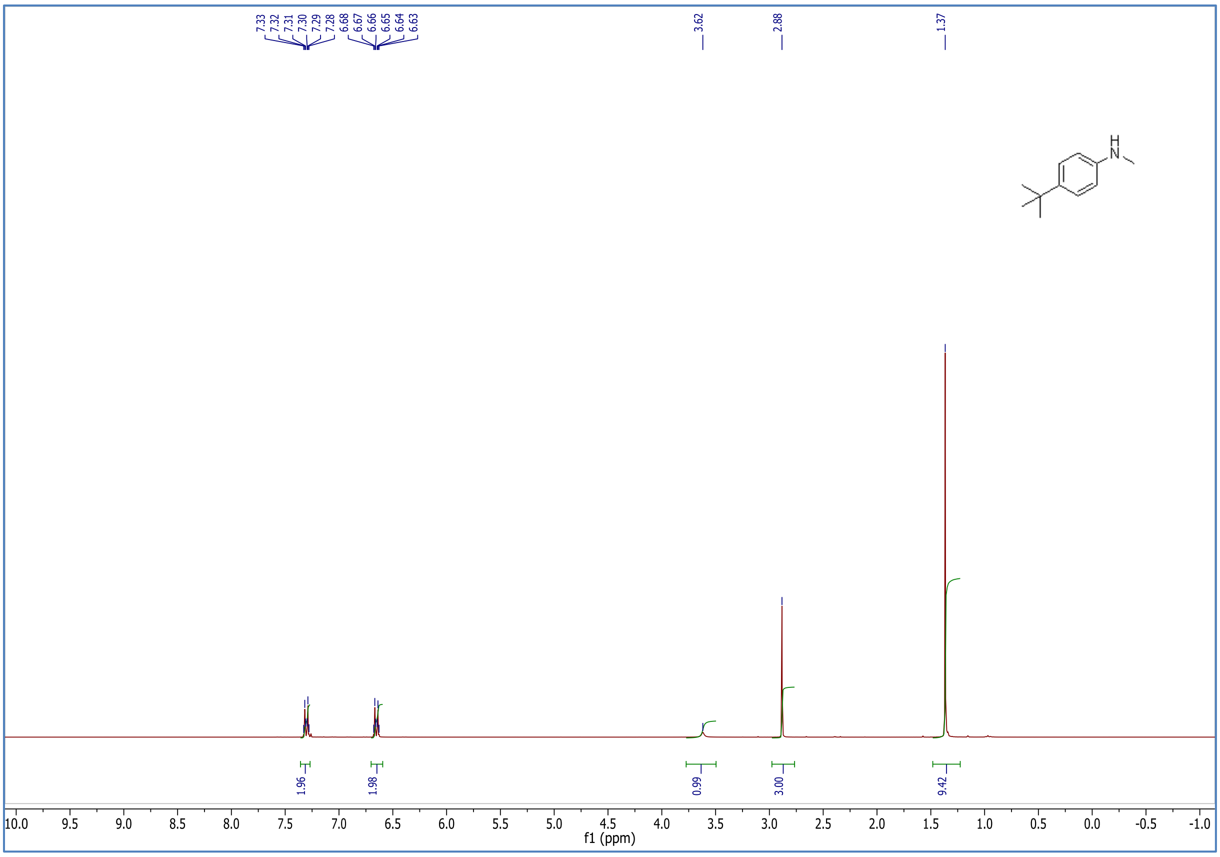

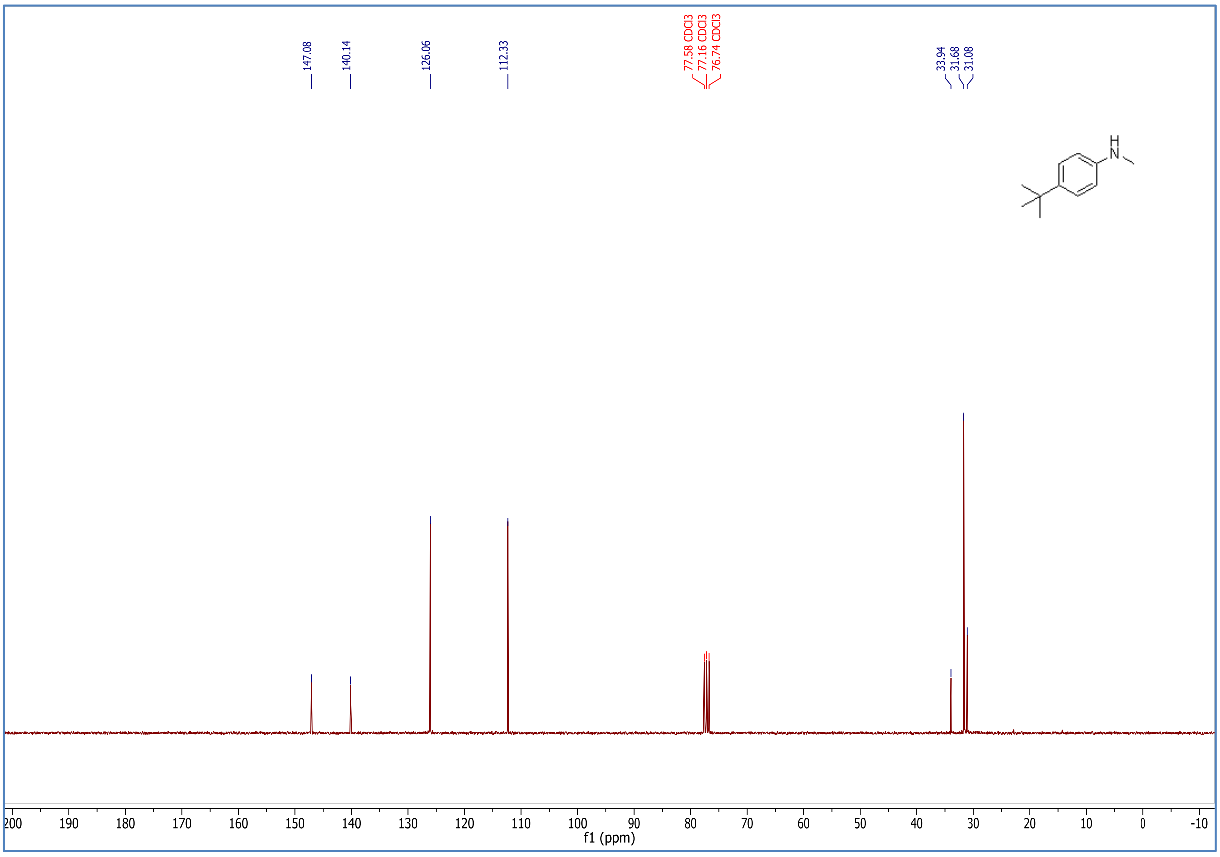

Supplementary Figure 56. ^{1}H and ^{13}C NMR of compound **9e**.

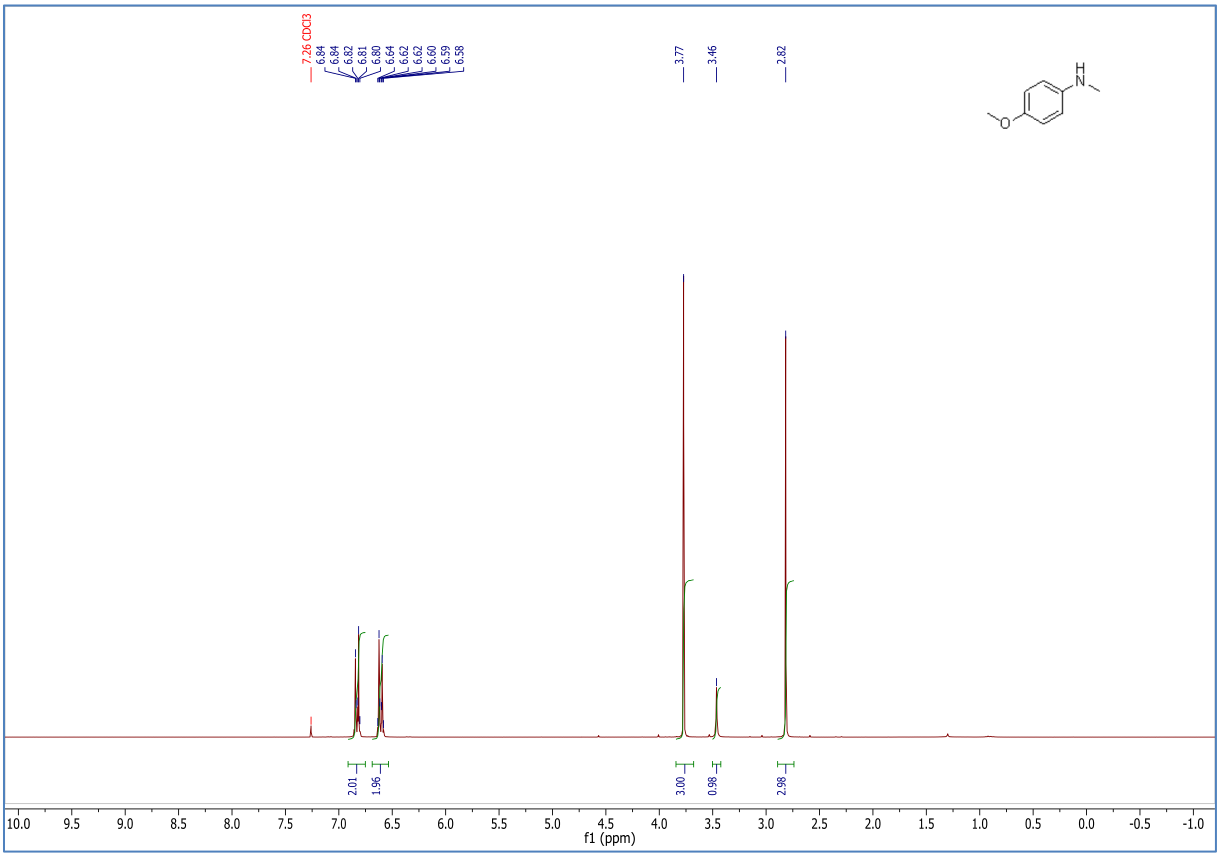

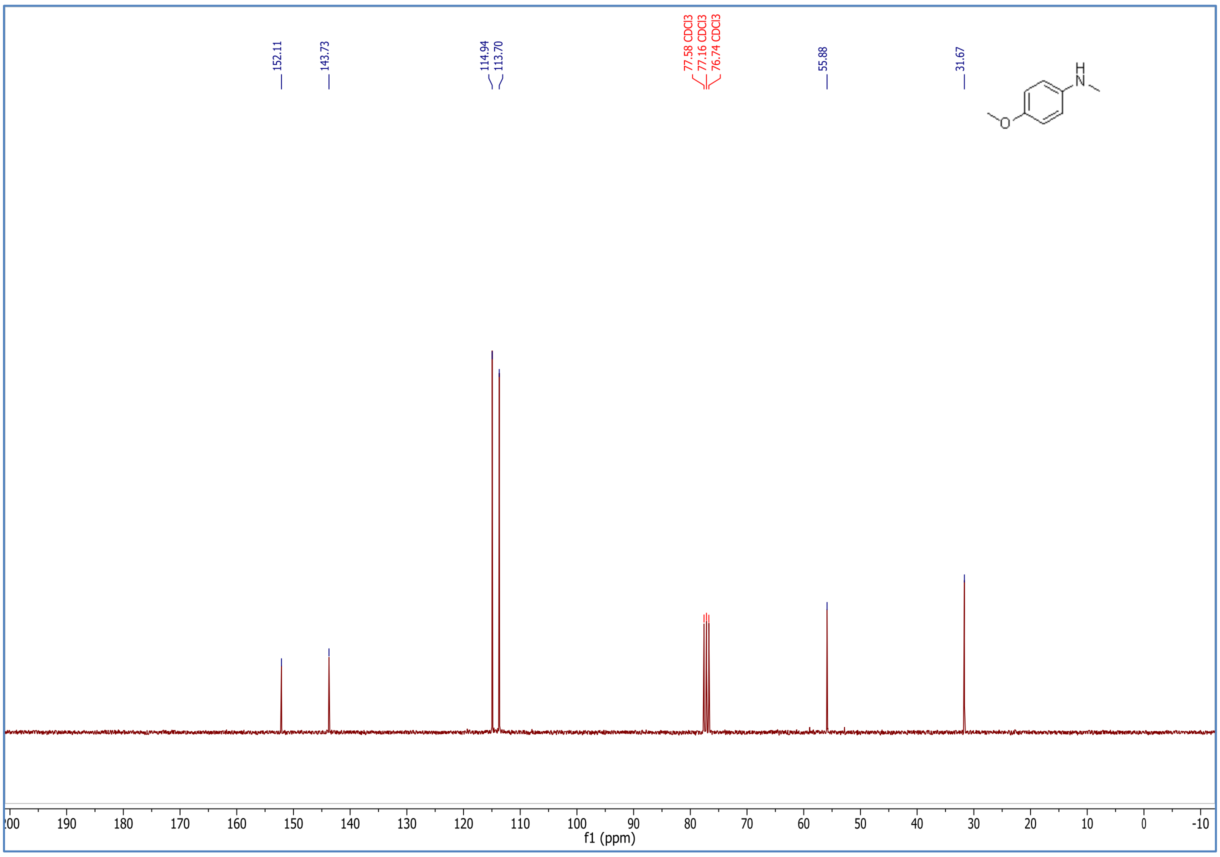

Supplementary Figure 57. ^{1}H and ^{13}C NMR of compound **9c**.

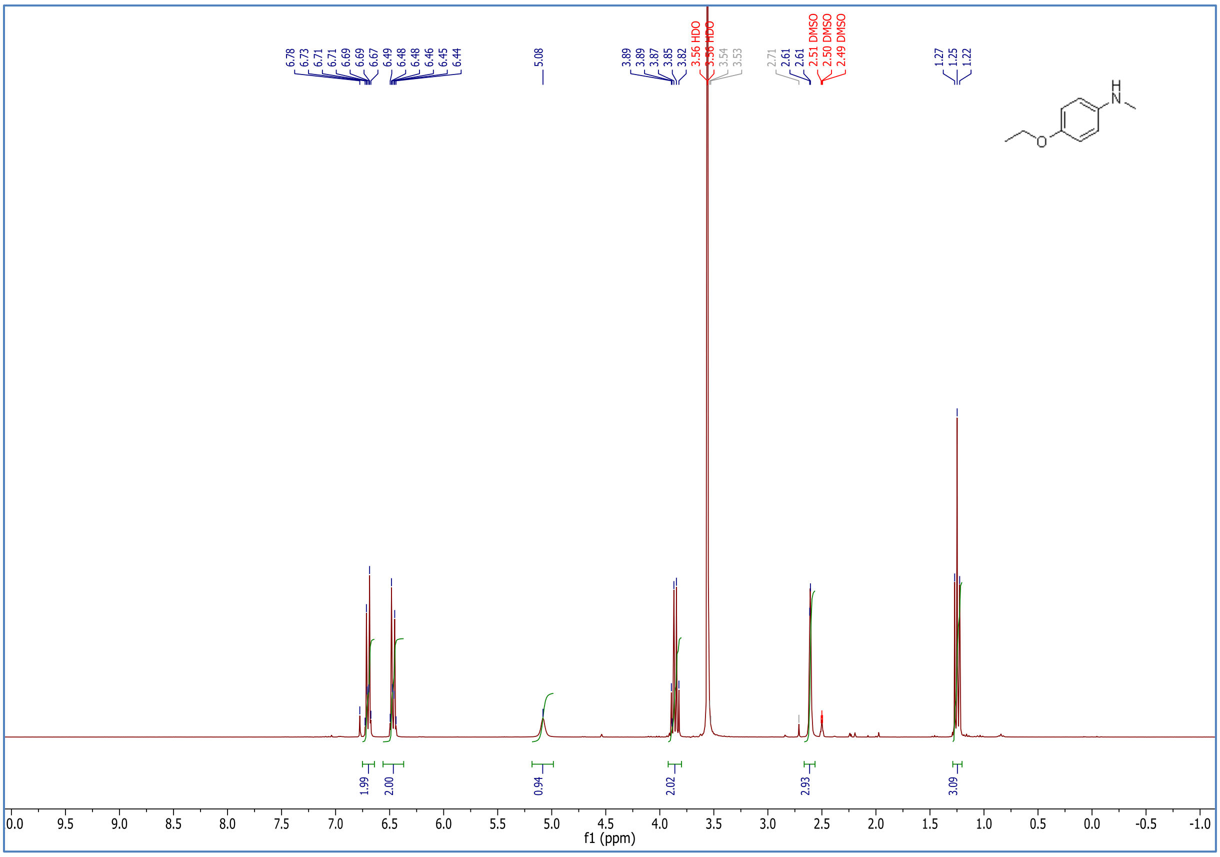

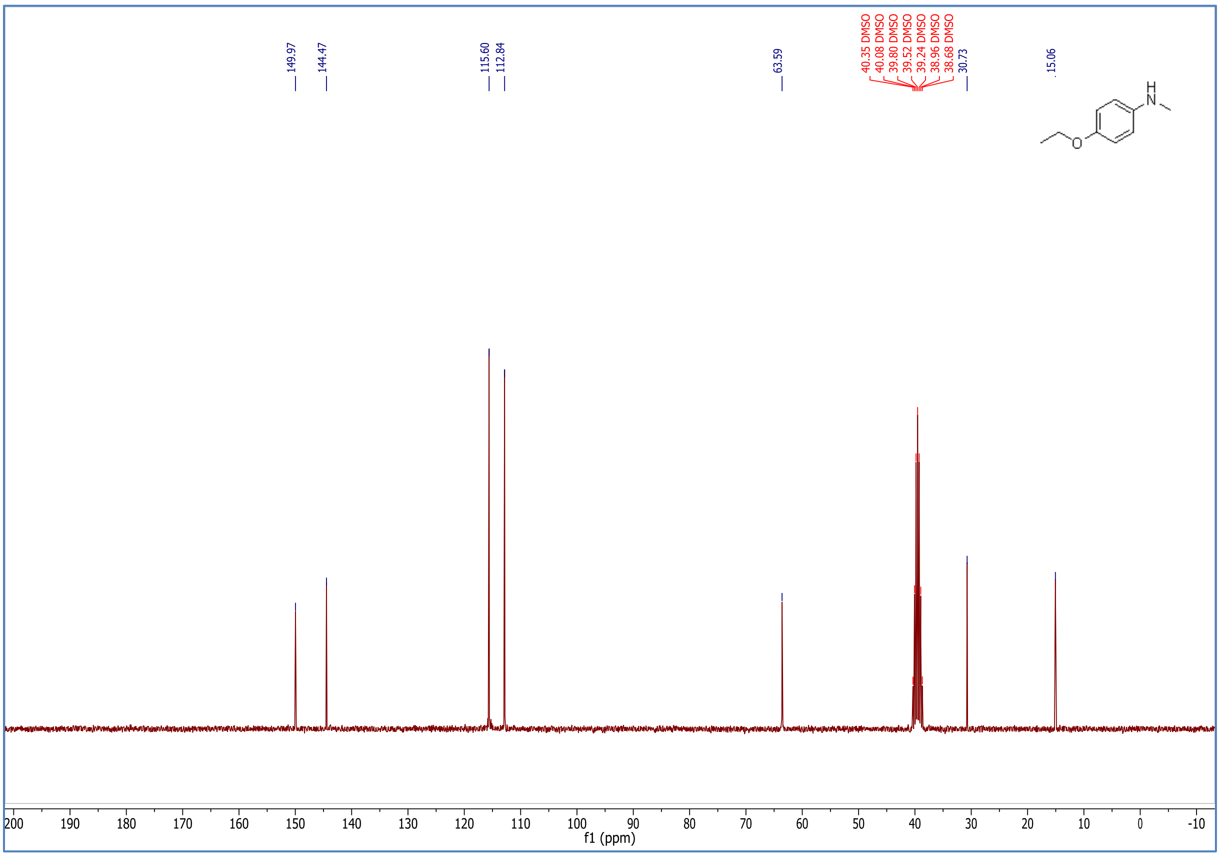

Supplementary Figure 58. ^{1}H and ^{13}C NMR of compound **9d**.

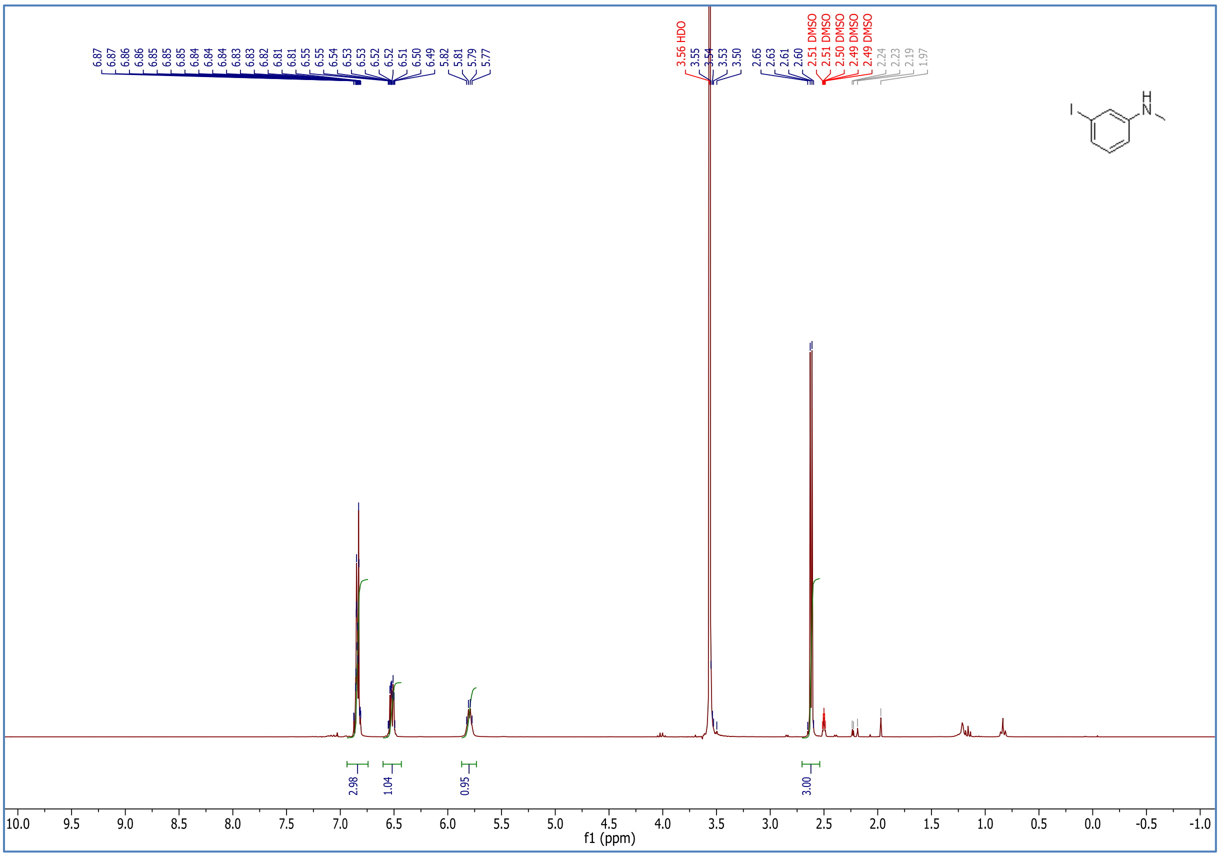

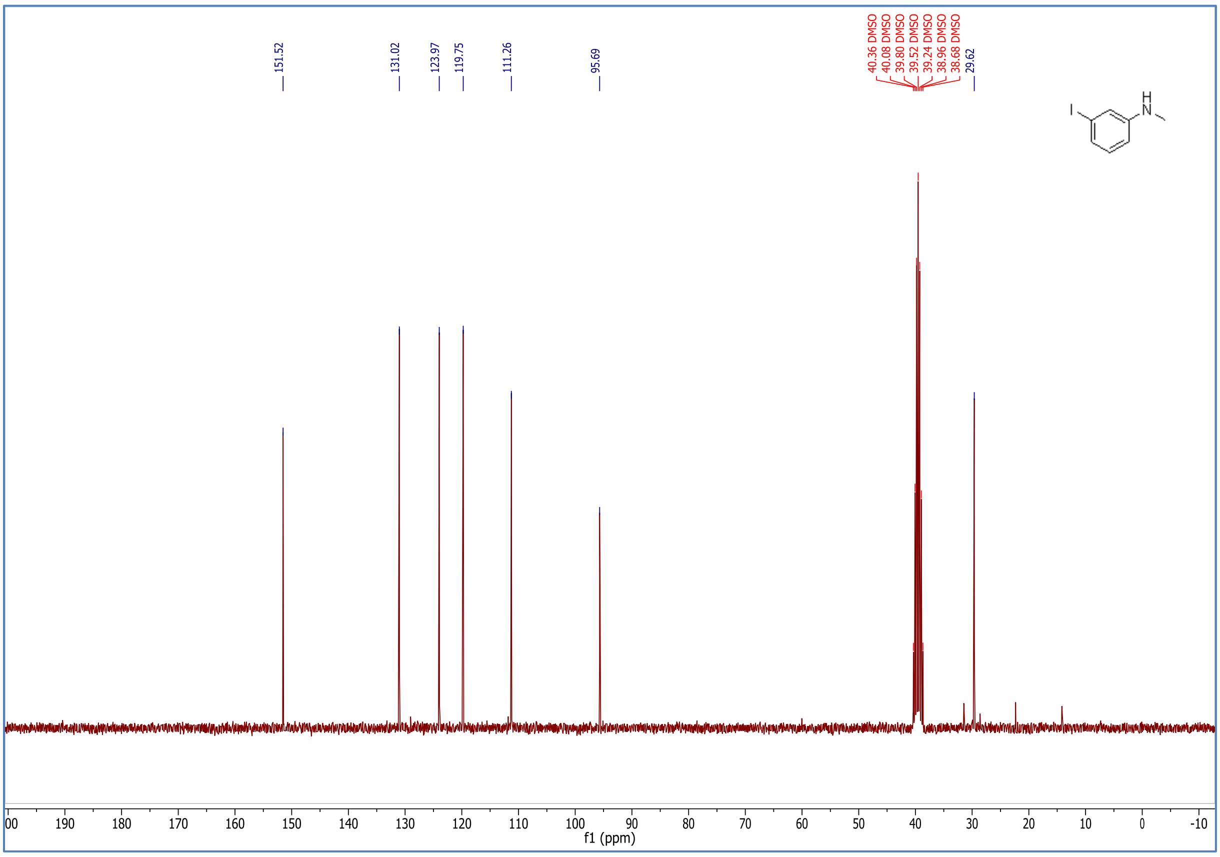

Supplementary Figure 59. ^{1}H and ^{13}C NMR of compound **9i**.

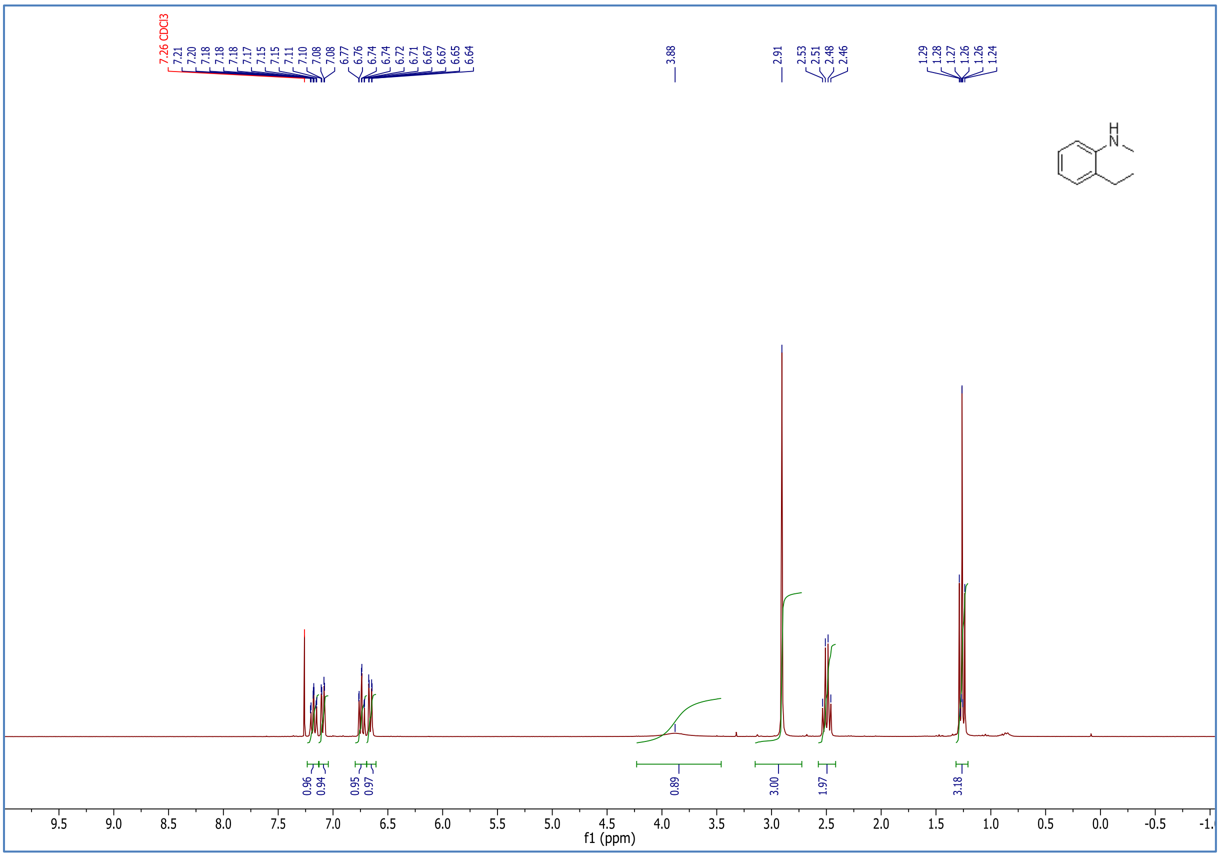

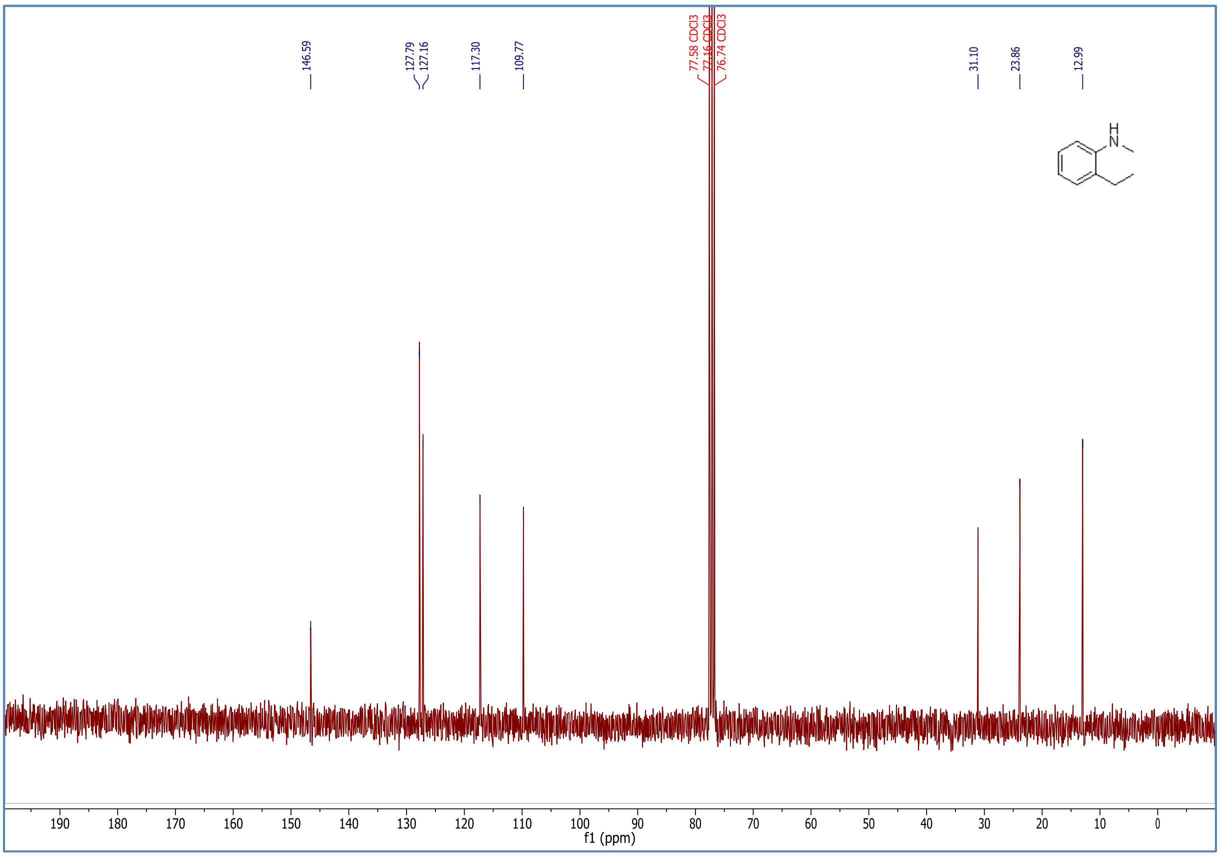

Supplementary Figure 60. ^{1}H and ^{13}C NMR of compound **9k**.

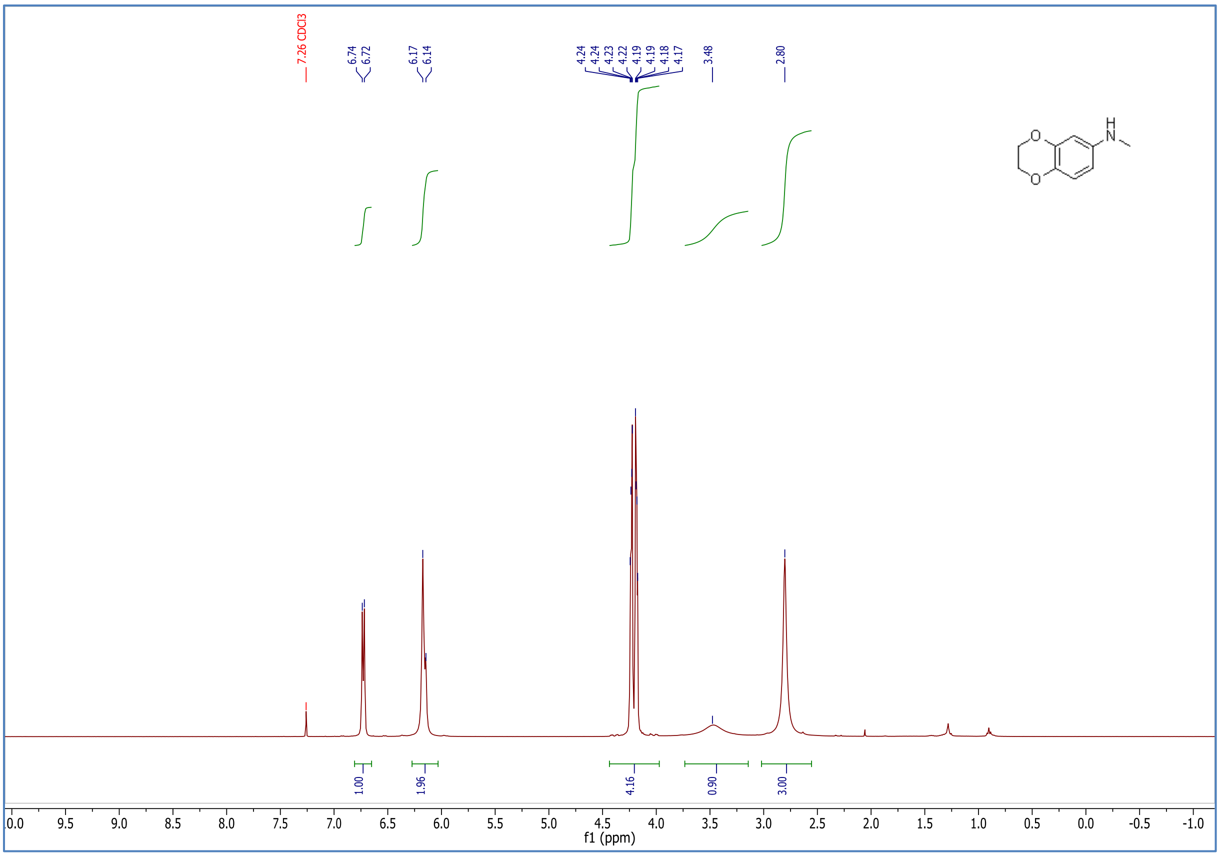

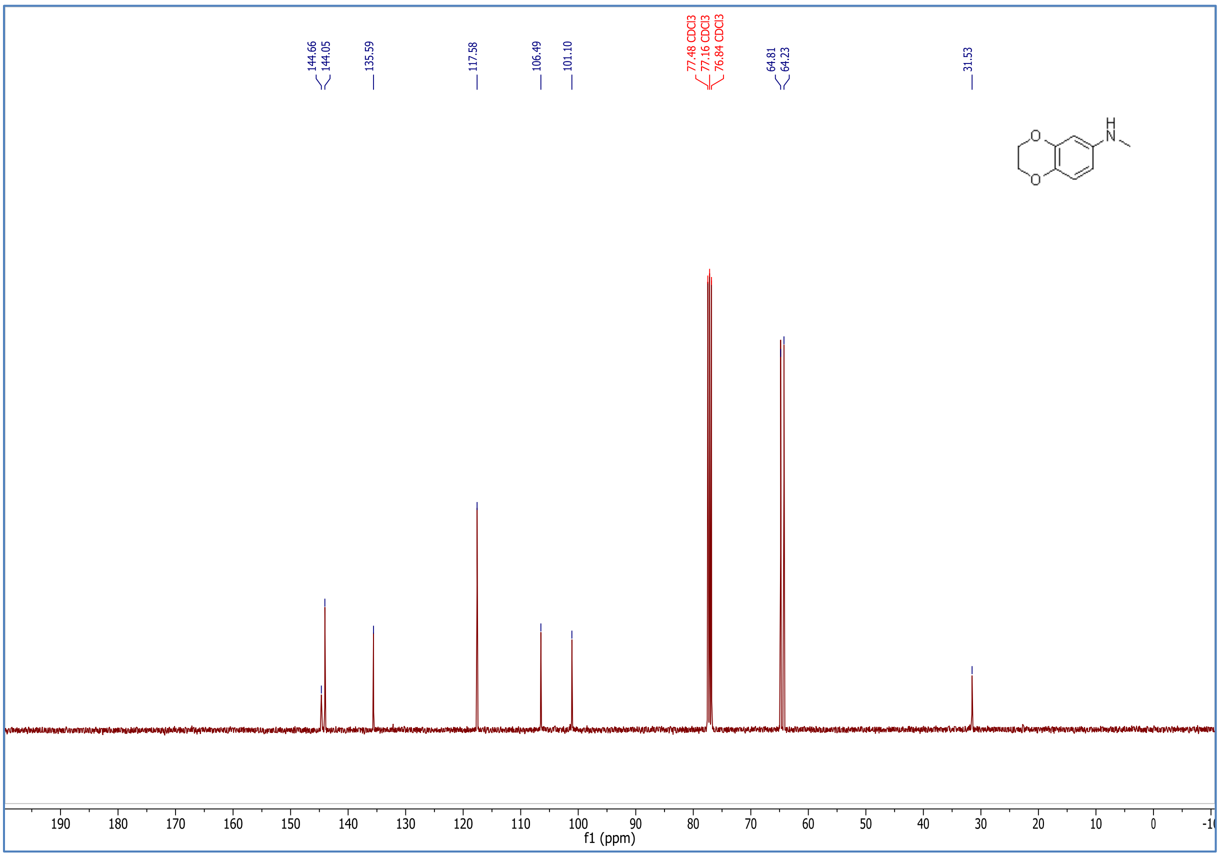

Supplementary Figure 61. ^{1}H and ^{13}C NMR of compound **9l**.

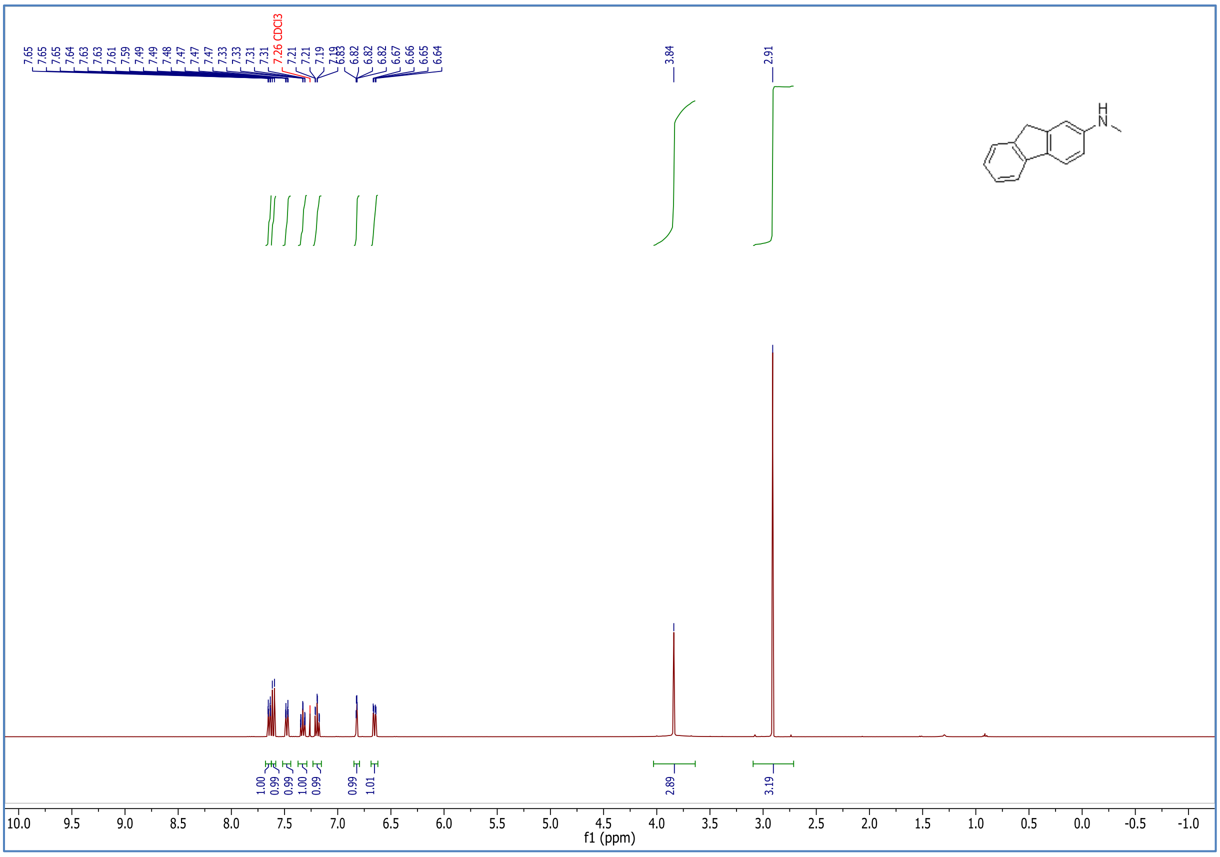

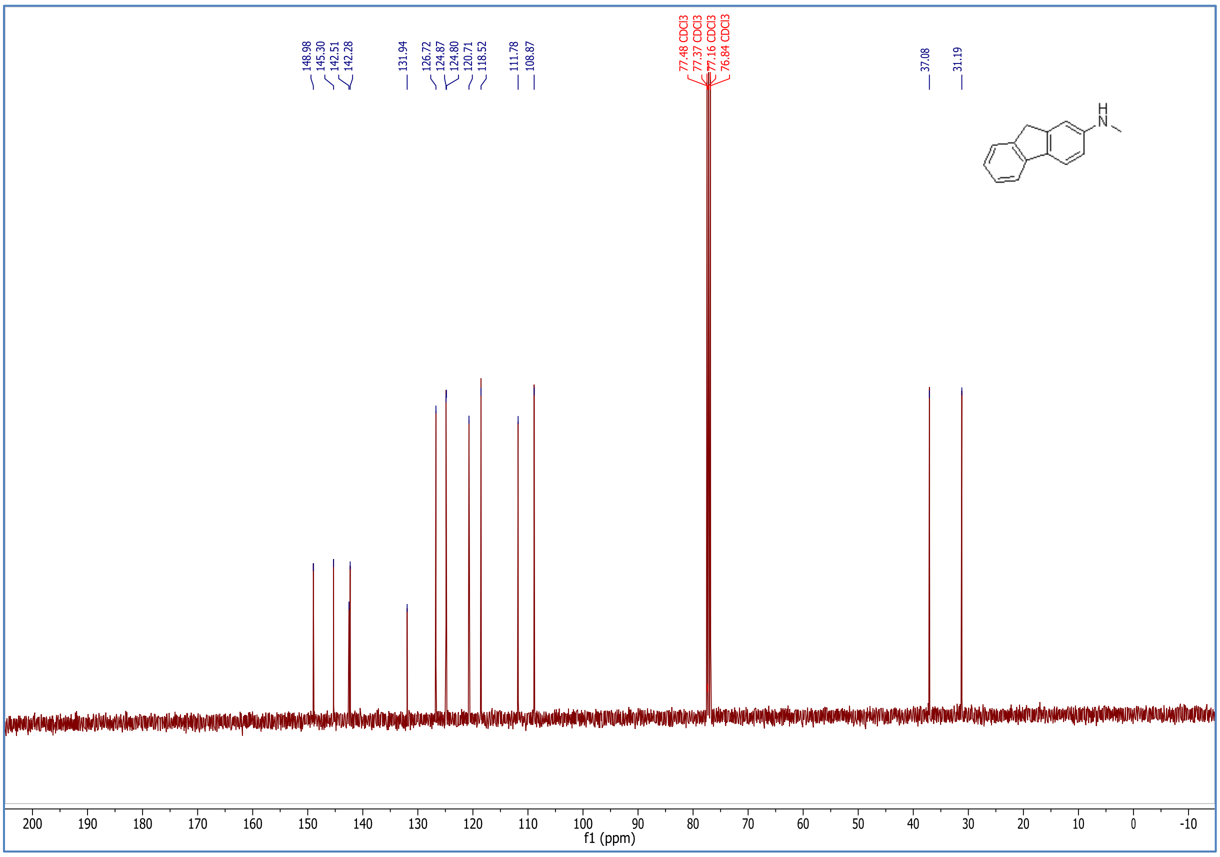

Supplementary Figure 62. ^{1}H and ^{13}C NMR of compound **9n**.

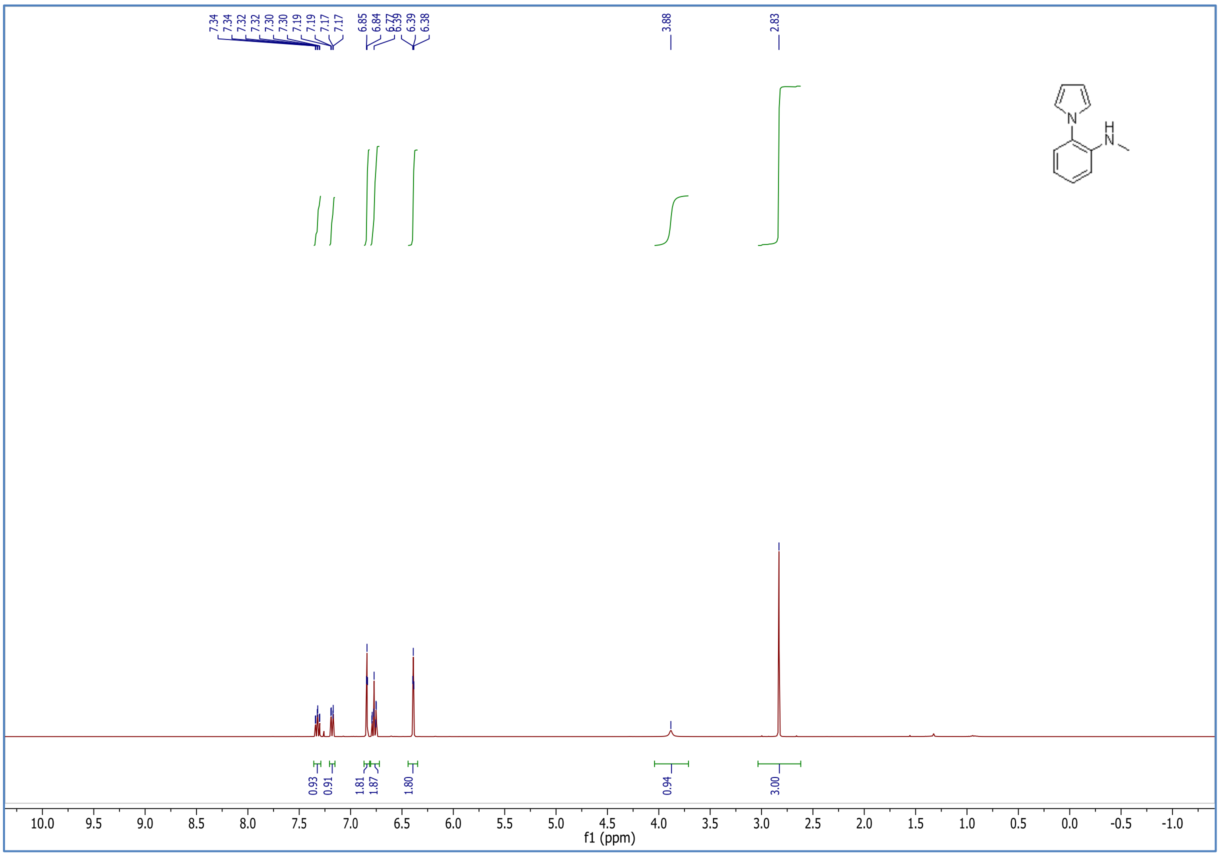

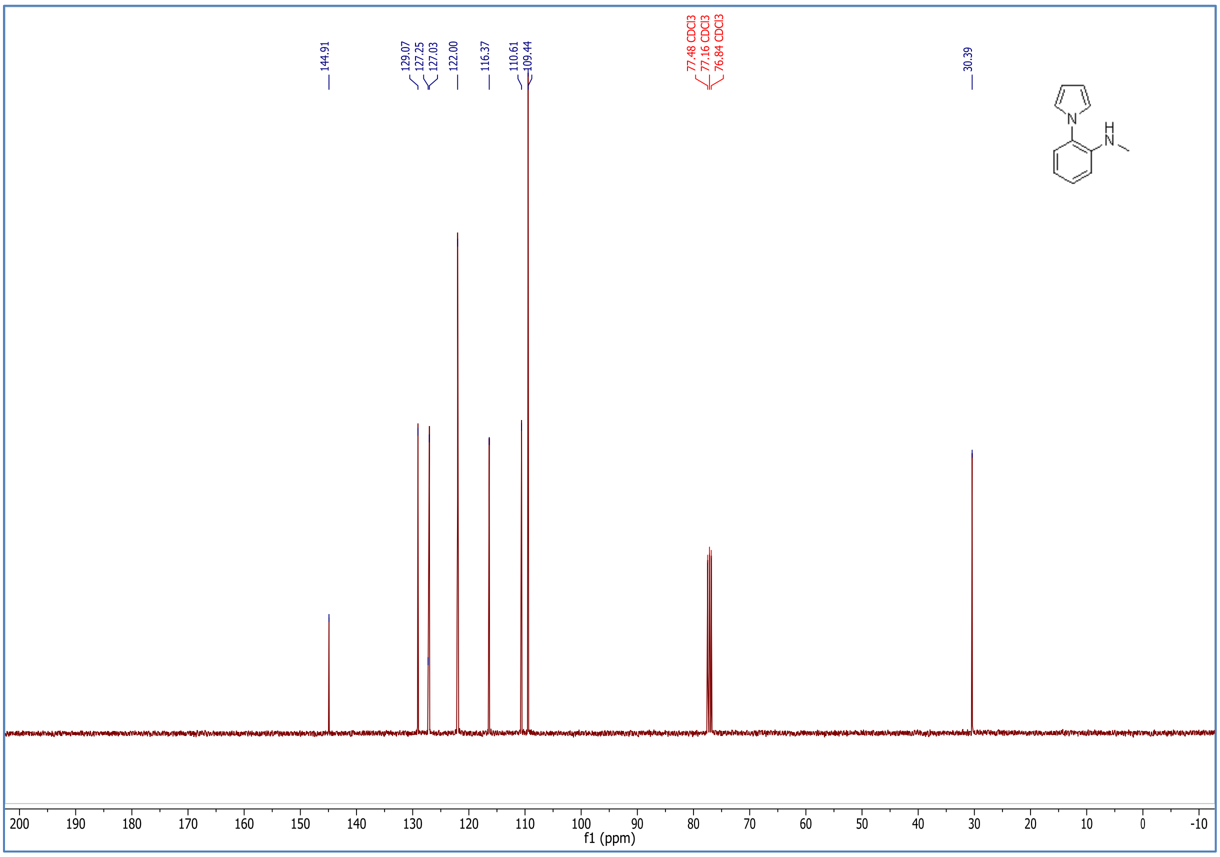

Supplementary Figure 63. ^{1}H and ^{13}C NMR of compound **9m**.

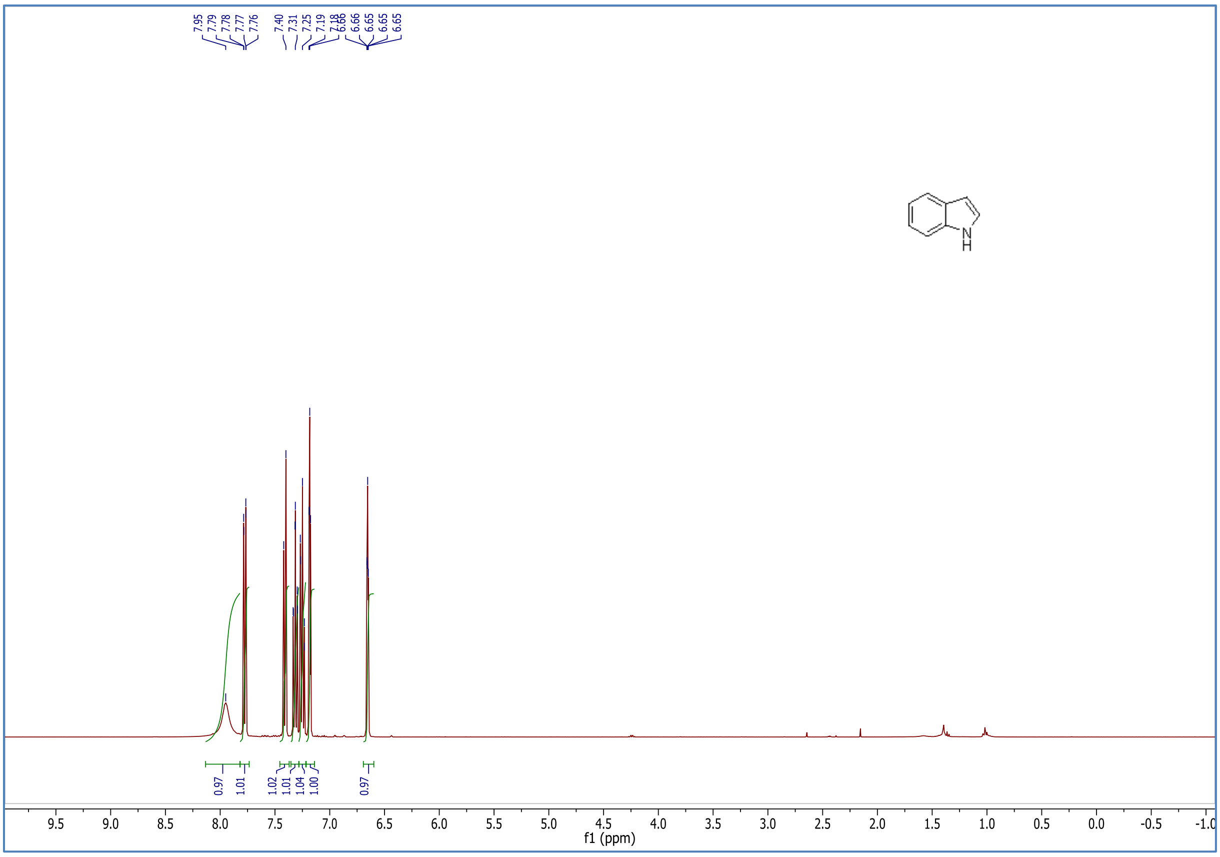

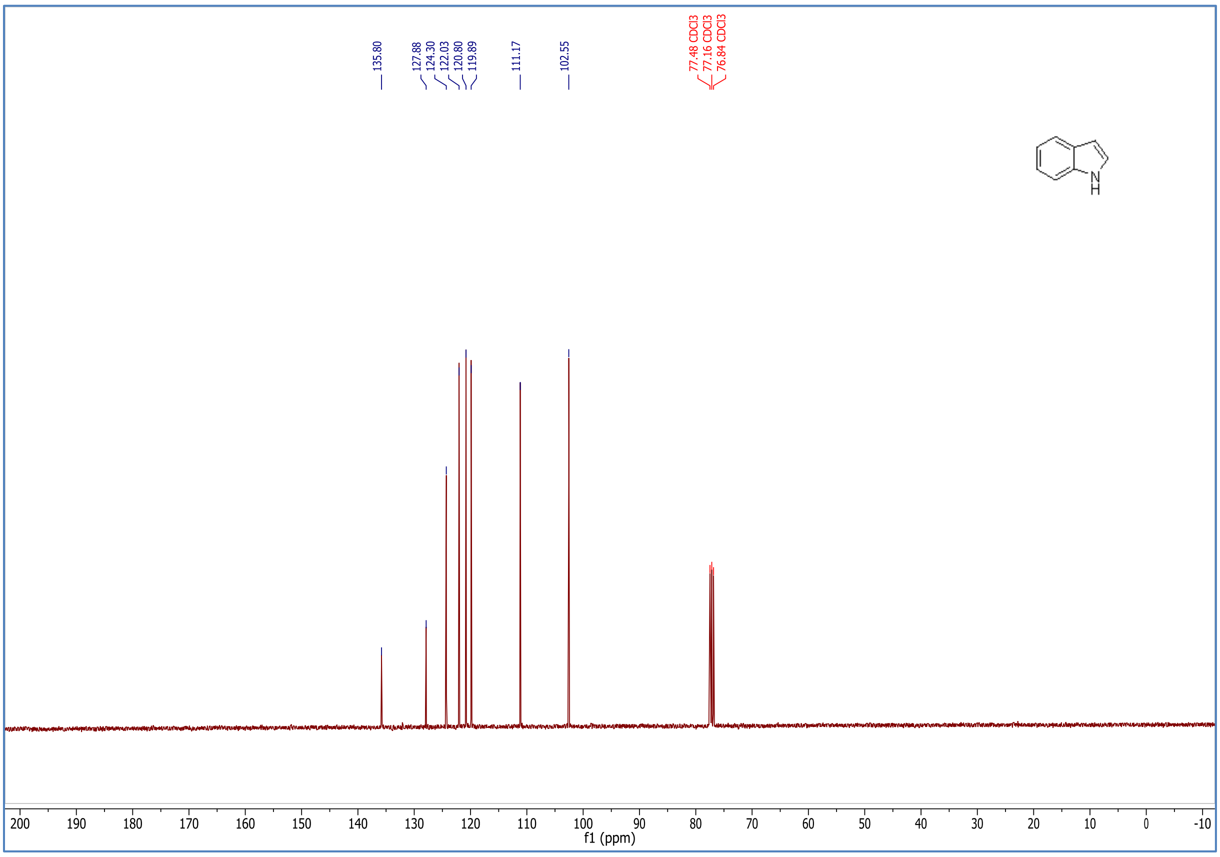

Supplementary Figure 64. ^{1}H and ^{13}C NMR of compound **8**.

Supplementary Table 1. Optimization with different bases[a]

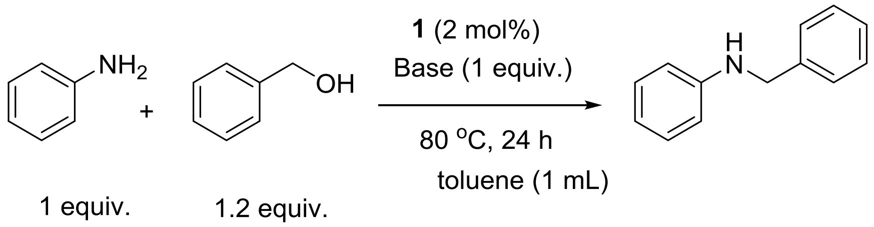

Entry[a]	Base	Conv. (%)[b]	Yield (%)[b]
1	*t*-BuOK	91	78
2	*t*-BuONa	65	45
3	Cs_2CO_3	20	14
4	KOH	36	25
5	NaOEt	14	12
6[c]	NaOMe	65	28

[a] 0.5 mmol scale. [b] Conversion and yield were determined by GC using hexadecane as an internal standard. Differences between conversion and yield were caused by the formation of corresponding secondary aldimine. [c] 11% *N*-methyl aniline were detected.

Supplementary Table 2. Solvent screening[a]

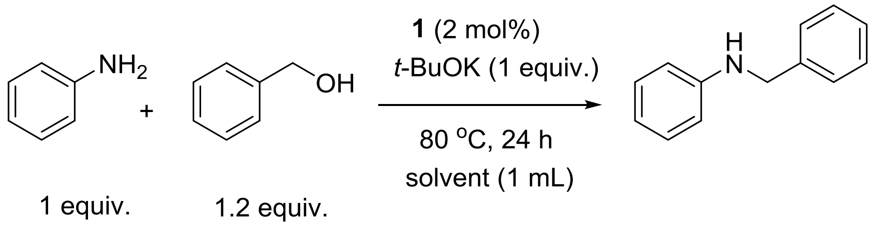

Entry[a]	Solvent	Conv.(%)[b]	Yield (%)[b]
1	*t*-amyl alcohol	44	15
2	THF	72	55
3	1,4 Dioxane	82	65
4	CPME	87	74
5	DMF	43	18
6	toluene	91	78

[a] 0.5 mmol scale. [b] Conversion and yield were determined by GC using hexadecane as an internal standard. Differences between conversion and yield were caused by the formation of corresponding secondary aldimine. CPME = cyclopentyl methyl ether.

Supplementary Table 3. Screening of the quantity of base[a]

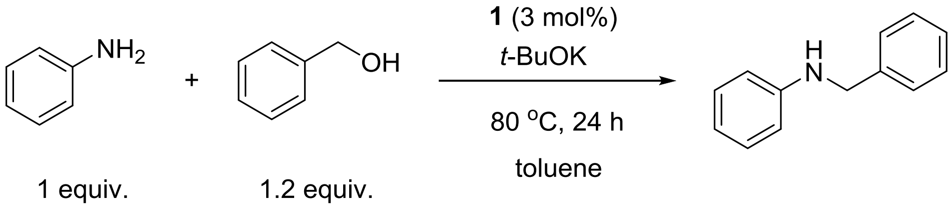

Entry[a]	t-BuOK (equiv.)	Conv. (%)[b]	Yield (%)[b]
1	0.1	14	12
2	0.2	27	20
3	0.5	73	46
4	0.75	99	97
5	1	99	96

[a] 0.5 mmol scale. [b] Conversion and yield were determined by GC using hexadecane as an internal standard. Differences between conversion and yield were caused by the formation of corresponding secondary aldimine.

Supplementary Table 4. Variation of reaction conditions with complex **1**[a]

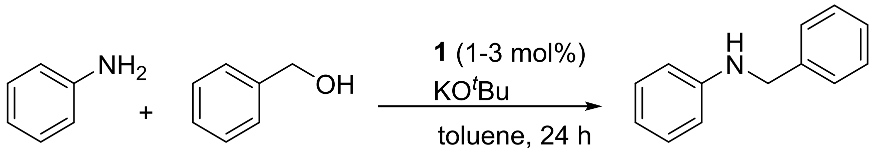

Entry	Amine (equiv.)	Alcohol (equiv.)	**1** (mol%)	t-BuOK (equiv.)	Temp (°C)	Yield[b] (%)
1[c]	1	1.2	**3**	-	140	-
2	1	1.2	**-**	1	80	-
3	1	1.2	**2**	1	80	78
4	1	**1.2**	3	1	80	96
5	**1.2**	1	3	1	80	91
6	1	1.2	3	**0.5**	80	46
7	1	1.2	3	**0.75**	80	97
8	1	1.2	**1**	1	80	54
9[d]	1	1.2	**1**	1	80	88
10	1	1.2	3	1	**60**	52
11	1	1.2	2	1	**100**	56
12[e]	1	1.2	2	1	100	71
13	**1**	**1**	2	1	80	66
14[f]	1	1.2	3	1	80	76
15[g]	1	1.2	3	0.2	80	47
16[h]	1	1.2	3	0.2	80	14

[a]0.5 mmol scale. [b]Conversion and yield were determined by GC using hexadecane as an internal standard [c]5% *N*-Benzylideneaniline detected. [d] 48 h. [e] Neat condition. [f]16 h reaction time. [g] 72 h. [h] Molecular sieves (100 mg) used.

Supplementary Methods

I. Synthesis and characterization of {MnBr(CO)$_2$[NH(CH$_2$CH$_2$P(*i*Pr)$_2$)$_2$]} (1)

Please note: The preparation of complexes **1, 2** and **3** has been submitted for publication.[1] We repeat here the reported syntheses for the reader's convenience**.**

To the orange-yellow suspension of Mn(CO)$_5$Br (719 mg, 2.6 mmol) in toluene (40 mL) was added [HN(CH$_2$CH$_2$P(*i*Pr$_2$)$_2$] (794 mg, 2,8 mmol, 10%wt in THF) dropwise. The suspension turned into a clear yellow solution within 10 min. After the gas evolution had ceased, the reaction mixture was heated to 100 °C and further stirred for 20 h under argon. During the heating, a yellow precipitate was formed. The reaction mixture was cooled to room temperature and concentrated *in vacuo*. The crude was thoroughly washed with heptane and dried under vaccum affording the title compound as yellow powder (1.162 mg, 90%).

^{1}H NMR (400 MHz, C$_6$D$_6$, 297 K): δ 3.27 (m, 2H, PC*H*(CH$_3$)$_2$), 2.83 (br. t, *J* = 12.5 Hz, 1H, N*H*), 2.47 (m, 2H, NC*H*$_2$CH$_2$), 2.22 (m, 2H, PC*H*(CH$_3$)$_2$), 1.95 (m, 2H, NC*H*$_2$CH$_2$), 1.66 (m, 2H, NCH$_2$C*H*$_2$), 1.52 (m, 6H, PCH(C*H*$_3$)$_2$), 1.32 (m, 6H, PCH(C*H*$_3$)$_2$), 1.25 (m, 2H, NCH$_2$C*H*$_2$), 1.22 (m, 6H, PCH(C*H*$_3$)$_2$), 1.07 (m, 6H, PCH(C*H*$_3$)$_2$).

^{31}P{^{1}H} NMR (162 MHz, C$_6$D$_6$, 297 K): δ = 81.8 (s).

^{13}C{^{1}H} NMR (101 MHz, C$_6$D$_6$, 297 K): δ 52.7 (vt, *J* = 5.2 Hz, N*C*H$_2$CH$_2$), 27.1 (vt, *J* = 5.9 Hz, NCH$_2$*C*H$_2$,), 26.1 (vt, *J* = 9.3 Hz, P*C*H(CH$_3$)$_2$), 24.4 (vt, *J* = 9.5 Hz, P*C*H(CH$_3$)$_2$), 20.3 (vt, *J* = 1 Hz, PCH(*C*H$_3$)$_2$), 20.1 (vt, *J* = 1 Hz, PCH(*C*H$_3$)$_2$), 18.9 (s, PCH(*C*H$_3$)$_2$), 18.4 (vt, *J* = 2 Hz, PCH(*C*H$_3$)$_2$).

IR-ATR (solid): ῡ [cm^{-1}] 1903 (s, ῡ CO), 1815 (s, ῡ CO).

ESI-HRMS (*m/z pos*): Calculated for [C$_{18}$H$_{36}$MnNO$_2$P$_2$]: 416.16745; found: 416.16752[M+H-Br]$^+$.

Elemental analysis: Calculated for [C$_{18}$H$_{37}$BrMnNO$_2$P$_2$]: C, 43.56; H, 7.51; N, 2.82. Found: C, 43.38; H, 7.45; N, 2.62.

II. Synthesis and characterization of {MnBr(CO)$_2$[NH(CH$_2$CH$_2$P(Cy)$_2$)$_2$]} (2)

To the orange-yellow suspension of Mn(CO)$_5$Br (110 mg, 0.22 mmol) in toluene (5 mL) was added [HN(CH$_2$CH$_2$P(Cy$_2$)$_2$] (57.5 mg, 0.21 mmol). The reaction mixture was heated to 100 °C and well stirred for a period of 20 h. The reaction mixture was then allowed to reach room temperature. Hereafter, the suspension was concentrated *in vacuo* leaving behind the

crude which was washed with heptane and dried under vaccum. The title compound was isolated as yellow solid (110 mg, 76%).

^{1}H NMR (400 MHz, C_6D_6, 297 K): δ 3.07 (br, 1H, NH), 3.03 (m, 2H, P-CH), 2.75 (m, 2H, Cy-CH_2), 2.65 (br, 2H, N-CH_2), 2.16 (m, 2H, P-CH), 2.02 (m, 2H N-CH_2 + 2H Cy-CH_2), 1.86 (m, 2H, P-CH_2), 1.33 (m, 2H, P-CH_2), remainder Cy-CH_2: 18 multiplets between 1.91 and 1.17 ppm (not further analyzed).

^{31}P{^{1}H} NMR (162 MHz, C_6D_6, 297 K): δ = 73.6 (s).

^{13}C{^{1}H} NMR (101 MHz, C_6D_6, 297 K): δ 52.6 (vt, *J* = 5.5 Hz, N-CH_2), 37.0 (vt, *J* = 8.5 Hz, P-CH), 36.2 (vt, *J* = 9 Hz, P-CH), 30.9 (s), 30.2 (s), 29.4 (vt), 28.4 (s), 28.3 (vt), 28.1 (vt), 27.9 (vt), 27.6 (vt), 26.8 (s), 26.5 (s), all Cy-CH_2, 25.5 (vt, *J* = 6 Hz, P-CH_2).
IR-ATR (solid): ῡ [cm^{-1}] 1913 (s, ῡ CO), 1826 (s, ῡ CO).
ESI-HRMS (*m/z pos*): Calculated for [$C_{30}H_{53}MnNO_2P_2$]: 576.29265; found: 576.29253[M-Br]$^+$

III. Synthesis of {$MnCl_2$[NH(CH_2CH_2P(*i*Pr)$_2$)$_2$]} (3)

A dried Schlenk tube was charged with $MnCl_2$ (41 mg, 0.33 mmol), THF (5 mL) and [HN(CH_2CH_2P(*i*Pr_2)$_2$] (100 mg, 0.33 mmol, 10%wt in THF) in this order and the reaction mixture was stirred overnight. A white precipitate was formed and the volatiles were removed *in vacuo* and isolated as a white solid with 76% yield.

ESI-HRMS (*m/z pos*): Calculated for [$C_{16}H_{37}Cl_2MnNP_2$]: 431.12315; found: 431.12334.

Elemental analysis: Calculated for [$C_{16}H_{37}Cl_2MnNP_2$]: C, 44.56; H, 8.65; N, 3.25. Found: C, 44.67; H, 8.68; N, 3.40.

IV. Synthesis and characterization of complex 4.

Synthesis and characterization of this complex reported in the following manuscript.

Gonzalez, M. A. *et al.* Manganese carbonyls bearing tripodal polypyridine ligands as photoactive carbon monoxide-releasing molecules. *Inorg. chem.* **51**, 601-608 (2012).

^{1}H NMR (300 MHz, DMSO-*d_6*) δ 8.94 (d, *J* = 5.0 Hz, 2H), 7.90 (t, *J* = 7.7 Hz, 2H), 7.56 (t, *J* = 7.0 Hz, 1H), 7.50 (d, *J* = 7.8 Hz, 2H), 7.42 (t, *J* = 6.6 Hz, 2H), 4.80-4.72 (m, 2H), 4.43 (br d, *J* = 17.5 Hz, 2H).

^{13}C NMR (75 MHz, DMSO-d_6) δ 219.4, 160.9, 151.9, 139.1, 125.0, 122.4, 61.0.

IR-ATR (solid): ῡ [cm^{-1}] 2026 (s, ῡ CO), 1913 (s, ῡ CO), 1888 (s, ῡ CO).

V. Typical procedure (A) for the manganese catalyzed *N*-alkylation of amines with alcohols

An oven-dried 25-mL Schlenk tube, prepared with a stirring bar, was charged with the yellow complex **1** (14.9 mg, 0.03 mmol), *t*-BuOK (84 mg, 0.75 mmol) and dry toluene (2 mL). Then, the corresponding alcohol (1.2 mmol) and amine (1 mmol) were added to the red colored suspension. Solid materials were weighed into the Schlenk tube under air, and the Schlenk tube was subsequently connected to a Schlenk line and vacuum-argon exchange was done for three times. Liquid compounds and solvent were charged under an argon flow. The Schlenk tube was placed into an aluminum block and heated to 80 °C and stirred for a given time. The reaction mixture was cooled to RT, quenched with water and extracted with ethyl acetate. The organic layers were then dried over $MgSO_4$ and concentrated under reduced pressure. The residue was purified by flash chromatography on silica gel (*n*-pentane/diethylether) to afford the desired product. The product was analyzed by ^{1}H- and ^{13}C-NMR spectroscopy.

VI. General procedure (B) for the *N*-methylation of amines using methanol

An oven dried pressure tube was charged with Mn complex **1** (14.9 mg, 0.03 mmol) and *t*-BuOK (112 mg, 1 mmol). Amines (1 mmol) were weighed into the pressure tube under air, and the pressure tube was connected to a Schlenk line and vacuum-argon exchange was performed three times. Liquid amines and dry, degassed methanol (1 mL) were charged under an argon stream after the three vacuum-argon exchanges. The pressure tube was closed with a Teflon® stopper and was heated to 100 °C. After 24 h, the reaction mixture was cooled to room temperature and extracted with ethyl acetate / water. The organic layer was dried over $MgSO_4$ and transferred into a round bottom flask. SiO_2 (350 mg) was added to the mixture. The solvent was removed *in vacuo* and the product was purified by column chromatography using heptane and ethyl acetate. The product was analyzed by ^{1}H- and ^{13}C-NMR spectroscopy.

VII. Data for isolated products

N-Benzyl-4-methoxyaniline (5b)[2]

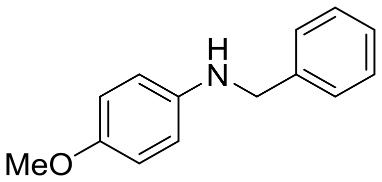

The compound was prepared as described in the general procedure **A** (m = 191 mg, 90% isolated yield).Yellow oil; **^{1}H NMR** (300 MHz, $CDCl_3$): δ 7.42 – 7.30 (m, 5H), 6.84 – 6.81 (m, 2H), 6.80 – 6.61 (m, 2H), 4.31 (s, 2H), 3.77 (s, 4H). **^{13}C NMR** (75 MHz, $CDCl_3$): δ 152.3, 142.4, 139.6, 128.7, 127.7, 127.3, 114.9, 114.9, 55.8, 49.4.

N-Benzyl-4-methylaniline (5c)[3]

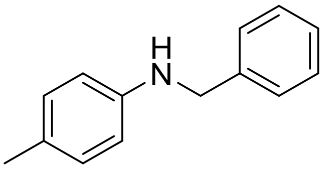

The compound was prepared as described in the general procedure **A** (m = 171 mg, 87% isolated yield). Colorless oil; **^{1}H NMR** (300 MHz, $CDCl_3$): δ 7.58 – 7.33 (m, 5H), 7.21 – 7.01 (m, 2H), 6.79 – 6.58 (m, 2H), 4.41 (s, 2H), 2.47 – 2.27 (m, 3H). **^{13}C NMR** (75 MHz, $CDCl_3$): δ 145.9, 129.8, 128.6, 127.5, 127.2, 126.7, 113.1, 48.6, 20.5.

N-Benzyl-4-ethoxyaniline (5d)[4]

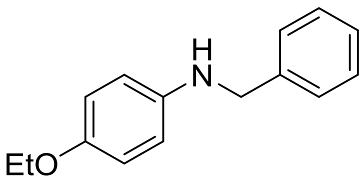

The compound was prepared as described in the general procedure **A** (m = 180 mg, 79% isolated yield). White solid; **^{1}H NMR** (300 MHz, $CDCl_3$): δ 7.44 – 7.32 (m, 5H), 6.87 – 6.84 (m, 2H), 6.83 – 6.62 (m, 2H), 4.32 (s, 2H), 4.00 (q, *J* = 7.0 Hz, 2H), 3.77 (s, 1H), 1.43 (t, *J* = 7.0 Hz, 3H). **^{13}C NMR** (75 MHz, $CDCl_3$): δ 151.5, 142.4, 139.7, 128.6, 127.6, 127.2, 115.8, 114.2, 64.1, 49.3, 15.1.

N-Benzyl-4-(methylthio)aniline (5e)[5]

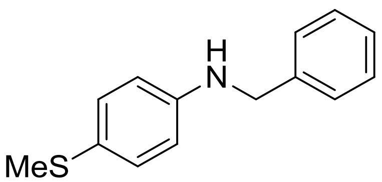

The compound was prepared as described in the general procedure **A** (m = 161 mg, 71% isolated yield). yellow oil; **^{1}H NMR** (300 MHz, $CDCl_3$): δ 7.28 – 7.16 (m, 5H), 7.15 – 7.08 (m, 2H), 6.50 – 6.45 (m, 2H), 4.20 (s, 2H), 3.97 (s, 1H), 2.30 (s, 3H). **^{13}C NMR** (75 MHz, $CDCl_3$): δ 146.9, 139.2, 131.4, 128.7, 127.4, 127.3, 124.4, 113.5, 48.2, 19.1.

***N*-Benzyl-4-bromoaniline (5h)**[3]

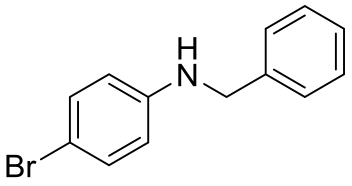

The compound was prepared as described in the general procedure **A** (m = 231 mg, 88% isolated yield). Colorless oil; **^{1}H NMR** (300 MHz, $CDCl_3$): δ 7.29 – 7.12 (m, 7H), 6.42 – 6.37 (m, 2H), 4.19 (s, 2H), 3.96 (s, 1H). **^{13}C NMR** (75 MHz, $CDCl_3$): δ 147.1, 138.9, 131.9, 128.7, 127.4, 127.4, 114.5, 109.2, 48.2.

***N*-Benzyl-2-chloroaniline (5i)**[6]

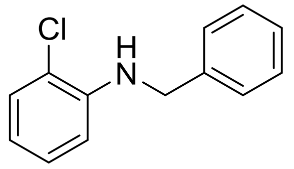

The compound was prepared as described in the general procedure **A** (m = 152 mg, 70% isolated yield). Colorless oil; **^{1}H NMR** (300 MHz, $CDCl_3$): δ 7.45 – 7.34 (m, 6H), 7.19 – 7.14 (m, 1H), 6.72 (td, *J* = 7.6, 1.1 Hz, 2H), 4.84 (s, 1H), 4.46 (s, 2H). **^{13}C NMR** (75 MHz, $CDCl_3$): δ 143.9, 138.8, 129.2, 128.8, 128.7, 127.9, 127.4, 127.3, 119.2, 117.5, 111.6, 47.9.

***N*-Benzyl-3-(trifluoromethyl)aniline (5j)**[7]

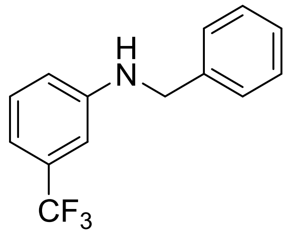

The compound was prepared as described in the general procedure **A** (m = 212 mg, 84% isolated yield). Colorless oil; **^{1}H NMR** (300 MHz, $CDCl_3$): δ 7.47 – 7.28 (m, 6H), 7.05-7.02 (m, 1H), 6.93-6.91 (m, 1H), 6.82 – 6.81 (m, 1H), 4.39 (s, 2H), 4.33 – 4.19 (m, 1H). **^{13}C NMR** (75 MHz, $CDCl_3$): δ 148.3, 138.6, 131.6 (q, $J_{C\text{-}F}$ = 31 Hz), 129.8, 128.8, 127.6, 126.3, 122.3, 115.8, 113.9 (q, $J_{C\text{-}F}$ = 3.9 Hz), 109.1 (q, $J_{C\text{-}F}$ = 3.9 Hz), 48.1.

N-Benzyl-3,5-dimethoxyaniline (5f)[8]

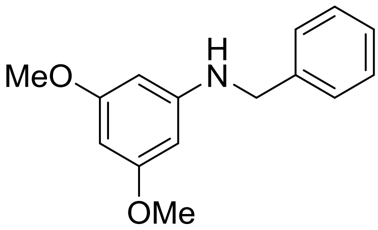

The compound was prepared as described in the general procedure **A** (m = 221 mg, 91% isolated yield). Colorless oil; **^{1}H NMR** (300 MHz, $CDCl_3$): δ 7.27 – 7.13 (m, 5H), 5.81-5.73 (m, 3H), 4.18 (s, 2H), 4.00 (br.s, 1H), 3.62 (s, 6H). **^{13}C NMR** (75 MHz, $CDCl_3$): δ 161.8, 150.1, 139.3, 128.7, 127.6, 127.3, 91.8, 90.0, 55.2, 48.4.

***N*-Benzyl-3,5-di-*tert*-butylaniline (5g)**

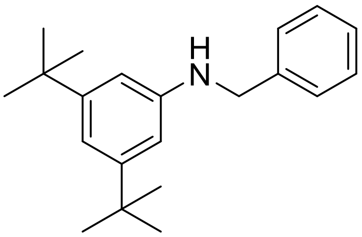

The compound was prepared as described in the general procedure **A** (m = 181 mg, 61% isolated yield). Colorless oil; **^{1}H NMR** (300 MHz, $CDCl_3$): δ 7.59– 7.44 (m, 5H), 7.04 (t, J = 1.6 Hz, 1H), 6.72 (d, J = 1.6 Hz, 2H), 4.50 (s, 2H), 4.09 (br. s, 1H), 1.50 (s, 18H). **^{13}C NMR** (75 MHz, $CDCl_3$): δ 151.7, 147.7, 139.8, 128.6, 127.9, 127.3, 112.4, 107.7, 48.9, 34.9, 31.6. **GCMS-EI** (70 eV): m/z (%) = 295 (M^+, 100), 280 (11), 207 (3), 91 (36). **HRMS** (EI, m/z) calcd. for $C_{21}H_{29}N$: 295.22945; found: 295.22920.

***N*-Benzyl-4-vinylaniline (5k)**

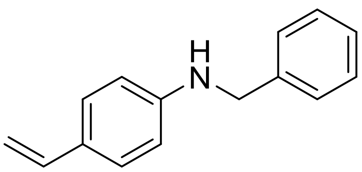

The compound was prepared as described in the general procedure **A** (m = 185 mg, 89% isolated yield). Yellow sticky solid; **^{1}H NMR** (300 MHz, $CDCl_3$): δ 7.58 – 7.24 (m, 7H), 6.79 – 6.60 (m, 3H), 5.64 (dd, J = 17.6, 1.1 Hz, 1H), 5.13 (dd, J = 10.9, 1.1 Hz, 1H), 4.40 (s, 2H), 4.17 (s, 1H). **^{13}C NMR** (75 MHz, $CDCl_3$): δ 147.9, 139.3, 136.7, 128.7, 127.5, 127.4, 127.4, 127.3, 112.7, 109.5, 48.2. **GCMS-EI** (70 eV): m/z (%) = 209 (M^+, 82), 132 (11), 91 (100), 77 (12), 65 (17). **HRMS** (EI, m/z) calcd. for $C_{15}H_{15}N$: 209.11990; found: 209.11981.

***N*-Benzyl-3-vinylaniline (5l)**

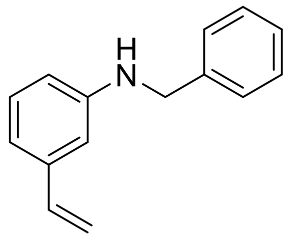

The compound was prepared as described in the general procedure **A** (m = 189 mg, 90% isolated yield). Colorless liquid; **^{1}H NMR** (300 MHz, $CDCl_3$): δ 7.49 – 7.22 (m, 6H), 6.94 – 6.72 (m, 4H), 5.81 (dd, *J* = 17.6, 1.1 Hz, 1H), 5.32 (dd, *J* = 10.9, 1.1 Hz, 1H), 4.42 (s, 2H), 4.09 (s, 1H). **^{13}C NMR** (75 MHz, $CDCl_3$): δ 148.3, 139.4, 138.6, 137.3, 129.4, 128.8, 127.6, 127.3, 115.9, 113.5, 112.6, 110.7, 48.3. **GCMS-EI** (70 eV): m/z (%) = 209 (M^+, 92), 208 (43), 132 (18), 91 (100), 77 (14), 65 (18). **HRMS** (EI, m/z) calcd. for $C_{15}H_{15}N$: 209.11990; found: 209.11960.

***N*-Benzylpyridin-2-amine (5m)**[6]

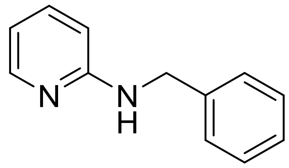

The compound was prepared as described in the general procedure **A** (m = 170 mg, 92% isolated yield). White solid; **^{1}H NMR** (300 MHz, $CDCl_3$): δ 7.96-7.94 (m, 1H), 7.29 – 7.23 (m, 6H), 6.47-6.43 (m, 1H), 6.24 (dt, *J* = 8.4, 0.9 Hz, 1H), 5.18 (s, 1H), 4.38 (d, *J* = 5.7 Hz, 2H). **^{13}C NMR** (75 MHz, $CDCl_3$): δ 158.7, 148.1, 139.2, 137.5, 128.6, 127.4, 127.2, 113.1, 106.7, 46.3.

***N*-Benzyl-2,3-dihydrobenzo[b][1,4]dioxin-6-amine (5o)**

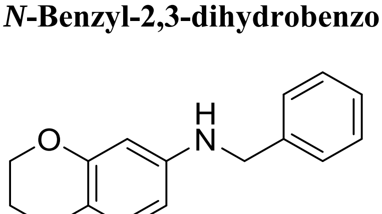

The compound was prepared as described in the general procedure **A** (m = 203 mg, 84% isolated yield). White crystal; **^{1}H NMR** (300 MHz, $CDCl_3$): δ 7.45 – 7.28 (m, 5H), 6.76 (d, *J* = 8.5 Hz, 1H), 6.26 – 6.20 (m, 2H), 4.28 (s, 2H), 4.25-4.17 (m, 4H), 3.79 (s, 1H). **^{13}C NMR** (75 MHz, $CDCl_3$): δ 144.0, 143.2, 139.6, 135.7, 128.6, 127.6, 127.2, 117.6, 106.7, 101.6, 64.7, 64.2, 49.0. **GCMS-EI** (70 eV): m/z (%) = 241 (M^+, 100), 184 (11), 150 (52), 122 (37), 91 (46). **HRMS** (EI, m/z) calcd. for $C_{15}H_{15}O_2N$: 241.10973; found: 241.10968.

***N*-Benzylbenzo[d][1,3]dioxol-5-amine (5p)**

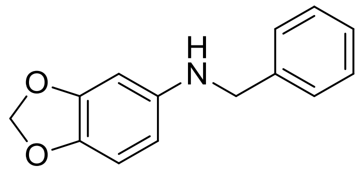

The compound was prepared as described in the general procedure **A** (m = 204 mg, 89% isolated yield). White solid; **^{1}H NMR** (400 MHz,$CDCl_3$): δ 7.41 – 7.27 (m, 5H), 6.65 (d, *J* = 8.2 Hz, 1H), 6.30 (d, *J* = 2.1 Hz, 1H), 6.11 (dd, *J* = 8.2, 2.1 Hz, 1H), 5.85 (s, 2H), 4.27 (s, 2H). **^{13}C NMR** (101 MHz,$CDCl_3$): δ 148.3, 143.2, 140.1, 138.9, 128.6, 127.7, 127.4, 108.6, 105.1, 100.6, 96.5, 49.6. **GCMS-EI** (70 eV): m/z (%) = 227 (M+, 100), 136 (72), 91 (64), 65 (13).

***N*-Benzyl-9H-fluoren-2-amine (5q)**

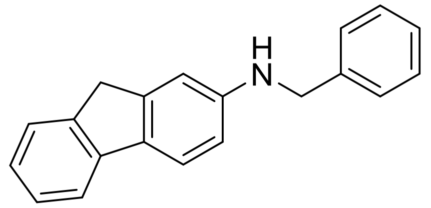

The compound was prepared as described in the general procedure **A** (m = 200 mg, 74% isolated yield). Yellow solid; **^{1}H NMR** (300 MHz, $CDCl_3$): δ 7.66-7.63 (m, 1H), 7.57 (d, *J* = 8.1 Hz, 1H), 7.38 – 7.29 (m, 6H), 7.22 – 7.12 (m, 2H), 6.70 (dd, *J* = 8.1, 2.1 Hz, 1H), 6.51 (d, *J* = 2.1 Hz, 1H), 4.18 (t, *J* = 7.6 Hz, 1H), 3.63 (s, 2H), 3.12 (d, *J* = 7.6 Hz, 2H). **^{13}C NMR** (75 MHz, $CDCl_3$): δ 148.8, 146.0, 145.7, 141.3, 140.1, 131.9, 129.6, 128.3, 127.1, 126.4, 125.1, 124.6, 120.6, 118.6, 114.3, 111.8, 48.5, 40.3. **GCMS-EI** (70 eV): m/z (%) = 271 (M^+, 48), 180 (100), 152 (21), 91 (8). **HRMS** (EI, m/z) calcd. for $C_{20}H_{17}N$: 271.13555; found: 271.13545.

***N*-Benzylphenanthren-9-amine (5r)**

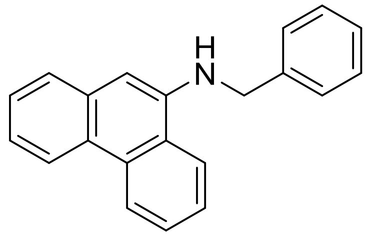

The compound was prepared as described in the general procedure **A** (m = 257 mg, 91% isolated yield). White solid; **^{1}H NMR** (300 MHz, $CDCl_3$): δ 8.75 (dd, *J* = 8.2, 1.4 Hz, 1H), 8.59 (dd, *J* = 8.3, 1.3 Hz, 1H), 7.94 – 7.89 (m, 1H), 7.74 – 7.60 (m, 3H), 7.56 – 7.51 (m, 3H), 7.48 – 7.38 (m, 4H), 6.88 (s, 1H), 4.71 (s, 1H), 4.59 (s, 2H). **^{13}C NMR** (75 MHz, $CDCl_3$): δ

140.9, 139.1, 133.7, 131.2, 128.9, 128.0, 127.6, 127.0, 126.8, 126.6, 126.4, 125.5, 125.4, 123.6, 123.1, 122.5, 120.5, 102.8, 48.8. **GCMS-EI** (70 eV): m/z (%) = 283 (M^+, 100), 192 (12), 165 (86), 91 (27). **HRMS** (EI, m/z) calcd. for $C_{21}H_{17}N$ (M)+ 283.13555; found 283.13516.

***N*-Benzyl-[1,1'-biphenyl]-2-amine (5s)**

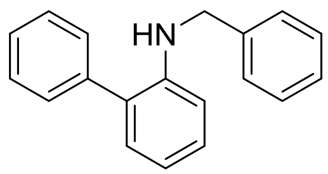

The compound was prepared as described in the general procedure **A** (m = 194 mg, 75% isolated yield).Colorless crystals; **^{1}H NMR** (400 MHz, $CDCl_3$): δ 7.54 – 7.44 (m, 4H), 7.42 – 7.31 (m, 5H), 7.31 – 7.26 (m, 1H), 7.25 – 7.19 (m, 1H), 7.15 (dd, *J* = 7.4, 1.7 Hz, 1H), 6.82 (td, *J* = 7.4, 1.1 Hz, 1H), 6.70 (dd, *J* = 8.2, 1.1 Hz, 1H), 4.44 (s, 1H), 4.37 (s, 2H). **^{13}C NMR** (101 MHz,$CDCl_3$): δ 145.0, 139.6, 130.4, 129.5, 129.1, 128.8, 128.7, 127.8, 127.4, 127.2, 117.3, 110.9, 48.3. **GCMS-EI** (70 eV): m/z (%) = 260 (20), 259 (M+, 100), 258 (37), 182 (12), 168 (28), 167 (38), 166 (10), 152 (11), 91 (50), 65 (10).

***N*-(4-Methoxybenzyl)aniline (6a)**[6]

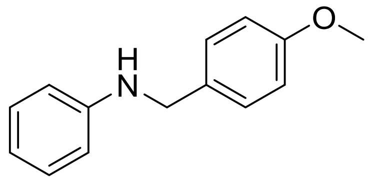

The compound was prepared as described in the general procedure **A** (m = 173 mg, 81% isolated yield). Colorless oil; **^{1}H NMR** (300 MHz, $CDCl_3$): δ 7.42 – 7.38 (m, 2H), 7.33 – 7.26 (m, 2H), 7.02 – 6.97 (m, 2H), 6.84 (tt, *J* = 7.3, 1.1 Hz, 1H), 6.79 – 6.70 (m, 2H), 4.35 (s, 2H), 3.90 (s, 3H, *NH*). **^{13}C NMR** (75 MHz, $CDCl_3$): δ 158.9, 148.2, 131.4, 129.3, 128.8, 117.6, 114.1, 114.1, 112.9, 55.3, 47.8.

***N*-(4-Chlorobenzyl)aniline (6c)**[6]

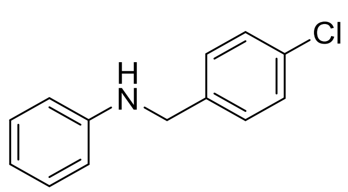

The compound was prepared as described in the general procedure **A** (m = 207 mg, 95% isolated yield). Colorless oil; **^{1}H NMR** (300 MHz, $CDCl_3$): δ 7.26-7.78 (m, 4H), 7.17 – 7.12 (m, 2H), 6.73 – 6.71 (m, 1H), 6.69 – 6.56 (m, 2H), 4.25 (s, 2H), 4.02 (m, 1H). **^{13}C NMR** (75 MHz, $CDCl_3$): δ 147.9, 138.1, 132.9, 129.4, 128.8, 128.8, 117.9, 113.0, 47.7.

4-Methoxy-*N*-(4-methylbenzyl)aniline (6b)[7]

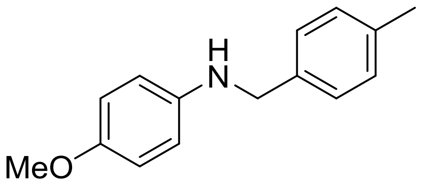

The compound was prepared as described in the general procedure **A** (m = 142 mg, 63% isolated yield). White solid; **^{1}H NMR** (300 MHz, $CDCl_3$): δ 7.35 – 7.25 (m, 2H), 7.24 – 7.21 (m, 2H), 6.88 – 6.83 (m, 2H), 6.69 – 6.64 (m, 2H), 4.30 (s, 2H), 3.80 (s, 4H), 2.43 (s, 3H). **^{13}C NMR** (75 MHz, $CDCl_3$): δ 152.2, 142.6, 136.7, 136.7,129.3, 127.6, 114.9, 114.1, 55.8, 48.9, 21.2.

N-(3,5-Dimethylbenzyl)aniline (6c)

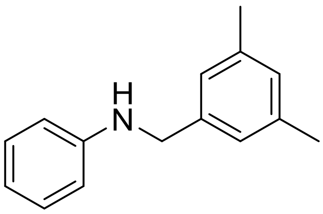

The compound was prepared as described in the general procedure **A** (m = 194 mg, 92% isolated yield). Colorless oil; **^{1}H NMR** (300 MHz, $CDCl_3$): δ 7.37-7.32 (m, 2H), 7.16 – 7.09 (m, 3H), 6.92-6.89 (s, 1H), 6.87-6.86 (dt, J = 7.7, 1.1 Hz, 2H), 4.37 (s, 2H), 4.20 – 3.96 (m, 1H), 2.48 (s, 6H). **^{13}C NMR** (75 MHz, $CDCl_3$): δ 148.3, 139.4, 138.2, 129.3, 128.9, 125.4, 117.5, 112.8, 48.4, 21.3. **GCMS-EI** (70 eV): m/z (%) = 211 (M^+, 63), 196 (8), 119 (100), 104 (9), 91 (15), 77 (19). **HRMS** (EI, m/z) calcd. for $C_{25}H_{17}N$ (M)+ 211.13555; found 211.13547.

4-Methoxy-*N*-(naphthalen-1-ylmethyl)aniline (6e)[9]

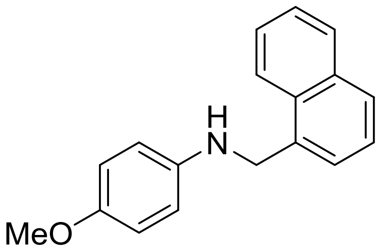

The compound was prepared as described in the general procedure A (m = 174 mg, 66% isolated yield). Yellow solid; **^{1}H NMR** (300 MHz, $CDCl_3$): δ 8.20 – 8.08 (m, 1H), 8.03 – 7.90 (m, 1H), 7.91 – 7.82 (m, 1H), 7.65 – 7.52 (m, 3H), 7.48 (dd, *J* = 8.1, 7.0 Hz, 1H), 7.01 – 6.79 (m, 2H), 6.81 – 6.52 (m, 2H), 4.72 (s, 2H), 3.82 (s, 3H (OMe)+1H (NH)). **^{13}C NMR** (75 MHz, $CDCl_3$): δ 152.2, 142.6, 134.7, 133.9, 131.6, 128.8, 128.1, 126.3, 126.0, 125.8, 125.6, 123.6, 115.0, 114.0, 55.8, 47.3.

***N*-(Furan-2-ylmethyl)aniline (6f)**[10]

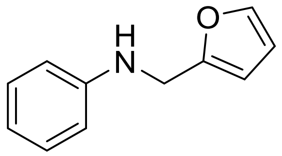

The compound was prepared as described in the general procedure **A** (m = 88mg, 51% isolated yield). Colorless oil; **^{1}H NMR** (300 MHz, $CDCl_3$): δ 7.27-7.26 (m, 1H), 7.12 – 7.06 (m, 2H), 6.65-6.58 (m, 1H), 6.56 – 6.55 (m, 2H), 6.23-6.21(m, 1H), 6.14-6.12 (m, 1H), 4.20 (s, 2H), 3.83 (s, 1H). **^{13}C NMR** (75 MHz, $CDCl_3$): δ 152.8, 147.6, 141.9, 129.3, 118.1, 113.2, 110.4, 107.1, 41.5.

***N*-(Furan-2-ylmethyl)-4-methoxyaniline (6g)**[7]

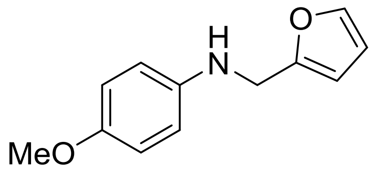

The compound was prepared as described in the general procedure **A** (m = 75 mg, 37% isolated yield). Yellow oil; **^{1}H NMR** (300 MHz, $CDCl_3$): δ 7.39-7.38 (m, 1H), 6.84 – 6.79 (m, 2H), 6.69 – 6.64 (m, 2H), 6.35-6.33 (s, 1H), 6.25-6.23 (m, 1H), 4.28 (s, 2H), 3.76 (s, 4H). **^{13}C NMR** (75 MHz, $CDCl_3$): δ 153.1, 152.6, 141.9, 141.8, 114.8, 114.7, 110.4, 106.9, 55.7, 42.5.

***N*-(Benzo[d][1,3]dioxol-5-ylmethyl)aniline (6h)**[11]

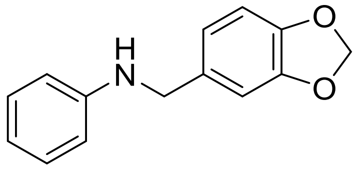

The compound was prepared as described in the general procedure **A** (m = 195 mg, 86% isolated yield). White solid; **^{1}H NMR** (300 MHz, $CDCl_3$): δ 7.29 – 7.18 (m, 2H), 6.95 – 6.74 (m, 4H), 6.72 – 6.63 (m, 2H), 5.97 (s, 2H), 4.27 (s, 2H), 4.04 (s, 1H). **^{13}C NMR** (75 MHz, $CDCl_3$): δ 148.1, 147.9, 146.8, 133.4, 129.3, 120.6, 117.6, 112.9, 108.4, 108.1, 101.0, 48.2.

***N*-(Thiophen-3-ylmethyl)pyridin-2-amine (6i)**

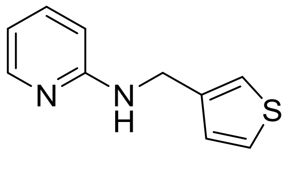

The compound was prepared as described in the general procedure **A** (m = 151 mg, 80% isolated yield). White solid; **^{1}H NMR** (300 MHz, $CDCl_3$): δ 8.21 – 7.90 (m, 1H), 7.39 (ddd, *J* = 8.5, 7.1, 1.5 Hz, 1H), 7.31 – 7.23 (m, 1H), 7.19 – 7.13 (m, 1H), 7.06 (dd, *J* = 4.9, 1.3 Hz, 1H), 6.60-6.56 (m, *J* = 7.2, 5.0, 1.0 Hz, 1H), 6.38 (d, *J* = 7.9 Hz, 1H), 5.12 (s, 1H), 4.49 (d, *J* = 5.3 Hz, 2H). **^{13}C NMR** (75 MHz, $CDCl_3$): δ 158.6, 148.2, 140.3, 137.5, 127.2, 126.2, 121.6, 113.2, 106.9, 41.8**. GCMS-EI** (70 eV): m/z (%) = 190 (M^+, 100), 189 (23), 157 (11), 112 (57), 97 (79), 78 (38).

***N*-(Thiophen-2-ylmethyl)pyridin-2-amine (6j)**

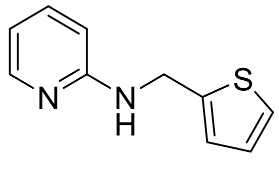

The compound was prepared as described in the general procedure **A** (m = 140 mg, 74% isolated yield). White solid; **^{1}H NMR** (300 MHz, $CDCl_3$): δ 8.13-8.10 (m, 1H), 7.44-7.38 (m, 1H), 7.20 (dd, *J* = 5.0, 1.3 Hz, 1H), 7.02-7.00 (m, 1H), 6.97-6.94 (m, 1H), 6.63-6.58 (m, 1H), 6.42 (d, *J* = 8.4 Hz, 1H), 5.08 (s, 1H), 4.69 (dd, *J* = 5.9, 1.0 Hz, 2H). **^{13}C NMR** (75 MHz, $CDCl_3$): δ 158.2, 148.2, 142.7, 137.5, 126.8, 125.2, 124.7, 113.5, 107.4, 41.3. GCMS-EI (70eV): m/z (%) = 190 (M^+, 95), 157 (20), 112 (41), 97 (100), 78 (30).

***N*-(4-(Methylthio)benzyl)pyridin-2-amine (6k)**

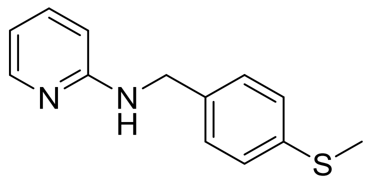

The compound was prepared as described in the general procedure **A** (m = 162 mg, 71% isolated yield). White solid; **^{1}H NMR** (300 MHz, $CDCl_3$): δ 8.18 (ddd, *J* = 5.2, 1.9, 0.9 Hz, 1H), 7.49 (ddd, *J* = 8.4, 7.1, 1.9 Hz, 1H), 7.44 – 7.29 (m, 4H), 6.68 (ddd, *J* = 7.2, 5.0, 0.9 Hz, 1H), 6.46 (dt, *J* = 8.5, 0.9 Hz, 1H), 5.19 (s, 1H), 4.56 (d, *J* = 5.7 Hz, 2H), 2.57 (s, 3H). **^{13}C NMR** (75 MHz, $CDCl_3$): δ 158.6, 148.1, 137.5, 137.2, 136.2, 128.0, 127.0, 113.2, 106.8, 45.8, 16.1. **GCMS-EI** (70 eV): m/z (%) = 230 (M^+, 82), 215 (7), 152 (28), 137 (100), 122 (18), 78 (26). **HRMS** (EI, m/z) calcd. for $C_{13}H_{14}N_2S$ $(M)^+$ 230.08722; found 230.08720.

2-(1H-Pyrrol-1-yl)-N-(thiophen-3-ylmethyl)aniline (6l)

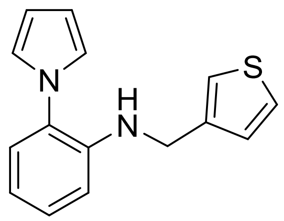

The compound was prepared as described in the general procedure **A** (m = 170 mg, 67% isolated yield). Colorless oil; ^{1}**H NMR** (300 MHz, $CDCl_3$): δ 7.39 – 7.33 (m, 2H), 7.28 – 7.23 (m, 1H), 7.19 – 7.17 (m, 1H), 7.08 (dd, *J* = 5.0, 1.3 Hz, 1H), 6.96 – 6.90 (m, 2H), 6.90 – 6.80 (m, 2H), 6.46-6.44 (m, 2H), 4.41 (s, 2H), 4.26 (s, 1H). ^{13}**C NMR** (75 MHz, $CDCl_3$): δ 143.5, 140.2, 128.9, 127.4, 127.2, 126.7, 126.3, 121.9, 121.3, 116.9, 111.6, 109.6, 43.4. **GCMS-EI** (70 eV): m/z (%) = 254 (M^+, 100), 253 (30), 169 (18), 157 (59), 135 (11), 97 (65), 77 (10).

***N*-((3-(1H-Pyrrol-1-yl)thiophen-2-yl)methyl)pyridin-2-amine (6m)**

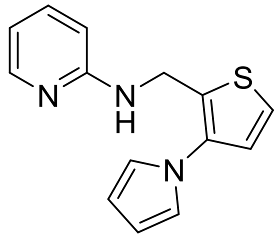

The compound was prepared as described in the general procedure A (m = 52 mg, 21% isolated yield). Colorless oil; ^{1}**H NMR** (300 MHz, $CDCl_3$): δ 8.13-8.11 (m , 1H), 7.43 – 7.37 (m, 1H), 7.20 (dd, *J* = 5.3, 0.7 Hz, 1H), 7.00 (d, *J* = 5.3 Hz, 1H), 6.90-6.89 (m, 2H), 6.64 – 6.60 (m, 1H), 6.36 – 6.31 (m, 3H), 4.83 (s, 1H), 4.64 (d, *J* = 5.8 Hz, 2H). ^{13}**C NMR** (75 MHz, $CDCl_3$): δ 157.8, 148.2, 137.5, 136.9, 132.2, 125.1, 123.7, 121.9, 113.8, 109.5, 107.6, 38.7. **HRMS** (EI, m/z) calcd. for $C_{14}H_{13}N_3S$ $(M+1)^+$ 256.09029; found 256.09076.

***N*-((1,3-Diphenyl-1H-pyrazol-4-yl)methyl)pyridin-2-amine (6n)**

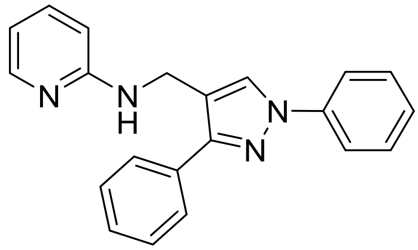

^{1}**H NMR** (300 MHz, $CDCl_3$): δ 8.15 – 8.13 (m, 1H), 7.98 (s, 1H), 7.80 – 7.71 (m, 4H), 7.47 – 7.37 (m, 6H), 7.30 – 7.25 (m, 1H), 6.65-6.60 (m, 1H), 6.41 (d, *J* = 8.4 Hz, 1H), 4.82 (s, 1H), 4.59 (d, *J* = 5.2 Hz, 2H). ^{13}**C NMR** (75 MHz, $CDCl_3$): δ 158.2, 151.2, 148.1, 139.9, 137.5, 133.0, 129.3, 128.6, 128.0, 127.7, 127.5, 126.3, 118.8, 113.4, 107.2, 37.3. **HRMS** (EI, m/z) calcd. for $C_{21}H_{18}N_4$ $(M+1)^+$ 327.16042; found 327.16058.

***N*-Octylpyridin-2-amine (6o)**[12]

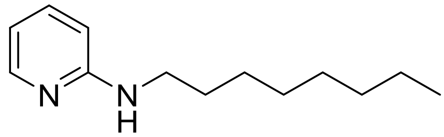

The compound was prepared as described in the general procedure **A** (m = 175 mg, 85% isolated yield). White solid;**^{1}H NMR** (300 MHz, $CDCl_3$): δ 8.05-8.03 (m, 1H), 7.39-7.33 (m, 1H), 6.52 (dd, *J* = 6.7, 5.4 Hz1H), 6.33 (d, *J* = 8.4 Hz, 1H), 4.69 (s, 1H), 3.23-3.17 (m, 2H), 1.63 – 1.53 (m, 2H), 1.29 – 1.23 (m, 10H), 0.99 – 0.54 (m, 3H). **^{13}C NMR** (75 MHz, $CDCl_3$): δ 159.0, 148.2, 137.3, 112.5, 106.3, 42.3, 31.8, 29.6, 29.4, 29.3, 27.1, 22.7, 14.1.

***N*-Hexylpyridin-2-amine (6p)**[13]

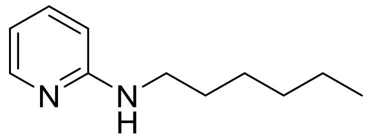

The compound was prepared as described in the general procedure **A** (m = 135 mg, 76% isolated yield). Colorless oil;**^{1}H NMR** (300 MHz, $CDCl_3$): δ 8.04-8.02 (m, 1H), 7.38-7.33 (m, 1H), 6.52-6.47 (m, 1H), 6.32 (d, *J* = 8.5 Hz, 1H), 4.70 (s, 1H), 3.23-3.16 (m, 2H), 1.62 – 1.52 (m, 2H), 1.40 – 1.24 (m, 6H), 0.88 – 0.83(m, 3H). **^{13}C NMR** (75 MHz, $CDCl_3$): δ 159.0, 148.2, 137.4, 112.5, 106.3, 42.3, 31.6, 29.5, 26.7, 22.6, 14.1.

***N*-Pentylpyridin-2-amine (6q)**

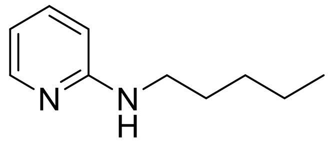

The compound was prepared as described in the general procedure **A** (m = 141 mg, 86% isolated yield White solid; **^{1}H NMR** (300 MHz, $CDCl_3$): δ 8.03 – 78.01 (m, 1H), 7.34 (ddd, *J* = 8.7, 7.1, 1.8 Hz, 1H), 6.50-6.45 (m, 1H), 6.31 (d, *J* = 8.5 Hz, 1H), 4.77 (s, 1H), 3.21-3.15 (m, 2H), 1.61-1.51 (m, 2H), 1.34 – 1.28 (m, 4H), 0.88 – 0.83 (m, 3H). **^{13}C NMR** (75 MHz, $CDCl_3$): δ 159.0, 148.1, 137.3, 112.4, 106.2, 42.2, 29.2, 22.4, 13.9. **GCMS-EI** (70 eV): m/z (%) = 164 (M^+, 15), 121 (28), 107 (100), 94 (32), 78 (33).

***N*-Butylpyridin-2-amine (6r)**

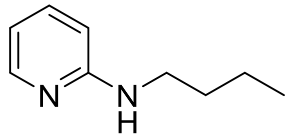

The compound was prepared as described in the general procedure **A** (m = 140 mg, 93% isolated yield). White solid; **^{1}H NMR** (300 MHz, $CDCl_3$): δ 8.03-8.00 (m, 1H), 7.33 (ddd, *J* = 8.8, 7.1, 1.9 Hz, 1H), 6.50-6.44 (m, 1H), 6.30 (d, *J* = 8.5 Hz, 1H), 4.78 (s, 1H), 3.34 – 3.00 (m, 2H), 1.62 – 1.46 (m, 2H), 1.44 – 1.29 (m, 2H), 0.89 (t, *J* = 7.3 Hz, 3H). **^{13}C NMR** (75

MHz, $CDCl_3$): δ 159.0, 148.0, 137.3, 112.4, 106.3, 41.9, 31.6, 20.2, 13.8. **GCMS-EI** (70 eV): m/z (%) = 150 (M^+, 18), 121 (30), 107 (100), 94 (30), 78 (39).

***N*-Ethylpyridin-2-amine (6s)**[14]

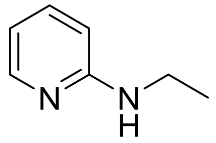

The compound was prepared as described in the general procedure **A** (m = 59 mg, 49% isolated yield). Yellow oil; **^{1}H NMR** (300 MHz, $CDCl_3$): δ 8.07 – 7.80 (m, 1H), 7.40 (ddd, *J* = 8.6, 7.0, 1.8 Hz, 1H), 6.57 – 6.50 (m, 1H), 6.39 (d, *J* = 8.5, 1H), 4.59 (br. s, 1H), 3.32-3.24 (m, 2H), 1.24 (t, *J* = 7.2 Hz, 3H). **^{13}C NMR** (75 MHz, $CDCl_3$): δ 158.8, 148.0, 137.6, 112.7, 106.5, 37.0, 14.9. **GCMS-EI** (70 eV): m/z (%) = 122 (M^+, 39), 107 (100), 94 (31), 78 (52), 67 (18).

(E)-N-Benzyl-4-styrylaniline (7a)

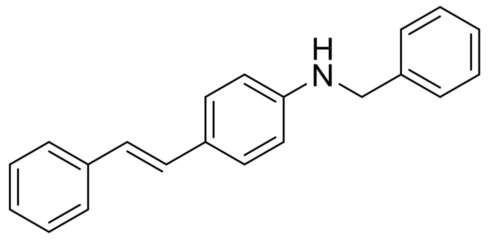

The compound was prepared as described in the general procedure **A** (m = 251 mg, 88% isolated yield). Yellow solid; **^{1}H NMR** (300 MHz, $CDCl_3$): δ 7.49 – 7.46 (m, 2H), 7.38 – 7.21 (m, 10H), 7.10 – 6.88 (m, 2H), 6.68 – 6.66 (m, 2H), 4.88 (s, 1H), 4.37 (s, 2H). **^{13}C NMR** (75 MHz, $CDCl_3$): δ 147.2, 138.8, 138.1, 128.8, 128.7, 127.8, 127.7, 127.5, 126.9, 126.2, 125.1, 113.6, 48.7. **GCMS-EI** (70 eV): m/z (%) = 285 (M^+, 100), 194 (32), 167 (21), 165 (19), 91 (28). **HRMS** (EI, m/z) calcd. for C21H19N (M)+ 285.15120; found 285.15112.

(*E*)-*N*-(4-Chlorobenzyl)-4-styrylaniline (7b)

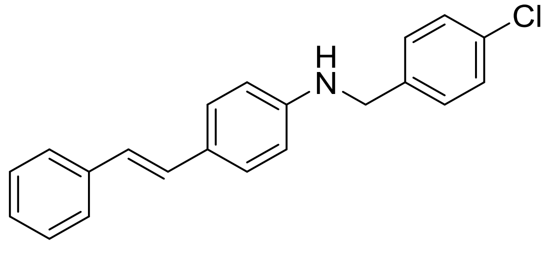

The compound was prepared as described in the general procedure **A** (m = 307 mg , 96% isolated yield). Yellow solid; **^{1}H NMR** (300 MHz,$CDCl_3$): δ 7.51 – 7.44 (m, 2H), 7.39 – 7.33 (m, 3H), 7.32 – 7.29 (m, 4H), 7.25 – 7.18 (m, 1H), 7.03 (d, *J* = 16.3 Hz, 1H), 6.91 (d, *J* = 16.3 Hz, 1H), 6.70 – 6.55 (m, 2H), 4.34 (s, 2H). **^{13}C NMR** (75 MHz, $CDCl_3$): δ 147.04, 138.08, 137.49, 133.21, 128.93, 128.73, 127.86, 127.00, 126.21, 125.16, 113.52, 47.91. **GCMS- EI**

(70 eV): m/z (%) = 319 (M^+, 100), 194 (51), 178 (12), 167 (27), 165 (24), 152 (17), 127 (12), 125 (30), 89 (13).

(*E*)-*N*-(3,5-Dimethylbenzyl)-4-styrylaniline (7c)

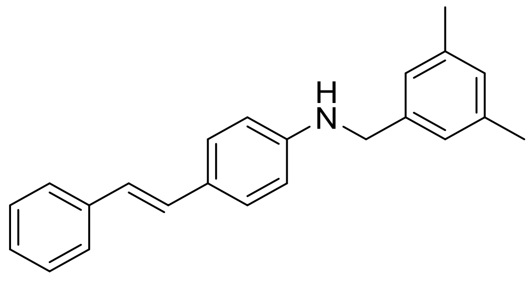

The compound was prepared as described in the general procedure **A** (m = 306 mg, 97% isolated yield). Yellow solid; **^{1}H NMR** (300 MHz, $CDCl_3$): δ 7.53 – 7.43 (m, 2H), 7.41 – 7.29 (m, 4H), 7.25 – 7.17 (m, 1H), 7.09 – 6.86 (m, 5H), 6.66 (dd, *J* = 9.0, 2.1 Hz, 2H), 4.28 (s, 2H), 2.32 (s, 6H). **^{13}C NMR** (75 MHz, $CDCl_3$): δ 138.9, 138.4, 138.2, 129.2, 128.9, 128.7, 127.9, 127.4, 126.9, 126.2, 125.6, 124.8, 113.4, 48.7, 21.4. **GCMS-EI** (70 eV): m/z (%) = 313 (M^+, 100), 194 (18), 167 (11), 165 (11), 119 (49).

(*E*)-*N*-(Benzo[*d*][1,3]dioxo-5-ylmethyl)-4-styrylaniline (7e)

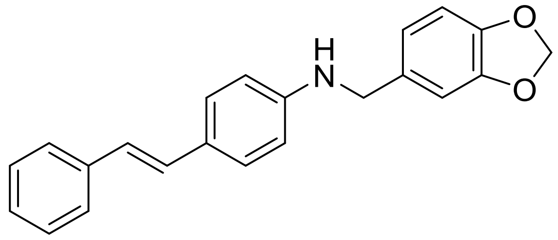

The compound was prepared as described in the general procedure **A** (m = 306 mg, 93% isolated yield). Yellow solid **^{1}H NMR** (300 MHz,$CDCl_3$): δ 7.54 – 7.43 (m, 2H), 7.41 – 7.28 (m, 4H), 7.25 – 7.17 (m, 1H), 7.04 (d, *J* = 16.3 Hz, 1H), 6.98 – 6.74 (m, 4H), 6.67 – 6.55 (m, 2H), 5.96 (s, 2H), 4.27 (s, 2H), 4.15 (s, 1H).**^{13}C NMR** (75 MHz, $CDCl_3$): δ 148.1, 147.8, 146.9, 138.2, 133.1, 128.9, 128.7, 127.9, 127.2, 126.9, 126.2, 124.8, 120.7, 113.1, 108.5, 108.1, 101.2, 48.2. **GCMS-EI** (70 eV): m/z (%) = 329 (M^+, 62), 165 (9), 135 (100), 77(13).

(*E*)-4-Styryl-*N*-(thiophen-2-ylmethyl)aniline (7d)

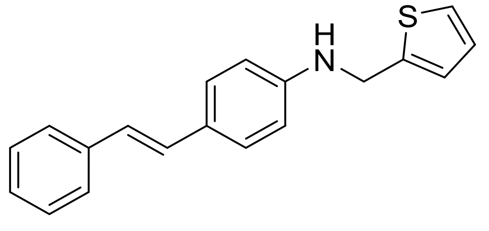

The compound was prepared as described in the general procedure **A** (m = 263 mg, 90% isolated yield). Yellow solid; **^{1}H NMR** (300 MHz,$CDCl_3$): δ 7.53 – 7.44 (m, 2H), 7.43 – 7.29 (m, 5H), 7.25 – 7.17 (m, 2H), 7.12 – 6.85 (m, 3H), 6.69 – 6.61 (m, 2H), 4.38 (s, 2H), 4.11 (s, 1H). **^{13}C NMR** (75 MHz,$CDCl_3$): δ 147.8, 140.3, 138.2, 128.9, 128.7, 127.9, 127.3, 127.2,

126.9, 126.4, 126.2, 124.8, 121.9, 113.1, 43.8. **GCMS-EI** (70 eV): m/z (%) = 291 (M^+, 100), 194 (35), 178 (10), 165 (17), 152 (12), 97 (49).

Indole (8)

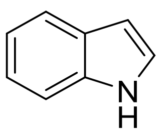

The compound was prepared as described in the general procedure **B** (m = 115 mg, 98% isolated yield).White crystals.**^{1}H NMR** (400 MHz, $CDCl_3$): δ 7.81 – 7.74 (s, 1H), 7.41 (d, *J* = 8.0 Hz, 1H), 7.34 – 7.28 (m, 1H), 7.28 – 7.22 (m, 1H), 7.21 – 7.16 (m, 1H), 6.69 – 6.62 (m, 1H). **^{13}C NMR** (101 MHz, $CDCl_3$): δ 135.8, 127.9, 124.3, 122.0, 120.8, 119.9, 111.2, 102.6. **GCMS-EI** (70 eV): m/z (%) = 117 (M^+, 100), 116 (9.66), 90 (41), 89 (34), 63 (14).

***N*-Methyl-*p*-toluidine (9a)[15]**

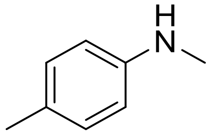

The compound was prepared as described in the general procedure **B** (m = 104 mg, 86% isolated yield). Yellow solid. **^{1}H NMR** (300 MHz, DMSO-d_6): δ 6.89 (d, *J* = 8.1 Hz, 2H), 6.44 (d, *J* = 8.4 Hz, 2H), 5.36 – 5.19 (m, 1H), 2.62 (d, *J* = 5.1 Hz, 3H), 2.14 (s, 3H). **^{13}C NMR** (75 MHz, DMSO-d_6): δ 147.9, 129.5, 124.1, 112.0, 30.2, 20.3. **GCMS-EI** (70 eV): m/z (%) = 121 (M^+, 70), 120 (100), 106 (8), 91 (18), 77 (10), 65 (7).

4-Ethyl-*N*-methylaniline (9b)

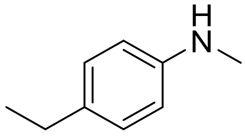

The compound was prepared as described in the general procedure **B** (m = 115 mg, 85% isolated yield). Brownish oil. **^{1}H NMR** (300 MHz, DMSO-d_6): δ = 7.03 – 6.80 (m, 2H), 6.60 – 6.35 (m, 2H), 5.53 – 5.05 (m, 1H), 2.63 (d, *J* = 4.1 Hz, 3H), 2.44 (q, *J* = 7.6 Hz, 2H), 1.10 (t, *J* = 7.6 Hz, 3H). **^{13}C NMR** (75 MHz, DMSO-d_6): δ = 148.1, 130.9, 128.3, 112.0, 30.2, 27.6, 16.4. **GCMS-EI** (70 eV): (EI, 70eV): m/z (%) = 135 (M^+, 37), 120 (100), 119 (9), 91 (8), 77 (9).

4-*tert*-Butyl-*N*-methylaniline (9e)[16]

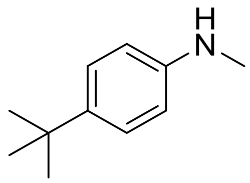

The compound was prepared as described in the general procedure **B** (m = 139 mg, 85% isolated yield). Yellow oil. **^{1}H NMR** (300 MHz, $CDCl_3$): δ 7.35 – 7.27 (m, 2H), 6.70 – 6.59 (m, 2H), 3.62 (s, 1H), 2.88 (s, 3H), 1.37 (s, 9H). **^{13}C NMR** (75 MHz, $CDCl_3$): δ 147.1, 140.1, 126.0, 112.3, 33.9, 31.7, 31.0. **GCMS-EI** (70 eV): m/z (%) = 163 (M^+, 21), 149 (11), 148 (100), 133 (15), 132 (11), 120 (25).

4-Methoxy-*N*-methylaniline (9c)[17]

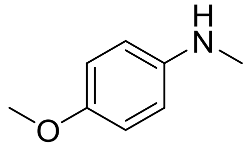

The compound was prepared as described in the general procedure **B** (m = 126 mg, 92% isolated yield). Yellow solid. **^{1}H NMR** (300 MHz, $CDCl_3$): δ = 6.91 – 6.75 (m, 2H), 6.69 – 6.53 (m, 2H), 3.77 (s, 3H), 3.46 (bs, 1H), 2.82 (s, 3H). **^{13}C NMR** (75 MHz, $CDCl_3$): δ = 152.1, 143.7, 114.9, 113.7, 55.9, 31.7. **GCMS-EI** (70 eV): m/z (%) = 137 (M^+, 51), 122 (100), 94 (48), 65 (17).

4-Ethoxy-*N*-methylaniline (9d)[18]

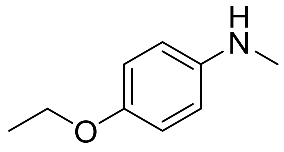

The compound was prepared as described in the general procedure **B** (m = 133 mg, 88% isolated yield). Yellow oil. **^{1}H NMR** (300 MHz, DMSO-d_6): δ 6.75 – 6.64 (m, 2H), 6.56 – 6.37 (m, 2H), 5.08 (s, 1H), 3.86 (q, J = 7.0 Hz, 2H), 2.61 (s, 3H), 1.25 (t, J = 7.0 Hz, 3H). **^{13}C NMR** (75 MHz, DMSO-d_6): δ 150.0, 144.5, 115.6, 112.8, 63.6, 30.7, 15.1. **GCMS-EI** (70 eV): m/z (%) = 151 (M^+, 35), 123 (16), 122 (100), 94 (20), 65 (8).

***3*-Iodo-*N*-methylaniline (9i)**[19]

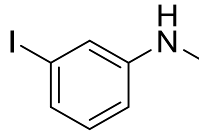

The compound was prepared as described in the general procedure **B** (m = 168 mg, 94% isolated yield). Yellow oil. **^{1}H NMR** (300 MHz, DMSO-d_6): δ 6.94 – 6.74 (m, 3H), 6.60 – 6.43 (m, 1H), 5.80 (q, J = 4.6 Hz, 1H), 2.62 (d, J = 5.1 Hz, 3H). **^{13}C NMR** (75 MHz, DMSO-

d$_6$): δ 151.5, 131.0, 124.0, 119.8, 111.3, 95.7, 29.6. **GCMS-EI** (70 eV): m/z (%) = 233 (M$^+$, 100), 232 (22), 106 (17), 105 (8), 104 (9), 79 (12), 77 (18).

***N*-Methyl-3-vinylaniline (9h)**

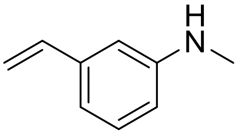

The compound was prepared as described in the general procedure **B** (m = 89 mg, 67% isolated yield). Yellow oil. **^{1}H NMR** (300 MHz,CDCl$_3$): δ 7.19 (t, *J* = 7.7 Hz, 1H), 6.94 – 6.79 (m, 1H), 6.79 – 6.63 (m, 2H), 6.63 – 6.53 (m, 1H), 5.76 (dd, *J* = 17.6, 1.0 Hz, 1H), 5.25 (dd, *J* = 10.9, 1.0 Hz, 1H), 3.68 (s, 1H), 2.87 (s, 3H). **^{13}C NMR** (75 MHz,CDCl$_3$): δ 149.5, 138.6, 137.4, 129.4, 115.7, 113.5, 112.3, 110.1, 30.9. **GCMS-EI** (70 eV): m/z (%) = 133 (M$^+$, 100), 132 (99), 117 (11), 104 (12), 103 (12), 77 (17).

4-Bromo-*N*-methylaniline (9f)[20]

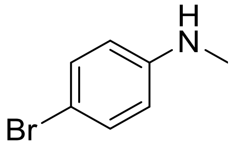

The compound was prepared as described in the general procedure **B** (m = 162 mg, 87% isolated yield). Brownish oil. **^{1}H NMR** (300 MHz,CDCl$_3$): δ 7.39 – 7.14 (m, 2H), 6.59 – 6.38 (m, 2H), 3.70 (s, 1H), 2.81 (s, 3H). **^{13}C NMR** (75 MHz, CDCl$_3$): δ 148.3, 131.9, 114.1, 108.9, 30.8. **GCMS-EI** (70 eV): m/z (%) = 186 (M$^+$, 85), 185 (100), 106 (11), 105 (37), 104 (26), 91 (19), 79 (15), 78 (17), 77 (28), 76 (15), 75 (16), 74 (17), 65 (11), 64 (12), 63 (22), 51 (13), 50 (20).

***N*-Methylpyridin-3-amine (9g)[17]**

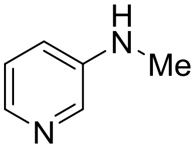

The compound was prepared as described in the general procedure **B** (m = 96 mg, 89% isolated yield). Yellow oil. **^{1}H NMR** (300 MHz,CDCl$_3$): δ 8.00 (d, *J* = 2.6 Hz, 1H), 7.92 (d, *J* = 4.2 Hz, 1H), 7.06 (dd, *J* = 8.3, 4.6 Hz, 1H), 6.94 – 6.73 (m, 1H), 3.87 (s, 1H), 2.81 (s, 3H). **^{13}C NMR** (75 MHz, CDCl$_3$): δ 145.3, 138.4, 135.7, 123.8, 118.1, 30.3. **GCMS-EI** (70 eV): m/z (%) = 108 (M$^+$, 100), 107 (97), 80 (16), 78 (22), 66 (12), 52 (14), 51 (20), 50 (14), 39 (24), 38 (11).

2-Iodo-*N*-methylaniline (9j)[21]

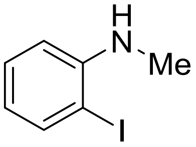

The compound was prepared as described in the general procedure **B** (m = 133 mg, 57% isolated yield). Yellow oil. **^{1}H NMR** (300 MHz, $CDCl_3$): δ 7.68 (dd, *J* = 7.8, 1.5 Hz, 1H), 7.33 – 7.17 (m, 1H), 6.58 (dd, *J* = 8.2, 1.4 Hz, 1H), 6.52 – 6.42 (m, 1H), 4.27 (s, 1H), 2.90 (s, 3H). **^{13}C NMR** (75 MHz,$CDCl_3$): δ 148.2, 138.9, 129.5, 118.6, 110.1, 85.2, 31.1. **GCMS-EI** (70 eV): m/z (%) = 233 (M^+, 100), 232 (34), 127 (28), 106 (16), 105 (12), 104 (15), 79 (15), 78 (12), 77 (32), 51 (10).

2-Ethyl-*N*-methylaniline (9k)

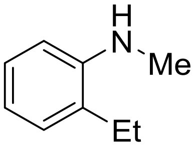

The compound was prepared as described in the general procedure **B** (m = 99 mg, 73% isolated yield). Yellow oil. **^{1}H NMR** (300 MHz, $CDCl_3$): δ 7.18 (td, *J* = 7.8, 1.6 Hz, 1H), 7.09 (dd, *J* = 7.4, 1.5 Hz, 1H), 6.74 (td, *J* = 7.4, 1.0 Hz, 1H), 6.69 – 6.61 (m, 1H), 3.88 (s, 1H), 2.91 (s, 3H), 2.50 (q, *J* = 7.5 Hz, 2H), 1.26 (t, *J* = 7.5 Hz, 3H). **^{13}C NMR** (75 MHz, $CDCl_3$): δ 127.8, 127.2, 117.3, 109.8, 31.1, 23.9, 13.0. **GCMS-EI** (70 eV): m/z (%) = 135 (M^+, 46), 120 (100), 119 (12), 118 (13), 91 (25), 77 (13).

***N*-Methyl-2,3-dihydrobenzo[b][1,4]dioxin-6-amine (9l)**

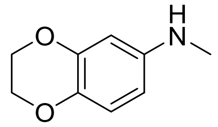

The compound was prepared as described in the general procedure **B** (m = 143 mg, 86% isolated yield). Brown oil. **^{1}H NMR** (400 MHz, $CDCl_3$): δ = 6.73 (d, *J* = 7.8 Hz, 1H), 6.27 – 6.03 (m, 2H), 4.44 – 3.97 (m, 4H), 3.48 (s, 1H), 2.80 (s, 3H). **^{13}C NMR** (101 MHz, $CDCl_3$): δ = 144.7, 144.1, 135.6, 117.6, 106.5, 101.1, 64.8, 64.2, 31.5. **GCMS-EI** (70 eV): m/z (%) = 165 (M^+, 100) 110 (13), 109 (91), 108 (99), 81 (12), 80 (26), 55 (12), 52 (12), 51 (11).

***N*-Methyl-9*H*-fluoren-2-amine (9m)**

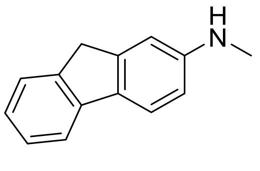

The compound was prepared as described in the general procedure **B** (m = 137 mg, 70% isolated yield).Yellow solid. **^{1}H NMR** (400 MHz, $CDCl_3$): δ 7.64 (dt, *J* = 7.7, 0.9 Hz, 1H),

7.60 (d, *J* = 8.2 Hz, 1H), 7.48 (dt, *J* = 7.5, 1.0 Hz, 1H), 7.37 – 7.29 (m, 1H), 7.19 (td, *J* = 7.4, 1.1 Hz, 1H), 6.82 (q, *J* = 1.1 Hz, 1H), 6.65 (dd, *J* = 8.2, 2.2 Hz, 1H), 3.84 (s, 3H; N*H* + Ar-CH_2-Ar), 2.91 (s, 3H). **^{13}C NMR** (101 MHz, $CDCl_3$): δ 149.0, 145.3, 142.5, 142.3, 131.9, 126.7, 124.9, 124.8, 120.7, 118.5, 111.8, 108.9, 37.1, 31.2 . **GCMS-EI** (70 eV): m/z (%) = 196 (14), 195 (M^+, 100), 194 (59), 180 (15), 165 (30), 152 (21).

N-Methyl-2-(1-H-pyrrol-1-yl)aniline (9n)

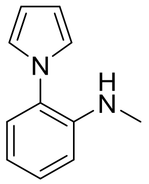

The compound was prepared as described in the general procedure **B** (m = 90 mg, 52% isolated yield). White solid. **^{1}H NMR** (400 MHz,$CDCl_3$): δ 7.32 (td, *J* = 7.8, 1.6 Hz, 1H), 7.18 (dd, *J* = 7.7, 1.4 Hz, 1H), 6.84 (t, *J* = 2.1 Hz, 2H), 6.81 – 6.72 (m, 2H), 6.39 (t, *J* = 2.1 Hz, 2H), 3.88 (s, 1H), 2.83 (s, 3H). **^{13}C NMR** (101 MHz, $CDCl_3$): δ 144.9, 129.1, 127.3, 127.0, 122.0, 116.4, 110.6, 109.4, 30.4. **GCMS-EI** (70 eV): m/z (%) = 172 (M^+, 65), 171 (100), 156 (14), 145 (14), 77 (12).

Supplementary References

[1]Elangovan, S. *et al.* Selective Catalytic Hydrogenations of Nitriles, Ketones and Aldehydes by Well-Defined Manganese Pincer Complexes. *J. Am. Chem. Soc.*, DOI: 10.1021/jacs.6b03709 (2016).

[2] Wetzel, A. *et al.* Selective alkylation of amines with alcohols by Cp*–Iridium(III) half-sandwich complexes. *Org. lett.* **15**, 266-269 (2013).

[3] Cui, X., Deng, Y. & Shi, F. Reductive *N*-alkylation of nitro compounds to *N*-Alkyl and *N,N*-dialkyl amines with glycerol as the hydrogen source. *ACS Catal.* **3**, 808-811 (2013).

[4] Xu, Q. *et al.* Discovery and mechanistic studies of a general air-promoted metal-catalyzed aerobic *N*- alkylation reaction of amides and amines with alcohols. *J. Org. Chem.,* **76**, 5759–5773 (2011).

[5] Zhou, W., Fan, M., Yin, J., Jiang, Y. & Ma, D. CuI/Oxalic diamide catalyzed coupling reaction of (hetero)aryl chlorides and amines. *J. Am. Chem. Soc.* **137**, 11942-11945 (2015).

[6] Yang, H., Cui, X., Dai, X., Deng, Y. & Shi, F. Carbon-catalysed reductive hydrogen atom transfer reactions. *Nat Commun* **6**, 6478 (2015).

[7] Nguyen, Q. P. B. & Kim, T. H. S-Benzyl isothiouronium chloride as a recoverable organocatalyst for the direct reductive amination of aldehydes. *Tetrahedron Lett.* **52**, 5004-5007 (2011).

[8] Rosler, S., Ertl, M., Irrgang, T. & Kempe, R. Cobalt-catalyzed alkylation of aromatic amines by alcohols. *Angew. Chem. Int. Ed.*, **54**, 15046–15050 (2015).

[9] Park, J. W. & Chung, Y. K. Hydrogen-free cobalt–rhodium heterobimetallic nanoparticle-catalyzed reductive amination of aldehydes and ketones with amines and nitroarenes in the presence of carbon monoxide and water. *ACS Catal.* **5**, 4846-4850 (2015).

[10] Corre, Y. *et al.* Efficient hydrosilylation of imines using catalysts based on iridium(III) metallacycles. *Catal. Sci. Technol.* **5**, 1452-1458 (2015).

[11] Sreedhar, B., Reddy, P. S. & Devi, D. K. Direct one-pot reductive amination of aldehydes with nitroarenes in a domino fashion: catalysis by gum-acacia-stabilized palladium nanoparticles. *J. Org. Chem.* **74**, 8806-8809 (2009).

[12] Wheaton, C. A., Bow, J.-P. J. & Stradiotto, M. New phosphine-functionalized NHC ligands: discovery of an effective catalyst for the room-temperature amination of aryl chlorides with primary and secondary amines. *Organometallics* **32**, 6148-6161 (2013).

[13] Doherty, S. *et al.* ortho,ortho′-Substituted KITPHOS Monophosphines: highly efficient ligands for palladium-catalyzed C-C and C-N bond formation. *Adv. Synth. Catal.* **352**, 201-211 (2010).

[14] Krein, D. M. & Lowary, T. L. A Convenient Synthesis of 2-(Alkylamino)pyridines. *J. Org. Chem.* **67**, 4965-4967 (2002).

[15] Cui, X. *et al.* Ruthenium-Catalyzed Nitro and Nitrile Compounds Coupling with Alcohols: Alternative Route for N-Substituted Amine Synthesis *Chem. Eur. J.* **17**, 2587-2591 (2011).

[16] Arredondo, Y. *et. al.* Preparation, Antimicrobial Evaluation, and Mutagenicity of [2-Hydroxyaryl]- [1-methyl-5-nitro-lH-2-imidazolyl]methanols, [5-tert-Butyl-2-methylaminophenyl] - [1-methyl-5-ni tro- 1H-2-imidazolyl]methanol, and [2-Hydroxyaryl]-[1-methyl-5-nitro-1H-2-imidazolyl] ketones *Bioorg. Med. Chem.* **5**, 1959-1968 (1997).

[17] Fors, B. P. *et. al.* A Highly Active Catalyst for Pd-Catalyzed Amination Reactions: Cross-Coupling Reactions Using Aryl Mesylates and the Highly Selective Monoarylation of Primary Amines Using Aryl Chlorides *J. Am. Chem. Soc.* **130**, 13552-13554 (2008).

[18] Gangee, A *et. al.* Synthesis of N^4-(substituted phenyl)-N^4-alkyl/desalkyl-9H-pyrimido[4,5-b]indole-2,4-diamines and identification of new microtubule disrupting compounds that are effective against multidrug resistant cells *Bioorg. Med. Chem* **21**, 891-902 (2013).

[19] Padmanabhan, S., Reddy, N. L., & Durant, G. J. A Convenient One Pot Procedure for N-Methylation of Aromatic Amines Using Trimethyl Orthoformate *Synth. Commun.* **27**, 691-699 (1997).

[20] Maibunkaew, T. *et al.* Practical and Metal-Free Electrophilic Aromatic Halogenation by Interhalogen Compounds Generated In Situ from N-Halosuccinimide and Catalytic TMSCl *Synlett* 1769-1775 (2014).

[21] Zhou, P.-X. *et al.* Palladium-catalyzed insertion of α-diazocarbonyl compounds for the synthesis of cyclic amino esters *Chem. Commun.***49**, 561-563 (2013).
